# Posicionamento sobre Doença Isquêmica do Coração – A Mulher no Centro do Cuidado – 2023

**DOI:** 10.36660/abc.20230303

**Published:** 2023-07-27

**Authors:** Gláucia Maria Moraes de Oliveira, Maria Cristina Costa de Almeida, Daniela do Carmo Rassi, Érika Olivier Vilela Bragança, Lidia Zytynski Moura, Magaly Arrais, Milena dos Santos Barros Campos, Viviana Guzzo Lemke, Walkiria Samuel Avila, Alexandre Jorge Gomes de Lucena, André Luiz Cerqueira de Almeida, Andréa Araujo Brandão, Andrea Dumsch de Aragon Ferreira, Andreia Biolo, Ariane Vieira Scarlatelli Macedo, Breno de Alencar Araripe Falcão, Carisi Anne Polanczyk, Carla Janice Baister Lantieri, Celi Marques-Santos, Claudia Maria Vilas Freire, Denise Pellegrini, Elizabeth Regina Giunco Alexandre, Fabiana Goulart Marcondes Braga, Fabiana Michelle Feitosa de Oliveira, Fatima Dumas Cintra, Isabela Bispo Santos da Silva Costa, José Sérgio Nascimento Silva, Lara Terra F. Carreira, Lucelia Batista Neves Cunha Magalhães, Luciana Diniz Nagem Janot de Matos, Marcelo Heitor Vieira Assad, Marcia M. Barbosa, Marconi Gomes da Silva, Maria Alayde Mendonça Rivera, Maria Cristina de Oliveira Izar, Maria Elizabeth Navegantes Caetano Costa, Maria Sanali Moura de Oliveira Paiva, Marildes Luiza de Castro, Marly Uellendahl, Mucio Tavares de Oliveira, Olga Ferreira de Souza, Ricardo Alves da Costa, Ricardo Quental Coutinho, Sheyla Cristina Tonheiro Ferro da Silva, Sílvia Marinho Martins, Simone Cristina Soares Brandão, Susimeire Buglia, Tatiana Maia Jorge de Ulhôa Barbosa, Thais Aguiar do Nascimento, Thais Vieira, Valquíria Pelisser Campagnucci, Antonio Carlos Palandri Chagas

**Affiliations:** 1 Universidade Federal do Rio de Janeiro Rio de Janeiro RJ Brasil 1Universidade Federal do Rio de Janeiro (UFRJ), Rio de Janeiro, RJ – Brasil; 2 Centro Universitário de Belo Horizonte Belo Horizonte MG Brasil Centro Universitário de Belo Horizonte, Belo Horizonte, MG – Brasil; 3 Faculdade de Medicina Universidade Federal de Goiás Goiânia GO Brasil Faculdade de Medicina da Universidade Federal de Goiás (UFG), Goiânia, GO – Brasil; 4 RitmoCheck São José dos Campos SP Brasil RitmoCheck, São José dos Campos, SP – Brasil; 5 Pontifícia Universidade Católica do Paraná Curitiba PR Brasil Pontifícia Universidade Católica do Paraná (PUC-PR), Curitiba, PR – Brasil; 6 Hospital do Coração São Paulo SP Brasil Hospital do Coração (HCor), São Paulo, SP – Brasil; 7 Hospital Universitário de Sergipe Aracaju SE Brasil Hospital Universitário de Sergipe, Aracaju, SE – Brasil; 8 Cardiocare – Clínica Cardiológica Ltda Curitiba PR Brasil Cardiocare – Clínica Cardiológica Ltda., Curitiba, PR – Brasil; 9 Hospital das Clínicas Faculdade de Medicina Universidade de São Paulo São Paulo SP Brasil Instituto do Coração (Incor) do Hospital das Clínicas da Faculdade de Medicina da Universidade de São Paulo (FMUSP), São Paulo, SP – Brasil; 10 Hospital Agamenon Magalhães Recife PE Brasil Hospital Agamenon Magalhães, Recife, PE – Brasil; 11 Santa Casa de Misericórdia de Feira de Santana Feira de Santana BA Brasil Santa Casa de Misericórdia de Feira de Santana, Feira de Santana, BA – Brasil; 12 Universidade do Estado do Rio de Janeiro Rio de Janeiro RJ Brasil Universidade do Estado do Rio de Janeiro (UERJ), Rio de Janeiro, RJ – Brasil; 13 Instituto de Neuro e Cardiologia de Curitiba Curitiba PR Brasil Instituto de Neuro e Cardiologia de Curitiba (INC), Curitiba, PR – Brasil; 14 Universidade Federal do Rio Grande do Sul Porto Alegre RS Brasil Universidade Federal do Rio Grande do Sul (UFRGS), Porto Alegre, RS – Brasil; 15 Santa Casa de Misericórdia de São Paulo São Paulo SP Brasil Santa Casa de Misericórdia de São Paulo, São Paulo, SP – Brasil; 16 Hospital de Messejana Fortaleza CE Brasil Hospital de Messejana, Fortaleza, CE – Brasil; 17 Hospital de Clínicas UFRGS Porto Alegre RS Brasil Hospital de Clínicas da UFRGS, Porto Alegre, RS – Brasil; 18 Centro Universitário Faculdade de Medicina ABC Santo André SP Brasil Centro Universitário Faculdade de Medicina ABC, Santo André, SP – Brasil; 19 Universidade Tiradentes Aracaju SE Brasil Universidade Tiradentes (UNIT), Aracaju, SE – Brasil; 20 Hospital São Lucas Rede D’Or São Luis Aracaju SE Brasil Hospital São Lucas Rede D’Or São Luis, Aracaju, SE – Brasil; 21 Empresa Brasileira de Serviços Hospitalares Belo Horizonte MG Brasil Empresa Brasileira de Serviços Hospitalares (EBSERH), Belo Horizonte, MG – Brasil; 22 Hospital São Lucas da Pontifícia Universidade Católica do Rio Grande do Sul Porto Alegre RS Brasil Hospital São Lucas da Pontifícia Universidade Católica do Rio Grande do Sul (PUC-RS), Porto Alegre, RS – Brasil; 23 Instituto do Coração de Pernambuco Recife PE Brasil Instituto do Coração de Pernambuco, Recife, PE – Brasil; 24 Universidade Federal de São Paulo São Paulo SP Brasil Universidade Federal de São Paulo (UNIFESP), São Paulo, SP – Brasil; 25 Instituto do Câncer do Estado de São Paulo São Paulo SP Brasil Instituto do Câncer do Estado de São Paulo, São Paulo, SP – Brasil; 26 Pronto Socorro Cardiológico de Pernambuco Universidade de Pernambuco Recife PE Brasil Pronto Socorro Cardiológico de Pernambuco da Universidade de Pernambuco (PROCAPE/UPE), Recife, PE – Brasil; 27 Cardiologia Nuclear de Curitiba Curitiba PR Brasil Cardiologia Nuclear de Curitiba, Curitiba, PR – Brasil; 28 Hospital Pilar Curitiba PR Brasil Hospital Pilar, Curitiba, PR – Brasil; 29 Faculdade de Medicina Universidade Federal da Bahia Salvador BA Brasil Faculdade de Medicina da Universidade Federal da Bahia (UFBA), Salvador, BA – Brasil; 30 Hospital Israelita Albert Einstein São Paulo SP Brasil Hospital Israelita Albert Einstein, São Paulo, SP – Brasil; 31 Instituto Nacional de Cardiologia Rio de Janeiro RJ Brasil Instituto Nacional de Cardiologia (INC), Rio de Janeiro, RJ – Brasil; 32 Hospital Socor Belo Horizonte MG Brasil Hospital Socor, Belo Horizonte, MG – Brasil; 33 SPORTIF – Clínica do Exercício e do Esporte Belo Horizonte MG Brasil SPORTIF – Clínica do Exercício e do Esporte, Belo Horizonte, MG – Brasil; 34 Universidade Federal de Alagoas Maceió AL Brasil Universidade Federal de Alagoas (UFAL), Maceió, AL – Brasil; 35 Centro Universitário do Estado Pará Belém PA Brasil Centro Universitário do Estado Pará (CESUPA), Belém, PA – Brasil; 36 INTERVE Natal RN Brasil INTERVE, Natal, RN – Brasil; 37 Faculdade IPEMED de Ciências Médicas Belo Horizonte MG Brasil Faculdade IPEMED de Ciências Médicas, Belo Horizonte, MG – Brasil; 38 DASA – Diagnósticos da América S/A São Paulo SP Brasil DASA – Diagnósticos da América S/A, São Paulo, SP – Brasil; 39 Rede D’Or Rio de Janeiro RJ Brasil Rede D’Or, Rio de Janeiro, RJ – Brasil; 40 Instituto Dante Pazzanese de Cardiologia São Paulo SP Brasil Instituto Dante Pazzanese de Cardiologia, São Paulo, SP – Brasil; 41 Faculdade de Ciências Médicas Universidade de Pernambuco Recife PE Brasil Faculdade de Ciências Médicas da Universidade de Pernambuco (UPE), Recife, PE – Brasil; 42 Hospital Universitário Osvaldo Cruz Universidade de Pernambuco Recife PE Brasil Hospital Universitário Osvaldo Cruz da Universidade de Pernambuco (UPE), Recife, PE – Brasil; 43 CEMISE Oncoclínicas Aracaju SE Brasil CEMISE Oncoclínicas, Aracaju, SE – Brasil; 44 Hospital das Clínicas Universidade Federal de Pernambuco Recife PE Brasil Hospital das Clínicas da Universidade Federal de Pernambuco (UFPE), Recife, PE – Brasil; 45 CARDIOCENTRO Cirurgia Cardiovascular Brasília DF Brasil CARDIOCENTRO Cirurgia Cardiovascular, Brasília, DF – Brasil; 46 Hospital de Base do Distrito Federal Brasília DF Brasil Hospital de Base do Distrito Federal, Brasília, DF – Brasil; 47 Cardio Ritmo Serviços Médicos Salvador BA Brasil Cardio Ritmo Serviços Médicos, Salvador, BA – Brasil; 48 Rede D’Or Aracaju SE Brasil Rede D’Or, Aracaju, SE – Brasil; 49 Hospital Universitário Universidade Federal de Sergipe Aracaju SE Brasil Hospital Universitário da Universidade Federal de Sergipe (UFS), Aracaju, SE – Brasil; 50 Irmandade da Santa Casa de São Paulo São Paulo SP Brasil Irmandade da Santa Casa de São Paulo, São Paulo, SP – Brasil

**Table:** 

Posicionamento sobre Doença Isquêmica do Coração – A Mulher no Centro do Cuidado – 2023	
O relatório abaixo lista as declarações de interesse conforme relatadas à SBC pelos especialistas durante o período de desenvolvimento deste posicionamento, 2022/2023.	
Especialista	Tipo de relacionamento com a indústria
Alexandre Jorge Gomes de Lucena	Declaração financeira A - Pagamento de qualquer espécie e desde que economicamente apreciáveis, feitos a (i) você, (ii) ao seu cônjuge/ companheiro ou a qualquer outro membro que resida com você, (iii) a qualquer pessoa jurídica em que qualquer destes seja controlador, sócio, acionista ou participante, de forma direta ou indireta, recebimento por palestras, aulas, atuação como proctor de treinamentos, remunerações, honorários pagos por participações em conselhos consultivos, de investigadores, ou outros comitês, etc. Provenientes da indústria farmacêutica, de órteses, próteses, equipamentos e implantes, brasileiras ou estrangeiras: - Cardiopapers; Afya.
André Luiz Cerqueira de Almeida	Declaração financeira A - Pagamento de qualquer espécie e desde que economicamente apreciáveis, feitos a (i) você, (ii) ao seu cônjuge/ companheiro ou a qualquer outro membro que resida com você, (iii) a qualquer pessoa jurídica em que qualquer destes seja controlador, sócio, acionista ou participante, de forma direta ou indireta, recebimento por palestras, aulas, atuação como proctor de treinamentos, remunerações, honorários pagos por participações em conselhos consultivos, de investigadores, ou outros comitês, etc. Provenientes da indústria farmacêutica, de órteses, próteses, equipamentos e implantes, brasileiras ou estrangeiras: - Boston Scientific: Palestrante Prótese.
Andréa Araujo Brandão	Declaração financeira A - Pagamento de qualquer espécie e desde que economicamente apreciáveis, feitos a (i) você, (ii) ao seu cônjuge/ companheiro ou a qualquer outro membro que resida com você, (iii) a qualquer pessoa jurídica em que qualquer destes seja controlador, sócio, acionista ou participante, de forma direta ou indireta, recebimento por palestras, aulas, atuação como proctor de treinamentos, remunerações, honorários pagos por participações em conselhos consultivos, de investigadores, ou outros comitês, etc. Provenientes da indústria farmacêutica, de órteses, próteses, equipamentos e implantes, brasileiras ou estrangeiras: - Servier: Triplixan; Daiichi Sankyo: Benicar; Libbs: Venzer. B - Financiamento de pesquisas sob sua responsabilidade direta/pessoal (direcionado ao departamento ou instituição) provenientes da indústria farmacêutica, de órteses, próteses, equipamentos e implantes, brasileiras ou estrangeiras. - Servier: Hipertensão Arterial. Outros relacionamentos Financiamento de atividades de educação médica continuada, incluindo viagens, hospedagens e inscrições para congressos e cursos, provenientes da indústria farmacêutica, de órteses, próteses, equipamentos e implantes, brasileiras ou estrangeiras: - Servier: Hipertensão Arterial.
Andrea Dumsch de Aragon Ferreira	Nada a ser declarado
Andreia Biolo	Declaração financeira B - Financiamento de pesquisas sob sua responsabilidade direta/pessoal (direcionado ao departamento ou instituição) provenientes da indústria farmacêutica, de órteses, próteses, equipamentos e implantes, brasileiras ou estrangeiras. - Alnylam Pharmaceuticals: Amiloidose.
Antonio Carlos Palandri Chagas	Declaração financeira A - Pagamento de qualquer espécie e desde que economicamente apreciáveis, feitos a (i) você, (ii) ao seu cônjuge/ companheiro ou a qualquer outro membro que resida com você, (iii) a qualquer pessoa jurídica em que qualquer destes seja controlador, sócio, acionista ou participante, de forma direta ou indireta, recebimento por palestras, aulas, atuação como proctor de treinamentos, remunerações, honorários pagos por participações em conselhos consultivos, de investigadores, ou outros comitês, etc. Provenientes da indústria farmacêutica, de órteses, próteses, equipamentos e implantes, brasileiras ou estrangeiras: - Novo Nordisk; Viatris; Instituto Vita Nova. Outros relacionamentos Financiamento de atividades de educação médica continuada, incluindo viagens, hospedagens e inscrições para congressos e cursos, provenientes da indústria farmacêutica, de órteses, próteses, equipamentos e implantes, brasileiras ou estrangeiras: - Novo Nordisk.
Ariane Vieira Scarlatelli Macedo	Declaração financeira A - Pagamento de qualquer espécie e desde que economicamente apreciáveis, feitos a (i) você, (ii) ao seu cônjuge/ companheiro ou a qualquer outro membro que resida com você, (iii) a qualquer pessoa jurídica em que qualquer destes seja controlador, sócio, acionista ou participante, de forma direta ou indireta, recebimento por palestras, aulas, atuação como proctor de treinamentos, remunerações, honorários pagos por participações em conselhos consultivos, de investigadores, ou outros comitês, etc. Provenientes da indústria farmacêutica, de órteses, próteses, equipamentos e implantes, brasileiras ou estrangeiras: - Bayer: Anticoagulação e insuficiência cardíaca; Pfizer: Anticoagulação e amiloidose; Jannsen: Leucemia. Outros relacionamentos Financiamento de atividades de educação médica continuada, incluindo viagens, hospedagens e inscrições para congressos e cursos, provenientes da indústria farmacêutica, de órteses, próteses, equipamentos e implantes, brasileiras ou estrangeiras: - Bayer: Insuficiência cardíaca.
Breno de Alencar Araripe Falcão	Declaração financeira A - Pagamento de qualquer espécie e desde que economicamente apreciáveis, feitos a (i) você, (ii) ao seu cônjuge/ companheiro ou a qualquer outro membro que resida com você, (iii) a qualquer pessoa jurídica em que qualquer destes seja controlador, sócio, acionista ou participante, de forma direta ou indireta, recebimento por palestras, aulas, atuação como proctor de treinamentos, remunerações, honorários pagos por participações em conselhos consultivos, de investigadores, ou outros comitês, etc. Provenientes da indústria farmacêutica, de órteses, próteses, equipamentos e implantes, brasileiras ou estrangeiras: - Edwards Lifescience: Proctor de TAVI e TMVR; Medtronic: Proctor de TAVI; Boston Scientific: Proctor de CTO PCI.
Carisi Anne Polanczyk	Nada a ser declarado
Carla Janice Baister Lantieri	Nada a ser declarado
Celi Marques-Santos	Nada a ser declarado
Claudia Maria Vilas Freire	Nada a ser declarado
Daniela do Carmo Rassi	Nada a ser declarado
Denise Pellegrini	Nada a ser declarado
Elizabeth Regina Giunco Alexandre	Declaração financeira A - Pagamento de qualquer espécie e desde que economicamente apreciáveis, feitos a (i) você, (ii) ao seu cônjuge/ companheiro ou a qualquer outro membro que resida com você, (iii) a qualquer pessoa jurídica em que qualquer destes seja controlador, sócio, acionista ou participante, de forma direta ou indireta, recebimento por palestras, aulas, atuação como proctor de treinamentos, remunerações, honorários pagos por participações em conselhos consultivos, de investigadores, ou outros comitês, etc. Provenientes da indústria farmacêutica, de órteses, próteses, equipamentos e implantes, brasileiras ou estrangeiras: - Lilly: Trulicity, Jardiance, Glyxambi. Outros relacionamentos Financiamento de atividades de educação médica continuada, incluindo viagens, hospedagens e inscrições para congressos e cursos, provenientes da indústria farmacêutica, de órteses, próteses, equipamentos e implantes, brasileiras ou estrangeiras: - Novo Nordisk: Ozempic.
Érika Olivier Vilela Bragança	Nada a ser declarado
Fabiana Goulart Marcondes Braga	Declaração financeira A - Pagamento de qualquer espécie e desde que economicamente apreciáveis, feitos a (i) você, (ii) ao seu cônjuge/ companheiro ou a qualquer outro membro que resida com você, (iii) a qualquer pessoa jurídica em que qualquer destes seja controlador, sócio, acionista ou participante, de forma direta ou indireta, recebimento por palestras, aulas, atuação como proctor de treinamentos, remunerações, honorários pagos por participações em conselhos consultivos, de investigadores, ou outros comitês, etc. Provenientes da indústria farmacêutica, de órteses, próteses, equipamentos e implantes, brasileiras ou estrangeiras: - Novartis: Palestras; AstraZeneca: Palestras e Conselho Consultivo; Boehringer: Conselho Consultivo.
Fabiana Michelle Feitosa de Oliveira	Nada a ser declarado
Fatima Dumas Cintra	Nada a ser declarado
Gláucia Maria Moraes de Oliveira	Nada a ser declarado
Isabela Bispo Santos da Silva Costa	Nada a ser declarado
José Sérgio Nascimento Silva	Nada a ser declarado
Lara Terra F. Carreira	Nada a ser declarado
Lidia Zytynski Moura	Declaração financeira A - Pagamento de qualquer espécie e desde que economicamente apreciáveis, feitos a (i) você, (ii) ao seu cônjuge/ companheiro ou a qualquer outro membro que resida com você, (iii) a qualquer pessoa jurídica em que qualquer destes seja controlador, sócio, acionista ou participante, de forma direta ou indireta, recebimento por palestras, aulas, atuação como proctor de treinamentos, remunerações, honorários pagos por participações em conselhos consultivos, de investigadores, ou outros comitês, etc. Provenientes da indústria farmacêutica, de órteses, próteses, equipamentos e implantes, brasileiras ou estrangeiras: - Novartis: Entresto; AstraZeneca: Forxiga; Boehringer: Jardiance; Bayer: Vericiguat; Vifor: Ferrinject. B - Financiamento de pesquisas sob sua responsabilidade direta/pessoal (direcionado ao departamento ou instituição) provenientes da indústria farmacêutica, de órteses, próteses, equipamentos e implantes, brasileiras ou estrangeiras. - Bayer: Vericiguat.
Lucelia Batista Neves Cunha Magalhães	Nada a ser declarado
Luciana Diniz Nagem Janot de Matos	Nada a ser declarado
Magaly Arrais	Declaração financeira A - Pagamento de qualquer espécie e desde que economicamente apreciáveis, feitos a (i) você, (ii) ao seu cônjuge/ companheiro ou a qualquer outro membro que resida com você, (iii) a qualquer pessoa jurídica em que qualquer destes seja controlador, sócio, acionista ou participante, de forma direta ou indireta, recebimento por palestras, aulas, atuação como proctor de treinamentos, remunerações, honorários pagos por participações em conselhos consultivos, de investigadores, ou outros comitês, etc. Provenientes da indústria farmacêutica, de órteses, próteses, equipamentos e implantes, brasileiras ou estrangeiras: - Edwards / Boston: Implante transcateter valvar; Medtronic: Implante.
Marcelo Heitor Vieira Assad	Declaração financeira A - Pagamento de qualquer espécie e desde que economicamente apreciáveis, feitos a (i) você, (ii) ao seu cônjuge/ companheiro ou a qualquer outro membro que resida com você, (iii) a qualquer pessoa jurídica em que qualquer destes seja controlador, sócio, acionista ou participante, de forma direta ou indireta, recebimento por palestras, aulas, atuação como proctor de treinamentos, remunerações, honorários pagos por participações em conselhos consultivos, de investigadores, ou outros comitês, etc. Provenientes da indústria farmacêutica, de órteses, próteses, equipamentos e implantes, brasileiras ou estrangeiras: - Novo Nordisk: Semaglutida; AstraZeneca: Dapagliflozina; BI: Empagliflozina; GSK: Shingrix; Biolab: Evolucumabe; Daiichi Sankyo: Benicar Triplo; Novartis: Dislipidemia. B - Financiamento de pesquisas sob sua responsabilidade direta/pessoal (direcionado ao departamento ou instituição) provenientes da indústria farmacêutica, de órteses, próteses, equipamentos e implantes, brasileiras ou estrangeiras. - Amgen: LP(A). Outros relacionamentos Financiamento de atividades de educação médica continuada, incluindo viagens, hospedagens e inscrições para congressos e cursos, provenientes da indústria farmacêutica, de órteses, próteses, equipamentos e implantes, brasileiras ou estrangeiras: - Novo Nordisk: Semaglutina; BI: Empagliglozina.
Marcia M. Barbosa	Nada a ser declarado
Marconi Gomes da Silva	Nada a ser declarado
Maria Alayde Mendonça Rivera	Nada a ser declarado
Maria Cristina Costa de Almeida	Nada a ser declarado
Maria Cristina de Oliveira Izar	Declaração financeira A - Pagamento de qualquer espécie e desde que economicamente apreciáveis, feitos a (i) você, (ii) ao seu cônjuge/ companheiro ou a qualquer outro membro que resida com você, (iii) a qualquer pessoa jurídica em que qualquer destes seja controlador, sócio, acionista ou participante, de forma direta ou indireta, recebimento por palestras, aulas, atuação como proctor de treinamentos, remunerações, honorários pagos por participações em conselhos consultivos, de investigadores, ou outros comitês, etc. Provenientes da indústria farmacêutica, de órteses, próteses, equipamentos e implantes, brasileiras ou estrangeiras: - Amgen: Repatha; Amryt Pharma: Lojuxta; AstraZeneca: Dapagliflozina; Aché: Trezor, Trezete; Biolab: Livalo; Abbott: Lipidil; EMS: Rosuvastatina; Eurofarma: Rosuvastatina; Sanofi: Praluent, Zympass, Zympass Eze, Efluelda; Libbs: Plenance, Plenance Eze; Novo Nordisk: Ozempic, Victoza; Servier: Acertamlo, Alertalix; PTCBio: Waylivra. B - Financiamento de pesquisas sob sua responsabilidade direta/pessoal (direcionado ao departamento ou instituição) provenientes da indústria farmacêutica, de órteses, próteses, equipamentos e implantes, brasileiras ou estrangeiras. - PTCBio: Waylivra; Amgen: Repatha; Novartis: Inclisiran, Pelacarsen; NovoNordisk: Ziltivekimab. Outros relacionamentos Financiamento de atividades de educação médica continuada, incluindo viagens, hospedagens e inscrições para congressos e cursos, provenientes da indústria farmacêutica, de órteses, próteses, equipamentos e implantes, brasileiras ou estrangeiras: - Novo Nordisk: Diabetes.
Maria Elizabeth Navegantes Caetano Costa	Declaração financeira A - Pagamento de qualquer espécie e desde que economicamente apreciáveis, feitos a (i) você, (ii) ao seu cônjuge/ companheiro ou a qualquer outro membro que resida com você, (iii) a qualquer pessoa jurídica em que qualquer destes seja controlador, sócio, acionista ou participante, de forma direta ou indireta, recebimento por palestras, aulas, atuação como proctor de treinamentos, remunerações, honorários pagos por participações em conselhos consultivos, de investigadores, ou outros comitês, etc. Provenientes da indústria farmacêutica, de órteses, próteses, equipamentos e implantes, brasileiras ou estrangeiras: - Libbs: Plenance Enze; Servier: Vastarel. Outros relacionamentos Financiamento de atividades de educação médica continuada, incluindo viagens, hospedagens e inscrições para congressos e cursos, provenientes da indústria farmacêutica, de órteses, próteses, equipamentos e implantes, brasileiras ou estrangeiras: - Libbs; Servier: Participação em congresso.
Maria Sanali Moura de Oliveira Paiva	Nada a ser declarado
Marildes Luiza de Castro	Declaração financeira A - Pagamento de qualquer espécie e desde que economicamente apreciáveis, feitos a (i) você, (ii) ao seu cônjuge/ companheiro ou a qualquer outro membro que resida com você, (iii) a qualquer pessoa jurídica em que qualquer destes seja controlador, sócio, acionista ou participante, de forma direta ou indireta, recebimento por palestras, aulas, atuação como proctor de treinamentos, remunerações, honorários pagos por participações em conselhos consultivos, de investigadores, ou outros comitês, etc. Provenientes da indústria farmacêutica, de órteses, próteses, equipamentos e implantes, brasileiras ou estrangeiras: - AstraZeneca: Forxiga/Insuficiência cardíaca; Servier: Acertil/Hipertensão arterial.
Marly Uellendahl	Declaração financeira A - Pagamento de qualquer espécie e desde que economicamente apreciáveis, feitos a (i) você, (ii) ao seu cônjuge/ companheiro ou a qualquer outro membro que resida com você, (iii) a qualquer pessoa jurídica em que qualquer destes seja controlador, sócio, acionista ou participante, de forma direta ou indireta, recebimento por palestras, aulas, atuação como proctor de treinamentos, remunerações, honorários pagos por participações em conselhos consultivos, de investigadores, ou outros comitês, etc. Provenientes da indústria farmacêutica, de órteses, próteses, equipamentos e implantes, brasileiras ou estrangeiras: - GE/Healthcare: Palestras e treinamentos na área de Ressonância Magnética Cardiovascular.
Milena dos Santos Barros Campos	Nada a ser declarado
Mucio Tavares de Oliveira Junior	Declaração financeira A - Pagamento de qualquer espécie e desde que economicamente apreciáveis, feitos a (i) você, (ii) ao seu cônjuge/ companheiro ou a qualquer outro membro que resida com você, (iii) a qualquer pessoa jurídica em que qualquer destes seja controlador, sócio, acionista ou participante, de forma direta ou indireta, recebimento por palestras, aulas, atuação como proctor de treinamentos, remunerações, honorários pagos por participações em conselhos consultivos, de investigadores, ou outros comitês, etc. Provenientes da indústria farmacêutica, de órteses, próteses, equipamentos e implantes, brasileiras ou estrangeiras: - Sanofi/Pasteur: Vacinas; AstraZeneca / Boehringer Ingelheim / Merck: palestras; Novo Nordisk: Conselho consultivo.
Olga Ferreira de Souza	Outros relacionamentos Financiamento de atividades de educação médica continuada, incluindo viagens, hospedagens e inscrições para congressos e cursos, provenientes da indústria farmacêutica, de órteses, próteses, equipamentos e implantes, brasileiras ou estrangeiras: - Daiichi Sankyo.
Ricardo Alves da Costa	Nada a ser declarado
Ricardo Quental Coutinho	Nada a ser declarado
Sheyla Cristina Tonheiro Ferro da Silva	Declaração financeira A - Pagamento de qualquer espécie e desde que economicamente apreciáveis, feitos a (i) você, (ii) ao seu cônjuge/ companheiro ou a qualquer outro membro que resida com você, (iii) a qualquer pessoa jurídica em que qualquer destes seja controlador, sócio, acionista ou participante, de forma direta ou indireta, recebimento por palestras, aulas, atuação como proctor de treinamentos, remunerações, honorários pagos por participações em conselhos consultivos, de investigadores, ou outros comitês, etc. Provenientes da indústria farmacêutica, de órteses, próteses, equipamentos e implantes, brasileiras ou estrangeiras: - Palestras para Novartis: Entresto; AstraZeneca: Forxiga/ Xigduo; aliaça Boeringher-Lilly: Jardiance; Servier: Acertil, Acertalix, triplixan; Novonordisk: Saxenda, Ozempic, Rybelsus; Libbis: Naprix; Vifor: Ferinject. Outros relacionamentos Financiamento de atividades de educação médica continuada, incluindo viagens, hospedagens e inscrições para congressos e cursos, provenientes da indústria farmacêutica, de órteses, próteses, equipamentos e implantes, brasileiras ou estrangeiras: - Aliança Boeringher-Lilly: Jardiance; Novonordisk: Ozempic, Rybelsus, Saxenda; Servier: Acertil, Acertalix, Triplixan.
Sílvia Marinho Martins	Nada a ser declarado
Simone Cristina Soares Brandão	Nada a ser declarado
Susimeire Buglia	Nada a ser declarado
Tatiana Maia Jorge de Ulhôa Barbosa	Nada a ser declarado
Thais Aguiar do Nascimento	Declaração financeira A - Pagamento de qualquer espécie e desde que economicamente apreciáveis, feitos a (i) você, (ii) ao seu cônjuge/ companheiro ou a qualquer outro membro que resida com você, (iii) a qualquer pessoa jurídica em que qualquer destes seja controlador, sócio, acionista ou participante, de forma direta ou indireta, recebimento por palestras, aulas, atuação como proctor de treinamentos, remunerações, honorários pagos por participações em conselhos consultivos, de investigadores, ou outros comitês, etc. Provenientes da indústria farmacêutica, de órteses, próteses, equipamentos e implantes, brasileiras ou estrangeiras: - Abbott: Consultoria.
Thais Vieira	Outros relacionamentos Financiamento de atividades de educação médica continuada, incluindo viagens, hospedagens e inscrições para congressos e cursos, provenientes da indústria farmacêutica, de órteses, próteses, equipamentos e implantes, brasileiras ou estrangeiras: - Boehringer / AstraZeneca / Torrent / Novo Nordisk: Palestrante.
Valquíria Pelisser Campagnucci	Nada a ser declarado
Viviana Guzzo Lemke	Nada a ser declarado
Walkiria Samuel Avila	Nada a ser declarado

Sumário

1. Introdução/Highlights 11

1.1. Introdução 12

1.2 Destaques deste Posicionamento 12

**1.2.1. Epidemiologia** 12

**1.2.2. Bases Fisiopatológicas da Doença Aterotrombótica** 12

**1.2.3. Apresentação Clínica, Diagnóstico e Tratamento Clínico** 12

**1.2.4. Diagnóstico por Avaliação Funcional Gráfica** 13

**1.2.5. Diagnóstico por Imagem Cardiovascular Não Invasiva** 13

**1.2.6. Arritmias na Cardiopatia Isquêmica** 13

**1.2.7. Aterotrombose na Gravidez, Contracepção, Infertilidade, Síndrome Antifosfolípide** 14

**1.2.8. Cardiomiopatia Isquêmica** 14

**1.2.9. Intervenção Coronariana Percutânea** 14

**1.2.10. Revascularização Miocárdica e Transplante Cardíaco** 14

**1.2.11. Reabilitação na Cardiomiopatia Isquêmica** 15

2. Epidemiologia da Doença Isquêmica do Coração nas Mulheres 15

2.1. Introdução 15

2.2. Mortalidade 15

2.3. Prevalência e Incidência 18

2.4. Carga de Doenças 21

2.5. Fatores de Risco 21

2.6. Conclusão 22

3. Bases Fisiopatológicas da Doença Aterotrombótica 23

3.1. Introdução 23

3.2. Ruptura de Placa 23

3.3. Dissecção Espontânea de Coronária 23

3.4. Espasmo Coronariano 24

3.5. Disfunção Microvascular Coronariana 24

3.6. Embolia e Trombose 24

3.7. Síndrome de Takotsubo 24

3.8. Miocardite 24

4. Apresentação Clínica, Diagnóstico e Tratamento Clínico 25

4.1. Dor Torácica de Etiologia Isquêmica 25

4.2. Fatores de Risco Estabelecidos e Modificáveis 26

4.3. Fatores de Risco Estabelecidos e Não Modificáveis 27

4.4. Fatores de Risco Específicos da Mulher 27

4.5. Fatores de Risco Sub-reconhecidos 28

**4.5.1. Recomendações** 28

4.6. Tratamento Medicamentoso nas Diferentes Formas de Manifestação Isquêmica 28

**4.6.1. Recomendações** 29

5. Diagnóstico por Avaliação Funcional Gráfica 29

5.1. Eletrocardiograma de Repouso 29

5.2. Teste Ergométrico 29

**5.2.1. Recomendações** 30

5.3. Teste Cardiopulmonar de Exercício 30

6. Diagnóstico por Imagem Cardiovascular Não Invasiva 32

6.1. Introdução 32

6.2. Ecocardiograma de Repouso e sob Estresse 32

6.3. Ultrassonografia Vascular 33

6.4. Tomografia Computadorizada 34

6.5. Ressonância Magnética Cardíaca 34

6.6. Medicina Nuclear 35

7. Arritmias na Cardiomiopatia Isquêmica 36

7.1. Fibrilação Atrial e Doença Isquêmica do Coração 36

7.2. Arritmias Ventriculares: Morte Súbita, Prevenção e Tratamento 37

7.3. Terapia de Ressincronização Cardíaca 39

7.4. Recomendações 40

8. Aterotrombose na Gravidez, Contracepção, Infertilidade, Síndrome Antifosfolípide 41

8.1. Introdução 41

8.2. Período da Gravidez 41

8.3. Contracepção 41

**8.3.1. Recomendações** 44

8.4. Infertilidade 44

8.5. Síndrome Antifosfolípide 45

**8.5.1. Recomendações** 47

9. Cardiomiopatia Isquêmica na Mulher 47

9.1. Introdução 47

9.2. Tratamento Clínico 48

9.3. Dispositivos e Insuficiência Cardíaca Avançada 48

9.4. Cardiodesfibrilador Implantável 51

9.5. Insuficiência Cardíaca Avançada 51

9.6. Recomendações 51

**9.6.1. Manejo Clínico e Indicações de Terapias Avançadas** 51

10. Intervenção Coronariana Percutânea 51

10.1. Introdução 51

10.2. Acesso Vascular para o Cateterismo Cardíaco e Intervenção Coronariana Percutânea em Mulheres 51

10.3. Diagnóstico 52

**10.3.1. Angiografia Coronária** 52

**10.3.2. Imagem Intravascular** 52

**10.3.3. Testes Invasivos com Guia de Medição** 53

**10.3.3.1. Reserva de Fluxo Fracionada** 53

**10.3.3.2. Razão de Pressão Instantânea Livre de Onda** 53

**10.3.4. Testes Funcionais** 53

10.4. Tratamento Percutâneo da Doença Aterotrombótica Coronária em Mulheres 54

**10.4.1. Revascularização para Síndromes Coronarianas Crônicas** 54

**10.4.1.1. Doença do Tronco de Coronária Esquerda** 55

**10.4.1.2. Oclusão Total Crônica** 55

**10.4.2. Revascularização para Infarto do Miocárdio sem Supradesnivelamento do Segmento ST** 55

**10.4.3. Revascularização para Infarto do Miocárdio Com Supradesnivelamento do Segmento ST** 55

**10.4.3.1. Estratégias na Abordagem da Doença Coronariana Múltipla**

**10.4.3.2. Choque Cardiogênico** 55

**10.4.4. Considerações sobre o Dispositivo durante a Revascularização Percutânea** 55

**10.4.4.1. Stents Farmacológicos** 55

**10.4.4.2. Balão Farmacológico** 55

**10.4.4.3. Aterectomia Rotacional e Litotripsia Intravascular** 56

10.5. Terapia Farmacológica Adjunta 56

10.6. Gaps no Conhecimento 57

10.7. Recomendações 58

11. Intervenção Cirúrgica, Transplante Cardíaco 58

11.1. Revascularização do Miocárdio 58

**11.1.1. Cirurgia de Revascularização do Miocárdio em Mulheres – Recomendações** 60

11.2. Transplante Cardíaco 60

**11.2.1. Transplante Cardíaco em Mulheres – Recomendações** 62

12. Reabilitação na Cardiomiopatia Isquêmica das Mulheres 62

Referências 63

## 1. Introdução/Highlights

### 1.1. Introdução

As diferenças entre os sexos vão além das questões cromossômicas entre homens (XY) e mulheres (XX). Os valores sociais, as percepções e os comportamentos distintos moldam padrões e criam diferentes papéis na sociedade, o que pode gerar diferenças no estilo de vida e comportamento, possivelmente influenciando epidemiologia, manifestação clínica e tratamento.^1^

É importante destacar que, do ponto de vista clínico, a doença isquêmica do coração (DIC) ocorre mais precocemente no homem. Contudo, a incidência e a prevalência na mulher aumentam acentuadamente após a menopausa. Enfatiza-se ainda que a maior proporção de mulheres com sintomas anginosos e síndrome coronariana aguda (SCA) tem DIC não obstrutiva.

A DIC em mulheres inclui a aterosclerose coronariana clássica e compreende fisiopatologia variada, como disfunção microvascular coronariana, disfunção endotelial, anormalidades vasomotoras e dissecção espontânea de artéria coronária (DEAC).^2^

Em relação à anatomia, mulheres têm artérias coronárias epicárdicas menores do que homens, mesmo após ajuste para superfície corporal e massa do ventrículo esquerdo (VE). Porém, em comparação com os homens, as mulheres têm menor prevalência de aterosclerose coronariana obstrutiva e características de placa diversas, ainda que em níveis comparáveis de isquemia.^3^

As mulheres que apresentam DIC obstrutiva geralmente são mais velhas do que os homens, têm mais comorbidades cardiovasculares e maior incidência de desfechos cardiovasculares adversos, incluindo mortalidade após infarto agudo do miocárdio (IAM).^4^

As mulheres são menos propensas do que os homens a apresentar ruptura de placa e, nelas, a revascularização da artéria ocluída pode ser mais difícil devido a sangramento no local de acesso e artérias coronárias pequenas e mais tortuosas.^5^

A dor torácica é o sintoma mais prevalente de IAM em ambos os sexos. No entanto, as mulheres são mais propensas a apresentar sintomas atípicos, incluindo dor na parte superior das costas e pescoço, fadiga, náuseas e vômitos.^6^ A maioria das mulheres com IAM apresenta sintomas prodrômicos de falta de ar, fadiga incomum ou desconforto em braço/mandíbula nas semanas anteriores. Angina estável é a apresentação clínica mais frequente em mulheres com DIC em oposição a IAM ou morte súbita.^7^

Revisão recentemente publicada resume alguns aspectos relacionados a vantagens e desvantagens, bem como valores de sensibilidade e especificidade de acordo com o gênero, dos principais métodos diagnósticos.^8^ A menor sensibilidade do teste ergométrico (TE) para detecção de doença coronariana obstrutiva em mulheres limita sua utilização no cenário da cardiomiopatia isquêmica (CMI).^9^ A ecocardiografia sob estresse (exercício ou dobutamina - ESE) tem performance melhor do que o TE, embora inferior à de outros métodos, com estudos mostrando desempenho similar ou inferior em mulheres.^10,11^ A incorporação de avaliação por doppler tecidual tem permitido análise quantitativa de viabilidade. A cintilografia miocárdica com imagens obtidas por tomografia computadorizada por emissão de fóton único (SPECT) tem boa performance em mulheres, principalmente quanto à sensibilidade.^12^ Algumas limitações em mulheres são relacionadas a falsos positivos decorrentes de atenuação mamária e menor acurácia em corações pequenos.^13^ Para avaliação de estresse miocárdio, a tomografia por emissão de pósitron (PET) é superior ao SPECT em qualidade de imagem e acurácia, tanto em mulheres quanto em homens.^14^ Dados adicionais caracterizam inflamação e vulnerabilidade das placas, eventos adversos e potencial benefício de revascularização.^15^

O TE e a ESE são considerados seguros na gestação, pois evitam exposição a radiação, enquanto dobutamina e dipiridamol são considerados categoria B. Técnicas de SPECT e PET-CT devem ser evitadas, mas a ressonância magnética cardíaca (RMC) é uma boa opção na gestação.^16^

Nas últimas décadas, têm sido documentadas diferenças na fisiologia e fisiopatologia cardiovascular entre mulheres e homens. Essas diferenças incluem as propriedades eletrofisiológicas da célula cardíaca, o que pode influenciar na ocorrência de arritmias clínicas distintas entre os sexos. Possivelmente, tais diferenças são de origem multifatorial. Entretanto, a ação hormonal e a influência autonômica são fatores importantes no comportamento eletrofisiológico distinto entre mulheres e homens.^17^

A média da frequência cardíaca (FC) em mulheres é aproximadamente 3 a 5 batimentos/minuto mais alta que a observada em homens.^17^ Além disso, foi documentado um menor tempo de recuperação do nó sinusal, menor intervalo HV, maior velocidade de condução ventricular e aumento no intervalo QT em mulheres.^18^

A prevalência de taquicardia sinusal inapropriada é muito maior em mulheres. Um estudo com 321 pacientes acompanhados por taquicardia sinusal inapropriada mostrou que 92% deles eram do sexo feminino.^19^ Aproximadamente 60% das taquicardias de QRS estreito observadas na prática clínica são secundárias a taquicardia por reentrada nodal, sendo sua prevalência duas vezes maior nas mulheres.^20^ O período refratário da via lenta é menor no sexo feminino, o que pode aumentar a janela de indutibilidade arrítmica e justificar o maior número de casos em mulheres.^21^ Entretanto, vale ressaltar que essa característica não interfere no sucesso do tratamento por ablação, que corresponde a 95% dos casos em ambos os sexos. Em contraste, a taquicardia por reentrada atrioventricular predomina no sexo masculino.^18^ Os homens apresentam mais frequentemente uma via acessória manifesta, com localização lateral esquerda. Por outro lado, nas mulheres, observa-se quase três vezes mais vias à direita.^21^

A incidência de fibrilação atrial (FA) ajustada para idade é uma vez e meia a duas vezes maior em homens. Entretanto, o risco de FA ao longo da vida é semelhante em ambos os sexos devido à maior expectativa de vida no sexo feminino. Nas mulheres, existe um aumento desproporcional de FA com o avançar da idade, de tal forma que, aos 85 anos, as diferenças na prevalência são discretas.^17,22^ Além disso, as mulheres são mais sintomáticas e apresentam pior qualidade de vida quando comparadas com os homens. Os mecanismos associados às diferenças entre os sexos na FA são inúmeros, mas é importante ressaltar que a DIC, mais observada no sexo masculino, pode corroborar com a maior incidência de FA nesse grupo. Em relação ao tratamento com drogas antiarrítmicas (DAA), as mulheres apresentam mais efeitos adversos. O aumento no intervalo QT basal pode afetar a tolerância ao uso de DAA, especialmente da classe III, exigindo uma monitorização mais cuidadosa nesse grupo de pacientes. Em relação aos resultados da ablação por cateter, estudos observacionais demonstram que as mulheres são submetidas menos frequente e mais tardiamente a ablação, em geral com evolução pior após o procedimento.^23^

As arritmias ventriculares em pacientes com coração normal apresentam características epidemiológicas variáveis entre os sexos. A taquicardia ventricular (TV) de via de saída do ventrículo direito é mais frequente em mulheres, ao passo que as arritmias com origem fascicular ocorrem mais em homens.^24^ Pacientes pré-púberes do sexo masculino com síndrome do QT longo tipo I e pré-púberes do sexo feminino com síndrome do QT longo tipo II apresentam maior risco de arritmia ventricular.^25^ A ocorrência de morte súbita cardíaca em mulheres é quase a metade da ocorrência em homens, mesmo após ajuste para fatores predisponentes.^26^

As mulheres com insuficiência cardíaca (IC) geralmente são mais idosas que os homens e têm maior prevalência de IC com fração de ejeção preservada (ICFEp). Além disso, mostram mais cardiopatia não isquêmica, diabetes *mellitus* (DM) e hipertensão arterial sistêmica (HAS). Vários fatores levam a menor inclusão de mulheres nos estudos sobre DIC. Os relacionados à condição da paciente são (1) necessidade de viajar e se ausentar do trabalho, (2) ausentar-se das responsabilidades com os filhos, a família e o lar, (3) necessidade de elevado nível de compromisso, (4) barreiras socioeconômicas, psicológicas, culturais e de saúde. Os fatores relacionados ao estudo são: (1) baixas taxas de encaminhamento e triagem de elegibilidade, (2) falta de critério de elegibilidade relacionado ao sexo, (3) liderança heterogênea dos estudos, principalmente composta por homens, (4) exclusão de idosos. Ações futuras são importantes para uma maior inclusão de mulheres portadoras de DIC nos grandes estudos.^27^Este posicionamento, através de ação conjunta das especialidades da cardiologia com expertise na mulher, tem como principal objetivo divulgar informações sobre a DIC sob vários aspectos para um melhor entendimento de suas particularidades, visando melhor tratar essas pacientes e consequentemente reduzir sua morbimortalidade.

### 1.2. Destaques deste Posicionamento

#### 1.2.1. Epidemiologia

• A DIC mantém-se como a principal causa de morte de mulheres e homens no Brasil. Houve diminuição mais pronunciada do percentual da taxa de mortalidade por DIC padronizada nas mulheres entre os anos de 1990 e 2019, -55,5 (II_95%_, -58,7; -52,3), do que nos homens, -49,5 (II_95%_, -52,5; -46,6), nesse mesmo período. Esse declínio foi desigual nas unidades da federação em ambos os sexos, estando relacionado com o envelhecimento da população e com o índice sociodemográfico (SDI) de 2019.
• A incidência e a prevalência da DIC vêm diminuindo no Brasil ao longo dos últimos 20 anos em mulheres e homens, embora tenha ocorrido aumento na mortalidade precoce por DIC entre 18 anos e 55 anos, especialmente nas mulheres. Nas mulheres, houve uma diferença entre as regiões brasileiras na incidência de DIC padronizada por idade, que foi maior nas regiões Sudeste e Sul e menor na região Norte.
• As mulheres apresentaram taxas significativamente menores de angioplastia primária e significativamente maiores de mortalidade hospitalar. A prevalência de MINOCA (infarto do miocárdio na ausência de obstrução arterial coronária) é maior nas mulheres, com mortalidade semelhante à da DIC obstrutiva, associando-se com risco de eventos maiores.
• O estudo do *Global Burden of Diseases* (GBD) 2019 estimou taxa padronizada de DALYs (anos de vida ajustados por incapacidade) por DIC por 100 mil habitantes de 1.088,4 (992,8; 1.158,9) nas mulheres e de 2.116,5 (II_95%_, 1.989,9; 2.232,2) nos homens. A DIC foi a segunda causa mais comum de DALYs no Brasil nas mulheres (após distúrbios neonatais) e nos homens (após violência interpessoal) em 2019. Essas taxas foram heterogêneas nas regiões geográficas brasileiras e a tendência das taxas de DALYs padronizadas por idade de 1990 a 2019, nas mulheres, assemelhou-se à das taxas de mortalidade.
• As mulheres apresentam maior frequência de fatores de risco cardiovascular (FRCV) não tradicionais, como estresse mental e depressão, e sofrem maior consequência das desvantagens sociais devido a raça, etnicidade e renda. As mulheres têm ainda os fatores de risco (FR) inerentes ao sexo, como gravidez, menopausa e menarca, entre outros.

#### 1.2.2. Bases Fisiopatológicas da Doença Aterotrombótica

A doença coronariana obstrutiva, caracterizada pela presença de placas de aterosclerose nas paredes das artérias coronárias, é o substrato mais frequente de DIC nas mulheres. Entretanto, é reconhecido que a doença coronariana não obstrutiva com evidências de danos ao músculo cardíaco ou outros sinais de enfermidade coronariana afeta desproporcionalmente mais mulheres.

• Os mecanismos fisiopatológicos envolvidos na MINOCA incluem ruptura de placa coronariana, DEAC, vasoespasmo coronariano, disfunção microvascular coronariana e embolismo/trombose. Importante destacar as síndromes que mimetizam clinicamente MINOCA, como Takotsubo, miocardite e cardiomiopatia não isquêmica.

#### 1.2.3. Apresentação Clínica, Diagnóstico e Tratamento Clínico

• As diferenças biológicas e socioculturais específicas do sexo feminino na apresentação da dor torácica da DIC podem explicar as diferenças em sua apresentação clínica, seu diagnóstico e seu manejo, levando a atrasos na conduta e, consequentemente, desfechos desfavoráveis.
• Os sintomas isquêmicos das mulheres são mais relacionados ao estresse emocional ou mental e menos frequentemente precipitados pela atividade física, em comparação aos homens. Escore de risco global e caracterização de angina não devem ser usados uniformemente em mulheres e homens, devido ao impacto diferente dos FR e às manifestações clínicas variáveis entre os sexos.
• Existem diferentes proporções na relevância dos FR entre os sexos, como HAS, obesidade, DM e tabagismo. Os FR específicos do sexo feminino são relevantes na estratificação de risco: pré-eclâmpsia e diabetes gestacional aumentam o risco cardiovascular (RCV) da mulher por toda a vida.
• Mulheres são menos submetidas a coronariografia e tratamento cirúrgico, incluindo suporte circulatório mecânico no choque cardiogênico. No entanto, têm maior mortalidade e complicações pós-operatórias, apesar de menor carga aterosclerótica.
• Menos de 50% das pacientes são submetidas a tratamento medicamentoso adequado, além de ser baixa a aderência ao tratamento e existir subutilização de reabilitação cardíaca.
• O tratamento da MINOCA e da isquemia do miocárdio sem doença coronariana obstrutiva (INOCA) baseia-se na mudança de estilo de vida, controle dos FR e tratamento antianginoso.

#### 1.2.4. Diagnóstico por Avaliação Funcional Gráfica

• A posição inadequada dos eletrodos no eletrocardiograma (ECG) pode causar diagnóstico equivocado nas mulheres. Mamas volumosas ou próteses mamárias podem gerar complexos de baixa voltagem e reduzem a amplitude da onda R nas derivações V1 e V2, simulando área inativa. O ECG na avaliação de dor torácica utiliza os mesmos critérios diagnósticos descritos para o sexo masculino, exceto para análise de lesão subepicárdica.
• O TE é recomendado como método inicial de escolha na avaliação de mulheres sintomáticas de risco intermediário para DIC, com ECG de repouso normal e capazes de se exercitar. Além das alterações do segmento ST, a capacidade de exercício, as respostas cronotrópica e da pressão arterial (PA), a recuperação da FC e a avaliação do escore de Duke (ED) são informações prognósticas que aumentam a acurácia do TE, especialmente nas mulheres. A capacidade funcional é a variável prognóstica mais importante para morbimortalidade por todas as causas em mulheres, incluindo as assintomáticas. A incapacidade de atingir 5 MET é preditora independente de alto risco, com aumento de três vezes na mortalidade em comparação àquelas que atingem mais de 8 MET.
• O teste cardiopulmonar de exercício (TCPE) permite realizar diagnóstico, prognóstico, acompanhamento após intervenção terapêutica e prescrição de exercícios aeróbicos na DIC. O TCPE apresenta maior acurácia diagnóstica na DIC de mulheres do que o TE. Além dos critérios clínicos, hemodinâmicos e eletrocardiográficos, o TCPE fornece a análise do pulso de oxigênio (PO_2_), que permite inferir a disfunção ventricular isquêmica induzida pelo esforço, cujo achado pode ser relevante no diagnóstico da DIC macro e microvascular na mulher.

#### 1.2.5. Diagnóstico por Imagem Cardiovascular Não Invasiva

• A ESE apresenta boa acurácia na investigação de DIC nas mulheres, avalia a função sistólica do VE, permite o diagnóstico diferencial, além de oferecer segurança pela ausência de radiação.
• A ultrassonografia vascular (USV) é útil na detecção de placas carotídeas como modificador de risco em mulheres de probabilidade intermediária e/ou FR não tradicionais, no rastreamento de aneurisma de aorta abdominal em mulheres tabagistas ou ex-tabagistas entre 55 anos e 75 anos e na busca de doença arterial periférica (DAP) silenciosa.
• Angiotomografia coronariana (Angio-TC) apresenta boa acurácia diagnóstica e prognóstica na avaliação de DIC em mulheres, caracterizando e quantificando as lesões. Classicamente, mulheres apresentam lesões menos calcificadas e não obstrutivas em relação a homens.
• A RMC oferece maiores informações na detecção da DIC na mulher: identifica isquemia por DIC obstrutiva e não obstrutiva, MINOCA, avalia viabilidade miocárdica, distingue a doença isquêmica da inflamatória e define diagnóstico de Takotsubo.
• A imagem nuclear avalia todo o espectro da DIC, desde doença coronariana obstrutiva até disfunção microvascular coronariana, sem limitações quanto a função renal, arritmias, obesidade e dispositivos intracardíacos.

#### 1.2.6. Arritmias na Cardiopatia Isquêmica

• As mulheres têm mais taquicardia sinusal inapropriada e taquicardia por reentrada nodal e menos FA, arritmias ventriculares malignas, como TV e fibrilação ventricular (FV), e morte súbita cardíaca do que os homens. A despeito da diferença na prevalência entre os sexos, as mulheres se beneficiam do tratamento das arritmias cardíacas.
• As mulheres com FA têm maior prevalência de HAS, obesidade, depressão, ICFEp e doença valvar como causa da arritmia. São também preditores de risco de FA nas mulheres a falta ou escassez de exercício, a monoterapia com estrogênio e a multiparidade. As mulheres com FA têm alto risco de acidente vascular cerebral (AVC), não havendo diferença significativa no risco de AVC ou de embolia sistêmica e sangramento gastrointestinal entre mulheres e homens em uso de anticoagulantes. Foi demonstrada redução significativa de hemorragia intracraniana e mortalidade por todas as causas nas mulheres com FA em uso de anticoagulantes de ação direta (DOACs). As mulheres com FA têm pior qualidade de vida do que os homens e são menos submetidas a procedimentos, como a ablação por cateter ou cardioversão elétrica (CVE).
• As mulheres têm menos CMI que os homens e aquelas com DIC e portadoras de cardiodesfibrilador implantável (CDI) apresentam menos episódios de TV/FV e de tempestade elétrica e ainda menos choques pelo CDI do que os homens. As mulheres têm menos CMI e menor carga de fibrose do que os homens submetidos à terapia de ressincronização cardíaca (TRC) e respondem melhor à TRC do que os homens, com maiores intervalos de tempo até a primeira hospitalização e menor mortalidade. As terapias como CDI e TRC trazem benefícios na mortalidade. As mulheres representam em torno de 30% das populações dos estudos com essas terapias.
• O percentual de mulheres contempladas nos estudos de ablação de TV na população com DIC é baixo (7-13%). A menor indicação de procedimentos invasivos, a menor indução de TV sustentada e o menor número de choques apropriados são fatores que podem contribuir para esse percentual reduzido.

#### 1.2.7. Aterotrombose na Gravidez, Contracepção, Infertilidade, Síndrome Antifosfolípide

• A doença aterotrombótica é uma das causas mais frequentes de IAM durante a gravidez e o puerpério.
• A conduta diante da DIC aguda durante a gravidez deve priorizar a vida materna e seguir as recomendações para a população em geral.
• A tríade conjugada (tabagismo, idade acima de 35 anos e uso prolongado, > 10 anos, de anticoncepcional combinado oral) é considerada o fator determinante da manifestação clínica da doença aterotrombótica durante a gravidez e o puerpério.
• A queixa de dor torácica durante a gravidez em mulheres que apresentam FR para a doença cardiovascular (DCV) não deve ser subestimada e deve seguir protocolo convencional de investigação para SCA.
• Os contraceptivos não estão isentos de efeitos aterotrombóticos, mas a não prescrição incorre no risco de gravidez não planejada, principalmente na adolescência e em mulheres com comorbidades. A seleção do método de contracepção deve ser individualizada, atender à preferência e idade da paciente, assim como à segurança e eficácia do método.
• A frequência de distúrbios metabólicos pró-ateroscleróticos, particularmente obesidade e aumento de colesterol total, LDL-colesterol e triglicerídeos, é maior entre as mulheres que sofrem de infertilidade.
• A terapêutica da infertilidade é considerada um potencial FR para os distúrbios hipertensivos na gestação subsequente. Contudo, ainda não foi demonstrada correlação entre tratamento para fertilização e eventos cardiovasculares.
• Diagnóstico de síndrome antifosfolípide (SAF) deve ser presumido diante da manifestação clínica de trombose vascular e/ou complicações obstétricas recorrentes, sendo sua investigação obrigatória na presença de AVC e IAM em mulheres jovens.

#### 1.2.8. Cardiomiopatia Isquêmica

• A IC relacionada à CMI é uma importante causa de morbidade e mortalidade em mulheres, que tendem a desenvolvê-la em idade mais avançada que os homens.
• A ICFEp é mais comum em mulheres, mas a etiologia isquêmica tende a manifestar-se na forma dilatada e de fração de ejeção reduzida, ainda que com menos fibrose endomiocárdica quando comparada com homens.
• As mulheres estão pouco representadas em ensaios clínicos para IC. Apesar disso, as recomendações para terapias medicamentosas e avançadas nas diretrizes não sugerem tratamento individualizado para as mulheres.

#### 1.2.9. Intervenção Coronariana Percutânea

• No procedimento intervencionista, estratégias para redução no risco de sangramento e de complicações vasculares devem ser adotadas, priorizando a via de acesso radial e a utilização de fármacos adjuntos em doses adequadas ao peso e à função renal.
• Avaliação funcional na doença coronariana, através da análise da reserva de fluxo fracionada (FFR), é de grande valia naquelas obstruções intermediárias (40% a 70%), quando não se tem isquemia comprovada por métodos não invasivos. As mulheres parecem ter valores de FFR mais altos para doença coronariana não obstrutiva, ratificando ser ainda mais relevante medir a FFR em mulheres, além de ser também um preditor univariado significativo de prognóstico para esse grupo.
• Devem-se identificar perfis de alto risco para sangramento e perseguir a excelência nos resultados da intervenção através de seleção criteriosa e preparo adequado das lesões, baixo limiar para utilização de guia de imagem intravascular, implante otimizado dos *stents* e estratégia antiplaquetária pós-intervenção personalizada.
• Devem-se utilizar estratégias no laboratório de hemodinâmica para otimizar o diagnóstico etiológico nos casos de MINOCA.
• Deve-se considerar o diagnóstico de disfunção microvascular coronariana, mais frequente em mulheres, tanto em cenários crônicos como agudos, que pode ser corroborado por métodos fisiológicos invasivos coronarianos.

#### 1.2.10. Revascularização Miocárdica e Transplante Cardíaco

• O uso de enxertos arteriais na revascularização miocárdica (RVM) é menor em mulheres.
• Na evolução pós-operatória, as mulheres apresentam maior taxa de complicações.
• A indicação cirúrgica retardada tem impacto negativo nos resultados pós-operatórios.
• Atualmente 25% dos transplantados cardíacos são realizados em mulheres, que apresentam maior taxa de complicações, principalmente rejeição, e têm melhor sobrevida do que os homens após o transplante cardíaco.
• Mulheres evoluem com incidência menor de tumores malignos no pós-operatório.

#### 1.2.11. Reabilitação na Cardiomiopatia Isquêmica

• O encaminhamento à reabilitação cardíaca deve fazer parte da prescrição médica para mulheres com DIC, inclusive nos casos de DEAC e MINOCA.
• A avaliação inicial e a prescrição do programa de reabilitação cardíaca devem ser direcionadas pelas especificidades da mulher para que haja maior engajamento e menor desistência.

## 2. Epidemiologia da Doença Isquêmica do Coração nas Mulheres

### 2.1. Introdução

O crescimento da população e o aumento da expectativa de vida geraram incremento do número total de mortes por DIC no mundo. As taxas de mortalidade por DIC padronizadas por idade em mulheres e homens diminuíram de forma gradual na maioria dos países, provavelmente devido a melhorias no diagnóstico e tratamento, ainda que tenha ocorrido um aumento relevante da obesidade, da elevação da glicose sérica de jejum e da síndrome metabólica.^28-31^

A DIC mantém-se como a principal causa de morte em mulheres e homens no Brasil. A incidência e a prevalência da DIC vêm diminuindo no Brasil ao longo dos últimos 20 anos em mulheres e homens, embora tenha ocorrido aumento na mortalidade precoce por essa causa, entre 18 anos e 55 anos, especialmente nas mulheres. A DIC também foi a segunda maior causa de DALYs nas mulheres no Brasil no período de 1990 a 2019.^31^

As mulheres apresentam maior impacto dos FRCV tradicionais e têm pior prognóstico, apesar de a carga de risco por DIC e a carga aterotrombótica serem menores. As mulheres apresentam maior frequência de FRCV não tradicionais, como estresse mental e depressão, e sofrem maior consequência das desvantagens sociais devidas a raça, etnicidade e renda.^32^

A MINOCA predomina nas mulheres.^29^ Os desfechos são substancialmente piores em comparação aos homens, além disso as mulheres mais jovens (< 55 anos) e os subgrupos de mulheres definidos por raça, etnia, *status* socioeconômico e escolaridade apresentam disparidades ainda mais marcantes quanto a diagnóstico, tratamento e prognóstico da DIC.^29,32^

Este capítulo tem como objetivo sumarizar os achados sobre a epidemiologia da DIC nas mulheres, especialmente as brasileiras.

### 2.2. Mortalidade

Dados recentes do projeto GBD de 2021 estimaram para a DCV no Brasil taxas padronizadas de 3.568,0 DALYs (um DALY representa a perda do equivalente a um ano de plena saúde) e 162,2 mortes por 100 mil habitantes, com uma taxa de prevalência padronizada de 6.905,6 por 100 mil habitantes. Ainda segundo o GBD 2021, as taxas estimadas para a DIC na América Latina Tropical (Brasil e Paraguai) em 2021 foram as seguintes: prevalência de 1.989,5, mortalidade de 67,7 e 1.439,6 DALYs por 100 mil habitantes. Embora um progresso considerável tenha sido feito na diminuição do número de mortes por DCV desde 1980 até o final de 2021, houve um aumento preocupante da taxa de mortalidade bruta e do número de DALYs nos últimos anos por DCV.^28^

A DCV é a principal causa de morte em mulheres no mundo e foi responsável por aproximadamente um terço do total de mortes em mulheres em 2021. A mortalidade por DCV diminuiu globalmente nos últimos 30 anos, com declínio mais significativo em países com alto SDI (SDI = média composta pela renda per capita, nível educacional médio e taxa de fertilidade). No entanto, em regiões de alta renda, a tendência de redução da mortalidade por DCV diminuiu e, em 2017, aumentou o número de mortes em mulheres de alguns países, como Estados Unidos e Canadá.^29^

Na região das Américas, a taxa de mortalidade por DIC ajustada por idade diminuiu no período de 2000 a 2019 nos homens, passando de 149,08 (II_95%_, 138,23; 168,08) para 96,02 (II_95%_, 83,48; 117,19), com decréscimo percentual de -2,3 (II_95%_, -2,5; -2,1), e nas mulheres, passando de 92,36 (II_95%_, 81,35; 109,42) para 54,84 (II_95%_, 45,28; 71,76), com decréscimo percentual de -2,7 (II_95%_, -3,0; -2,5). Nesse mesmo período, as taxas de mortalidade diminuíram significativamente em 24 países. Costa Rica, Canadá e Chile tiveram os maiores decréscimos percentuais, enquanto aumento significativo ocorreu na República Dominicana e em Granada.^30^

**Figura 2.1:**
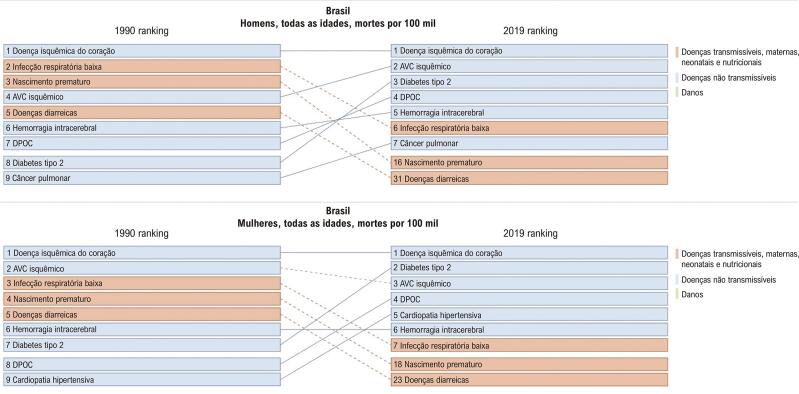
– Ranking das taxas de mortalidade (por 100 mil habitantes) de acordo com sexo, no Brasil, em 1990 e 2019.

**Figura 2.2:**
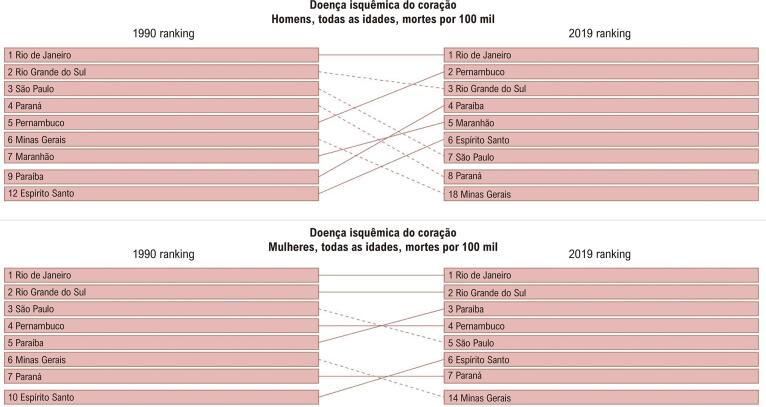
– Ranking das taxas de mortalidade por doença isquêmica do coração (por 100 mil habitantes) de acordo com as unidades da federação e por sexo, no Brasil, em 1990 e 2019.

**Figura 2.3:**
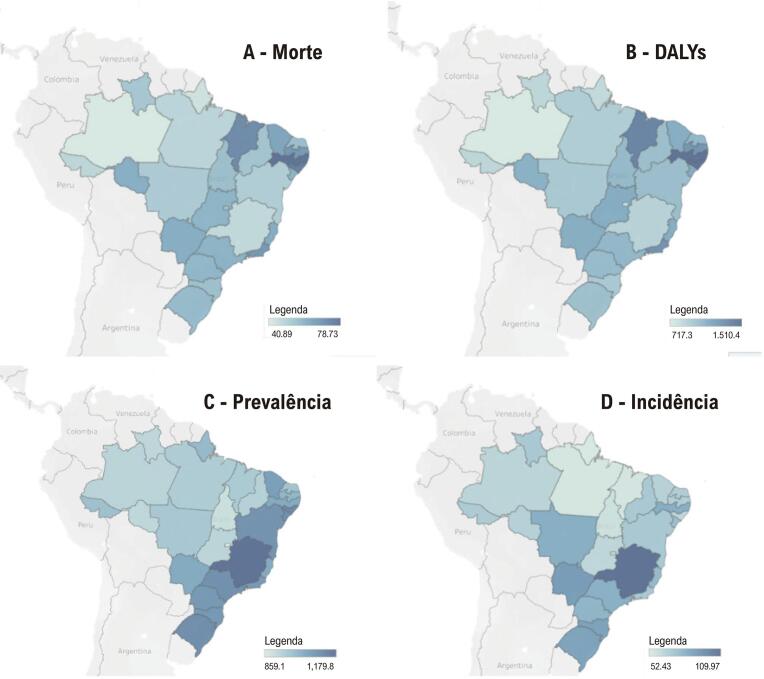
– Doença isquêmica do coração: taxas padronizadas de mortalidade (A), DALYs (B), prevalência (C) e incidência (D), por 100 mil habitantes, de acordo com as unidades da federação brasileira, nas mulheres, em 2019.

No Brasil, de 1990 a 2019, observou-se um declínio na taxa de mortalidade por DCV padronizada nas mulheres. Segundo o estudo GBD 2019, a DIC (definida como indivíduos com infarto do miocárdio prévio, angina estável ou IC isquêmica) foi a principal responsável pela morte de mulheres, seguida por DM tipo 2 e AVC, nessa ordem (Figura 2.1). Esse declínio foi desigual nas unidades da federação em ambos os sexos (Figura 2.2 e Figura 2.3A), sendo relacionado com o envelhecimento da população e o SDI de 2019.

Fonte: Estudo Global Burden of Diseases (GBD) 2019.*^31^*

Fonte: Estudo Global Burden of Diseases (GBD) 2019.*^31^*

Fonte: Estudo Global Burden of Diseases (GBD) 2019.^31^

**Figura 2.4:**
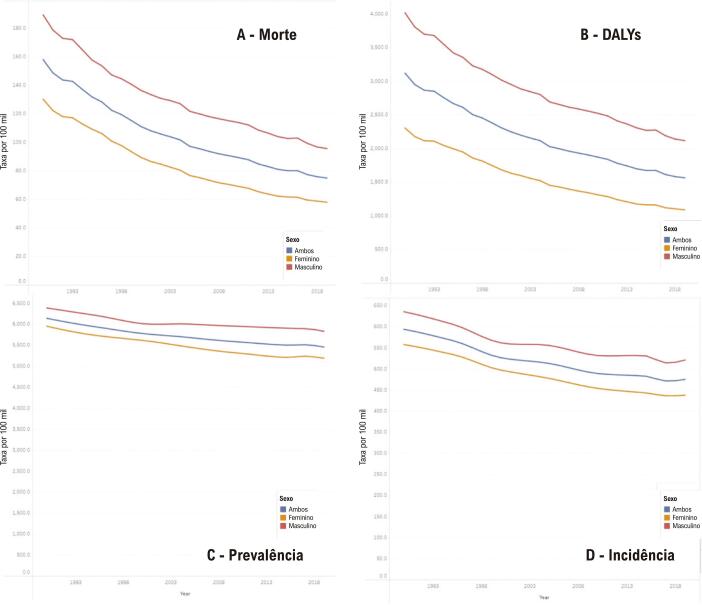
– Doença isquêmica do coração: taxas padronizadas de mortalidade (A), DALYs (B), prevalência (C) e incidência (D), por 100 mil habitantes, de acordo com o sexo, no Brasil, em 2019.

Dados do GBD 2019 estimaram que, em 2019, as taxas de mortalidade padronizadas por idade por DIC foram 58 (II_95%_, 51; 63) e 96 (II_95%_, 88; 101) por 100 mil habitantes no Brasil nas mulheres e nos homens, respectivamente. Entre os anos de 1990 e 2019, houve diminuição mais pronunciada do percentual da taxa de mortalidade por DIC padronizada nas mulheres, -55,5 (II_95%_, -58,7; -52,3), do que nos homens, -49,5 (II_95%_, -52,5; -46,6) (Figura 2.4A). Em todos os grupos etários, as taxas de mortalidade por DIC foram maiores nos homens do que nas mulheres e aumentaram com o envelhecimento em ambos os sexos (Tabela 2.1).^31^

Fonte: Estudo Global Burden of Diseases (GBD) 2019.*^31^*

**Tabela 2.1:** – Números de mortes, taxas de mortalidade, DALYs, prevalência e incidência, por 100 mil habitantes, e variações percentuais das taxas, devido a doença isquêmica do coração, por faixas etárias e sexo, Brasil, 1990 e 2019.

DOENÇAS ISQUÊMICAS DO CORAÇÃO	1990		2019		Variação Percentual da Taxa (II 95%)
	Número (II 95%)	Taxa (II 95%)	Número (II 95%)	Taxa (II 95%)	
**MORTE**					
**Mulheres**					
15-49 anos	3909,7 (3741,1;4083)	10 (9,6;10,5)	3813,4 (3539,1;4106,8)	6,5 (6,1;7)	-35,1 (-40,4;-28,7)
50-69 anos	15601,9 (14922,1;16385,3)	191,1 (182,8;200,7)	20769 (19331,1;22110,3)	97 (90,3;103,2)	-49,3 (-53,2;-44,9)
70+ anos	30524,2 (27413,3;32326,3)	1301,6 (1169;1378,5)	50625,3 (42567,4;55652)	670,5 (563,8;737)	-48,5 (-53,1;-44,6)
Padronizada por idade	50035,8 (46474,1;52421,2)	130,1 (118,1;137,2)	75207,7 (66247,3;81307,1)	57,9 (51;62,6)	-55,5 (-58,7;-52,3)
Todas as idades	50035,8 (46474,1;52421,2)	66,5 (61,7;69,6)	75207,7 (66247,3;81307,1)	67,8 (59,8;73,3)	2 (-6,1;9,8)
**Homens**					
15-49 anos	9180,2 (8829;9551,6)	24,3 (23,4;25,3)	9018,2 (8513,2;9574,1)	15,8 (14,9;16,8)	-35 (-39,3;-30,4)
50-69 anos	29205,2 (28207,5;30291,4)	388,1 (374,8;402,5)	39360,8 (37264,7;41601,2)	207,9 (196,9;219,8)	-46,4 (-50;-42,7)
70+ anos	28825,8 (26903;30176,3)	1529,2 (1427,2;1600,9)	47659,7 (42457,3;51239)	860,6 (766,7;925,3)	-43,7 (-47,9;-40,1)
Padronizada por idade	67211,2 (64502,5;69571)	189,3 (178,2;197)	96038,7 (89069,1;101545,9)	95,6 (87,7;101,3)	-49,5 (-52,5;-46,6)
Todas as idades	67211,2 (64502,5;69571)	91,4 (87,7;94,6)	96038,7 (89069,1;101545,9)	90,8 (84,2;96)	-0,6 (-7,2;5,6)
**INCIDÊNCIA**					
**Mulheres**					
15-49 anos	7197 (5499,8;9046,3)	18,5 (14,1;23,2)	11635,5 (9059,9;14317,5)	19,9 (15,5;24,5)	7,6 (0,9;15,5)
50-69 anos	16567,2 (13251,3;20250,1)	203 (162,3;248,1)	45832,6 (37412,2;55078,3)	214 (174,7;257,2)	5,4 (0,8;11,4)
70+ anos	16711,1 (13816,2;20044,8)	712,6 (589,2;854,8)	43920,1 (37060,4;51680,9)	581,7 (490,8;684,4)	-18,4 (-21,7;-14,7)
Padronizada por idade	40475,2 (35479;45761,4)	88,7 (77,7;99,9)	101388,2 (89389,1;114296,5)	78,2 (69;88,1)	-11,8 (-14,7;-8,5)
Todas as idades	40475,2 (35479;45761,4)	53,8 (47,1;60,8)	101388,2 (89389,1;114296,5)	91,5 (80,6;103,1)	70,1 (64,3;76)
**Homens**					
15-49 anos	13927,2 (11155;17134,5)	36,9 (29,6;45,4)	22270 (17691,1;27177,9)	39,1 (31;47,7)	5,8 (0,2;11,6)
50-69 anos	33048,3 (27279,8;39599,5)	439,2 (362,5;526,2)	84424,7 (70604,7;101021)	446 (373;533,7)	1,6 (-2,6;6,2)
70+ anos	21875,5 (18078,2;25972,6)	1160,5 (959,1;1377,8)	52578,4 (44648;61725,3)	949,5 (806,2;1114,6)	-18,2 (-22,1;-14,6)
Padronizada por idade	68851 (60766,7;77363,5)	167,8 (148;189)	159273,1 (140195,3;178778,9)	148 (130,4;166,3)	-11,8 (-14,7;-9)
Todas as idades	68851 (60766,7;77363,5)	93,6 (82,6;105,2)	159273,1 (140195,3;178778,9)	150,5 (132,5;169)	60,9 (55,6;66,1)
**PREVALÊNCIA**					
**Mulheres**					
15-49 anos	67405,4 (56057,1;81268,7)	173,1 (144;208,7)	124695,9 (103531,2;149594,6)	213,2 (177;255,8)	23,2 (20;26,5)
50-69 anos	204529 (170177,7;249197,2)	2505,7 (2084,9;3053)	534224,8 (445383,3;647280,5)	2494,7 (2079,8;3022,6)	-0,4 (-2,8;2)
70+ anos	212483,8 (175519;255435,7)	9060,9 (7484,6;10892,5)	694914,7 (582274,2;826926,3)	9203,3 (7711,5;10951,6)	1,6 (-1,7;5)
Padronizada por idade	484418,3 (417746,5;563544,8)	1071,8 (925,1;1242,3)	1353835,3 (1172305,1;1562949,3)	1045,6 (904,6;1208,7)	-2,4 (-4,2;-0,5)
Todas as idades	484418,3 (417746,5;563544,8)	643,6 (555;748,7)	1353835,3 (1172305,1;1562949,3)	1221,1 (1057,4;1409,8)	89,7 (85,1;95,2)
**Homens**					
15-49 anos	147353,9 (119423,1;180805,2)	390,8 (316,7;479,5)	280252,9 (227615,4;344765)	491,6 (399,3;604,8)	25,8 (21,9;29,2)
50-69 anos	477021,9 (392697,1;589599,4)	6339 (5218,4;7835)	1231669,7 (1021223,9;1511754,2)	6506,9 (5395,1;7986,6)	2,6 (0,2;5)
70+ anos	371414,9 (301282,3;450276,3)	19703,6 (15983;23887,2)	1138137,6 (940499,4;1375886,1)	20552,3 (16983,4;24845,5)	4,3 (1,6;7,3)
Padronizada por idade	995790,6 (851258,4;1169649,6)	2498,8 (2137;2941,3)	2650060,2 (2275770;3115190,4)	2534 (2170,4;2975,5)	1,4 (-0,4;3,3)
Todas as idades	995790,6 (851258,4;1169649,6)	1353,6 (1157,1;1589,9)	2650060,2 (2275770;3115190,4)	2504,8 (2151;2944,4)	85 (80,7;89,2)
**DALY**					
**Mulheres**					
15-49 anos	189243,4 (181288,6;197784,2)	486 (465,6;508)	182088,5 (169104,9;195954)	311,4 (289,2;335,1)	-35,9 (-41,1;-29,8)
50-69 anos	453002,6 (433556,6;475548,9)	5549,8 (5311,6;5826,1)	607233,3 (565939,9;646284,2)	2835,6 (2642,8;3018)	-48,9 (-52,8;-44,6)
70+ anos	407987,8 (373418,1;429318,3)	17397,8 (15923,6;18307,4)	625969,5 (538771,7;681196,9)	8290,2 (7135,3;9021,6)	-52,3 (-56,1;-49)
Padronizada por idade	1050233,8 (998139;1093952,2)	2303,2 (2162,5;2403,9)	1415291,4 (1291761,5;1506628,9)	1088,4 (992,8;1158,9)	-52,7 (-56,1;-49,3)
Todas as idades	1050233,8 (998139;1093952,2)	1395,3 (1326,1;1453,4)	1415291,4 (1291761,5;1506628,9)	1276,6 (1165,2;1359)	-8,5 (-15,3;-1,6)
**Homens**					
15-49 anos	443883,8 (427008;461879,9)	1177,1 (1132,3;1224,8)	437993 (414351,3;464321,6)	768,3 (726,8;814,5)	-34,7 (-38,8;-30,3)
50-69 anos	873719,6 (844632,7;906253,8)	11610,6 (11224,1;12042,9)	1188364,7 (1122117,2;1256445,4)	6278,1 (5928,1;6637,8)	-45,9 (-49,5;-42,3)
70+ anos	425524,4 (400309;444510,9)	22574,1 (21236,4;23581,3)	679374,4 (620110,3;725589)	12268 (11197,9;13102,6)	-45,7 (-49,2;-42,3)
Padronizada por idade	1743127,7 (1681550;1801264,2)	4013,2 (3852,3;4150,3)	2305732,1 (2173570,4;2429437,7)	2116,5 (1989,9;2232,2)	-47,3 (-50,4;-44)
Todas as idades	1743127,7 (1681550;1801264,2)	2369,5 (2285,8;2448,5)	2305732,1 (2173570,4;2429437,7)	2179,4 (2054,4;2296,3)	-8 (-13,6;-2,2)

Estudo com dados do Brasil oriundos do Sistema de Informação sobre Mortalidade (SIM) reportou que o coeficiente de morte relacionada à DIC permaneceu estável para mulheres nas regiões Norte e Centro-Oeste entre 1981 e 2001, enquanto diminuiu no Sul e Sudeste e aumentou no Nordeste. Para homens, houve tendência decrescente nos eventos nas regiões Sul e Sudeste.^33^

Estudo que avaliou 166.514 procedimentos de angioplastia coronariana para tratamento de DIC realizados em 180 hospitais, entre 2005 e 2008, reportou mortalidade hospitalar média de 2,3% (mínimo 0%, máximo 11,4%), que variou por região geográfica, sendo menor no Sudeste (2,0%) e maior no Norte (3,6%). A taxa de mortalidade foi maior entre mulheres e nos pacientes acima de 65 anos.^34^

As mulheres submetidas a RVM têm maior mortalidade e mais complicações pós-operatórias, apesar de menor carga aterosclerótica. O aumento da mortalidade no momento da RVM é maior em idades mais jovens do mais avançadas, estimando-se um risco três vezes maior de morte em mulheres com idade < 50 anos, apesar do ajuste para os FR.^35^

Estudo realizado no período de 1996 a 2016 com dados do SIM corrigidos por *garbage code* [causas que não devem ser consideradas como causa básica de morte ou são inespecíficas, sendo, portanto, consideradas insuficientes em termos de prevenção, como, por exemplo, códigos I50 da CID-10 (insuficiência cardíaca) e R96 (morte súbita), entre outras] e subnotificação analisou as tendências da mortalidade por IAM de acordo com sexo, regiões do Brasil e residência na capital *versus* não capital. Os autores relataram que a taxa de mortalidade por IAM padronizada por idade diminuiu 44% no país, com diferenças regionais significativas (+5% no Norte, +11% no Nordeste, -35% no Centro-Oeste, -68% no Sudeste e -85% no Sul). As variações temporais foram mais pronunciadas nas mulheres e nas capitais. A taxa padronizada por idade de mortalidade por IAM corrigida diminuiu 49% e 23% entre as mulheres que viviam nas capitais e em outras municipalidades, respectivamente.^36^

Outro estudo realizado com dados do SIM observou um declínio de aproximadamente 2,2% nos últimos 20 anos para o IAM nas regiões geográficas com maior desenvolvimento (Sudeste, Sul e Centro-Oeste), estabilização na região Norte e aumento na região Nordeste. Os autores também previram que essa tendência se prolongará até o ano 2030. Essas variações foram provavelmente relacionadas às melhorias no desenvolvimento social, nos FRCV, no acesso ao sistema de saúde e sua cobertura e à melhor discriminação nas codificações das declarações de óbito nas regiões Norte e Nordeste.^37^

No registro VICTIM, foram avaliados 878 pacientes com diagnóstico de IAM com supradesnivelamento de segmento ST (IAMCSST) admitidos em quatro hospitais com capacidade para realizar angioplastia primária em Sergipe, sendo um público e três privados, no período de dezembro de 2014 a junho de 2018. Desses pacientes, 33,4% eram mulheres. Do total, apenas 53,3% dos pacientes foram submetidos à reperfusão miocárdica (134 mulheres *versus* 334 homens). As mulheres apresentaram taxas significativamente menores de angioplastia primária (44% *versus* 54,5%; p = 0,003) e significativamente maiores de mortalidade hospitalar (16,1% *versus* 6,7%; p < 0,001) do que os homens.^38^

Outro estudo unicêntrico prospectivo realizado em Recife com 709 pacientes consecutivos com IAMCSST (36% de mulheres; média de idade, 61 anos), no período de fevereiro de 2018 a fevereiro de 2019, observou que as mulheres eram mais velhas (63,13 anos *versus* 60,53 anos, p = 0,011), mais frequentemente apresentavam HAS (75,1% *versus*. 62,4%, p = 0,001), DM (42,2% *versus* 27,8%, p < 0,001) e dislipidemia (34,1% *versus* 23,9%, p= 0,004), além de serem menos submetidas à intervenção coronariana percutânea (ICP) por acesso radial (23,7% *versus* 46,1%, p < 0,001) do que os homens. A taxa de mortalidade intra-hospitalar foi significativamente maior nas mulheres em comparação com os homens (13,2% *versus* 5,6%, p = 0,001) e sexo feminino mostrou-se um preditor independente de mortalidade intra-hospitalar (OR 2,79; IC 95%, 1,15 – 6,76; p = 0,023).^39^

### 2.3. Prevalência e Incidência

Segundo dados do estudo GBD 2019, a taxa de prevalência de DIC padronizada por idade no Brasil foi de 1.046 (II_95%_, 905; 1.209) por 100 mil mulheres e 2.534 (II_95%_, 2.170; 2.975) por 100 mil homens (Tabela 2.1).^31^Houve uma diferença entre as regiões brasileiras na prevalência padronizada por idade de DIC nas mulheres, que foi maior nas regiões Sudeste e Sul e menor na região Norte (Figura 2.3C).^31^ Houve redução do percentual da taxa padronizada de prevalência de DIC por 100 mil habitantes nas mulheres entre os anos de 1990 e 2019, de -2,4 (II_95%_, -4,2; -0,5), mas discreto aumento nos homens, de 1,4 (II_95%_, -0,4; 3,3), nesse mesmo período (Tabela 2.1 e Figura 2.4C).

O estudo GBD 2019 estimou uma incidência de DIC de 260.661 (II_95%_, 230.100-293.617) eventos (principalmente infarto do miocárdio) no Brasil em 2019. Em todos os grupos etários, a DIC teve maior incidência nos homens do que nas mulheres (Tabela 2.1). Em 2019, a taxa de incidência de DIC padronizada por idade foi 78 (II_95%_, 69; 88) por 100 mil mulheres e 148 (II_95%_, 130; 166) por 100 mil homens. Houve uma diferença entre as regiões brasileiras na incidência de DIC padronizada por idade nas mulheres, que foi maior nas regiões Sudeste e Sul e menor na região Norte (Figura 2.3D).^31^ Houve redução do percentual da taxa padronizada de incidência de DIC por 100 mil habitantes entre os anos de 1990 e 2019 nas mulheres, -11,8 (II_95%_, -14,7; -8,5), e nos homens, -11,8 (II_95%_, -14,7; -9), nesse mesmo período (Tabela 2.1 e Figura 2.4D).

A prevalência do IAM em mulheres é menor nas jovens do que em outras faixas etárias, mas existem tendências preocupantes nos últimos anos, dado que a proporção atribuível a pacientes jovens (35-54 anos) aumentou de 27% para 32% nas últimas duas décadas, com maior aumento de mulheres jovens (21% a 31%).^40^

A prevalência de MINOCA é maior nas mulheres. O estudo VIRGO (*Variation in Recovery: Role of Gender on Outcomes of Young AMI Patients*), realizado entre 2008 e 2012, incluiu prospectivamente 2.690 pacientes com IAM e idades entre 18 anos e 55 anos em 103 hospitais, na proporção de mulheres para homens de 2:1. Nos 2.374 pacientes submetidos a estudo angiográfico coronariano, as mulheres apresentaram cinco vezes mais chances de ter MINOCA do que os homens (14,9% *versus* 3,5%; OR 4,84; IC 95%, 3,29 – 7,13). As mulheres com obstrução coronariana significativa eram mais propensas a estar na menopausa (55,2% *versus* 41,2%; p < 0,001) ou ter um histórico de diabetes gestacional (16,8% *versus* 11,0%; p = 0,028). Cabe ressaltar que a mortalidade de 1 e 12 meses nos pacientes com MINOCA foi semelhante à mortalidade por obstrução coronariana nos mesmos 1 e 12 meses.^30^

### 2.4. Carga de Doenças

**Figura 2.5:**
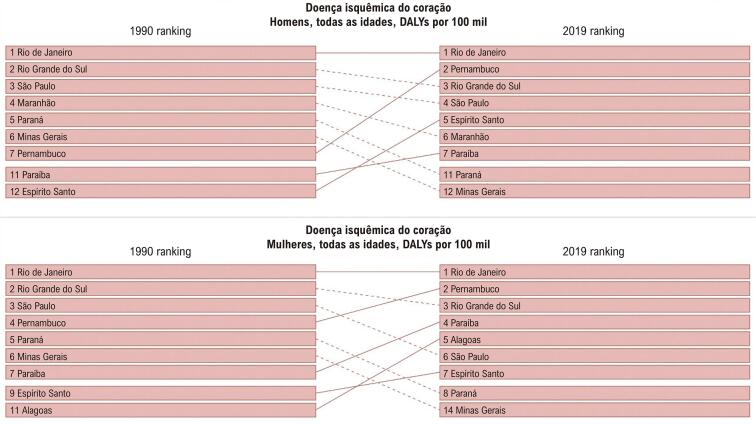
– Ranking das taxas de DALYs devidos a doença isquêmica do coração, por 100 mil habitantes, de acordo com as unidades da federação e por sexo, no Brasil, em 1990 e 2019.

O estudo do GBD 2019 estimou taxa padronizada de DALYs por DIC por 100 mil habitantes de 1.088,4 (992,8; 1.158,9) nas mulheres e de 2.116,5 (II_95%_, 1.989,9; 2.232,2) nos homens (Tabela 2.1). Em 2019, DIC foi a segunda causa mais comum de DALYs no Brasil em mulheres, após complicações neonatais, e homens, após violência interpessoal.^31^ Essas taxas foram heterogêneas nas regiões geográficas brasileiras e a tendência das taxas de DALYs padronizadas por idade de 1990 a 2019 nas mulheres assemelhou-se à das taxas de mortalidade (Figura 2.3B). As unidades da federação com maior número de DALYs perdidos por DIC por 100 mil habitantes nas mulheres em 2019 foram Rio de Janeiro, Pernambuco, Rio Grande do Sul, Paraíba, Alagoas e São Paulo, nessa ordem (Figura 2.5).

Fonte: Estudo Global Burden of Diseases (GBD) 2019.*^31^*

Entre os anos de 1990 e 2019, houve maior redução do percentual da taxa padronizada de DALYs por DIC por 100 mil habitantes nas mulheres, -52,7 (II_95%_, -56,1; -49,3), do que nos homens, -47,3 (II_95%_, -50,4; -44) (Tabela 1 e Figura 4B).

**Figura 4.1:**
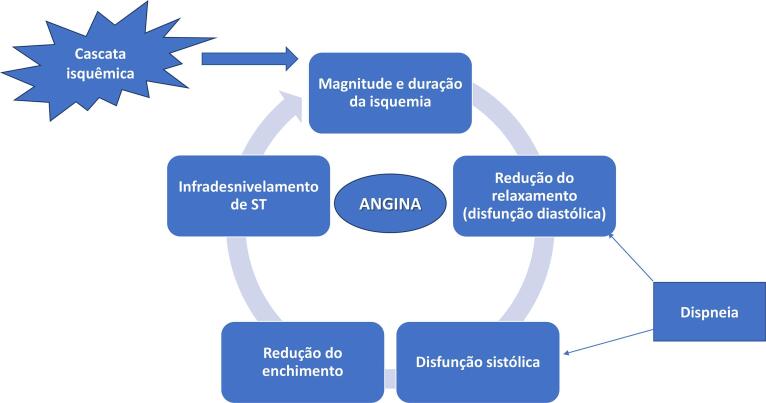
– Fisiopatologia da cascata isquêmica de equivalentes anginosos.

### 2.5. Fatores de Risco

Os FR tradicionais, como HAS, hiperlipidemia, DM, tabagismo, dieta pouco saudável e sedentarismo, são prevalentes nas mulheres com DIC, mas caminham lado a lado com os FR emergentes nas mulheres, como distúrbios metabólicos, distúrbios relacionados à gravidez, distúrbios autoimunes, apneia do sono, doenças crônicas, baixo nível socioeconômico, *burnout* e fatores psicossociais, como depressão e ansiedade.^41^

No estudo PURE (*Prospective Urban Rural Epidemiological Study*), 202.072 indivíduos com idade entre 35 anos e 70 anos, de comunidades urbanas e rurais, em 27 países, entre janeiro de 2005 e maio de 2019, foram acompanhados por uma média de 9,5 (IQR: 8,5–10,9) anos. As mulheres tiveram uma menor carga de FRCV usando dois escores de risco tradicionais diferentes (INTERHEART e Framingham). Estratégias de prevenção primária (estilo de vida saudável e uso de medicamentos comprovados) foram mais frequentes em mulheres, que, por sua vez, apresentaram menor incidência de DCV. No entanto, tratamentos de prevenção secundária para DIC foram menos frequentes em mulheres do que em homens. As diferenças entre mulheres e homens em relação a tratamentos e resultados foram mais marcantes em países de baixa e média renda, com poucas diferenças em países com alta renda, naqueles com ou sem DCV e DIC prévias.^42^

**Figura 2.6:**
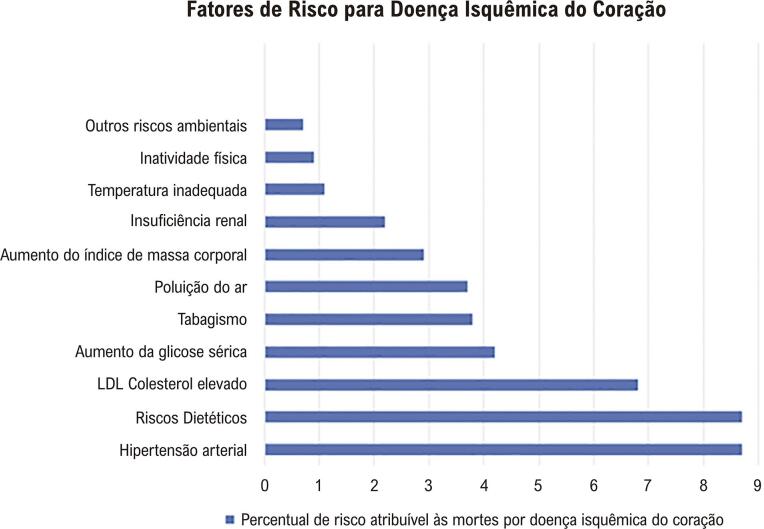
– Ranking dos fatores de risco atribuíveis a doença isquêmica do coração no mundo, em ambos os sexos, em 2021.

Os FR atribuíveis a DIC podem ser vistos na Figura 2.6. A HAS e os riscos dietéticos lideram o *ranking* dos FR atribuíveis a DIC, em ambos os sexos, mundialmente.^28^

Fonte: Estudo Global Burden of Diseases (GBD) 2019.*^28^*

No estudo caso-controle derivado do estudo VIRGO, com 2.264 pacientes com IAM (18-55 anos) e 2.264 controles pareados, 3.122 (68,9%) eram mulheres, com mediana de idade de 48 (44-52) anos. Os seguintes sete FR representaram coletivamente a maior parte do risco total de IAM em mulheres (83,9%) e homens (85,1%): DM [OR 3,59 (IC 95%, 2,72-4,74) em mulheres *versus* 1,76 (1,19-2,60) em homens]; depressão [OR 3,09 (IC 95%, 2,37-4,04) em mulheres *versus* 1,77 (1,15-2,73) em homens]; HAS [OR 2,87 (IC 95%, 2,31-3,57) em mulheres *versus* 2,19 (1,65-2,90) em homens]; tabagismo atual [OR 3,28 (IC 95%, 2,65-4,07) em mulheres *versus* 3,28 (2,65-4,07) em homens]; história familiar de infarto prematuro do miocárdio [OR 1,48 (IC 95%, 1,17-1,88) em mulheres *versus* 2,42 (1,71-3,41) em homens]; baixa renda familiar [OR 1,79 (IC 95%, 1,28-2,50) em mulheres *versus* 1,35 (0,82-2,23) em homens]; hipercolesterolemia [OR 1,02 (IC 95%, 0,81-1,29) em mulheres *versus* 2,16 (1,49-3,15) em homens]. Houve diferenças significativas entre os sexos nas associações de FR: HAS, depressão, DM, tabagismo atual e história familiar de DM tiveram associações mais fortes com IAM em mulheres jovens, enquanto a hipercolesterolemia teve uma associação mais forte em homens jovens.^43^

Estudo com 10.112 pacientes (29% mulheres) com DIC recrutados na Europa, Ásia e Oriente Médio entre 2012 e 2013 relatou que, em comparação com os homens, as mulheres eram menos propensas a atingir as metas de colesterol total [OR 0,50 (IC 95%, 0,43-0,59)], colesterol de lipoproteína de baixa densidade [OR 0,57 (IC 95%, 0,51-0,64)] e glicose [OR 0,78 (IC 95%, 0,70-0,87)], ou ser fisicamente ativas [OR 0,74 (IC 95%, 0,68-0,81)] ou não obesas [OR 0,82 (IC 95%, 0,74-0,90)]. Em contraste, as mulheres tiveram melhor controle da PA [OR 1,31 (IC 95%, 1,20-1,44)] e eram mais propensas a não fumar [OR 1,93 (IC 95%, 1,67-2,22)] do que os homens. Os autores concluíram que o controle para prevenção secundária dos FR relacionados com a DIC foi geralmente pior nas mulheres do que nos homens.^44^

### 2.6. Conclusão

A DIC contribui significativamente para a morbimortalidade das mulheres. Embora as taxas de mortalidade, DALYs, prevalência e incidência de DIC tenham diminuído ao longo dos últimos 20 anos, os dados indicam que a mortalidade por DIC nas mulheres jovens entre 35 anos e 54 anos está aumentando. O reconhecimento, a discussão, a educação e o tratamento apropriado da DIC nas mulheres são necessários para reduzir os *gaps* no diagnóstico e tratamento, além dos desfechos desfavoráveis nas mulheres.

## 3. Bases Fisiopatológicas da Doença Aterotrombótica

### 3.1. Introdução

**Figura 3.1:**
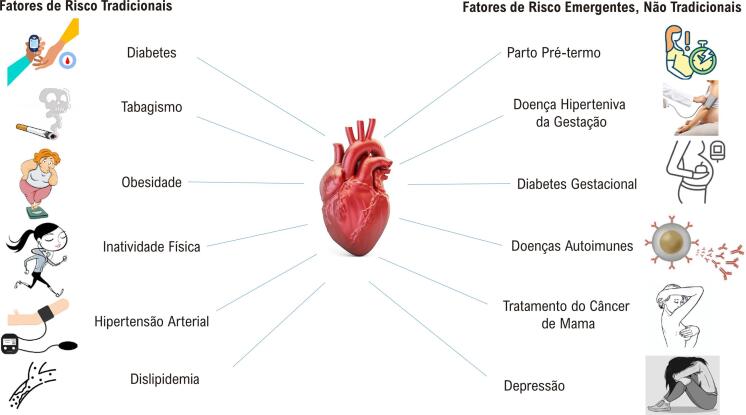
– Fatores de risco tradicionais e não tradicionais para a doença aterosclerótica cardiovascular na mulher.

O reconhecimento de FR tradicionais para a DCV aterosclerótica, bem como de FR emergentes e não tradicionais únicos ou mais frequentes nas mulheres, e de seus diferentes impactos contribui para o novo entendimento dos mecanismos que levam aos piores desfechos nas mulheres (Figura 3.1). Maior detalhamento sobre a importância dos FR no sexo feminino pode ser visto no capítulo subsequente.

As apresentações podem ser agudas, como MINOCA, que representa 3% a 15% dos casos, ou estarem relacionadas a casos de INOCA.^45,46^ O critério diagnóstico para MINOCA é a presença de IAM sem lesões obstrutivas (> 50%) em qualquer artéria epicárdica e ausência de diagnóstico alternativo.^45-47^

Os mecanismos fisiopatológicos envolvidos na MINOCA incluem ruptura de placa coronariana, DEAC, vasoespasmo, disfunção microvascular coronariana e embolismo/trombose. Importante destacar síndromes que mimetizam clinicamente MINOCA, como Takotsubo, miocardite e cardiomiopatia não isquêmica.^48^

### 3.2. Ruptura de Placa

É considerado um termo amplo que envolve ruptura, erosão e nódulos calcificados nas artérias. A ruptura da placa com formação trombótica é a manifestação usual das SCA, mas pode não obstruir a luz do vaso. A erosão é a formação de trombo adjacente à superfície luminal, resultado de apoptose celular e recrutamento de neutrófilos. Os nódulos calcificados usualmente são identificados por imagem intravascular como protrusões na luz do vaso, podendo ocorrer fratura dos nódulos com acúmulo de fibrina na capa fibrosa.^48^ O sexo feminino e tabagismo estão associados à maior incidência de erosão do que de ruptura da placa. As alterações intravasculares podem ser detectadas por ultrassom ou tomografia de coerência óptica (OCT). O Estudo HARP demonstrou que ruptura de placa foi a causa mais frequente de MINOCA, sendo evidenciada em 43% das mulheres com essa condição.^49^

### 3.3. Dissecção Espontânea de Coronária

A DEAC resulta da formação de um falso lúmen arterial na ausência de doença aterosclerótica significativa. Existem dois mecanismos descritos, um envolvendo laceração da camada íntima e resultando em falso lúmen (*inside-out*) e outro onde ocorre uma hemorragia intramural com ou sem ruptura da camada íntima (*outside-in*).^48^ Os fatores predisponentes são múltiplos, como fatores genéticos, sexo feminino, gestação e terapia com estrógenos, displasia fibromuscular, doenças inflamatórias sistêmicas, além de fatores externos, como estresse emocional, atividade física intensa e uso de drogas estimulantes. Dados de registros internacionais apontam para DEAC como a principal causa de IAM no período perigestacional, ocorrendo mais no terceiro trimestre da gestação e logo após o parto.^50^

De acordo com as características angiográficas, quatro tipos de DEAC são descritos: tipo 1: parede arterial evidente com múltiplos lúmens radiolúcidos; tipo 2: um segmento com estreitamento difuso (normalmente > 20mm) e segmento normal proximal e distalmente (tipo 2A) ou estreitamento difuso que se estende até a extremidade distal do vaso (tipo 2B); tipo 3: um segmento curto de estenose (< 20mm) que mimetiza aterosclerose; tipo 4: caracterizado por dissecção com oclusão total abrupta de um segmento coronariano distal. De acordo com as diretrizes internacionais, a DEAC que resulta em obstrução tipo 4 não configura MINOCA, sendo que os demais tipos com lesões não obstrutivas ou não identificadas na angiografia são incluídos nessa classificação.^51^

### 3.4. Espasmo Coronariano

O espasmo coronariano como causa de isquemia é diagnosticado quando há dor torácica, com ou sem alteração isquêmica no ECG e vasoconstrição > 90% na angiografia, espontânea ou induzida com acetilcolina ou ergonovina. O mecanismo fisiopatológico é uma hiper-reatividade da camada muscular tanto das artérias epicárdicas quanto da microcirculação, embora não completamente elucidado.^52^ Alguns indivíduos têm gatilhos distintos que induzem espasmo, como estresse, hiperventilação, períodos diurnos e ciclos sazonais (*clusters*), sugerindo alterações intracelulares e pós-receptores relacionados com a hiper-reatividade. O tabagismo e a etnia asiática são descritos como fatores predisponentes, enquanto os demais FR tradicionais não parecem relacionados a maior risco. Alguns estudos sugerem que as mulheres são mais sensíveis a espasmo induzido por acetilcolina que ergonovina.^53^

### 3.5. Disfunção Microvascular Coronariana

A circulação coronariana microvascular compreende vasos com calibre inferior a 0,5mm de diâmetro, não visualizados na angiografia convencional, embora representem mais de 70% da resistência vascular coronariana.^54^A disfunção microvascular coronariana é definida como alteração dessa reserva de fluxo coronariano (RFC) ou o aumento da resistência intramiocárdica na ausência de obstrução de artérias epicárdicas. A avalição da RFC pode ser feita através de medidas não invasivas, com doppler ou termodiluição. A RFC é expressa como a razão entre o fluxo em hiperemia máxima e o fluxo em repouso. Um valor < 2,0 é considerado alterado. O índice de resistência máximo é calculado como o produto da pressão coronariana distal em máxima hiperemia e o tempo do fluxo médio, sendo um valor ≥ 25 indicativo de disfunção da microcirculação. O diagnóstico de doença da microcirculação foi recentemente proposto pelo COVADIS (*Coronary Vasomotor Disorders International Study*) *Group*, levando em consideração achados de isquemia, ausência de doença obstrutiva e evidência de função microvascular alterada.^54^

Do ponto de vista fisiopatogênico, as causas parecem ser diversas e complexas. Dois endótipos são descritos: estrutural e funcional, que muitas vezes coexistem. No aspecto estrutural, existe disfunção endotelial, remodelamento arteriolar e rarefação de capilares, o que leva a uma diminuição do incremento do fluxo coronariano em repouso e aumento da demanda durante esforço. A manifestação funcional está relacionada ao acoplamento cardíaco-coronário ineficiente, resultado do aumento do tônus microvascular durante pico do exercício e durante o repouso, levando a uma maior demanda de oxigênio do miocárdio no cenário de reserva vasodilatadora esgotada saturada.^48,52^

Vários estudos demonstraram diferenças relacionadas ao sexo na resposta aos testes funcionais, indicando que as mulheres apresentam mais disfunção microvascular coronariana e vasoespasmo epicárdico em comparação com os homens.^53^

### 3.6. Embolia e Trombose

A embolização de artéria epicárdica sem substrato de aterosclerose é uma causa incomum de MINOCA e, usualmente, um diagnóstico de exclusão ou presuntivo.^55^ O *National Cerebral and Cardiovascular Center Group* classifica essa condição como possível quando existe evidência de embolia/trombose em artéria coronária sem aterosclerose, embolização concomitante para outras artérias ou múltiplos locais e/ou na evidência de fatores predisponentes para formação de êmbolos. Várias causas são descritas para fonte de êmbolos, como diretas, paradoxais, iatrogênicas ou mesmo hipercoagulabilidade sistêmica. As fontes diretas incluem trombos formados no apêndice atrial esquerdo (AAE) na vigência de FA, no VE e nas válvulas mitral e aórtica, bem como em segmentos coronarianos proximais. Os êmbolos podem ter origem hematogênica ou de conteúdo tissular outro, como neoplasia e *debris* valvares. Indivíduos com *shunts* direito-esquerdo, como a presença de comunicação atrial, forame oval patente, malformações arteriovenosas, podem apresentar embolização paradoxal. O incremento de procedimentos invasivos coronarianos, valvares e de acesso sistêmico também tem sido relacionado com casos de embolização coronariana.^45,55^

### 3.7. Síndrome de Takotsubo

A síndrome de Takotsubo é uma entidade cardíaca aguda, com apresentação clínica semelhante à de uma SCA.^56^ Várias apresentações clínicas e fenotípicas são reconhecidas, sendo a típica um perfil de anormalidade de contração ventricular esquerda circunferencial (bi-) que se estende além de um território de suprimento da artéria coronária e parece seguir a inervação simpática cardíaca anatômica. A síndrome afeta predominantemente mulheres na pós-menopausa, sendo, em mais de 70% dos casos, precedida por estresse emocional ou físico. Há um desequilíbrio entre os sexos, com mulheres representando 90% dos casos de síndrome de Takotsubo, responsável por até 10% das mulheres na pós-menopausa com dor torácica aguda. As diferenças de gênero na fisiopatologia permanecem pouco descritas e são uma área para pesquisas futuras. A patogênese da síndrome de Takotsubo é indefinida. Vários mecanismos fisiopatológicos envolvendo isquemia miocárdica (espasmo coronariano multiarterial, disfunção microvascular, infarto do miocárdio abortado), obstrução da via de saída do VE, toxicidade miocárdica por catecolaminas transmitidas pelo sangue, mudança induzida por epinefrina no tráfego de sinal e disfunção do sistema nervoso autônomo (SNA) foram propostos.^56,57^

### 3.8. Miocardite

A miocardite é uma doença inflamatória do coração que pode ser causada por infecção, ativação do sistema imune ou toxicidade, sendo a causa mais frequente a infecciosa. Uma das apresentações comuns da miocardite é a mimetização de quadro de IAM, com dor precordial e aumento de troponina, usualmente em homens jovens. Os aspectos fisiopatológicos variam de acordo com o agente agressor e a etiologia, mas envolvem degeneração e necrose do cardiomiócito de origem não isquêmica. Em uma série de casos de miocardite por parvovírus B19, a indução de espasmo coronariano epicárdico reproduziu os sintomas, sugerindo que parte da sintomatologia pode ser de origem coronariana e estar relacionada a uma arterite induzida pelo vírus.^58^

## 4. Apresentação Clínica, Diagnóstico e Tratamento Clínico

### 4.1. Dor Torácica de Etiologia Isquêmica

A dor torácica é a segunda queixa mais comum nos serviços de emergência, superada apenas pelas causas traumáticas.^59,60^ Acomete entre 20% e 40% da população,^61^ sendo mais frequente nas mulheres. É preciso, entretanto, conhecer as diferenças na apresentação clínica e no diagnóstico e manejo da DIC entre mulheres e homens.^60^Apenas 5,1% dos pacientes com dor torácica na emergência apresentam SCA e mais da metade mostra etiologia não cardíaca.^59^

Até recentemente, as publicações raramente se voltavam para diferenças sexo-específicas nas DCV, nem compreendíamos as diferenças fisiopatológicas que afetam exclusivamente as mulheres^32^ (Figura 4.1). A falta de reconhecimento das diferenças biológicas e socioculturais específicas do sexo na apresentação da dor torácica da DIC pode, em parte, explicar essas disparidades, o que leva a atrasos no diagnóstico e tratamento. Os sintomas isquêmicos das mulheres são mais relacionados ao estresse emocional ou mental e menos frequentemente precipitados pela atividade física em comparação aos dos homens. Diferenças de sexo e gênero nos mecanismos de dor, incluindo suscetibilidade psicológica, reatividade do SNA e inervação visceral, provavelmente contribuem para as diferenças na apresentação da dor torácica. Portanto, escores de risco e caracterização de angina típica/atípica não parecem mais relevantes e não devem ser usados uniformemente em mulheres e homens.^60^

As diferenças entre os sexos na percepção da dor são bem descritas, tendo o sexo feminino maior consciência somática em comparação ao masculino. Isso potencialmente leva as mulheres a terem maior sensibilidade, mas menor especificidade para dor torácica cardíaca. Mulheres mais jovens na pré-menopausa com níveis relativamente altos de estrogênio têm uma maior percepção da dor em comparação às mais velhas na pós-menopausa com níveis mais baixos de estrogênio.^62^

Uma avaliação abrangente das diferenças de sexo e gênero na dor inclui contribuições de causa próxima de experiência (abuso, trabalho de parto e parto), psicológica (ansiedade, depressão, estresse pós-traumático), genética (impressão do cromossomo X/cromossomo Y), neuroquímica (adenosina, expressão de citocinas), organizacional (ação de esteroides no desenvolvimento), ativacional (ação de esteroides na idade adulta), nível de sistemas (conectividade cortical, modulação do nervo vago) e sociocultural (papéis de gênero, expectativas de papéis de gênero).^62^

A isquemia desencadeada por estresse mental prediz mortalidade duas vezes maior e a resposta autonômica anormal ao estresse leva ao aumento da reatividade vascular coronariana.

Na história clínica de dor torácica em mulheres, é fundamental incluir, além das manifestações de DCV, os FR clássicos, os FR específicos do sexo feminino e os determinantes psicossociais da saúde, notadamente depressão e estresse. Em geral, a dor isquêmica miocárdica localiza-se no tórax, porém pode ocorrer em qualquer região desde o epigástrio até a mandíbula ou os dentes, entre as omoplatas ou em qualquer dos braços até o pulso e os dedos.^63^

As mulheres geralmente apresentam sintomas de dor semelhantes aos dos homens, porém têm maior prevalência de outros sintomas, como palpitações, dispneia, dor maxilar, cervical e no dorso. Um subestudo do ensaio clínico High-STEACS (troponina de alta sensibilidade na avaliação de pacientes com SCA com 1.941 pacientes, sendo 39% mulheres) avaliou a dor torácica de forma prospectiva e com descritores de natureza típica, presença de radiação e de sintomas adicionais mais comuns em mulheres com suspeita da doença. Em comparação aos homens, as mulheres mais frequentemente relataram palpitações como sintoma inicial (11% *versus* 7%), irradiação da dor para o braço esquerdo (36% *versus* 31%), dorso (31% *versus* 17%) ou pescoço/mandíbula (28% *versus* 20%) e maior propensão a náusea (34% *versus* 22%).^64^

Dispneia pode acompanhar a dor ou desconforto torácico e sintomas menos específicos, como fadiga ou desmaio, náusea, queimação, inquietação ou sensação de morte iminente, podem ocorrer. A duração do desconforto é breve, em geral menos de dez minutos.^65^ Um aspecto peculiar de manifestação da dor isquêmica na mulher seria a sensação de desconforto torácico, mesmo na SCA grave. Apesar da variabilidade individual, o sofrimento induzido pela isquemia miocárdica costuma ser característico e, portanto, fundamental para o diagnóstico. Por isso, critérios mais prováveis de serem associados à isquemia foram descritos como “típicos”, mais comuns nos homens, e “atípicos” (náuseas, desconforto, epigastralgia), mais comuns nas mulheres. Porém, a denominação de dor “atípica” como característica mais feminina deve ser evitada, devendo-se pensar em padronizar a avaliação dos sintomas de isquemia miocárdica na mulher. Além disso, “dor torácica atípica” é um termo problemático, pois, embora tenha a intenção de indicar ausência de sintomas “típicos”, sugere origem não cardíaca, devendo, assim, ser desencorajado o seu uso.^59^

Uma melhor compreensão dos sintomas femininos de angina e uma estratificação de risco mais adaptada às mulheres permitem o reconhecimento precoce, diagnóstico correto e tratamento ideal, melhorando o prognóstico e a evolução da DIC nas mulheres.

“Dor no peito” significa, além disso, dor, pressão, aperto ou desconforto no tórax, ombros, braços, pescoço, costas, parte superior do abdome ou mandíbula, bem como dispneia e fadiga, que devem ser considerados como equivalentes anginosos. Portanto, são sintomas inespecíficos, descritos até mesmo como fraqueza, tontura, diaforese e dispepsia, podendo ocorrer como manifestações de isquemia miocárdica em mulheres.^66^A dor ou desconforto torácico ocorre em mais de 80% de mulheres e homens com SCA, porém as mulheres atribuem mais esse sintoma a condição não cardíaca, como refluxo, estresse ou ansiedade, o que pode prejudicar o diagnóstico.^67^Nas mulheres, ocorrem mais sintomas adicionais do que nos homens, sendo elas mais propensas a se apresentarem sem dor torácica.

Por terem as mulheres formas de apresentação da SCA diferentes, elas correm maior risco de diagnóstico incorreto e atraso no diagnóstico e tratamento, o que pode levar a eventos catastróficos, como parada cardíaca. Sabe-se que pacientes com dispneia de origem cardíaca como manifestação única têm risco duas vezes maior de morte súbita.^68^

Mulheres com SCA tendem a ser mais idosas e, portanto, têm mais comorbidades do que homens. Apresentam sintomas contínuos, tentam automedicação em casa e informam sintomas semelhantes no passado.

A idade jovem e a ausência de desconforto torácico estão entre os preditores mais fortes de diagnóstico errôneo de SCA e de alta hospitalar de setores de emergência de forma precoce ou inadequada. Mulheres que receberam alta com o diagnóstico de dor torácica inespecífica podem ter duas vezes mais eventos coronarianos subsequentes.^67^

Os escores de risco criados para estratificar pacientes não são perfeitos, notadamente em mulheres, onde fatores emergentes e específicos não são considerados. O escore HEART teve melhor desempenho do que os escores TIMI, GRACE e EDACS na previsão de eventos adversos em pacientes de emergência que apresentaram dor torácica ou equivalentes anginosos, mas 5,7% dos pacientes classificados como baixo risco apresentaram eventos.^12^No escore HEART, as considerações clássicas para a estratificação são História, ECG, Idade (*Age*), Fatores de risco e Troponina, sendo a pontuação a soma dessas cinco variáveis. Como as mulheres têm apresentação clínica variável, alterações ao ECG muitas vezes dúbias e FR específicos do sexo feminino, aquele e muitos outros escores têm potência reduzida para estratificar essas pacientes.^68^

No âmbito da cardiologia, conhecer os FR permite ao profissional de saúde exercer estratégias de prevenção que podem, a médio ou longo prazo, alterar prognóstico e desfecho da DCV.^69^

Diante das divergências anatômicas e metabólicas entre os sexos, deve-se, ao estudar grupos de risco, considerar essa distinção. Existem, portanto, diferentes proporções na relevância dos FR, explicitando a importância de estudá-los separadamente.^69,70^ Relevante se faz abordar FR bem estabelecidos (modificáveis e não modificáveis) e os específicos do sexo feminino. Alguns fatores de impacto na saúde cardiovascular da mulher são sub-reconhecidos, como depressão, violência doméstica e fatores psicossociais, e merecem ser abordados.^32^ Em geral, os FR manifestam-se conjuntamente, sendo que, quanto mais FR a paciente apresentar, maior é o seu RCV global.^29^

### 4.2. Fatores de Risco Estabelecidos e Modificáveis

• **Obesidade/Sobrepeso:** A obesidade é uma doença e, para mulheres em idade reprodutiva, é o fator modificável que mais diretamente se associa à presença de HAS. A obesidade na gestação promove maior risco de desenvolver doença hipertensiva da gravidez e/ou diabetes gestacional, que exercem papel importante na mortalidade materno-fetal. No Framingham Heart Study, a obesidade aumentou o risco de doença coronariana em 64% nas mulheres e em 46% nos homens.^29,71^
• **Sedentarismo:** É o FR que tem impacto direto na DCV desde a primeira infância.^29^ Desde os primeiros anos de vida, a mulher tem progressivamente menor participação em atividade física, o que leva a uma cascata de alterações produzidas pela falta de condicionamento físico, com grande relevância clínica no processo de envelhecimento não saudável.^29,67^ Em países onde a mulher está sob limitações sociais/religiosas, há piora dos dados cardiovasculares, dificultando a promoção da saúde e promovendo maior prevalência de outros FR, como obesidade.^29^
• **Tabagismo:** É um hábito que se apresenta de forma epidêmica. Sua prevalência em homens é maior que em mulheres; entretanto, estudos recentes demostram que o RCV associado ao tabagismo é 25% maior em mulheres do que em homens.^29^ Importante lembrar que o cigarro eletrônico tem se consolidado como prática popular, atingindo internacionalmente cada vez mais mulheres jovens, contribuindo para a formação de lesões endoteliais. Em todas as faixas etárias, exceto dos 30-44 anos, as mulheres têm um risco 25% maior de DIC associada ao tabagismo em comparação aos homens.^29,67^
• **Dislipidemia:** É um FR relacionado ao estilo de vida associado a fatores genéticos, mas estudos mostram que há menor atenção à saúde da mulher e às propostas terapêuticas em comparação às do homem. Fator agravante a ser considerado é a menopausa, devido ao aumento relevante nas concentrações de colesterol total e LDL-colesterol e menor efeito protetor do HDL-colesterol, levando assim, a maior predisposição a DCV. As estratégias de tratamento são as bem estabelecidas nas diretrizes, não diferindo das estratégias dos homens. Porém, cabe ressaltar que a regressão do ateroma e o benefício da redução dos níveis de LDL-colesterol parecem maiores em mulheres do que em homens.^29,71^
• **Diabetes mellitus:** Mulheres com DM possuem um risco de DIC fatal três vezes maior do que mulheres não diabéticas. O infarto do miocárdio ocorre mais cedo e existe maior mortalidade em mulheres do que em homens com DM. Mulheres diabéticas apresentam risco significativamente maior de desenvolver doença arterial crônica e/ou ICFEp do que homens.^67^ Considerando o aumento mundial de DM, diretamente relacionado ao aumento da obesidade, mulheres com DM apresentam 44% maior incidência de DIC do que homens.^29^
• **Estresse:** Saúde mental é tema de grande relevância, principalmente após a pandemia da COVID-19. Dados mostram que a relação entre estresse e RCV é bem estabelecida, onde ansiedade e depressão estão associadas ao aumento de morbimortalidade por DCV.^29,67^A ocorrência de violência doméstica, que atinge grande parte da população feminina, é fator desencadeante de estresse crônico importante, levando a sequelas que persistem mesmo após o término do abuso, ocasionando outros transtornos mentais.^29^
• **Hipertensão arterial sistêmica:** É o FR de maior importância para as mulheres. Hipertensas apresentam maior risco de IAM comparado a homens hipertensos devido à maior velocidade de progressão da doença, maior negligência no diagnóstico e menor aderência ao tratamento.^29,70^ Apesar de as diretrizes de HAS serem bem estabelecidas, recentemente tem-se discutido o impacto de se estabelecerem diferentes metas pressóricas por sexo.^29^

### 4.3. Fatores de Risco Estabelecidos e Não Modificáveis

• **Histórico familiar:** Mulheres com menos de 65 anos e história materna de IAM apresentam risco quatro vezes maior de SCA que homens da mesma idade ou mulheres mais velhas.^67^
• **Idade:** A idade propicia o desenvolvimento de síndrome metabólica, que conceitualmente associa os principais FR, ou seja, DM, HAS, obesidade e dislipidemia,^70^ e leva ao aumento da resistência insulínica e do estado pró-inflamatório e pró-trombótico, aumentando o risco de DCV.^29^

### 4.4. Fatores de Risco Específicos da Mulher

• **Menopausa:** O estrogênio é conhecido por seu efeito protetor do endotélio vascular. Na menopausa, a ausência da proteção estrogênica leva a efeitos negativos na função cardiovascular/metabolismo, tais como: alteração da gordura corporal, inflamação vascular, disfunção endotelial, aumento do tônus simpático e maior resistência à insulina.^67^ Entretanto, a terapia hormonal da menopausa não é indicada para prevenção primária ou secundária de DCV, sendo que seu uso se baseia na reposição em caso de menopausa prematura. Quando existem sintomas vasomotores acentuados, que comprometem a qualidade de vida, a terapia hormonal também pode ser usada, mas há necessidade de avaliação individual de forma interprofissional para segurança e eficácia do tratamento.^70,71^
• **Distúrbios relacionados à gravidez:** A mulher está sujeita a complicações como diabetes gestacional e doença hipertensiva da gravidez, o que aumenta em três a quatro vezes o risco de SCA ao longo de toda a sua vida.^70^ Sendo assim, sua história obstétrica deve ser investigada cuidadosamente e a presença dessas comorbidades dita o acompanhamento contínuo da mulher. Mulheres com pré-eclâmpsia têm um risco 3,7 vezes maior de desenvolver HAS 14 anos mais tarde, 2,16 vezes maior de DIC em 12 anos, 1,8 vez de AVC em 10 anos e 1,78 vez de tromboembolismo venoso em 5 anos. O diabetes gestacional aumenta o risco de DM tipo 2 em sete vezes, o risco de AVC em duas vezes e de infarto do miocárdio em quatro vezes, independentemente de a paciente se tornar ou não diabética.^29,71^
• **Anticoncepcionais hormonais orais:** O uso de anticoncepcionais hormonais orais dobra o risco de SCA de etiologia aterotrombótica, sendo potencializado pelo tabagismo, HAS e DM.^29^
• **Síndrome do ovário policístico:** Esta, por promover excesso de andrógenos e oligoanovulação, leva a maior probabilidade de desenvolver dislipidemia, HAS e DM.^29^
• **Distúrbios inflamatórios sistêmicos e autoimunes:** A inflamação crônica de patologias como lúpus eritematoso sistêmico e artrite reumatoide leva a disfunção endotelial e maior predisposição a DCV. As mulheres têm mais prevalência de tais distúrbios em relação aos homens. A razão entre mulheres e homens para artrite reumatoide é de 2,5:1 e para lúpus eritematoso sistêmico é de 9:1. O risco de IAM é duas a três vezes maior e de AVC, 50% maior em mulheres com artrite reumatoide. Para lúpus eritematoso sistêmico, o risco de IAM é de 9-50 vezes o da população geral. Os escores de risco habituais subestimam o RCV nas mulheres com artrite reumatoide e lúpus eritematoso sistêmico, sendo sugerido multiplicar por 1,5 o risco absoluto.^29,71^
• **Radiação e Quimioterapia para Tratamento do Câncer de Mama:** Radioterapia para tratamento do câncer de mama envolve exposição do coração à radiação ionizante, aumentando o risco de DIC. Esse risco é proporcional à dose, começa poucos anos após a exposição e continua por 20 anos. Existe associação com a presença de FR e é pior na irradiação da mama esquerda comparada à da direita. Pode se manifestar por valvopatias ou cardiomiopatias.^29,69^

Pelo menos 20% das mulheres apresentam evento coronariano sem FR para DCV bem estabelecidos.^69^ Importante avaliá-las de forma singular na identificação e controle dos FR, tanto os estabelecidos como os específicos, cruciais no acompanhamento longitudinal da mulher para prevenção de DCV e promoção da saúde.^29^

### 4.5. Fatores de Risco Sub-reconhecidos

• Alguns desses fatores, como os distúrbios de ansiedade/depressão e determinantes sociais de saúde, como abuso sexual e violência, privação socioeconômica e baixa escolaridade, são considerados potencializadores de DCV, sendo importante sua investigação na estratificação das mulheres. Ademais, os determinantes sociais de saúde também levam a desfechos negativos, como a inflamação vascular e a disfunção endotelial, principalmente quando associados aos FR clássicos.^32^
• Os distúrbios de ansiedade/depressão aumentam em duas vezes o risco de DIC, por alterarem o eixo hipotálamo-hipofisário-adrenal e SNA, aumentando o estresse oxidativo e a resposta inflamatória, com consequente disfunção endotelial, aterotrombose e DIC. Mulheres jovens têm maior risco de depressão e maior mortalidade após IAM do que homens, além de apresentarem menor declínio nas taxas de morte do que homens.^29^

#### 4.5.1. Recomendações

As recomendações específicas dos fatores de risco cardiovasculares estão descritas no Posicionamento sobre Saúde Cardiovascular da Mulher.^32^

## 4.6. Tratamento Medicamentoso nas Diferentes Formas de Manifestação Isquêmica

Comparadas aos homens, as mulheres, incluindo as mais jovens, têm maior atraso no diagnóstico e tratamento da SCA. Por conseguinte, são menos submetidas a coronariografia e tratamento cirúrgico,^72^ incluindo suporte circulatório mecânico no choque cardiogênico. Apresentam, portanto, maior probabilidade de óbito intra-hospitalar.

Parece que o menor índice de tratamento cirúrgico nas mulheres se deve à presença de MINOCA, artérias menos calibrosas e doença aterosclerótica menos grave. As mulheres apresentam maior prevalência de outros mecanismos fisiopatológicos da DIC, como cardiomiopatia de Takotsubo (estresse-induzida), disfunção microvascular coronariana, embolia coronariana, vasoespasmo coronariano e DEAC. Porém, esse dado não explica porque as mulheres são menos submetidas a coronariografia e a suporte circulatório no choque cardiogênico.^72^

A INOCA predomina em mulheres (> 50% *versus* 7-17% em homens) e associa-se a risco de desfechos desfavoráveis, como hospitalizações recorrentes, intervenções, pior qualidade de vida, maior mortalidade e cinco a dez vezes mais risco de desenvolver ICFEp. Portanto, seus diagnóstico e prognóstico, assim como a conduta nos casos de INOCA, continuam sendo um desafio para os médicos.^73^ Apesar de a INOCA se associar a risco de eventos maiores, menos de 50% das pacientes são submetidas a tratamento medicamentoso adequado, além de ser baixa a aderência ao tratamento prescrito e haver subutilização de reabilitação cardíaca.^74^ Sugere-se que as mulheres são menos submetidas à terapia medicamentosa por não haver evidências sobre seu tratamento, sobretudo quando existe disfunção microvascular coronariana associada.^75,76^ Outro fator que limita o consenso sobre seu tratamento é a presença de diferentes fenótipos na doença, ora predominando disfunção microvascular coronariana, ora vasoespasmo coronariano ou ambos. Portanto, as respostas terapêuticas são variadas e incertas.

O tratamento da INOCA/MINOCA baseia-se em três alicerces, a saber:^77^

1. Mudança de estilo de vida
2. Controle dos FR
3. Tratamento antianginoso

Mudança de estilo de vida, controle dos FRCV, como HAS, DM e dislipidemia, e reabilitação cardiovascular são medidas fundamentais na redução de morbimortalidade na INOCA e MINOCA,^73^ assim como na doença coronariana.

Estudos randomizados, como WISE e WARRIOR (em andamento), têm demonstrado a eficácia do inibidor da enzima conversora da angiotensina (IECA) na RFC e na melhora da angina. Portanto, pacientes hipertensas portadoras de INOCA/MINOCA podem se beneficiar com seu uso.^73^

As estatinas, por seu efeito inibitório sobre o estresse oxidativo e propriedades anti-inflamatórias, atuam na RFC e na disfunção microvascular coronariana, melhorando a tolerância ao esforço e reduzindo isquemia esforço-induzida, principalmente quando associada a diltiazem.^78^

Drogas antianginosas, como os nitratos, betabloqueadores (BB) e bloqueadores dos canais de cálcio (BCC), são utilizadas na INOCA e MINOCA, porém sua eficácia varia de acordo com o fenótipo predominante, uma vez que podem predominar disfunção microvascular coronariana, vasoespasmo coronariano ou ambos.^77^ Em pacientes com disfunção microvascular coronariana, os nitratos de longa ação podem piorar os sintomas, por possível efeito de roubo de fluxo, devendo-se dar preferência aos BCC. Nitratos de curta duração têm eficácia incerta e precisam ser usados repetidas vezes.

Os BCC são drogas de primeira escolha em caso de vasoespasmo coronariano, seguido dos nitratos. Em casos de disfunção microvascular coronariana, RFC reduzida e/ou resistência microcirculatória aumentada, sugerindo remodelamento arteriolar, são usados inicialmente BB e, em casos refratários, os BCC. Drogas como a ranolazina e a trimetazidina podem ser consideradas no tratamento da DIC não obstrutiva, principalmente no caso da angina refratária.

O BB mais utilizado é o nebivolol por sua ação B1 seletiva e efeito vasodilatador via produção de óxido nítrico. Ele tem sido estudado em mulheres com disfunção microvascular coronariana, melhorando a angina e a tolerância ao esforço físico. Seu uso intracoronário melhora a RFC e reduz o consumo de oxigênio (VO_2_) miocárdico. Além disso, melhora a pressão de enchimento do VE e RFC em pacientes com HAS não complicada, possivelmente pela ação do óxido nítrico na microcirculação.^78^

Drogas promissoras foram estudadas na INOCA, como o nicorandil e a trimetazidina. O primeiro é um ativador dos canais de potássio, levando a vasodilatação da microcirculação semelhante aos nitratos. A trimetazidina, droga que atua no metabolismo das células musculares cardíacas inibindo a oxidação dos ácidos graxos livres, leva à maior utilização da glicose na produção de fosfatos de alta energia. A trimetazidina em monoterapia ou em combinação a drogas antianginosas melhorou a tolerância ao esforço na angina estável e em pacientes com INOCA. É também descrita melhora na perfusão miocárdica e função endotelial, provavelmente devido à redução do estresse oxidativo. Uma dose única de trimetazidina aumenta a duração do exercício e melhora os sinais e sintomas de isquemia no ECG de esforço. Estudos recentes mostram evidência de cardioproteção metabólica na angina estável e SCA em pacientes após angioplastia percutânea com a trimetazidina.^73,78^

A ranolazina atua na inibição dos canais tardios de cálcio reduzindo sua concentração intracelular nos cardiomiócitos, melhorando assim a função de relaxamento e microcirculação. Parece ter uma ação favorável em mulheres com INOCA e pacientes com RFC reduzida.^73,78^

Como as mulheres apresentam o estresse mental ou emocional como deflagrador de dor isquêmica, maior suscetibilidade psicológica e maior reatividade do SNA, baixas doses de antidepressivos tricíclicos podem ajudar na redução dos sintomas.^77^

Concluindo, as mulheres devem ser abordadas com olhar diferenciado do ponto de vista cardiovascular, atentando-se para as peculiaridades das manifestações clínicas da DIC por diferentes mecanismos fisiopatológicos, o que dificulta o diagnóstico e, consequentemente, a abordagem terapêutica. Sendo assim, esses fatores podem trazer consequências muitas vezes deletérias à sua saúde. A análise dos FR deve ser ampla, buscando-se fatores potencializadores de risco, além dos FR específicos das mulheres e os sub-reconhecidos.

### 4.6.1. Recomendações

O tratamento da INOCA/MINOCA baseia-se na mudança de estilo de vida, controle dos FR e tratamento antianginoso. Nas hipertensas deve-se dar preferência aos IECA/bloqueador do receptor de angiotensina (BRA). As estatinas atuam na RFC e na disfunção microvascular, melhorando a tolerância ao esforço e reduzindo isquemia esforço-induzida. Como drogas antianginosas, podem-se usar nitratos, BB e BCC; porém, em pacientes com disfunção microvascular coronariana, os nitratos de longa ação podem piorar os sintomas, por possível efeito de roubo de fluxo, devendo-se dar preferência aos BCC. Os BCC são drogas de primeira escolha no vasoespasmo coronariano. Em casos de remodelamento arteriolar (disfunção microvascular coronariana, RFC reduzida e/ou resistência microcirculatória aumentada), usar inicialmente BB (o mais utilizado é o Nebivolol – ação B1 seletiva e vasodilatação via produção de óxido nítrico) e, em casos refratários, os BCC. Ranolazina e trimetazidina podem ser consideradas na angina refratária.^78^

Baixas doses de antidepressivos tricíclicos podem ajudar na redução dos sintomas em mulheres com estresse mental ou emocional como deflagrador de dor isquêmica.^77^

## 5. Diagnóstico por Avaliação Funcional Gráfica

### 5.1. Eletrocardiograma de Repouso

O ECG das mulheres apresenta diferenças quando comparado ao dos homens, como FC em repouso mais elevada, menor amplitude e maior duração do QRS, maiores intervalos QT e desvios do segmento ST.^79,80^

Para avaliação da DIC, a posição correta dos eletrodos é fundamental para evitar diagnóstico equivocado. Mamas volumosas ou próteses mamárias geram complexos de baixa voltagem e reduzem a amplitude da onda R nas derivações V1 e V2, simulando área inativa. Em contrapartida, mulheres mastectomizadas geram QRS maiores, simulando hipertrofia ventricular esquerda.^80^

O ECG na avaliação de dor torácica em mulheres mantém os critérios diagnósticos descritos para o sexo masculino, exceto para lesão subepicárdica. Nas mulheres, são considerados patológicos valores de elevação do ponto J e segmento ST ≥ 1,5 mm nas derivações precordiais V1 a V3.^80^ Outras variáveis, como maior ângulo QRS-T e maior duração do QRS, podem ser consideradas preditores independentes de morte, IC e IAM não fatal.^81^As anormalidades da repolarização ventricular na menopausa podem ser preditoras importantes de eventos coronarianos e mortalidade por DIC.^82^

### 5.2. Teste Ergométrico

O TE é um exame funcional acessível, seguro, reprodutível, de baixo custo, sem radiação, capaz de analisar a resposta cardiovascular ao esforço, propiciando informações clínicas, hemodinâmicas, metabólicas, autonômicas e eletrocardiográficas que auxiliam no diagnóstico, estratificação de risco, avaliação terapêutica e prescrição de exercícios físicos.^83^

A visão multifatorial do TE ampliou sua interpretação ao incluir variáveis prognósticas, sobretudo nas mulheres. É recomendado como método inicial de escolha na avaliação de mulheres sintomáticas de risco intermediário para DIC, com ECG de repouso normal e capazes de se exercitar.^79,84^

Em meta-análise de 19 estudos, a sensibilidade média do TE foi menor nas mulheres em comparação aos homens (61% *versus* 70%), assim como a especificidade (72% *versus* 77%).^1^ Em mulheres sintomáticas, o valor preditivo positivo da depressão do segmento ST é menor do que nos homens (47% *versus* 77%, p < 0,05); no entanto, o valor preditivo negativo é similar (78% *versus* 81%). Um TE máximo e negativo é útil para afastar a presença de DIC obstrutiva significativa e prever excelente sobrevida livre de eventos.^79^

Nas mulheres, existe maior prevalência de alterações eletrocardiográficas sem correspondência de DIC obstrutiva.^83^ Os fatores que mais contribuem para menor acurácia diagnóstica incluem: menor prevalência de DIC multiarterial; maior prevalência de DIC não obstrutiva, prolapso valvar mitral, angina vasoespástica e microvascular; ECG com baixa voltagem e alterações da repolarização ventricular; sintomas predominantemente atípicos; fatores hormonais na pré- e pós-menopausa; menores tolerância ao esforço e FC atingida.^79,83^

Alterações do segmento ST com morfologia ascendente lenta ou convexa, principalmente restritas às derivações inferiores, que surgem em FC alta, maior carga de trabalho e rápida normalização na recuperação (dentro do 1º minuto) tendem a não se correlacionar com doença arterial coronariana obstrutiva significativa, ao contrário das alterações ≥ 2,0mm, morfologia horizontal ou descendente, em baixas carga e FC, persistentes por vários minutos na recuperação, sobretudo se acompanhadas de sintomatologia.^85^ Além das alterações do segmento ST, o TE contribui com importantes informações prognósticas por meio da análise de parâmetros, como capacidade funcional, respostas cronotrópica e da PA, recuperação da FC e avaliação de escores.

A capacidade funcional é o maior preditor independente de morte em mulheres, incluindo as assintomáticas.^86^A capacidade de atingir ≥ 10 MET é indicador de prevalência muito baixa de isquemia miocárdica significativa, independentemente do gênero.^87^Entretanto, quando < 7 MET são alcançados, a probabilidade de isquemia é significativamente maior (0,4% *versus* 7,1%).^88^ A incapacidade de atingir 5 MET é preditora independente de alto risco, com aumento de três vezes na mortalidade em comparação às mulheres que atingiram > 8 MET.^89^

A incapacidade de alcançar 85% da FC máxima prevista para a idade é preditiva de risco aumentado de mortalidade e DIC obstrutiva em mulheres.^79^ A presença de incompetência cronotrópica aumentou em 30% o risco de mortalidade por todas as causas.^89^ A diminuição da FC no primeiro minuto da recuperação < 12 bpm é outro importante marcador prognóstico, sendo preditor independente de mortalidade por todas as causas.^79^

O incremento da PA é menor, principalmente nas mulheres mais jovens, e a não elevação (“platô”) é achado frequente, em geral não relacionado à doença cardíaca, diferentemente do sexo masculino. Sua interpretação deve ser associada às demais variáveis.^89^

Os escores podem ser úteis para aperfeiçoar o diagnóstico de DIC, sendo o Escore de Duke o mais utilizado, com valor diagnóstico e prognóstico, e calculado pelo tempo de exercício (protocolo Bruce) – 5x (desnível do segmento ST) – 4x (angina, sendo: 0 = angina ausente, 1 = dor não limitante, 2 = dor limitante).^90^As categorias são: baixo risco (ED ≥ 5), risco moderado (ED de −10 a 4) e alto risco (ED ≤ −11).^91^ Quando baixo, o ED está associado a taxa de mortalidade anual de ≈0,25%, em contraste com ≈ 5% no alto risco, sendo as taxas de mortalidade mais baixas em mulheres. Em coorte de 976 mulheres sintomáticas com TE e angiografia, estenose coronariana ≥ 75% esteve presente em 19%, 35% e 89% daquelas com ED de baixo, moderado e alto risco, respectivamente.^92^ Mulheres com ED intermediário devem ser encaminhadas para estratificação de risco adicional com imagem sob estresse.^79^ Essa é uma ferramenta valiosa para prever o risco de eventos (morte cardíaca, IAM não fatal e revascularização tardia) entre as mulheres, mas pode ser menos eficaz naquelas ≥ 75 anos.^93^

A Tabela 5.1 apresenta os indicadores de alto risco no TE em mulheres.

**Tabela 5.1:** – Variáveis indicadoras de alto risco que merecem ser valorizadas e relatadas no teste ergométrico em mulheres.

Variável	Indicativos de alto risco
Capacidade de exercício	< 5 METs
Alterações do segmento ST	Infradesnivelamento ≥ 2mm Infradesnivelamento ≥ 1mm com < 5 MET ou > 5 min da recuperação. Supradesnivelamento ≥ 2mm (em derivações sem ondas Q ou aVR)
Queda da FC no 1º min da recuperação	≤ 12 bpm
Escore Duke	≤ -11
Resposta da PA	Diminuição > 10mmHg em relação ao repouso
Arritmias ventriculares	TV persistente/fibrilação

*Adaptado de Mieres et al.*^79^ *FC: frequência cardíaca; PA: pressão arterial; TV: taquicardia ventricular.*

Em situações com maior probabilidade de DCV, doenças ortopédicas, obesidade e sedentarismo, a associação com cintilografia de perfusão miocárdica (CPM) pode ser útil quando comparada com resultados de testes não conclusivos.

#### 5.2.1. Recomendações

A avaliação individualizada e as terapias anti-isquêmicas direcionadas após o TE devem ter por base a carga alcançada, os sintomas e o grau de anormalidade do exame.

A escolha do método diagnóstico deve ser baseada no perfil clínico da paciente, na probabilidade pré-teste de DCV, nos sintomas, no ECG de repouso e na presença/ausência de condições adequadas para realização de um TE com boa qualidade técnica. A análise deve ser multifatorial para otimizar a abordagem clínica e terapêutica.

## 5.3. Teste Cardiopulmonar de Exercício

O TCPE permite avaliação não invasiva dos sistemas cardiovascular, respiratório e periférico de maneira integrada ao esforço. Embora esteja bem estabelecido na avaliação clínica de pacientes com IC, sua indicação não está bem consolidada pelas diretrizes na investigação da DIC. Em artigo de revisão sobre aplicabilidade do TCPE na DIC, estabeleceram-se as seguintes indicações:^94^

1. Moderada a alta probabilidade pré-teste para DIC
2. Acréscimo na acurácia diagnóstica da DIC, quando alguma alteração clínica ou eletrocardiográfica estiver presente, dificultando o diagnóstico pelo TE
3. Área isquêmica miocárdica de grande magnitude, comprometendo a função ventricular esquerda
4. Avaliação evolutiva após RVM mecânica ou cirúrgica.

Além da avaliação dos critérios clínicos, hemodinâmicos e eletrocardiográficos, o TCPE gera informações adicionais dos parâmetros ventilatórios na análise da isquemia, quantifica a capacidade funcional com precisão e estratifica o prognóstico. A baixa capacidade funcional [VO_2_ < 60% do predito para idade e sexo] e sinais e sintomas de isquemia miocárdica em baixa carga (VO_2_ < 15 ml.kg^-1^.min^-1^) estratificam os pacientes como de alto risco para eventos cardiovasculares.^95^

Em pacientes com DIC macrovascular e defeitos de perfusão reversíveis na imagem de perfusão miocárdica, a avaliação das variáveis do TCPE aumentou, em comparação ao TE, a sensibilidade de 46% para 87% e a especificidade de 66% para 74%.^96^

O TCPE identifica o início da disfunção ventricular esquerda induzida por isquemia durante o esforço e, pode assim, melhorar a acurácia diagnóstica, principalmente no sexo feminino, reduzindo a taxa de TE falso positivo.^97^ Isso é particularmente importante, pois, na cascata isquêmica miocárdica, as alterações das funções diastólica e sistólica do VE ocorrem antes das alterações isquêmicas ao ECG.^98^

As principais variáveis obtidas durante o TCPE que retratam a disfunção do VE desencadeada pela DIC são: o tipo de curva e a ascensão do VO_2_, pulso de oxigênio (PO_2_) e taxa de trabalho (variação VO_2_/variação carga em *Watt*), essa última exclusivamente em cicloergômetro. Espera-se aumento linear no VO_2_ paralelamente à carga de trabalho de cerca de 10 ml/min/*watt* sob condições fisiológicas.^94,99^

**Figura 5.1:**
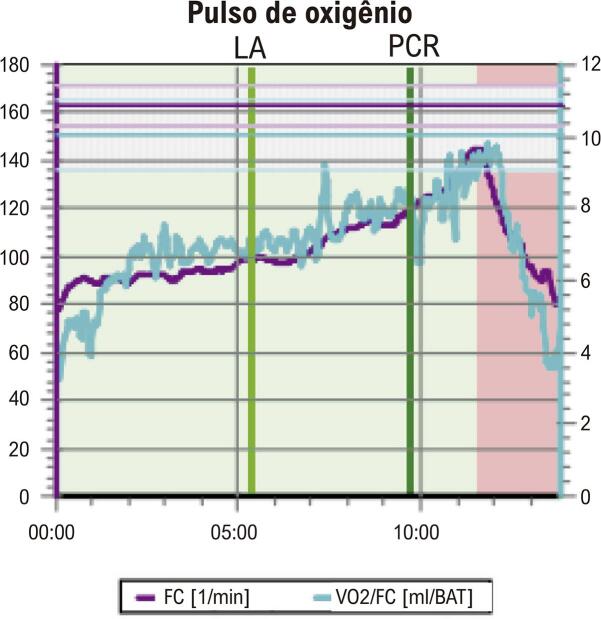
– Comportamento ascendente da curva do pulso de oxigênio em função do tempo. FC: frequência cardíaca; LA: limiar anaeróbico; PCR: ponto de compensação respiratória.

**Figura 5.2:**
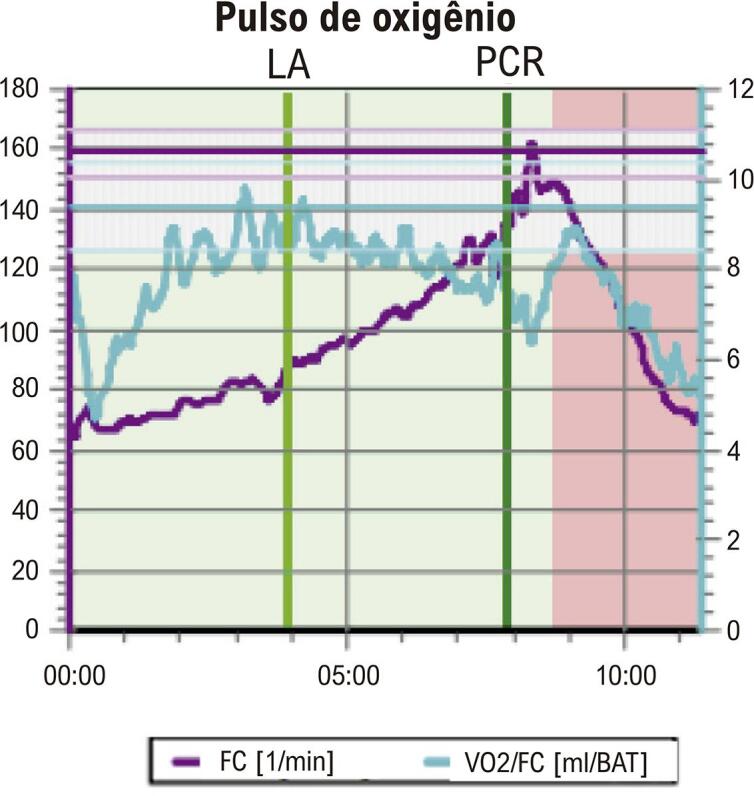
– Comportamento descendente da curva do pulso de oxigênio em função do tempo. FC: frequência cardíaca; LA: limiar anaeróbico; PCR: ponto de compensação respiratória.

A análise do PO_2_ melhora a acurácia do diagnóstico da DIC. Avalia-se tanto o valor atingido no pico do esforço em relação ao predito quanto o formato da curva em função do tempo, a qual deve apresentar uma tendência crescente, mais comumente em formato de parábola (Figura 5.1).^94,99^ Na presença de DIC obstrutiva, o volume sistólico reduzido provoca aumento compensatório da FC, que impacta tanto a taxa de trabalho cardíaco como o PO_2_. Notam-se redução da progressão da curva de VO_2_/carga e queda ou ausência de aumento progressivo do PO_2_ (Figuras 5.2 e 5.3). Na presença de platô precoce ou, principalmente, declínio do PO_2_ durante o esforço, a prescrição da intensidade do exercício pode ser limitada às cargas abaixo desse limiar.^99,100^

**Figura 5.3:**
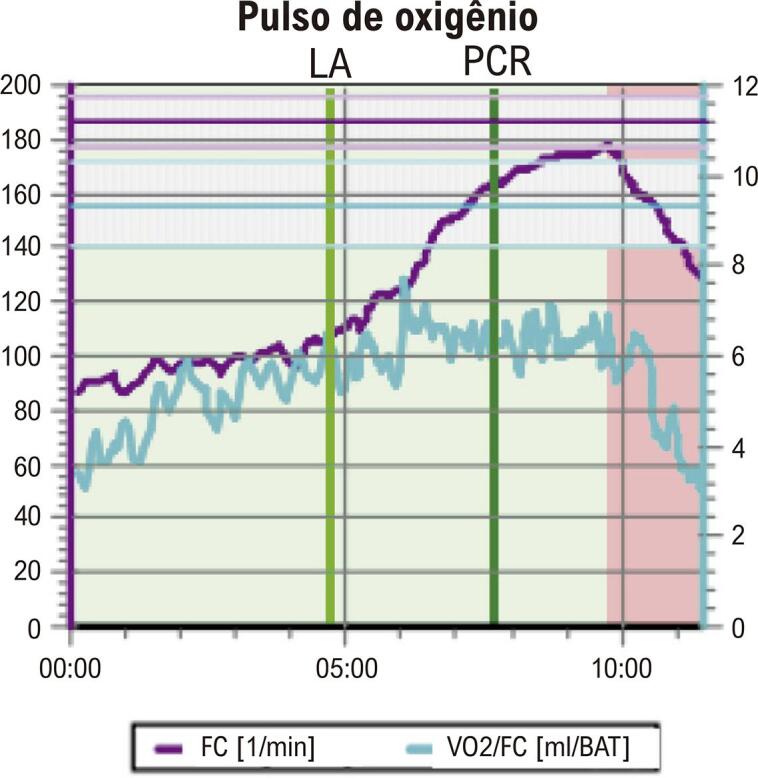
– Comportamento em platô da curva do curva do pulso de oxigênio em função do tempo. FC: frequência cardíaca; LA: limiar anaeróbico; PCR: ponto de compensação respiratória.

O subestudo do ORBITA-Trial analisou os parâmetros do TCPE em relação a isquemia miocárdica e sintomas anginosos nos pacientes com síndrome coronariana crônica. O platô do PO_2_ foi a única variável capaz de detectar a gravidade da isquemia e predizer a eficácia da ICP na DIC grave de único vaso.^101^

Trabalhos recentes avaliaram o papel do TCPE na disfunção microvascular coronariana, particularmente em mulheres, onde o TE tem menor acurácia. Estudo em mulheres na pós-menopausa, com angina típica e sem doença arterial coronariana obstrutiva à angiografia, demonstrou que a isquemia à cintilografia estava associada a disfunção sistólica do VE após o estresse, sugerindo DIC microvascular. Dado o potencial do TCPE para inferir disfunção do VE de forma não invasiva e custo-eficaz, a implementação desse exame na investigação da DIC microvascular aumenta o potencial diagnóstico em relação ao TE.^102,103^ O TCPE pode ser também uma ferramenta importante na avaliação de pacientes com bloqueio de ramo esquerdo (BRE) em virtude da limitação do TE nessa população. Curvas normais de VO_2_ e PO_2_ poderiam descartar estenose coronariana significativa como causa do BRE.^99^

É importante conhecer algumas limitações do TCPE, como restrição da avaliação da taxa de trabalho somente em cicloergômetro, menor sensibilidade na doença arterial coronariana uniarterial, condições clínicas associadas que podem interferir no VO_2_ (anemia, doença pulmonar, IC).^99^ O TCPE apresenta-se como uma ferramenta valiosa na prática clínica diária, mas futuras investigações devem ser direcionadas para avaliação mais precisa do seu papel no diagnóstico, prognóstico e avaliação da eficácia do tratamento da DIC, sobretudo, nas mulheres.

## 6. Diagnóstico por Imagem Cardiovascular Não Invasiva

### 6.1. Introdução

**Figura 6.1:**
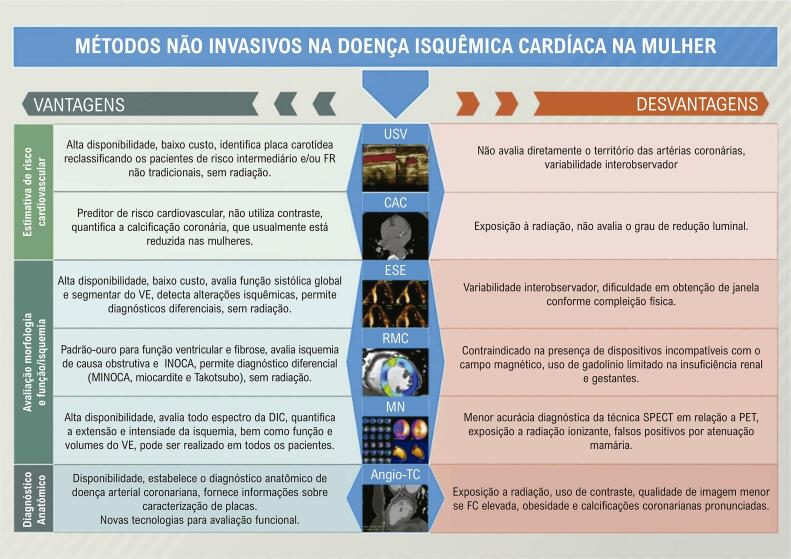
– Importância dos métodos de imagem não invasivos na doença isquêmica cardíaca na mulher. Angio-TC: angiotomografia computadorizada de coronárias; CAC: escore de cálcio; DIC: doença isquêmica cardíaca; ESE: ecocardiograma sob estresse; FC: frequência cardíaca; FR: fator de risco; INOCA: isquemia na ausência de obstrução arterial coronariana; MINOCA: infarto do miocárdio na ausência de obstrução arterial coronária; MN: medicina nuclear; PET: tomografia por emissão de pósitrons; RMC: ressonância magnética cardíaca; SPECT: tomografia computadorizada por emissão de fóton único; USV: ultrassom vascular (carótidas); VE: ventrículo esquerdo.

Os métodos de imagem não invasivos desempenham papel fundamental no diagnóstico e manejo da doença arterial coronariana. Nas mulheres, especialmente, existem particularidades quanto à etiologia da isquemia miocárdica envolvendo a DIC obstrutiva e a disfunção microvascular coronariana.^104^ Neste capítulo, as diversas modalidades de imagem cardiovascular serão abordadas, com informações importantes quanto à avaliação dos sintomas de isquemia miocárdica nas mulheres, definindo desempenho diagnóstico, peculiaridades relacionadas ao sexo feminino, assim como vantagens e limitações de cada método (Figura 6.1). Os valores de sensibilidade e especificidade dos principais métodos diagnósticos para mulheres e homens, de acordo com uma revisão recentemente publicada, estão demonstrados na Tabela 6.1.^8^

**Tabela 6.1:** – Sensibilidade e especificidade de acordo com o sexo, para os principais métodos diagnósticos.

Modalidade	TE	ESE	SPECT-CPM	PET-CPM	Angio-TC	RMC
Sensibilidade						
Mulheres	43-71%	70-96%	84-91%	81-100%	90-98%	84-91%
Homens	68-80 %	70-80%	85-94%	81-92%	90-97%	90-91%
Especificidade						
Mulheres	64-85%	79-92%	58-91%	86%	84%	81-88%
Homens	74-83%	84-93%	62-89%	85-89%	83%	74-94%

*Angio-TC: angiotomografia de artérias coronárias; CPM: cintilografia de perfusão miocárdica; ESE: ecocardiografia sob estresse; PET: tomografia por emissão de pósitrons; RMC: ressonância magnética cardíaca; SPECT: tomografia computadorizada por emissão de fóton único; TE: teste ergométrico.*

### 6.2. Ecocardiograma de Repouso e sob Estresse

O ecocardiograma transtorácico (ETT) de repouso ou com o emprego das diversas modalidades de estresse (seja exercício, administração de inotrópico ou vasodilatador) tem grande utilidade na detecção de isquemia miocárdica nas mulheres.^104^ A técnica é particularmente atraente pela ausência de radiação em tecido mamário, em especial nas mais jovens, considerando a expectativa de exposição cumulativa ao longo da vida, quando estudos seriados podem ser necessários. Soma-se a isso a boa acurácia do método.^104,105^

As alterações encontradas compatíveis com isquemia miocárdica incluem: anormalidades da contração segmentar das paredes do VE em repouso, resposta contrátil alterada durante o estresse, perfusão microvascular reduzida avaliada com emprego de contraste ecocardiográfico e/ou comprometimento do fluxo das artérias epicárdicas analisado através do Doppler.^79,106^ Além disso, o ETT é útil na identificação de outras causas de dor torácica, como pericardite, dissecção da aorta e embolia pulmonar.^59,79^

Recomenda-se o ETT em repouso para todas as pacientes com suspeita de DIC visando avaliar a contração segmentar, assim como a função sistólica global do VE, através da fração de ejeção do VE (FEVE).^59,106^ A presença de anormalidades contráteis regionais pode ser crônica ou aguda, questão melhor resolvida se estudos anteriores estiverem disponíveis para a comparação.^106^ É importante ressaltar que os segmentos com alteração por DIC devem ser contíguos e corresponder aos territórios das artérias coronárias, pois alterações segmentares podem ser causadas por uma variedade de outras condições não relacionadas à DIC.^106^

A contração segmentar do VE é avaliada visualmente pelo espessamento das paredes durante a sístole. Métodos quantitativos mais recentes vêm sendo aplicados, como a análise da deformação (*strain*) miocárdica longitudinal global do VE (SLGVE). As fibras musculares subendocárdicas, orientadas longitudinalmente, são mais vulneráveis à isquemia, sendo que a avaliação da SLGVE em repouso demonstrou ser mais sensível para a detecção de anormalidades segmentares quando comparada à análise da contratilidade da parede na SCA.^106^

A modalidade preferencial para a avaliação de isquemia utilizando-se a ESE é o esforço físico, especialmente a bicicleta supina. Além de fornecer dados adicionais prognósticos, como a capacidade funcional, esse método trabalha com dados mais fisiológicos na pesquisa da isquemia.^79^ Quando comparada ao TE, a ESE apresenta melhor sensibilidade e especificidade em mulheres.^59,79^ Uma meta-análise de 14 estudos, que avaliou a sensibilidade e a especificidade da ESE com estresse físico para detecção de DIC em mulheres, reportou valores de 79% (IC 95%, 74%–83%) e 83% (IC 95%, 74%–89%), respectivamente.^107^

A ESE com estresse farmacológico utilizando dobutamina é indicada, preferencialmente, para pacientes que não conseguem se exercitar.^79^ Os vasodilatadores, como dipiridamol ou adenosina, apresentam sensibilidade discretamente menor nas mulheres.^108^ Em uma comparação entre a ESE com dobutamina e o TE para a detecção de DIC em mulheres sintomáticas, a primeira demonstrou maior acurácia em mulheres com dor no peito, com sensibilidade de 70,4% *versus* 53,7% e especificidade de 94,6% *versus* 73,6% para detecção de estenose coronariana > 50%. A maior acurácia da ESE foi mantida após a exclusão das pacientes que conseguiram atingir FC maior do que 85% da prevista para a idade antes da indução da isquemia.^109^ Os resultados são divergentes quando se compara o desempenho diagnóstico da ESE entre homens e mulheres.^8^ A literatura mostra que o desempenho nas mulheres é semelhante ou inferior ao nos homens.^11,109^ Quando comparada à SPECT na pesquisa de isquemia miocárdica, a ESE tem maior capacidade de especificidade, uma vez que não apresenta falsos positivos por atenuação mamária.^8^

A ESE pode ser limitada por impossibilidade na obtenção de janelas acústicas adequadas no pico de estresse,^104^ particularmente em mulheres, em virtude do tecido mamário. O uso de agentes de realce ecocardiográfico tornou-se uma ferramenta importante para a visualização adequada do endocárdio de todos os segmentos do VE. Assim, na presença de dois ou mais segmentos contíguos com limitada qualidade técnica, indica-se o emprego desses agentes.^110^

Na gestação, a ESE com esforço físico ou sob estresse farmacológico é considerada segura e evita exposição à radiação; dobutamina e dipiridamol são considerados categoria B.^1^

### 6.3. Ultrassonografia Vascular

A aterotrombose é uma doença arterial sistêmica que envolve principalmente a íntima das artérias de grande e médio calibre (carótidas, aorta, coronárias e artérias periféricas). A USV é um excelente método para avaliação morfológica e classificação da gravidade da doença obstrutiva carotídea, aneurisma de aorta abdominal e DAP, sendo importante ferramenta no diagnóstico da aterotrombose em outros territórios arteriais acometidos simultaneamente ao território coronariano e particularmente atraente pelo baixo custo, alta disponibilidade e caráter não invasivo.^111^

A detecção de doença subclínica e as informações sobre as características da placa e da carga aterosclerótica mostram-se úteis em alguns cenários peculiares da doença aterosclerótica da mulher. A presença ou ausência de placa pode reestratificar pacientes além dos FR tradicionais e a quantificação da carga aterosclerótica pode personalizar ainda mais essa avaliação de risco.^32,112-114^ A identificação da placa e a medida da carga aterosclerótica em território carotídeo e/ou arterial periférico podem ser úteis para reclassificação do risco, como demonstrado por estudos recentes.^115^ A análise das características e do volume da placa pelo 3D parece melhorar significativamente a estimativa da carga aterosclerótica e a identificação de placas mais vulneráveis, tendo papel potencial na estratificação de risco de mulheres, cuja carga aterosclerótica parece ser maior que a de homens.^114-116^ O uso dos agentes de realce de USV é também uma ferramenta atraente para pesquisar neovascularização de placas carotídeas, pois mulheres apresentam mais sinais de neovascularização.^117^

A DAP é prevalente tanto em homens quanto em mulheres, sendo sua forma subclínica mais comum em mulheres. Pacientes com passado de pré-eclâmpsia, hipertensão gestacional e insuficiência placentária tem três vezes mais chance de desenvolver DAP.^118^ Recentemente, sugeriu-se que a avaliação da aterosclerose carotídea e femoral melhore a detecção precoce da doença, sendo que a presença da placa femoral é mais indicativa de DIC do que a carotídea. Em mulheres, as áreas de placa femoral e carotídea combinadas têm maior sensibilidade, podendo auxiliar na identificação de testes de estresse falsos positivos.^119^

Mulheres têm menor prevalência de aneurisma de aorta abdominal e apresentam a doença em idade mais avançada. Entretanto, têm quatro vezes maior probabilidade de sofrer ruptura em comparação a homens da mesma idade e resultados piores após cirurgia de emergência. A sensibilidade do exame físico é de 29% no aneurisma de aorta abdominal com diâmetro de 3-3,9cm; 50% naquele com diâmetro de 4-4,9cm; e 75% naquele com diâmetro acima de 5 cm, justificando grande parte do diagnóstico ser incidental. Assim, em mulheres selecionadas, indica-se o rastreamento: idade entre 55 e 75 anos, história familiar de aneurisma de aorta abdominal, tabagistas pesadas e/ou histórico de AVC.^120^

### 6.4. Tomografia Computadorizada

Particularidades da DIC nas mulheres tornam a estratificação não invasiva uma etapa importante no manejo dessas pacientes. Os achados nos exames das mulheres diferem daqueles dos homens com relação a anatomia, distribuição e extensão das lesões, bem como repercussão clínica. Nesse cenário, a avaliação da calcificação coronariana e do grau de obstrução das lesões pela angio-TC torna-se importante para definição de tratamento e seguimento das pacientes.

Anatomicamente, observa-se que as artérias coronárias epicárdicas nas mulheres são menores quando comparadas às dos homens, mesmo com ajuste para idade, massa corporal e área de superfície corporal. Além disso, o tônus vasomotor dessas artérias também é menor, determinando maior fluxo coronariano, que, associado ao tamanho reduzido das artérias, potencialmente resulta nos diferentes fenótipos da DIC entre os sexos.^121^

Apesar das diferenças anatômicas, o potencial diagnóstico de DIC pela angio-TC é semelhante entre os sexos,^122^ tendo a angio-TC excelente acurácia na detecção de DIC obstrutiva e caracterização de placa quando comparada a métodos invasivos.^123^ Os valores de sensibilidade e especificidade são apresentados na Tabela 6.1.^8^

O padrão da apresentação da DIC em mulheres é predominantemente não obstrutivo e a carga aterosclerótica da DIC não obstrutiva (definida como estenose <50%) acomete de modo mais acentuado as mulheres,^124,125^ com risco elevado de eventos coronarianos em comparação com a população em geral, particularmente maior em mulheres < 75 anos.^126,127^ Análises do registro CONFIRM, que avaliou 24.775 pacientes, sendo 12.128 mulheres, demonstraram risco aumentado de mortalidade em pacientes com DIC obstrutiva e não obstrutiva. Em mulheres, notou-se ainda que o risco era mais acentuado para aquelas com maior extensão da DIC (acometimento de três vasos) (HR: 4,21; IC 95%, 2,47-7,18; p < 0,0001 *versus* HR: 3,27; IC 95%, 1,96-5,45; p < 0,0001) em comparação aos homens.^128^ A presença de placas ateroscleróticas com alto risco trombótico (baixa atenuação, calcificação puntiforme e sinal de *napkin ring)* é preditora de eventos cardíacos em mulheres.^129^ A calcificação coronariana avaliada pelo escore de cálcio (CAC) também difere entre os sexos. Para pacientes com mesma raça e idade, a quantificação do CAC é menor em mulheres. Os dados mostram consistentemente que a prevalência e a gravidade da calcificação coronariana aumentam com a idade e o sexo masculino, tendo as mulheres demonstrado menor prevalência e gravidade.^130^

Novas tecnologias incorporam a avaliação funcional à angio-TC, o que permite conciliar a avalição anatômica à pesquisa de isquemia. A FFR por tomografia computadorizada tem o potencial de elevar a acurácia diagnóstica na detecção de DIC obstrutiva quando comparada à angio-TC isoladamente. Apesar de promissora, a aplicabilidade clínica da FFR ainda é reservada a poucos centros que detêm a tecnologia.^131^

A DEAC é bem avaliada pela angio-TC, sendo uma complicação mais frequente em mulheres que em homens. Pacientes gestantes têm mortalidade elevada associada a DEAC. A indicação de angio-TC nessas pacientes deve ser individualizada, considerando-se o contexto clínico, mas, de modo geral, a estratégia invasiva pode ser necessária quando a suspeita clínica é muito relevante.^132^

### 6.5. Ressonância Magnética Cardíaca

A RMC vem contribuindo para uma melhor compreensão dos fenômenos fisiopatológicos, alterações estruturais, diagnóstico e avaliação prognóstica da DCV na mulher.^132^ A RMC é um método de alta resolução espacial que permite avaliação da morfologia, função, isquemia e caracterização tecidual com elevada acurácia. O fato de não apresentar limitação do campo de visão por interposição do tecido mamário, obesidade, doença pulmonar ou eventuais próteses mamárias, permite uma avaliação mais detalhada do coração feminino. Além disso, por ser um método que não utiliza radiação ionizante, coloca-se como uma opção inofensiva para o tecido mamário feminino, sendo uma alternativa interessante, sobretudo em mulheres mais jovens. O coração da mulher possui características morfológicas específicas, sendo relativamente menor que o masculino, com volume, massa e espessura parietal de menores dimensões, podendo ser avaliado com maior precisão pela RMC.^132-134^

A avaliação da perfusão miocárdica pela primeira passagem do gadolínio sob estresse farmacológico apresenta elevada sensibilidade e está bem estabelecida em ambos os sexos, desde que estudos, como MR-Impact, MR-Impact II e CE-MARC, demonstraram elevada sensibilidade na detecção de isquemia em comparação à SPECT, sendo recomendada sobretudo para a população feminina.^135-137^

Nas mulheres, a disfunção microvascular coronariana é um mecanismo responsável pela presença de INOCA^138^ e a RMC é útil na identificação de defeito perfusional subendocárdico sob estresse farmacológico nessa população.^132,139^ Além disso, a caracterização tecidual através do realce tardio e mapas T1 e T2 permite fornecer uma melhor avaliação das consequências dos eventos cardiovasculares sobre o miocárdio, mais especificamente da doença aterotrombótica. Desse modo, infarto do miocárdio, MINOCA, Takotsubo e miocardite por agressão viral ou autoimune podem ser bem definidos por essa metodologia.^132,138^

A técnica do realce tardio está estabelecida como a melhor forma de identificar a fibrose *in vivo* e pode diferenciar a injúria de etiologia isquêmica da não isquêmica, permitindo o diagnóstico diferencial entre infarto do miocárdico e quadros inflamatórios. A quantificação do envolvimento da espessura transmural do segmento por fibrose é um determinante na avaliação do grau de viabilidade miocárdica e na avaliação prognóstica.^138,140^

As contraindicações para uso da RMC estão restritas a situações incompatíveis com o campo magnético e devem ser consideradas as limitações ao uso do gadolínio em pacientes com insuficiência renal (*clearance* de creatinina abaixo de 30 ml/min/SC) pelo risco de fibrose sistêmica nefrogênica e em gestantes pelo risco de exposição para o feto ao agente paramagnético.^131^

A possibilidade de diferenciação diagnóstica dos eventos cardiovasculares causadores de sintomatologia anginosa e isquemia na mulher coloca a RMC como um método funcional preferencial nesse grupo de pacientes.^137^ O avanço das novas sequências de perfusão miocárdica, softwares de avaliação de perfusão quantitativa, técnicas de correção de movimento e maior disponibilidade dos mapas paramétricos são perspectivas que contribuem para a crescente utilização dessa metodologia. A RMC apresenta, portanto, um papel promissor único de avaliação do coração da mulher, agregando maior quantidade de informações diagnósticas e prognósticas.^132^

### 6.6. Medicina Nuclear

A imagem nuclear consegue avaliar todo o espectro da DIC, desde DIC epicárdica obstrutiva até disfunção microvascular coronariana.^141^ Diretrizes nacionais e internacionais enfatizam, na avaliação da DIC, o uso da CPM, que pode ser realizada em todos os pacientes, independentemente de função renal, presença de arritmias, obesidade ou dispositivos intracardíacos.^79,142^ As técnicas empregadas são SPECT e PET.

Há mais de três décadas a CPM pela técnica SPECT é usada extensivamente na prática clínica devido à sua ampla disponibilidade e extensa literatura que respalda seu valor no diagnóstico e na estratificação de risco da DIC.^79,142,143^É o estudo de imagem não invasivo mais comumente empregado na avaliação de mulheres com risco intermediário-alto de DIC e sintomas isquêmicos estáveis.^141^

Um mesmo exame traz informações sobre a extensão e a gravidade das anormalidades de perfusão, a motilidade das paredes miocárdicas, a FEVE e o sincronismo intraventricular em repouso e após estresse.^79,142,143^ O déficit perfusional total e a extensão dos defeitos de perfusão podem ser calculados, sendo parâmetros objetivos da carga isquêmica e do miocárdio em risco, que facilitam a definição de conduta e o acompanhamento terapêutico.^142^

O TE deve ser a técnica de escolha para a fase de estresse, quando a paciente é capaz de se exercitar adequadamente (atingir ≥ 5MET), por fornecer importantes informações diagnósticas e prognósticas adicionais. Nas pacientes incapazes de se exercitar adequadamente, devemos optar pelo estresse farmacológico com um vasodilatador (dipiridamol, adenosina ou regadenoson). Outra opção é o estresse com um fármaco inotrópico positivo, como a dobutamina, sensibilizado ou não com atropina. Mulheres com BRE e dispositivos intracardíacos devem ser sempre submetidas ao estresse farmacológico com um vasodilatador, independentemente da sua capacidade funcional, para se evitar artefatos na imagem.^79,142^

O valor prognóstico de uma CPM normal nas mulheres é excelente, incluindo mulheres idosas e de diversos subconjuntos raciais e étnicos, com 99% de sobrevida livre de eventos, sendo semelhante à dos homens, como demonstrado em uma grande meta-análise.^104^

A maior desvantagem da CPM é a exposição à radiação, embora tecnologias mais recentes, como novas gama-câmaras de detectores de estado sólido (CZT), melhorias nos radiofármacos, protocolos e doses de traçador individualizadas, tenham reduzido essa exposição.^143^ Falsos positivos podem ocorrer devido a artefato por atenuação mamária na parede anterior do VE, além de poder haver menor acurácia em mulheres com corações pequenos.

Embora menos disponível, a CPM pela PET tem excelente desempenho diagnóstico, com sensibilidade de 90-92% e especificidade de 81-88% para detectar estenoses angiograficamente significativas.^104^ Além da maior acurácia, a PET permite quantificar o fluxo sanguíneo miocárdico em mililitros por minuto por grama de tecido, além de calcular a RFC, um importante marcador prognóstico de RCV.^143^ Com isso, a PET melhora a detecção de DIC obstrutiva multiarterial grave com padrão de isquemia balanceada e de disfunção microvascular coronariana, que podem ser difíceis de identificar com SPECT devido às suas limitações técnicas.^142^ Outra vantagem da PET é a menor exposição à radiação (dose efetiva de 2-3mSv *versus* 9-12mSv com SPECT), por utilizar radiofármacos de curta duração, o que a torna particularmente útil em mulheres em idade reprodutiva.^79^ Estudos com PET mostraram que a disfunção microvascular coronariana é prevalente em mulheres e homens (54% e 51%, respectivamente) e, independentemente do sexo, a RFC mostrou ser um poderoso preditor incremental de eventos, tendência mantida mesmo em pacientes sem calcificações coronarianas.^104^As novas gama-câmaras CZT também têm mostrado resultados promissores na avaliação de RFC pela técnica SPECT, sendo uma promessa para maior disponibilidade da avaliação da disfunção microvascular coronariana em regiões onde a técnica PET não está disponível.^141,142^

A RFC anormal está também associada à disfunção diastólica. Um estudo recente com 64,7% de mulheres sem DIC obstrutiva mostrou uma associação independente de redução da RFC (definida como < 2) e disfunção diastólica, bem como aumento de eventos cardiovasculares ou ICFEp. Esses achados sugerem uma ligação fisiopatológica entre disfunção microvascular coronariana e ICFEp.^104,144^

Protocolos de imagem com equipamentos híbridos (PET/CT ou SPECT/CT) adicionam a capacidade de avaliar alterações anatômicas com quantificação do CAC, aumentando assim a sensibilidade do teste para o diagnóstico de DIC com um único exame.^104,142^

## 7. Arritmias na Cardiomiopatia Isquêmica

### 7.1. Fibrilação Atrial e Doença Isquêmica do Coração

A incidência ajustada para idade de FA é de uma vez e meia a duas vezes maior em homens, mas o risco de FA ao longo da vida é semelhante em ambos os sexos devido à maior expectativa de vida no sexo feminino; aos 85 anos, as diferenças na prevalência são discretas.^1,19-23^ As mulheres são mais sintomáticas e apresentam pior qualidade de vida quando comparadas aos homens. Os mecanismos associados às diferenças entres os sexos na FA são inúmeros, mas é importante ressaltar que a DIC, mais observada no sexo masculino, pode contribuir para a maior incidência de FA nesse grupo.

Quando se analisa a estratégia de controle do ritmo com o uso de DAA, as mulheres apresentam maior ocorrência de eventos adversos. O aumento no intervalo QT basal pode afetar a tolerância ao uso de DAA, especialmente da classe III, exigindo uma monitorização mais cuidadosa nesse grupo de pacientes. Em relação aos resultados da ablação por cateter no sexo feminino, estudos observacionais demonstram que as mulheres são submetidas menos frequente e mais tardiamente a ablação, em geral com evolução pior após o procedimento, quando comparadas com os homens.^24^

Inúmeros estudos demonstraram que o tratamento da FA baseado no *“Atrial Fibrillation Better Care (ABC) pathway”* determina reduções significantes no risco de AVC, infarto do miocárdio, hospitalização e mortalidade, não havendo diferenças relacionadas ao sexo.^145^ Essa estratégia (ABC) utiliza três condutas fundamentais: ‘A’ - evitar o AVC; ‘B’ - oferecer melhor controle dos sintomas com foco no paciente; e ‘C’ - prevenir e/ou tratar os FRCV e comorbidades que contribuem para o aparecimento da FA.^146^

No que diz respeito ao ‘C’, mulheres com FA têm maior prevalência de HAS, obesidade, depressão, ICFEp e doença valvar como causa da FA.^146,147^ A atividade física é outro preditor importante nas mulheres: quanto maior a intensidade do exercício, menor será a incidência de FA. Mulheres que praticam atividade física vigorosa tem 28% de redução na incidência de FA.^148^ Em um estudo prospectivo de larga escala com 30.034 mulheres, a menopausa não foi significativamente relacionada à incidência de FA, enquanto o uso de monoterapia com estrogênio foi associado ao aumento do seu risco, sugerindo uma ligação fisiopatológica entre a exposição ao estrogênio e a arritmia em mulheres.^149^ Em comparação com nulíparas, multiparidade foi associada a um aumento linear no risco de FA em uma grande coorte de mulheres inicialmente saudáveis. A exposição repetitiva a mudanças hormonais, metabólicas e fisiológicas durante a gravidez pode predispor a FA mais tarde na vida.^150^

Quanto ao ‘A’, mulheres com FA têm maior incidência de AVC que homens, sendo nelas o AVC mais grave e determinando maior incapacidade permanente, razão pela qual a anticoagulação, embasada em escores de risco, é essencial em ambos os sexos.^23,151^ A terapia antitrombótica com anticoagulantes orais encontra-se indicada para portadoras de FA com CHA_2_DS_2_VASc maior ou igual a 2, mas a decisão deve ser individualizada.^146,147^ Em pacientes com indicação do uso contínuo de anticoagulantes, é necessária a avaliação do risco de sangramento causado por esses medicamentos, antes de se iniciar a sua utilização. Um risco de sangramento alto não contraindica a anticoagulação e norteia a necessidade de maior controle desses fatores. Existem inúmeros escores que podem ser utilizados com essa finalidade, sendo o escore HAS-BLED recomendado nas últimas diretrizes de FA. São considerados de maior risco de sangramento indivíduos com pontuação ≥ 3.^146,147^ Estudos clínicos sobre anticoagulação na FA têm demonstrado maior segurança na utilização dos DOACs - dabigatrana, rivaroxabana, apixabana, edoxabana - em relação à varfarina. A oclusão percutânea do AAE tem sido indicada quando há contraindicação absoluta à utilização de anticoagulantes, como ocorre no sangramento intracraniano de causa irreversível. A oclusão cirúrgica ou exclusão do AAE pode ser considerada quando o paciente com FA tem indicação de cirurgia cardíaca.^23,146,147,151-153^

A respeito do ‘B’, mulheres com FA são mais sintomáticas, têm pior qualidade de vida e maior mortalidade, o que justifica a necessidade de uma abordagem adequada e precoce quanto ao controle da FC com DAA ou ablação do nódulo atrioventricular e, quando possível, à reversão ao ritmo sinusal com CVE, cardioversão química ou ablação por cateter.^23,149-151^ A ablação por cateter por meio do isolamento elétrico das veias pulmonares está atualmente indicada para o tratamento da FA paroxística/persistente em pacientes sem doença vascular do enxerto, naqueles com taquicardiomiopatia induzida pela FA, bem como naqueles com doença vascular do enxerto e IC para reduzir hospitalização e mortalidade.^146,147^

A associação entre FA e CMI é menos frequente em mulheres do que em homens, mas os elevados riscos dessa associação não apresentam diferenças relacionadas ao sexo.^146^ Em pacientes com SCA, a incidência de FA varia entre 2% e 23% e está associada a maior mortalidade hospitalar no primeiro mês e no primeiro ano, bem como a maior frequência de AVC, sem evidências de que existem diferenças relacionadas ao sexo. Os desfechos acima são mais frequentes nos indivíduos que desenvolvem FA durante a internação por SCA, quando comparados aos que já apresentavam FA anterior ao evento isquêmico agudo.^154^ A instabilidade hemodinâmica atribuída à FA na vigência da SCA deve ser tratada com CVE sincronizada. Em pacientes estáveis, o controle da FC pode ser obtido com o uso de BB ou BCC endovenosos. A administração de amiodarona é uma alternativa apropriada para o controle da frequência ventricular, bem como pode favorecer a reversão ao ritmo sinusal. A propafenona não deve ser utilizada em pacientes com FA e SCA para reversão ao ritmo sinusal.^146,147^

Estudos recentes sobre revascularização em mulheres e homens com FA e SCA ou síndrome coronariana crônica submetidos a angioplastia e implante de s*tent* demonstram que, nos pacientes com indicação de anticoagulação pela FA, há recomendação para utilização de dupla antiagregação plaquetária (ácido acetilsalicílico e um inibidor do receptor P2Y12 - clopidogrel, prasugrel, ticagrelor) por pelo menos uma semana, naqueles com menor risco de trombose e/ou maior risco de sangramento, podendo chegar a quatro semanas, com suspensão do ácido acetilsalicílico após esse período.^153^ A terapia dupla (anticoagulante + antiagregante) deve ser mantida por pelo menos 6 meses em pacientes com FA e síndrome coronariana crônica e por 12 meses naqueles com FA e SCA.^146,147,155^

Na CMI aguda ou crônica associada à FA, não há evidência de que estratégias de investigação ou de tratamento devam ser diferentes em mulheres e homens; portanto, essas estratégias precisam ser igualmente oferecidas para a prevenção das inúmeras e graves complicações relacionadas a essas doenças.

A Tabela 7.1 resume as indicações do tratamento atual para FA, bem como aponta as particularidades e perspectivas relacionadas ao sexo feminino.

**Tabela 7.1:** – Tratamento da fibrilação atrial: indicações, particularidades e perspectivas relacionadas ao sexo feminino.

ESTRATÉGIAS DE TRATAMENTO	INDICAÇÕES	PARTICULARIDADES	PERSPECTIVAS
CONTROLE DA FC	1. Controle dos sintomas; 2. Estratégias: drogas isoladas ou em associação para todos os pacientes com FA; 3. Meta: FC < 110 bpm.	1. São mais sintomáticas; 2. São menos recrutadas para ensaios clínicos.	1. Maior valorização dos sintomas para estratégia terapêutica; 2. Maior recrutamento de mulheres nos ensaios clínicos.
BB Bloqueador de canais de Ca (diltiazem, verapamil) Digoxina Ablação do nódulo atrioventricular	1. BB: primeira escolha para qualquer FEVE; 2. Diltiazem/verapamil: pacientes com FEVE > 40%; 3. Digoxina: pacientes com FEVE <40%; 4. Baixa dose de digoxina associada a menor risco; 5. Amiodarona e ablação: quando há falha nos demais.	1. Recebem prescrição mais frequente de digoxina; 2. Têm indicação mais frequente de ablação do nódulo atrioventricular (em detrimento da reversão para sinusal)	1. Prescrição adequada e limitada no tempo da digoxina; 2. Monitoramento adequado dos eletrólitos.
CONTROLE DO RITMO	1. Restaurar e manter o ritmo sinusal para controle de sintomas e melhora da qualidade de vida; 2. Estratégias: CVE, cardioversão química e/ou ablação por cateter (associados a anticoagulação, controle da frequência e tratamento dos fatores de risco para FA).	1. São mais sintomáticas e têm pior qualidade de vida; 2. São menos recrutadas para ensaios clínicos.	1. Maior valorização dos sintomas para estratégia terapêutica; 2. Investigação sistemática da qualidade de vida; 3. Indicação precoce e adequada de estratégias, incluindo a ablação por cateter; 4. Maior recrutamento de mulheres nos ensaios clínicos.
CVE Drogas classe I Drogas classe III Ablação por cateter Cirurgia de Maze	1. CVE para pacientes com FA e instabilidade hemodinâmica, inclusive na vigência de SCA; 2. Drogas classe I em pacientes sem cardiopatia estrutural (incluindo isquêmica); 3. Ablação por cateter (isolamento elétrico completo das veias pulmonares) indicada para FA paroxística/persistente em pacientes sem doença vascular do enxerto, com taquicardiomiopatia induzida pela FA e naqueles com doença vascular do enxerto para reduzir hospitalização e mortalidade; 4. Cirurgia de Maze tem indicação concomitante à cirurgia mitral ou de revascularização.	1. Recebem menos indicação de CVE; 2. São encaminhadas mais tardiamente para estratégias de reversão ao ritmo sinusal incluindo ablação por cateter; 3. Há relatos de mais complicações em mulheres submetidas à ablação; 4. Maior frequência de eventos adversos relacionados ao uso de antiarrítmicos (síndrome do QT longo adquirido; doença do nó sinusal);	1. Monitoramento dos eletrólitos e do ECG quando em uso de antiarrítmicos; 2. Desenho adequado de cateteres para ablação em mulheres.
ANTICOAGULAÇÃO	1. Reduzir a formação de trombos e prevenir o AVC em pacientes com CHA_2_DS_2_VASc ≥ 3 (mulheres) e com baixo risco de sangramento (HAS-BLED <3).	1. Têm maior frequência de AVC, que pode ser mais grave e com maior limitação permanente e maior mortalidade; 2. São menos recrutadas para ensaios clínicos.	1. Estratificação periódica dos escores de trombose e de sangramento, para adequação terapêutica; 2. Maior indicação, em doses adequadas, de DOACs; 3. Controle adequado do INR ao longo do tempo, quando em uso de varfarina; 4. Maior recrutamento de mulheres nos ensaios clínicos.
DOACs Varfarina Oclusão do AAE	1. DOACs são preferíveis à varfarina, exceto quando FA é associada a estenose valvar mitral moderada/grave ou a próteses mecânicas; 2. Oclusão do AAE está indicada quando há contraindicação ao uso de anticoagulantes orais; 3. Reavaliações periódicas de CHA_2_DS_2_Vasc e HAS-BLED; 4. No uso de varfarina: INR alvo = 2,0-3,0, com tempo na faixa terapêutica >70%. 5. Em mulheres com FA e SCA há indicação de uso de anticoagulantes orais quando CHA_2_DS_2_Vasc ≥ 2.	1. Recebem doses menores de anticoagulantes; 2. Em uso de varfarina permanecem menos tempo na faixa terapêutica de RNI e mantêm maior risco residual de AVC; 3. Apresentam menor risco de sangramento em uso de DOACs.	

*AAE: apêndice atrial esquerdo; AVC: acidente vascular cerebral; BB: betabloqueador; CVE: cardioversão elétrica; DOACs: anticoagulantes de ação direta; FA: fibrilação atrial; FC: frequência cardíaca; FEVE: fração de ejeção de ventrículo esquerdo; INR: international normalized ratio; SCA: síndrome coronariana aguda.*

### 7.2. Arritmias Ventriculares: Morte Súbita, Prevenção e Tratamento

A representatividade das mulheres em ensaios clínicos tem aumentado nas últimas décadas, mas é ainda muito baixa, principalmente na cardiologia, inclusive em estudos sobre arritmias.^156^ A despeito de todos os avanços médicos, a morte súbita cardíaca permanece a maior causa de morte cardiovascular nos pacientes com CMI.^157^ Nesse contexto, encontra-se a DIC aguda e crônica, com suas características e arritmias ventriculares distintas.^158^

Uma recente revisão sobre arritmias ventriculares na isquemia miocárdica aguda, com foco no papel da idade e do sexo, concluiu que há falta de entendimento dos mecanismos responsáveis pelas diferenças sexo-dependentes na susceptibilidade para arritmias ventriculares malignas durante a isquemia aguda.^159^ Esse dado, associado à baixa representatividade das mulheres em ensaios clínicos, contribui para a falta de evidências científicas suficientes para desenvolver métodos sexo-dependentes de prevenção de morte súbita cardíaca. A despeito da pobreza de dados em relação aos mecanismos de arritmias em mulheres, indica-se que parâmetros que promovem as arritmias ventriculares, como fibrose, hipertrofia cardíaca, anormalidades na sinalização de cálcio e alterações eletrofisiológicas, apresentam-se de forma diferente no que diz respeito a idade e sexo, ressaltando-se a necessidade de pesquisas adicionais.^159^ Nos principais estudos de prevenção primária, o percentual de mulheres submetidas ao implante de CDI variou de 8% a 29%, denotando a baixa representatividade das mulheres, o que torna difícil a avaliação do real benefício do procedimento nessa população. Ademais, as análises individuais desses estudos sobre o benefício de mortalidade não são adequadas, uma vez que os estudos não foram desenhados com poder estatístico para responder à questão.^160^

A prevenção e o tratamento das arritmias ventriculares de todas as etiologias envolvem, sem distinção de sexo, o controle dos FR modificáveis, a utilização de DAA, a ablação por cateter e o emprego do CDI, esse último como prevenção primária ou secundária.

A realização do implante de CDI em pacientes com indicação é outro ponto importante. Em 2007, Hernandes *et al.* avaliaram mais de 13.000 pacientes do programa da sociedade americana GWTG-HF (*American Heart Association’s Get With the Guidelines–Heart Failure*) e concluíram que havia disparidade nas taxas de implantes de CDI em homens afrodescendentes, mulheres afrodescendentes e mulheres brancas, que foram 27%, 38% e 44% menores do que a taxa de implantes em homens brancos, respectivamente.^161^ A análise do programa GWTG-HF em 2012 demonstrou melhora das taxas e término da disparidade, alertando sobre a necessidade de se manter a atenção e a educação continuada em todo o mundo.^162^ No entanto, ainda há uma enorme diferença entre os pacientes que necessitam, conforme as diretrizes, e os que recebem o CDI, especialmente mulheres.

Nos portadores de CDI, existem diferenças entre mulheres e homens relacionadas a outros desfechos além da mortalidade.^163^ Uma subanálise do estudo MADIT-CRT concluiu que as mulheres apresentam incidência menor de TV/FV, sugerindo que nelas a morte súbita não arrítmica seja maior do que nos homens. Além disso, a presença de terapia de choque apropriado pelo CDI foi um preditor de morte, principalmente nas mulheres.^164^ Em 2022, foi realizado um estudo de avaliação de arritmias ventriculares em mulheres (*propensity score-matched analysis*) nos portadores de CDI. Após pareamento para as principais comorbidades, indicações, terapia concomitante e dados demográficos, as mulheres permaneciam com menor perfil de risco para arritmia ventricular sustentada do que os homens, exceto no subgrupo de portadoras de TRC e naquelas com FEVE < 30%.^165^

A ablação por cateter é uma terapia efetiva no controle das arritmias ventriculares e recomendada em diversos cenários nas diretrizes.^160,166,167^ No entanto, o percentual de mulheres contempladas nos estudos de ablação de TV na população com DIC é baixo (7-13%). A menor indicação de procedimentos invasivos, a menor indução de TV sustentada e o menor número de choques apropriados são fatores que podem contribuir para esse percentual reduzido.^166^

### 7.3. Terapia de Ressincronização Cardíaca

Estudos clínicos como MIRACLE, COMPANION, CARE-HF, MADIT-CRT, RAFT, REVERSE e MIRACLE demonstram que a TRC melhora desfechos em pacientes sintomáticos com FEVE reduzida e duração prolongada do QRS. Embora nesses estudos o percentual de mulheres tenha variado de 17% a 33%, um benefício ainda maior foi demonstrado em mulheres em comparação com homens (Tabela 7.2).^160,167^Tais estudos foram realizados em pacientes isquêmicos e não isquêmicos, sendo que a isquemia esteve presente em 36% a 69% dos pacientes estudados.^168-173^

**Tabela 7.2:** – Resultados dos estudos clínicos com terapia de ressincronização cardíaca de acordo com o gênero.

Estudo	N de pacientes	Critério inclusão	Randomização	HR por eventos (95%; valor de p
COMPANION^2^	Homens: 1.025 (67%) Mulheres: 495 (33%)	FEVE ≤ 35% NYHA III-IV QRS ≥ 120ms	TMO vs TMO + TRC-D	HR para morte - homem: 0,63 (0,4-0,9) mulher: 0,58 (0,25-1,13)
CARE-HF^3^	Homens: 597 (73%) Mulheres: 216 (27%)	FEVE ≤ 35% NYHA III-IV QRS ≥ 130ms VDVE ≥ 300ms	TMO vs TMO + TRC	HR para morte ou hospitalização cardíaca - homem: 0,62 (0,49-0,79) mulher: 0,64 (0,42-0,97)
MADIT CRT^4^	Homens: 1.367 (75%) Mulheres: 453 (25%)	FEVE ≤ 30% NYHA II-III QRS ≥ 120ms	CDI vs TRC-D	HR para evento IC ou morte - homem: 0,76 (0,59-0,97) mulher: 0,37 (0,22-0,61)
RAFT^5^	Homens: 1.490 (83%) Mulheres: 308 (17%)	FEVE ≤ 30% NYHA II-III QRS ≥ 130ms	CDI vs TRC-D	HR para morte ou hospitalização por IC - homem: 0,82 (0,7-0,95) mulher: 0,52 (0,35-0,85)
REVERSE^6^	Homens: 479 (78,5%) Mulheres: 131 (21,5%)	FEVE < 40% NYHA I-II QRS > 120ms	TRC-on vs TRC-off	HR desfecho clínico composto - homem: 0,69 (0,43-1,11) mulher: 0,75 (0,26-2,19)
MIRACLE^7^	Homens: 216 (67%) Mulheres: 107 (33%)	FEVE < 35% NYHA III-IV QRS > 130ms	TRC-on vs TRC-off	NYHA, qualidade de vida, capacidade exercício, mulheres, mas homens com TRC não, experimentaram tempos mais longos para a primeira hospitalização por IC ou morte (p = 0,157)

*CDI: cardiodesfibrilador implantável; FEVE: fração de ejeção do ventrículo esquerdo; HR: hazard ratio; IC: insuficiência cardíaca; NYHA: classe funcional da New York Heart Association; TMO: terapia medicamentosa otimizada; vs: versus; TRC: terapia de ressincronização cardíaca; TRC-D: terapia de ressincronização cardíaca com desfibrilador; VDVE: volume diastólico final do ventrículo esquerdo; on: ligada, off: desligada.*

O estudo MIRACLE avaliou 453 pacientes com IC em classe funcional III ou IV da NYHA, com FEVE ≤ 35% e duração do QRS ≥ 130ms, comparando TRC ligada *versus* desligada, sendo 33% dos pacientes mulheres.^173^ Woo *et al*. analisaram 8 subgrupos pré-especificados no estudo MIRACLE e, quando avaliada a resposta de acordo com o sexo, observaram que as mulheres submetidas a TRC apresentaram tempos maiores para primeira hospitalização ou morte comparadas com o grupo controle, mostrando um benefício na TRC nas mulheres.^174^

No MADIT-CRT foram avaliados 1.820 pacientes com IC (25% eram mulheres) em classe funcional I ou II da NYHA, FEVE ≤ 30% e duração do QRS ≥ 130ms. O estudo RAFT avaliou 1.798 pacientes com IC (17% eram mulheres) em classe funcional II ou III da NYHA, FEVE ≤ 30% e duração do QRS ≥ 120ms ou ≥ 200ms quando estimulados por marca-passo prévio. Ambos os estudos compararam a terapia de ressincronização cardíaca com desfibrilador (TRC-D) *versus* CDI e demonstraram curva de sobrevida livre de IC favorável ao grupo da TRC-D, com redução no desfecho combinado de evento de IC e morte de 34% e 25%, respectivamente. Na análise multivariada em ambos os estudos para o desfecho morte e IC, observou-se um benefício pronunciado nas mulheres. No MADIT-CRT, mulheres que receberam TRC-D tiveram curva de sobrevida mais favorável que mulheres e homens que receberam CDI ou mesmo homens que receberam TRC-D. Houve redução de 72% de risco de morte quando comparadas mulheres com TRC-D *versus* CDI, mas não houve diferença na curva de sobrevida entre os homens com TRC-D *versus* CDI.^175^

Uma meta-análise do MADIT-CRT, RAFT e REVERSE com 4.076 pacientes, 22% dos quais do sexo feminino, demonstrou, na análise de desfecho IC e morte, curvas favoráveis a TRC nas mulheres. Também para o desfecho morte, foram encontrados os mesmos resultados com curva favorável a TRC *versus* CDI nas mulheres e sem diferença entre os homens.^176^

Um estudo avaliou 741 pacientes com idade de 66±11 anos, 33% mulheres, 78% brancas, FEVE 28±9%, 58% com CMI, 75% com BRE e volume sistólico final do VE de 65±30 ml/m^2^, submetidos a implante de TRC-D, randomizados para intervalo atrioventricular fixo de 120ms ou programado de acordo com ecocardiograma ou com o algoritmo SmartDelay. O desfecho foi composto por morte e hospitalização por IC e redução >15% no diâmetro diastólico final em 6 meses. A resposta de ambos os sexos à TRC foi semelhante, sendo a disparidade nos resultados da TRC atribuída ao sexo explicada pelas diferenças que as mulheres apresentam no substrato, no tratamento e nas comorbidades.^177^

Beela *et al*. investigaram até que ponto melhores resultados da TRC não poderiam ser explicados pelas características basais dos sexos. Avaliaram 1.058 pacientes (24% mulheres) submetidos a TRC com desfecho primário de mortalidade por todas as causas. As mulheres tinham menos CMI e menos fibrose, mais BRE e mais dissincronia mecânica. Levando-se em consideração as características basais, a resposta de sobrevida foi similar entre os sexos. Nesse grupo, o melhor resultado encontrado nas mulheres foi devido à menor taxa de CMI e à menor carga de fibrose.^178,179^

A presença do BRE é fator determinante de melhor resposta à TRC e o mesmo benefício não é observado quando o BRE não está presente.^21^ Estudo com 75.079 pacientes com IC em classe funcional III ou IV da NYHA, FEVE reduzida (≤ 35%) e duração do QRS ≥120ms, comparando a TRC entre mulheres e homens, mostrou benefício em ambos os sexos. O mesmo ocorreu na presença do BRE, embora tenha sido observada uma curva de mortalidade mais atenuada nas mulheres. No grupo sem BRE, as mulheres apresentaram melhor curva de mortalidade que os homens para QRS ainda mais largos (150-159ms).^180^ As mulheres com BRE respondem melhor que os homens com QRS mais estreitos e uma possível razão para isso é que elas têm menor massa ventricular esquerda e QRS mais estreitos do que os homens (aproximadamente 10ms a menos). Portanto, qualquer prolongamento absoluto no QRS das mulheres pode corresponder a um maior grau de dissincronia.^181^

As mulheres têm o dobro de complicações maiores relacionadas aos procedimentos de TRC. Essa diferença também pode ser observada de forma significativa entre mulheres e homens submetidos a implante de ressincronizadores, sendo a complicação mais encontrada o pneumotórax/hemotórax. Infecção com necessidade de reoperação também foi mais frequente nas mulheres. O maior preditor de complicações nas mulheres foi o menor índice de massa corporal (IMC).^182^ As principais diferenças entre mulheres e homens submetidos a TRC estão listadas no Quadro 7.1

**Quadro 7.1:** – Principais diferenças entre mulheres e homens submetidos a terapia de ressincronização cardíaca.

1. Mulheres representam cerca de 30% ou menos das populações dos estudos
2. Comparadas com homens, mulheres têm mais: ✓ Cardiomiopatia não isquêmica ✓ Bloqueio de ramo esquerdo e QRS mais estreito ✓ Complicações no procedimento ✓ Idade ✓ Hipertensão e diabetes
3. Comparadas com homens, mulheres têm menos: ✓ Fibrilação atrial ✓ Cardiomiopatia isquêmica ✓ Fração de ejeção reduzida

**Figura 7.1:**
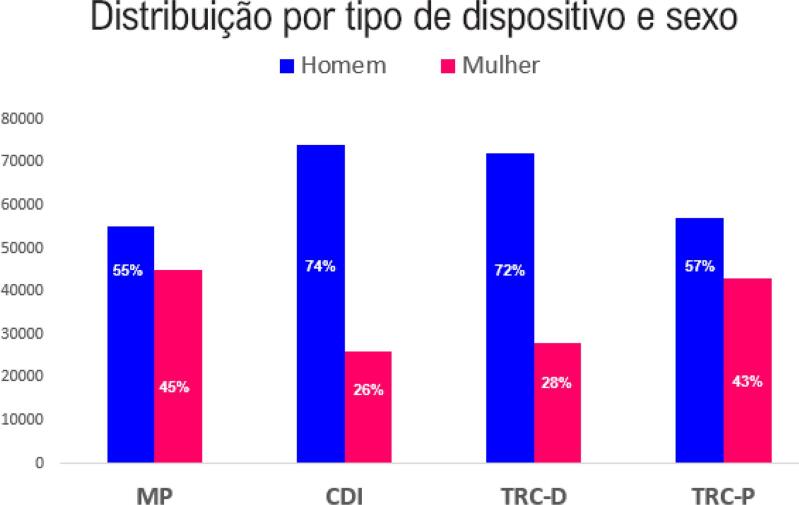
– Adaptado de Chen-Scarabelli et al.183 *Distribuição de dispositivos cardíacos eletrônicos implantáveis por tipo e por gênero. Adaptado de apresentação ACC 2015 Ellenbogen KA.*183 *MP: marca-passo; CDI: cardiodesfibrilador implantável; TRC-D: terapia de ressincronização com desfibrilador; TRC-P: terapia de ressincronização com marca-passo.*

Embora terapias como o CDI e a TRC tragam benefícios em termos de mortalidade, as mulheres recebem menos dispositivos que os homens (Figura 7.1). Essa desproporção sugere que ou as mulheres não preenchem os critérios para TRC, ou não são selecionadas nem mantidas de forma adequada nos estudos.^27,183^

### 7.4. Recomendações

Para acessar o risco de AVC é recomendado o escore de risco CHA_2_DS_2_VASc para identificar pacientes de baixo risco (pontuação do escore de CHA_2_DS_2_VASc =0 para homens e 1 para mulher), os quais não devem receber anticoagulação oral. A terapia antitrombótica com anticoagulantes orais encontra-se indicada para portadoras de FA com CHA2DS2VASc maior ou igual a 2, mas a decisão deve ser individualizada.^146,147^

As recomendações em relação a condutas nas arritmias são as mesmas para ambos os sexos.

## 8. Aterotrombose na Gravidez, Contracepção, Infertilidade, Síndrome Antifosfolípide

### 8.1. Introdução

Circunstâncias específicas do ciclo biológico da mulher acrescentam riscos que contribuem para a diversidade da evolução da aterosclerose e doença trombótica e ainda permanecem sob ampla investigação. Apresentamos neste capítulo tópicos específicos da idade reprodutiva, tais como gravidez, contracepção, infertilidade e SAF. Essa última, prevalente no sexo feminino, é considerada um potencial deflagrador da doença aterotrombótica na mulher.

### 8.2. Período da Gravidez

A DIC é pouco comum durante a gravidez. De acordo com dados da Organização Mundial da Saúde, a taxa de IAM é de 3,34 eventos por 100 mil gestações, sendo o IAM sem supradesnivelamento do segmento ST (IAMSSST) o mais frequente.^184^

**Figura 8.1:**
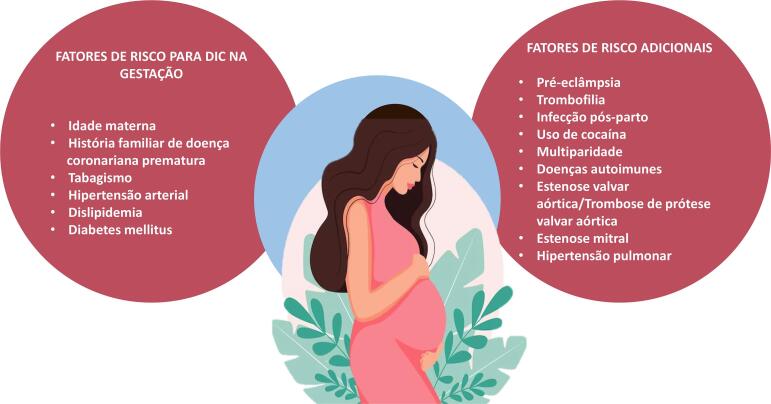
– Principais fatores de risco para doença isquêmica do coração na gravidez e fatores de risco adicionais. DIC: doença isquêmica do coração.

A Figura 8.1 mostra os principais FR para DIC durante a gravidez, além dos FR adicionais, delineados em anamnese minuciosa e exame físico completo.

**Figura 8.2:**
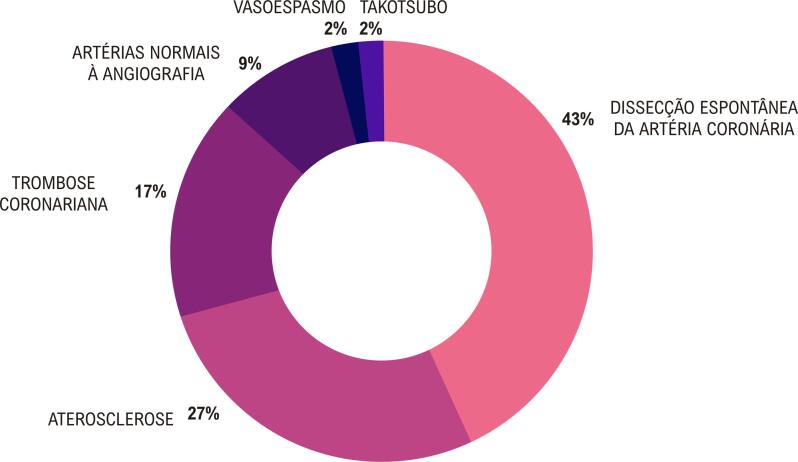
– Mecanismos fisiopatológicos do infarto do miocárdio durante a gravidez e o puerpério.

Idade materna acima dos 40 anos é um FR progressivo, de modo que, para cada ano de vida da mulher, há aumento de 20% de risco para infarto do miocárdio na gestação. Em uma revisão contemporânea,^185^ foram observados os mecanismos mais frequentes relacionados ao infarto do miocárdio durante a gestação e o puerpério (Figura 8.2).

A DEAC corresponde a quase metade das causas de infarto do miocárdio, com prevalência de cerca de 1,81 evento por 100 mil gestações, sendo mais frequente no último trimestre. Os resultados da DEAC associada à gestação têm pior prognóstico quando comparados aos da população geral.^51,186^

A conduta no infarto do miocárdio devido à doença aterotrombótica durante a gravidez e após o parto não difere daquela para a população geral, até mesmo em relação às técnicas de RVM percutânea ou cirúrgica.^187^ No que diz respeito à terapêutica farmacológica, o ácido acetilsalicílico em baixas doses é seguro para a gravidez e o feto,^188^ assim como o clopidogrel, devendo ser suspensos 7 dias antes da data programada do parto.

Nos casos de DEAC, geralmente o tratamento clínico tem sido o mais indicado, incluindo o uso de BB, que, à exceção do atenolol, podem ser utilizados durante todos os trimestres gestacionais.^186^ A ausência de isquemia residual ou disfunção ventricular permite a liberação para “nova” gestação com indicação individualizada.

A Tabela 8.1 sumariza os achados da DIC na gravidez e puerpério.

**Tabela 8.1:** – Resumo sobre definição, etiologia, fisiopatologia, apresentação clínica e prevenção da doença isquêmica do coração na gravidez e no puerpério.

	DEAC	ATEROSCLEROSE	VASOESPASMO	TROMBOSE CORONÁRIA	DOENÇA DE MICROCIRCULAÇÃO
DEFINIÇÃO	Ruptura espontânea da camada íntima arterial coronária e acúmulo de hematomas intramurais na “falsa luz” arterial	Relacionada aos fatores de risco tradicionais e emergentes para doença cardiovascular	Vasoconstrição difusa ou focal reversível da artéria coronária	Ausência de aterosclerose; possível relação com distúrbios de coagulação e hiperocoagulabilidade da gestação e puerpério; embolização paradoxal	Mecanismos ainda não estabelecidos, incluindo espasmos transitórios
ETIOLOGIA & FISOPATOLOGIA	Desarranjo e enfraquecimento da parede das artérias coronárias relacionadas aos hormônios da gravidez	Doença hipertensiva da gestação, diabetes gestacional, tabagismo, uso prolongado de anticoncepcionais anterior à gestação, idade acima de 35 anos	Disfunção endotelial, inflamação crônica, estresse oxidativo e hiper-reatividade do músculo liso	hipercoagulabilidade da gestação e fatores genéticos predisponentes	Aumento da reatividade vascular, uso de derivados da ergotamina
APRESENTAÇÃO CLÍNICA	Diversa: desde dor torácica leve à morte subita. Sintomas de síndrome coronariana aguda na gravidez e pós-parto	Dor torácica, mandibula, pescoço, fadiga e náuseas	Diversa: desde assintomatica até morte súbita, angina e infarto agudo do miocárdio		
PREVENÇÃO	Evitar estresse emocional, terapia hormonal e nova gravidez	Tratar os fatores de risco da doença cardiovascular e conscientização do diagnóstico precoce	Evitar drogas ilícitas, anfetamina e álcool		

*DEAC: dissecção espontânea de artéria coronária.*

### 8.3. Contracepção

A contracepção hormonal tem reconhecida eficácia e segurança em mulheres saudáveis, ainda que exista escassez de evidências quanto aos seus efeitos em portadoras de comorbidades, destacando-se HAS e doenças cerebrovasculares, ou com história de eventos isquêmicos, como trombose venosa profunda (TVP) e tromboembolismo pulmonar (TEP), independentemente da composição ou das vias de administração.

Nas últimas décadas, verifica-se um crescente número de mulheres jovens com doenças cardíacas, corroborando registros americanos que mostram uma prevalência de 11,5% de DCV entre mulheres na faixa etária de 20-29 anos.^189^ Adolescentes ou mulheres jovens frequentemente se deparam com a gravidez sem nunca terem recebido aconselhamento sobre planejamento da concepção. A gravidez, quer seja programada ou não, é uma “janela de oportunidade” para se propiciar a contracepção, tanto para mulheres saudáveis como para aquelas que apresentam algum tipo de comorbidade.^190^

Nesse cenário, vale mencionar que cerca de 0,69% da população brasileira identifica-se como transgênero.^191^ Essa faixa da população, apesar de ser designada feminina ao nascer, não recebe aconselhamento reprodutivo ao longo da vida, ainda que o uso do contraceptivo possa resultar em benefícios nos sintomas relacionados ao ciclo ovulatório e reduzir a gravidez indesejada.^192^

**Figura 8.3:**
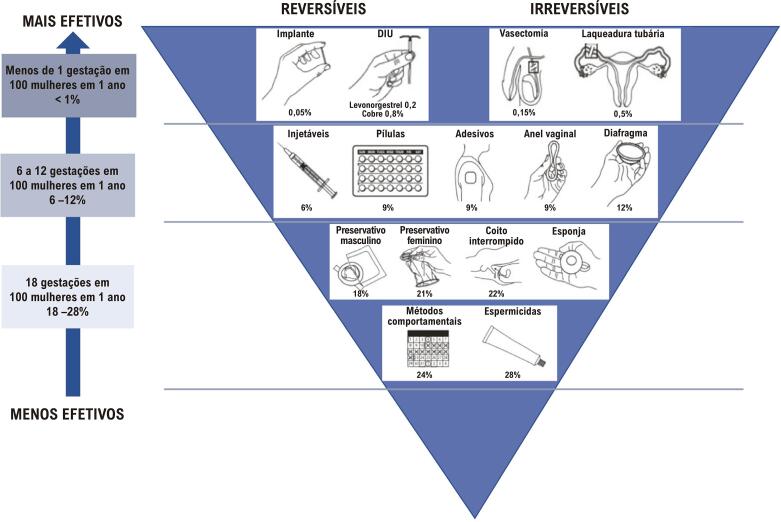
– Índice de Pearl dos principais métodos contraceptivos. DIU, dispositivo intrauterino. Fonte: adaptado de Curtis et al.*^193^*

Os contraceptivos hormonais são classificados em contraceptivos hormonais combinados (CHC - estrógeno e progesterona) orais e adesivos, contraceptivos progestágenos puros (CPP) e contraceptivos de longa duração (implante subcutâneo de etonogestrel, endoceptivo tratado com levonorgestrel). Entre os não hormonais, há o dispositivo intrauterino de cobre. O índice de falhas e eficácia pode ser calculado pelo índice de Pearl, que se baseia no número de gestações/100 mulheres/ano^193^(Figura 8.3).

Os contraceptivos em geral e os CHC, em particular, podem estar associados à doença aterotrombótica. Uma meta-análise demonstrou que o uso de CHC representa um risco 1,7 vez maior de infarto do miocárdio e de AVC isquêmico.^194^ O efeito deve-se à ação do estrogênio ao passar pelo fígado e promover alterações dos fatores hemostáticos que conduzem a um estado pró-coagulante e pró-trombótico. O mesmo não ocorre com contraceptivos CPP, exceto o acetato de medroxiprogesterona, que altera o perfil glicídico e lipídico.^195^

Ressalta-se que a presença dos FR para DCV (tabagismo > 15 cigarros/dia, idade > 35 anos, obesidade, HAS > 160/100mmHg, doença vascular, trombofilia, história de TVP/TEP, imobilização prolongada, AVC e isquemia miocárdica) eleva significativamente o risco de tromboembolismo em usuárias de contraceptivos hormonais. De acordo com os critérios de elegibilidade WHO-MEC 2015, nessas situações, estão contraindicados os CHC, os adesivos com contraceptivos combinados, o anel com contraceptivo combinado e o contraceptivo combinado injetável, podendo-se indicar os CPP, o implante subcutâneo de etonogestrel e o endoceptivo tratado com levonorgestrel^196^ (Quadro 8.1).

**Quadro 8.1:** – Uso de contraceptivos na doença trombótica e aterotrombótica.

Contraceptivos: Introdução e Continuação segundo Doença Trombótica e Aterotrombótica							
CONDIÇÃO	SUB-CONDIÇÃO	DIU-Cu	LNG	Implante	AMPD	CPP	CHC
DIC	Recente ou pregressa	1	2	3	3	2	4
Mutação trombogênica diagnosticada		1	2	2	2	2	4
FRCV	Idade avançada, tabagismo, DM, HAS, dislipidemia	1	2	2	3	2	4
AVC isquêmico	História de AVC	1	2	3	3	2	4
DCV	Não complicada Complicada (hipertensão pulmonar, fibrilação atrial e endocardite)	1	1	1	1	1	2
		1	1	1	1	1	4
TVP /TEP	História de TVP/TEP sem anticoagulação						
	Alto risco de recorrência	1	2	2	2	2	4
	Baixo risco de recorrência	1	2	2	2	2	3
	TVP/TEP agudo	2	2	2	2	2	4
	TVP/TEP anticoagulado por pelo menos 3 meses						
	Alto risco de recorrência	2	2	2	2	2	4
	Baixo risco de recorrência	2	2	2	2	2	4
	História familiar	1	1	1	1	1	2
	Grande cirurgia						
	Com imobilização prolongada	1	2	2	2	2	4
	Sem imobilização prolongada	1	1	1	1	1	2
	Pequena cirurgia sem imobilização	1	1	1	1	1	1

*Categorias: 1= sem restrição de uso; 2= benefício se sobrepõe ao risco potencial; 3= risco se sobrepõe ao benefício; 4= risco inaceitável. AMPD: acetato de medroxiprogesterona de depósito; AVC: acidente vascular cerebral; CHC: contraceptivo hormonal combinado; CPP: contraceptivo progestágeno puro; DCV: doença cardiovascular; DIC: doença isquêmica cardíaca; DIU-Cu: dispositivo intrauterino de cobre; DM: diabetes mellitus; FRCV: fatores de risco cardiovascular; HAS: hipertensão arterial sistêmica; LNG: Levonorgestrel; TEP: tromboembolismo pulmonar; TVP: trombose venosa profunda. Fonte: adaptado de Curtis et al.*^193^

Concluindo, embora os CHC sejam os mais utilizados em todo o mundo, destacamos que os implantes subcutâneos e os endoceptivos compostos por progestágenos puros apresentam menor impacto na doença aterotrombótica. Vale reforçar que a seleção da contracepção deve considerar os fatores intrínsecos da paciente, a segurança e a eficácia dos contraceptivos, além de envolver uma abordagem multidisciplinar ao longo da fase reprodutiva da mulher.^197^

#### 8.3.1. Recomendações

O uso de contraceptivos na doença trombótica e aterotrombótica deve ser direcionado segundo a presença ou não de FR para DCV, sendo nessas situações contraindicados os CHC, os adesivos com contraceptivos combinados, o anel com contraceptivo combinado e o contraceptivo combinado injetável. Nessas situações, podem-se indicar os CPP, o implante subcutâneo de etonogestrel e o endoceptivo tratado com levonorgestrel.^193^

## 8.4. Infertilidade

A infertilidade é uma doença caracterizada pela incapacidade de se estabelecer uma gravidez clínica após 12 meses de relações sexuais regulares e desprotegidas. Estima-se que afete entre 8% e 12% dos casais em idade reprodutiva em todo o mundo. A causa secundária é a forma mais comum de infertilidade feminina, muitas vezes devida a infecções do trato reprodutivo. Os três principais fatores que influenciam na probabilidade espontânea de concepção são o tempo de conceber uma gravidez programada, a idade da parceira e as causas relacionadas às doenças.^198^

Os fatores que afetam a fertilidade de ambos os sexos são hipogonadismo hipogonadotrófico, hiperprolactinemia, distúrbios da função ciliar, fibrose cística, infecções, doenças sistêmicas e fatores relacionados ao estilo de vida, enquanto a insuficiência ovariana prematura, síndrome do ovário policístico (SOP), endometriose, miomas uterinos e pólipos endometriais desempenham papel importante como causas da infertilidade feminina.^199^

Afora essas comorbidades, a infertilidade está frequentemente associada a distúrbios mentais, como depressão e transtornos de ansiedade, e tem forte relação com DCV pelo impacto dos hormônios androgênicos nos FR da DCV e na síndrome metabólica.^200^

Uma meta-análise^201^ que comparou grupos de mulheres da mesma faixa etária, com ou sem infertilidade, mostrou maior frequência de distúrbios metabólicos pró-ateroscleróticos, particularmente obesidade e aumento do colesterol total, LDL-colesterol e triglicerídeos entre as mulheres que sofriam infertilidade. Nessa meta-análise, um estudo isolado^202^ mostrou um aumento de DIC, AVC e IC nas mulheres com infertilidade no seguimento de pelo menos 5 anos em comparação às saudáveis (HR 1,35; 1,16–1,57; p < 0,0001).

As mulheres com SOP apresentam maior risco de obesidade, HAS, intolerância a glicose, dislipidemia e apneia obstrutiva do sono. Dentre as alterações metabólicas, a obesidade está presente em cerca de 50% dos casos. A resistência à insulina presente em 60% a 95% dos casos gera intolerância à glicose em 31% a 35% e DM tipo 2 em 7,5% a 20% dessas mulheres. Contudo, as alterações do perfil lipídico caracterizadas pelos baixos níveis de HDL-colesterol, altos níveis plasmáticos de triglicérides e aumento do LDL-colesterol são a anormalidade metabólica mais frequente na SOP.^203^

No decurso do 20º ano do estudo prospectivo CARDIA, a análise das imagens de calcificação de artérias coronárias e das medidas da espessura da camada médio-intimal (EMI) carotídea demonstrou risco elevado de DCV subclínica (OR 2,70; IC 95%, 1,31 – 5,60) quando hiperandrogenismo e anovulação estiveram presentes na SOP.^204^ Esses dados foram reforçados na meta-análise de Zhang *et al.*, que verificaram aumento de risco de infarto do miocárdio, DIC e AVC (OR 1,66; IC 95%, 1,32 – 2,08) nas portadoras de SOP.^205^

De igual importância, outra causa de infertilidade é a endometriose, com prevalência de quase 10% na população e que, por sua vez, está associada com doenças crônicas, como asma e doenças autoimunes, cânceres ginecológicos e DCV.^206^ Na endometriose, ocorre um processo inflamatório crônico mediado por substâncias, como molécula de adesão intercelular (ICAM-1), interleucina 1 e 6 (IL-1 e IL-6), fator de necrose tumoral (TNF-a) e fator de crescimento do endotélio vascular (VEGF), que induzem aumento do estresse oxidativo e do LDL-colesterol, com subsequente efeito aterogênico.^207^

Tem-se demonstrado que a endometriose está associada a FR bem estabelecidos para a DCV, como HAS e dislipidemia, com perfil aterogênico maior e aumento do risco de tromboembolismo venoso, doença arterial coronariana, IC e AVC. O estudo que considerou como desfecho primário a composição de DIC, IC e doença cerebrovascular estabeleceu a associação positiva desses eventos com endometriose (OR 1,24; 95% IC, 1,13 – 1,37), colocando-a como FR para DCV.^208^ Essas observações foram concordantes com um estudo prospectivo que mostrou a associação de endometriose com aumento do risco de infarto do miocárdio/isquemia coronariana e RVM cirúrgica e percutânea.^209^

A terapia de fertilização é considerada como um potencial FR para os distúrbios hipertensivos na gestação.^210^ Contudo, uma revisão sistemática que agrupou SCA, AVC, tromboembolismo venoso, HAS e DM em mulheres que se submeteram a tratamento de fertilização não demonstrou aumento dos eventos combinados. As sérias limitações dessa análise, com apenas seis estudos muito heterogêneos, não atenderam critério estatístico para nos dar uma evidência mais robusta.^211^

Em conclusão, a faixa etária reprodutiva é um momento oportuno para a estimativa do RCV ao longo da vida das mulheres. Nessa fase do ciclo biológico feminino, é importante a atenção para os norteadores de riscos e intervenções em patologias clínicas que não são incluídas nos escores de RCV tradicionais, estratificando essas mulheres de forma diferente para prevenção efetiva da DCV.

## 8.5. Síndrome Antifosfolípide

A SAF é uma doença trombótica autoimune que acomete preferencialmente mulheres jovens em uma proporção de 5:1.^212,213^ O diagnóstico é feito na suspeita clínica de trombose em qualquer leito vascular e/ou diante de complicações obstétricas recorrentes, tais como abortos espontâneos, partos prematuros e pré-eclâmpsia/eclâmpsia. A SAF causa insuficiência placentária e restrição do crescimento intrauterino associadas à presença persistente de anticorpos antifosfolipídeos (aPL): anticardiolipina (aCL), anti-beta2-glicoproteína 1 e/ou lúpus anticoagulante (LAC).^213^

Os aPL estão fortemente associados ao AVC em mulheres com idade abaixo de 50 anos. A presença de aCL e/ou LAC em pacientes jovens que sofreram AVC isquêmico ou ataque isquêmico transitório, sem diagnóstico concomitante de lúpus eritematoso sistêmico, é frequente. Além disso, pacientes com aPL e isquemia cerebral têm maior frequência de eventos múltiplos do que pacientes sem esses anticorpos. Um estado pró-trombótico associado a aPL pode ser um fator determinante de isquemia recorrente em mulheres com aterosclerose.^214^

A SAF está associada a infarto do miocárdio em aproximadamente 2,8% de pacientes acometidos pela doença. O mecanismo de isquemia miocárdica na SAF é considerado trombose aguda das artérias coronárias, que requer anticoagulação terapêutica, em contraste com a ruptura da placa aterosclerótica, que é efetivamente tratada com antiplaquetários e *stent.* Dada a forma diferente de tratamento, a distinção entre as pacientes que apresentam a SAF não diagnosticada é fundamental para o sucesso terapêutico e o prognóstico. Embora incomum, a SAF deve ser considerada em mulheres jovens com infarto do miocárdio, especialmente se forem identificadas tromboses prévias não provocadas, contagens de plaquetas mais baixas, tempo parcial de tromboplastina elevado e artérias coronárias normais ou trombose coronária. A anticoagulação deve ser mantida por toda a vida, mesmo no primeiro episódio. O papel do *stent* coronário nessas pacientes requer mais estudos.^215^

A evidência de títulos significativamente elevados de diferentes aPL na fase inicial do infarto do miocárdio sugere que esses anticorpos estão presentes antes do evento e não são secundários a ele. O desaparecimento de aPL elevado após 3 meses do infarto do miocárdio pode ser devido a um efeito de absorção ou possivelmente a um fenômeno cíclico semelhante ao encontrado em outras doenças autoimunes. Os aPL podem ser FR adicional para infarto do miocárdio e devem ser considerados especialmente em pacientes de faixa etária mais jovem sem FRCV aparentes.^216^

Os critérios clínicos e laboratoriais para o diagnóstico da SAF devem estar presentes de forma concomitante em uma janela inferior a 5 anos. O progresso nos conhecimentos sobre as bases moleculares do envolvimento vascular permite considerar que a SAF seja uma doença de origem multifatorial que se desenvolve em indivíduos geneticamente predispostos. Diversos mecanismos contribuem para o desenvolvimento de trombose nos pacientes com SAF, destacando-se o efeito sinérgico de autoanticorpos com moléculas pró-trombóticas, receptores de adesão, mediadores inflamatórios, estresse oxidativo e moléculas de sinalização intracelular.^217^

A presença dos aPL induz um estado pró-aterotrombótico através da expressão de moléculas pró-trombóticas e pró-inflamatórias, incluindo fator tecidual e VEGF, além de indução de estresse oxidativo e disfunção mitocondrial em monócitos e neutrófilos, juntamente com aumento da formação de “nichos” extracelulares de neutrófilos.^218^

A predisposição genética tem sido demostrada nas associações do antígeno leucocitário humano (HLA) com a doença e a ocorrência de aPL nos pacientes com SAF. Os genes do complexo principal de histocompatibilidade parecem influenciar não apenas na produção de autoanticorpos, mas também na própria expressão da doença.^218^ Polimorfismos genéticos também foram associados a trombose em pacientes com SAF, incluindo variantes de fatores de coagulação, moléculas antitrombóticas e mediadores inflamatórios.^219^ Os aPL induzem alterações genômicas e epigenéticas que sustentam um estado pró-trombótico.

**Figura 8.4:**
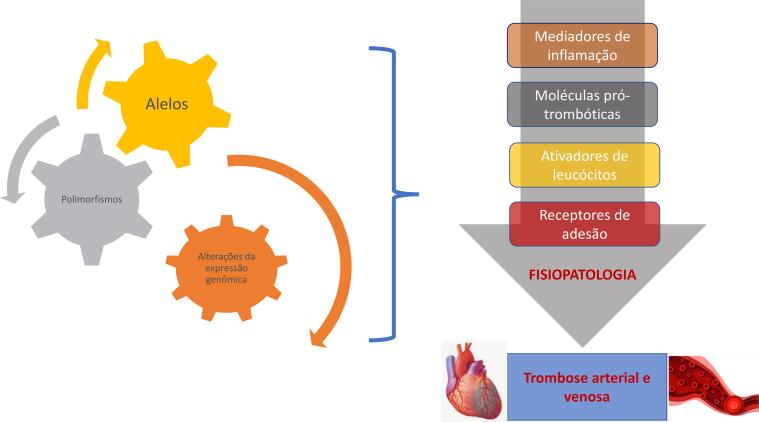
– Fatores de risco genômicos de aterotrombose na síndrome antifosfolípide (SAF).*^217^*

A epigenética, definida como mudanças ou modificações no DNA que influenciam o fenótipo sem alterar o genótipo, representa um mecanismo novo de regulação gênica. Mecanismos regulatórios epigenéticos e pós-transcricionais estão alterados nas doenças autoimunes e cardiovasculares, como modificações na metilação do DNA, nas histonas e nas atividades de microRNAs, alterando a expressão de genes e proteínas.^217^ Os microRNAs afetam o sistema imunológico e têm um papel importante na patogênese de condições autoimunes e inflamatórias, atuam como principais reguladores de vários alvos gênicos envolvidos nas características clínicas da SAF, como resposta imune, aterosclerose e trombose.^220^ É sabido que dois microRNAs (miR-19b/miR-20a) funcionam como potenciais moduladores do fator tecidual, o principal receptor envolvido no desenvolvimento da trombose na SAF. Assim, uma assinatura específica de circulação de microRNAs foi recentemente identificada em pacientes com SAF como potenciais biomarcadores^221^(Figura 8.4).

Uma vez que a paciente tenha tido um evento trombótico, independentemente de ser venoso ou arterial, a terapia de escolha é a varfarina, com alvo de INR (*International Normalized Ratio*) entre 2 e 3. A heparina, administrada de forma aguda no momento do evento trombótico, pode ter dois benefícios: primeiro, bloqueando a ativação do complemento (mesmo que apenas uma dose profilática), e segundo, como anticoagulante. Dado o alto risco de recorrência na SAF, a anticoagulação é recomendada a longo prazo, porque o risco de recorrência chegou a 24% quando a anticoagulação foi interrompida, particularmente no caso de lúpus eritematoso sistêmico.^214^

O contínuo progresso no conhecimento das bases genômicas e dos biomarcadores epigenéticos impulsiona a farmacologia clínica e proporciona maior segurança da terapêutica da SAF. Assim, embora estudos mais amplos sejam necessários, as novas descobertas permitem melhor entendimento sobre a possibilidade de novos modelos direcionados para opções terapêuticas na prevenção de trombose da SAF.

A menopausa representa uma transição significativa na vida da mulher e caracteriza-se pela cessação do ciclo menstrual e subsequente diminuição dramática nos níveis de hormônios sexuais, estrogênio e progesterona. Embora a testosterona também diminua em mulheres pós-menopáusicas, essa diminuição é mais gradual e os efeitos dos andrógenos na saúde da mulher após a transição da menopausa não são totalmente compreendidos. A terapia de reposição hormonal é um método pelo qual as mulheres podem controlar os sintomas da menopausa durante e após essa transição. No entanto, não há compreensão suficiente dos efeitos precisos que diferentes formas de terapia de reposição hormonal têm na fisiologia da mulher e particularmente no contexto do risco de DCV. A interação de idade avançada e sexo feminino com DCV em conjunto com os papéis de terapia de reposição hormonal e menopausa na progressão da DCV leva a questões futuras para abordar essas lacunas na compreensão atual da saúde de mulheres idosas em risco de DCV.^222^

### 8.5.1. Recomendações

A SAF deve ser considerada em mulheres jovens com infarto do miocárdio, em situações de tromboses prévias não provocadas, contagens de plaquetas mais baixas, tempo parcial de tromboplastina elevado e artérias coronárias normais ou trombose coronária. A anticoagulação deve ser mantida por toda a vida, mesmo no primeiro episódio, particularmente no lúpus eritematoso sistêmico.^214,215^

## 9. Cardiomiopatia Isquêmica na Mulher

### 9.1. Introdução

Em pesquisa envolvendo portadores de CMI submetidos a transplante cardíaco, a avaliação dos corações explantados apresentava aumento da massa do VE, do volume dos miócitos e do comprimento celular nos homens. Essa diferença não foi vista na cardiomiopatia idiopática, sugerindo que o gênero possa influenciar na adaptação miocárdica local à lesão isquêmica.^223^

A evolução após IAM entre mulheres é mais grave. Maiores índices de reinternação e mortalidade são registrados nas mulheres quando comparadas aos homens, persistindo mais altos com 1 (26% x 19%) e 5 anos (47% x 36%) após o quadro agudo.^224^ Já a IC sintomática após IAM é mais incidente no sexo feminino, especialmente nas mulheres mais velhas. O aumento do risco se prolonga além do episódio inicial do IAM. O sexo feminino é um preditor independente para choque cardiogênico, mesmo no IAM com menor extensão de DIC.^224^

Apesar de melhor FEVE e menor carga de DIC obstrutiva, mulheres com DIC e CMI apresentam menor capacidade funcional e qualidade de vida, mas mortalidade semelhante.^126^

Maior riqueza de informações na CMI diz respeito aos homens, possivelmente pela maior frequência. A HAS e o DM contribuem mais para o risco de IC nas mulheres, com padrão fenotípico distinto.^7^

Mesmo com debate ampliado na última década sobre a desigualdade na atenção às DCV entre gêneros, especialmente no tratamento do IAM, a menor inclusão de mulheres em ensaios clínicos e a menor otimização de seu tratamento persistem na atualidade. Os homens são mais prontamente atendidos e encaminhados para tratamento assim como recebem a terapia orientada por diretrizes com maior frequência.^2^ Provavelmente, de alguma forma, essa limitação diagnóstica-terapêutica esteja influenciando nos desfechos desfavoráveis entre as mulheres.

Em relação ao remodelamento em vigência de DIC, as diferenças parecem ter relação com hormônios sexuais, além do impacto de cromossomos sexuais e epigenética. Foi demonstrado que vários genes relacionados a processos adversos de remodelação cardíaca, como ativação de macrófagos, apoptose e metabolismo lipídico, estão localizados no cromossomo.^225^

Há relatos ainda de que as mitocôndrias femininas toleram melhor a privação de oxigênio e o dano oxidativo do que as masculinas, além de as mulheres parecerem estar mais bem protegidas da apoptose induzida por isquemia/reperfusão.^224,226^ Níveis elevados de cálcio aumentam a lesão de isquemia-reperfusão e o estrogênio pode diminuir os níveis de cálcio antes da isquemia, levando a menos lesões em mulheres.^227^

Em modelos animais de infarto e fibrose por apoptose maciça, a necrose na área infartada é mais extensa com mais conteúdo de colágeno em camundongos machos em comparação com fêmeas.^228^ Esse dado foi confirmado em ensaios com humanos, onde a apoptose após infarto do miocárdio segue o mesmo padrão, sendo mais prevalente em homens.^229^

Dessa forma, ainda que não pareçam melhorar mortalidade e hospitalizações, os estrogênios podem proteger contra o estresse oxidativo, modular a apoptose e, em especial, o estradiol pode reduzir fibrose ao regular negativamente a síntese de colágeno, ainda que a mortalidade permaneça alta.^224^

Na CMI, os dados sugerem que a obesidade seja mais frequente na população feminina, correlacionando-se com pior prognóstico em comparação com os homens.^230^

Quanto aos sintomas de IC, as mulheres tendem a se apresentar mais sintomáticas, com queixas de dispneia e ortopneia ao longo de todo o espectro de classe funcional quando comparadas com os homens.^230^

Diversos métodos não invasivos estão disponíveis para o diagnóstico da CMI na mulher e, em muitos casos, podem auxiliar não apenas no diagnóstico, como também na avaliação prognóstica e nas definições terapêuticas. A escolha da melhor modalidade deve levar em conta o desempenho diagnóstico, as peculiaridades relacionadas ao gênero e, em alguns casos, a presença de situações especiais, como gestação.

A angio-TC tem alta sensibilidade e performance similar em mulheres e homens.^231^ A adição de tecnologias para avaliação funcional permite a identificação de lesões hemodinamicamente significativas e auxilia nas definições terapêuticas. Além disso, ajuda no diagnóstico de DEAC, que afeta principalmente mulheres. A RMC tem elevada acurácia diagnóstica tanto em mulheres como em homens.^232^ Ademais, na cardiomiopatia, diferentes padrões de realce tardio e alterações teciduais podem auxiliar na identificação de etiologias não isquêmicas.

Em suma, diversas modalidades não invasivas estão disponíveis para avaliação de CMI. Conhecer as vantagens e desvantagens de cada uma auxilia na escolha do método a ser utilizado e na interpretação dos resultados. É importante lembrar, entretanto, que a avaliação invasiva com coronariografia pode ser necessária e não deve ser retardada quando restar dúvidas. Ainda, quanto maior a gravidade da cardiomiopatia e da apresentação clínica, bem como na presença de características que aumentem a probabilidade de etiologia isquêmica, seja pela presença de sintomas anginosos ou de alterações segmentares, avaliações definitivas que excluam ou identifiquem a presença da etiologia isquêmica devem ser consideradas.

### 9.2. Tratamento Clínico

Nas últimas décadas, avanços no tratamento da IC com fração de ejeção reduzida (ICFEr) foram observados em diferentes ensaios clínicos. Assim, as principais diretrizes recomendam o uso da terapia quádrupla, com IECA/BRA ou inibidores da neprilisina/bloqueadores de angiotensina (ARNI) + BB + antagonista da aldosterona + inibidores da SGLT2, para o tratamento de pacientes com ICFEr por apresentar impacto sobre mortalidade geral e cardiovascular.^233-235^

Para os pacientes que persistem sintomáticos, outras classes de medicamentos, como hidralazina-nitrato, ivabradina, digoxina e, mais recentemente, estimuladores da guanilato ciclase, além de TRC, podem ser acrescidas. Vale ressaltar que, na maioria desses estudos clínicos, a etiologia isquêmica foi a mais frequente, variando de 50% a 70% (Tabela 9.1). Ainda como tratamento com impacto em redução de mortalidade está indicado o uso de CDI, especialmente em pacientes com CMI.^7^

**Tabela 9.1:** – Estudos empregados na terapia da cardiomiopatia isquêmica.

Estudos	Medicamento	Mulheres	Isquêmicos	População	Desfecho primário	Resultado	Interação Homem/Mulher	P interação
**Ácido acetilsalicílico**								
Prevenção secundária	16 ensaios clínicos de prevenção secundária	----	Todos	17.000 pacientes Alto risco cardiovascular	Evento vascular sério	HR 0,81 (0,75–0,87) RRR 19%	Homens: HR 0,81 (0,73–0,90) Mulheres: HR 0,81 (0,64–1,02)	NS
**BB**								
CIBIS II	Bisoprolol* 10mg/d	20%	50%	2.647 pacientes IC NYHA CF III-IV FEVE ≤ 35% Seguimento: 16m	Morte geral	HR 0,66 (0,54-0,81) RRR = 34% NNT = 18	Homens: HR 0,53 (0,42–0,67) Mulheres: HR 0,37 (0,19–0,89)	NS
MERIT HF	Succinato de metoprolol* 200mg/d	22%	65%	3.991 pacientes IC NYHA CF II-IV FEVE ≤ 40% Seguimento: 12m	Morte geral	HR 0,66 (0,53–0,81) RRR = 34% NNT = 27	Em mulheres não houve redução de morte geral (7,5% x 6,9%)	< 0,05
COPERNICUS	Carvedilol* 25mg 2xd	20%	67%	2.289 pacientes IC NYHA CF IV FEVE < 25% Seguimento: 11m	Morte geral	RRR = 35% NNT = 15	Sem diferença significativa	NS
CIBIS II MERIT HF COPERNICUS					Morte geral (dados agrupados)		Homens: HR 0,66 (0,58 to 0,75) Mulheres: HR 0,69 (0,51 to 0,93)	NS
**IECA/BRAS**								
SOLVD	Enalapril* 10mg 2xd	20%	70%	2.569 pacientes IC NYHA CF II-IV FEVE ≤ 35% Seguimento: 37m	Morte geral	HR 0,84 (0,74-0,95) RRR = 16% NNT = 22	Em mulheres não houve redução de morte geral	< 0,05
CHARM	Candesartana* 32mg/d	32%	67%	2.028 pacientes IC NYHA CF II-IV FEVE < 40% Seguimento: 33m	Morte CV ou HIC	HR 0,77 (0,67–0,89) RRR = 27% NNT = 14	Sem diferença significativa	
**Antagonista de Aldosterona**								
RALES	Espironolactona* 25-50mg/d	27%	54%	1.663 pacientes IC NYHA CF III-IV FEVE ≤ 35% Seguimento: 24m	Morte geral	HR 0,69 (0,58-0,82) RRR = 31% NNT = 10	Homens: HR 0,71 (0,60–0,82) Mulheres: HR 0,72 (0,57–0,97)	NS
EMPHASIS	Eplerenone* 25-50mg/d	22%	68%	2.737 pacientes IC NYHA CF II FEVE ≤ 35% Seguimento: 21m	Morte CV ou HIC	HR 0,66 (0,56–0,78) RRR = 37% NNT = 13	Sem diferença significativa	0,36 (NS)
**ARNI**								
PARADIGM-HF	Sacubitril-Valsartana^†^ 200mg 2xd	21%		8.442 pacientes IC NYHA CF II-IV FEVE < 40% / FEVE ≤ 35% Seguimento: 27m	Morte CV ou HIC	HR 0,80 (0,73–0,87) RRR = 20% NNT = 21	Homens: HR 0,80 (0,73–0,87) Mulheres: HR 0,79 (0,66–0,94)	NS
**ISGLT2**								
DAPA-HF	Dapagliflozina* 10mg/dia	23%	55%	4.744 pacientes IC NYHA CF II-IV FEVE < 40% Seguimento: 18m	Morte CV ou HIC	HR 0,75 (0,65–0,85) RRR = 26% NNT = 21	Homens: HR 0,73 (0,63–0,85) Mulheres: HR 0,79 (0,59–1,06)	NS
EMPEROR-Reduced	Empagliflozina* 10mg/dia	24%	51%	3.730 pacientes IC NYHA CF II-IV FEVE < 40% Seguimento: 16m	Morte CV ou HIC	HR 0,75 (0,65–0,86) RRR = 25% NNT = 19	Homens: HR 0,80 (0,68–0,93) Mulheres: HR 0,59 (0,44–0,80)	NS
**Vasodilatadores diretos**								
A-HeFT	Hidralazina 225mg/d + Dinitrato de Isossorbida* 120mg/d	36%	23%	1.050 negros IC NYHA CF III/ IV FE ≤ 35%, ou FE < 45% se DDVE > 6,5cm ou > 2,9cm/m^2^ Seguimento: 18m	Escore composto primário (1ª. HIC, qualidade de vida e sobrevida livre de eventos)	RRR = 43% NNT = 25	Homens: HR 0,67 (0,49–0,92) Mulheres: HR 0,58 (0,39–0,86)	NS p = 0,806
**Inibidores If**								
SHIFT	Ivabradina* 5 – 7,5mg 2xd	23%	67%	6.558 pacientes IC FEVE < 35% Ritmo sinusal / FC > 70 Seguimento: 23m	Morte CV ou HIC	HR 0,82 (0,75–0,90) RRR = 18% NNT = 26	Homens: HR 0,84 (0,76–0,94) Mulheres: HR 0,74 (0,60–0,91)	NS
**Digitálicos**								
DIG	Digoxina* 0,25mg/d	22%	65%	6.800 pacientes FEVE < 45% IC NYHA CF II-III Seguimento: 37m	Morte geral Ausência de redução	Não houve redução de mortalidade	Aumento do risco de morte em mulheres se nível > 1,2 mg/mL	---
**Terapia de Ressincronização**								
MADIT-CRT	TRC-D versus CDI isolado	25%	50%	1.820 pacientes FEVE < 30% IC NYHA CF I-II QRS > 30ms Seguimento: 4,5 a	Morte geral ou evento IC não fatal	HR 0,66 (0,52–0,84) Maior benefício em mulheres	Homens: HR 0,76 (0,59–0,97) Mulheres: HR 0,37 (0,22–0,61)	p = 0,01

*ARNI: inibidores da neprilisina e bloqueadores dos receptores de angiotensina; BB: betabloqueadores; BRA: bloqueadores dos receptores de angiotensina II; CDI: cardiodesfibrilador implantável; CF: classe funcional; CV: cardiovascular; FC: frequência cardíaca; FEVE: fração de ejeção ventricular esquerda; HIC: hospitalização por IC; HR: hazard ratio; IC: insuficiência cardíaca; IECA: inibidores da enzima de conversão da angiotensina; iSGLT2: inibidores do cotransporte de sódio e glicose 2; NNT: definido para desfecho primário / morte por todas as causas no tempo total de seguimento; NS: não significativo; NYHA: New York Heart Association; RRR: redução de risco relativo; TRC: terapia de ressincronização cardíaca; TRC-D: terapia de ressincronização cardíaca associada a desfibrilador.*

Apesar dos avanços recentes, há desafios no tratamento de mulheres com CMI. O primeiro deles refere-se à sub-representação delas nos principais ensaios clínicos de ICFEr. Enquanto as mulheres representam aproximadamente 50% dos pacientes com ICFEp, na ICFEr, a representação delas varia de 20% a 30% apenas. Dessa forma, as diferenças gênero-específicas são limitadas, baseiam-se em análises de subgrupos e, por esse motivo, devem ser interpretadas com cautela.^7^

A terapêutica da CMI e os seus principais estudos balizadores são apresentados a seguir:

1. **Betabloqueadores:** meta-análise envolvendo os principais estudos de BB em ICFEr revelou que a redução de mortalidade ocorre de forma semelhante em mulheres e homens. Subanálises revelam que bisoprolol melhorou a sobrevida de 515 mulheres estudadas no CIBIS-II (HR 0,37; IC95%, 0,19 – 0,69), assim como carvedilol reduziu desfecho combinado de morte e hospitalização em 469 mulheres com FEVE < 25% no subestudo do COPERNICUS (HR 0,23; IC95%, 0,07–0,69) e succinato de metoprolol reduziu tempo de hospitalização por IC em mulheres com FEVE < 25% (p = 0,004), porém não reduziu mortalidade isoladamente.^236-238^
2. **Inibidores da ECA:** estudos com IECA, como SOLVD e CONSENSUS, demonstraram impacto na redução de mortalidade em pacientes com ICFEr em classe funcional II/III e IV da NYHA, respectivamente; porém, quando as mulheres foram avaliadas isoladamente, tal benefício não foi observado.^239^
3. **Bloqueadores dos receptores de angiotensina II:** dados de subanálise dos estudos CHARM (candesartana) mostraram que não há diferenças relacionadas ao sexo quanto ao desfecho primário do estudo de morte cardiovascular e hospitalização por IC.^240^
4. **Antagonista dos receptores de angiotensina-inibidores da neprilisina:** análise de subgrupos do estudo PARADIGM-HF sugere não haver diferença entre mulheres e homens em relação ao desfecho primário de morte cardiovascular ou hospitalização por IC. Recentemente, um estudo de vida real envolveu 427 pacientes, sendo 29% mulheres, e demonstrou tolerabilidade ao uso de ARNI semelhante em mulheres e homens.^241^
5. **Antagonistas de aldosterona:** a análise de subgrupos de estudos envolvendo espironolactona (RALES) e eplerenone (EMPHASIS) revelou que o impacto sobre morte geral e morte cardiovascular/hospitalização por IC, respectivamente, ocorre tanto em mulheres quanto em homens, não havendo diferença estatisticamente significativa.^7^
6. **Inibidores de SGLT2:** os estudos que avaliaram dapagliflozina (DAPA-HF) ou empagliflozina (EMPEROR-Reduced) em pacientes com ICFEr demonstraram que a adição dessa nova classe de medicações à terapia tripla gera redução de desfecho combinado de morte cardiovascular e hospitalização por IC e tal benefício ocorre de forma semelhante em mulheres e homens.^242,243^(Tabela 9.1).

Em relação às terapias adicionais, a subanálise dos estudos A-HeFT, que investigou a associação hidralazina-nitrato, e SHIFT, que avaliou ivabradina em pacientes com ICFEr, revelou redução de desfecho primário tanto em mulheres quanto em homens. Quanto ao uso de digoxina, não houve redução de mortalidade no estudo DIG na população total, mas é importante ressaltar que, no subgrupo de mulheres que apresentavam digoxinemia fora da faixa terapêutica, ou seja, maior do que 1,2 mg/ml, houve maior taxa de mortalidade. Portanto, sugere-se o uso de doses mais baixas e controle rigoroso de nível sérico em mulheres quando indicado o uso dessa medicação.^244-246^

Conforme descrito anteriormente, a maioria dos ensaios clínicos em ICFEr revelou eficiência equivalente à do tratamento medicamentoso em mulheres e homens, devendo-se utilizar as medicações que são modificadoras de prognóstico em ambos os sexos. No entanto, vale destacar que tais observações são resultantes de análises de subgrupos de estudos e, portanto, devem ser analisadas com cautela.

A Tabela 9.1 resume os dados dos principais ensaios clínicos em CMI e seus resultados comparando mulheres e homens.

### 9.3. Dispositivos e Insuficiência Cardíaca Avançada

Os avanços em dispositivos elétricos e mecânicos continuaram com benefícios substanciais para os sintomas, hospitalização e resultados dos pacientes, com fortes evidências em ambos os sexos.

A gama de dispositivos inclui CDI, TRC e desfibriladores de TRC. No entanto, dados recentes mostram que as mulheres são menos propensas a receber CDI e, quando o fazem, têm taxas mais altas de complicações relacionadas à implantação, como pneumotórax e infecção.^230^

### 9.4. Cardiodesfibrilador Implantável

Como já mencionado, as mulheres menos frequentemente têm como etiologia a DIC e, como resposta de remodelamento, menos fibrose e uma taxa mais baixa de arritmias ventriculares, resultando em menos morte súbita cardíaca. Uma meta-análise avaliando estudos que envolveram pacientes (n = 7.229) com cardiomiopatia isquêmica (74%) e não isquêmica, sendo 22% mulheres, mostrou que o benefício foi significativamente maior em homens (HR 0,67; IC 95%, 0,58-0,78, p < 0,001) do que em mulheres (HR 0,78; IC 95%, 0,57-1,05, p = 0,1).^247^

No entanto, mesmo após ajuste para idade e comorbidades, o sexo feminino tem menor probabilidade de receber um CDI quando comparado com o masculino.^248^

Ainda importante levar em conta que as mulheres têm taxas mais altas de complicações relacionadas ao implante de dispositivo, como pneumotórax, infecção, sangramento e tamponamento.^249^

Por outro lado, pelo perfil menos fibrótico e até mesmo por características estruturais e anatômicas, os estudos sugerem que as mulheres respondam mais favoravelmente à TRC, resultando em melhoria dos sintomas, qualidade de vida, FEVE e mortalidade. Dados do estudo MADIT-CRT, que comparou TRC-D ao implante de CDI isolado, revelaram maior benefício em mulheres conforme a Tabela 9.1 (p = 0,01).^170^

### 9.5. Insuficiência Cardíaca Avançada

Dispositivos de assistência ventricular já estão bem implementados na prática dos serviços de IC avançada como ponte ou destino. As mulheres são mais propensas a hospitalizações por IC avançada, mas menos a receber um dispositivo de assistência ventricular. Em estudo recente analisando o registro EUROMACS, 966 pacientes (151 mulheres) foram incluídos. Caracteristicamente, à época do implante, as mulheres apresentavam-se em piores perfis INTERMACS 1 e 2 (51,7% *versus* 41,6% nos homens) e experimentaram mais complicações, como sangramento maior (p = 0,001), arritmias (p = 0,02) e insuficiência ventricular direita (p < 0,001), com pior sobrevida em 1 ano (75,5% *versus* 83,2%).^7^

O transplante cardíaco continua sendo o padrão-ouro para o tratamento da IC avançada, com apenas 25% dos receptores do sexo feminino, em geral pela dificuldade de *match* entre altura, peso, tipo sanguíneo e painel imunológico. As mulheres também apresentam índices maiores de complicações após transplante cardíaco, incluindo rejeição mediada por anticorpos e vasculopatia do enxerto.

### 9.6. Recomendações

#### 9.6.1. Manejo Clínico e Indicações de Terapias Avançadas

**Classe I/B -** Pacientes femininas com CMI e IC com fração de ejeção reduzida devem receber tratamento medicamentoso guiado por diretrizes de IC.^233-235^

**Classe I/B -** Pacientes femininas com CMI e IC com fração de ejeção melhorada devem receber tratamento medicamentoso guiado por diretrizes de IC.^233-235^

**Classe I/B -** Pacientes femininas com CMI e IC com fração de ejeção preservada (FEVE > 50%) devem receber tratamento medicamentoso guiado por diretrizes de IC.^233-235^

**Classe I/C -** Pacientes femininas com CMI e IC com fração de ejeção reduzida, refratárias a tratamento medicamentoso guiado por diretrizes de IC devem ser encaminhadas à ressincronização cardíaca.^233-235^

**Classe I/C –** Pacientes do sexo feminino com CMI e IC avançada, refratárias a terapia medicamentosa e não medicamentosa guiada por diretrizes devem ser consideradas para transplante cardíaco.^233-235^

## 10. Intervenção Coronariana Percutânea

### 10.1. Introdução

Estudos recentes relatam um aumento significativo nas taxas de mortalidade nos casos de doença coronariana aguda em mulheres jovens (< 55 anos de idade).^250^ Apesar das crescentes evidências demonstrando diferenças entre os sexos quanto aos FR basais, anatomia e função coronariana, apresentação de sintomas, comorbidades, eficácia do tratamento e desfechos nas SCA, os mecanismos por trás dessas diferenças são em parte desconhecidos.^67^ Essas lacunas de conhecimento decorrem da sub-representação de mulheres na pesquisa clínica, devendo-se encorajar esforços da comunidade científica para a mudança desse paradigma.

Apesar do benefício geral da RVM, o sexo feminino tem sido consistentemente associado a um risco aumentado de sangramento e a complicações vasculares associadas à ICP, evidenciando a necessidade de se considerarem as principais diferenças biológicas, como o tamanho dos vasos dos acessos percutâneos e a prevalência de DIC não obstrutiva nas mulheres, além da necessidade de se aplicarem os cuidados orientados pelas diretrizes vigentes.^251^

Em comparação aos homens, as mulheres submetidas a ICP são mais velhas e têm maior prevalência de insuficiência renal, anemia e DM. As mulheres com SCA apresentam maior mortalidade e menor uso de terapias recomendadas, como estratégia invasiva precoce, e de terapia antitrombótica do que os homens.^252^

Fatores clínicos, como idade avançada, insuficiência renal, choque cardiogênico e uso de introdutores maiores, foram especificamente identificados como preditores de risco de sangramento em mulheres. No entanto, a propensão feminina para sangramento persiste além desses FR. Mecanismos específicos do sexo referentes a IMC, anatomia do sítio de punção, biologia plaquetária e farmacoterapia relacionada à ICP podem desempenhar um papel importante.

### 10.2. Acesso Vascular para o Cateterismo Cardíaco e Intervenção Coronariana Percutânea em Mulheres

Mason Sones realizou a primeira coronariografia seletiva em 1958 através de dissecção da artéria braquial. Em 1967, Judkins e Amplatz desenvolveram a técnica da punção da artéria femoral e os cateteres apropriados para essa técnica, que ainda hoje é bastante utilizada por ser excelente via em casos de angioplastias complexas que requerem introdutores maiores e em estudos de pontes e enxertos de pacientes com RVM cirúrgica prévia.^253^

A via radial, descrita em 1989 por Campeau, é uma técnica mais complexa e exige maior habilidade e experiência, assim como maior curva de aprendizagem. As mulheres apresentam, em maior proporção, artérias radiais de menor calibre e com mais tortuosidades, quando comparadas aos homens. Pacientes com doença renal crônica, DM, baixo IMC e idosos apresentam maior taxa de insucesso do acesso radial.^254,255^

O acesso por meio da artéria femoral predominou por décadas, devido às suas significativas viabilidade e reprodutibilidade. O acesso por meio da artéria radial, por sua vez, tem-se mostrado eficiente na redução de sangramentos e outras complicações vasculares, especialmente em pacientes com SCA e, por isso, vem se tornando a via de acesso de escolha nos últimos anos.^253^

O uso de acesso radial também pode estar associado a melhor qualidade de vida e menores custos em comparação ao acesso femoral.^253,255,256^ Entretanto, a magnitude do benefício associado ao acesso radial pode variar amplamente entre os pacientes, dependendo principalmente do risco do paciente de complicações do acesso femoral. Portanto, é fundamental garantir que o acesso radial seja usado preferencialmente em pacientes com maior risco de complicações do acesso vascular, como as mulheres.^256^

### 10.3. Diagnóstico

#### 10.3.1. Angiografia Coronária

As mulheres têm artérias coronárias epicárdicas significativamente menores do que os homens, mesmo após ajuste para idade, estrutura corporal e massa ventricular esquerda. A discrepância no tamanho dos vasos coronários entre mulheres e homens é considerada uma base importante para as diferenças entre os sexos nos desfechos da DIC, apesar do menor volume de placa aterosclerótica nas mulheres.^67,77^

A implicação importante do tamanho menor do vaso é que a carga comparativamente menor de placa aterosclerótica e trombo pode resultar em doença obstrutiva. Essa pode ser uma razão potencial para maior incidência de morte súbita cardíaca em mulheres mais jovens, apesar de menor carga de placa. Ademais, vasos menores podem representar um risco maior de reestenose.^77^

**Figura 10.1:**
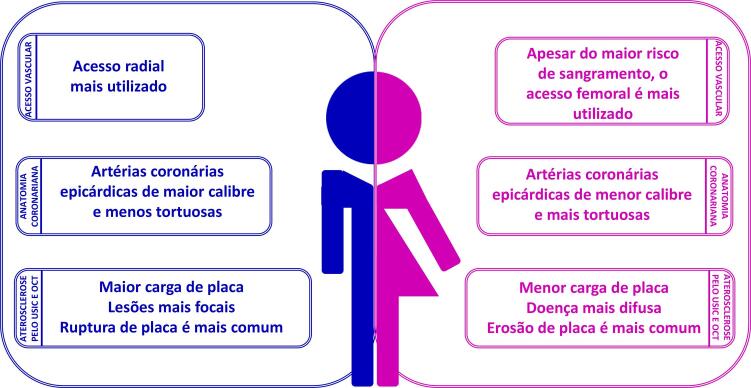
– Diagnóstico da doença isquêmica do coração. Criação dos autores. USIC, ultrassom intracoronariano; OCT, tomografia de coerência óptica.

Além disso, o fluxo sanguíneo miocárdico basal e hiperêmico, conforme avaliado pela PET, é tipicamente maior em mulheres em comparação com homens. Sugere-se, portanto, que o diâmetro menor das artérias coronárias epicárdicas femininas, juntamente com seu fluxo sanguíneo miocárdico basal mais alto, resulte em um aumento significativo nas condições de estresse de cisalhamento endotelial em mulheres, podendo explicar algumas diferenças sexuais na suscetibilidade à DIC^67,77^ (Figura 10.1).

#### 10.3.2. Imagem Intravascular

As características da placa aterosclerótica, a resposta vascular ao implante de *stents* coronarianos e o endotélio vascular podem ser avaliados com estreita correlação com os achados histopatológicos por meio dos métodos de imagem intravascular realizados durante o cateterismo cardíaco. As tecnologias atuais de imagem intracoronária incluem principalmente o ultrassom intracoronariano (USIC), a OCT e a espectroscopia de infravermelho próximo (NIRS).^67^

O USIC permite a quantificação precisa da carga de placa aterosclerótica com resolução axial de 70-200 mm e penetração > 5mm. Por esse método, o estudo PROSPECT (*Providing Regional Observations to Study Predictors of Events in the Coronary Tree*) conseguiu avaliar o efeito do sexo na extensão e nas características da DIC e comprovar diferenças significativas entre os sexos. As mulheres apresentam DIC menos extensa. A morfologia e a composição da placa na mulher apresentam menor núcleo necrótico e calcificação, com similar carga aterosclerótica de placa, porém menor área luminal mínima e mais lesões com área luminal mínima < 4,0mm^2^, comparada ao homem.^257^

Já a OCT tem uma resolução lateral de 10-20mm e permite a caracterização de placas e trombos, mas com pouca penetração, impedindo a avaliação precisa da carga de placa aterosclerótica. No entanto, sua utilização deve-se à sua alta resolução e ao seu poder de detecção da morfologia da placa aterosclerótica, ajudando na identificação das placas vulneráveis ou fibroateromas de capa fina. Além disso, a OCT permite o esclarecimento de alguns mecanismos de agudização da doença coronariana não obstrutiva, como a dissecção, e corrobora achados que permitem diferenciar a doença coronariana entre os sexos*.*^258,259^ O NIRS usa um cateter de fibra óptica com um padrão de absorção específico para lipídios e outros componentes da placa, que consegue melhorar a acurácia da identificação da placa aterosclerótica vulnerável.^67^

Juntos, esses três métodos de imagem intravascular nos permitiram conhecer algumas diferenças nas características da aterosclerose e da DIC entre os sexos. Mulheres têm menor carga de placa, a erosão é mais prevalente que a ruptura como mecanismo de agudização da DIC e observou-se maior concentração de cristais de colesterol e calcificação nas placas ateroscleróticas rotas.^67,259^

#### 10.3.3. Testes Invasivos com Guia de Medição

A rotina diagnóstica e terapêutica tem se concentrado na estenose das artérias coronárias epicárdicas, embora as evidências nas últimas décadas tenham estabelecido o conceito de que a estenose epicárdica obstrutiva não é condição obrigatória para causar sintomas isquêmicos de doença coronariana estável. Inúmeras publicações evidenciam que mulheres sintomáticas são mais propensas do que homens a apresentar DIC não obstrutiva e disfunção microvascular coronariana, perfazendo até cerca de 40% dos casos.^260^

**10.3.3.1. Reserva de Fluxo Fracionada**

A medição da FFR consiste no uso de fios-guia de angioplastia com ponta de sensor que foram desenvolvidos para medir a pressão e o fluxo através de uma estenose coronária no laboratório de cateterismo. A FFR mede as pressões proximal (pressão aórtica) e distal (pressão do fio-guia) nas lesões estenóticas no fluxo máximo e cria uma relação de pressão, representando a proporção do fluxo através dessa estenose. Para medições precisas de FFR, são necessárias pressões obtidas durante hiperemia. O fluxo sanguíneo máximo (hiperemia) é mais comumente induzido por adenosina intravenosa (140mcg/kg/min) ou intracoronária (artéria coronária direita 50-100mcg, artéria coronária esquerda 100-200mcg em *bolus*). A razão entre a pressão coronária distal e a pressão aórtica (conforme registrada no cateter-guia) durante a hiperemia máxima é chamada de FFR. Um valor normal é 1, enquanto valores < 0,80 estão associados a isquemia provocável com uma precisão maior que 90%.^261^

Para graus determinados de estenose, a FFR em pacientes do sexo feminino tende a ser muito maior do que naqueles do sexo masculino. Considerando que pacientes do sexo feminino correm maior risco de mortalidade intra-hospitalar e resultados adversos após ICP, o papel da medida do FFR deve ser enfatizado especialmente em mulheres para evitar ICP desnecessária.^262^

**10.3.3.2. Razão de Pressão Instantânea Livre de Onda**

Um índice derivado da pressão de repouso, independente da adenosina, foi desenvolvido e testado como substituto da FFR. Usando análise de intensidade de onda, determinou-se que o período de diástole em que o equilíbrio entre as ondas de pressão da aorta e a reflexão microcirculatória distal era o “período livre de ondas” atendeu aos requisitos da FFR para ter um mínimo de resistência constante. A pressão diastólica/pressão na aorta (Pd/Pa) durante o período sem onda (75% na diástole terminando 5 ms antes da onda R) é chamada de razão de pressão instantânea livre de onda (iFR). Foi demonstrado que, em pontos de corte de iFR > 0,93 ou < 0,86, houve uma forte correlação com valores normais e anormais de FFR (usando 0,80 como ponto de corte de FFR). No estudo ADVISE II, a iFR foi comparada à FFR em 690 estenoses intermediárias. Comparado à FFR (< 0,80), o corte da iFR de 0,89 classificou corretamente 83% das estenoses. A iFR classificou corretamente essas estenoses fora da zona cinza de iFR igual a 0,85 a 0,94 com 92% de concordância. Assim, a abordagem híbrida iFR-FFR para estenose intermediária pode ser avaliada sem a necessidade de estímulo hiperêmico em 65% dos pacientes.^262,263^

#### 10.3.4. Testes Funcionais

A vasoconstricção microvascular coronária também pode ser avaliada através de testes funcionais, usando-se a acetilcolina intracoronária, produzindo vasodilatação coronariana na presença de endotélio saudável e vasoconstrição paradoxal na presença de disfunção endotelial. Doses incrementais são administradas durante 3 minutos até que a resposta seja produzida ou a dose-alvo seja atingida.

A resposta positiva para espasmo coronário epicárdico é redução focal ou difusa do diâmetro da artéria coronária > 90% (em comparação com o estado relaxado). Os pacientes apresentam reprodução da angina e alterações isquêmicas no ECG. Esses pacientes serão considerados como tendo angina vasoespástica.

A resposta positiva para a vasoconstrição microvascular é a ausência de espasmo coronariano epicárdico (nenhuma redução do diâmetro ou redução < 90%). Os pacientes têm reprodução da angina e alterações isquêmicas do ECG (infradesnivelamento ou elevação do segmento ST).

**Figura 10.2:**
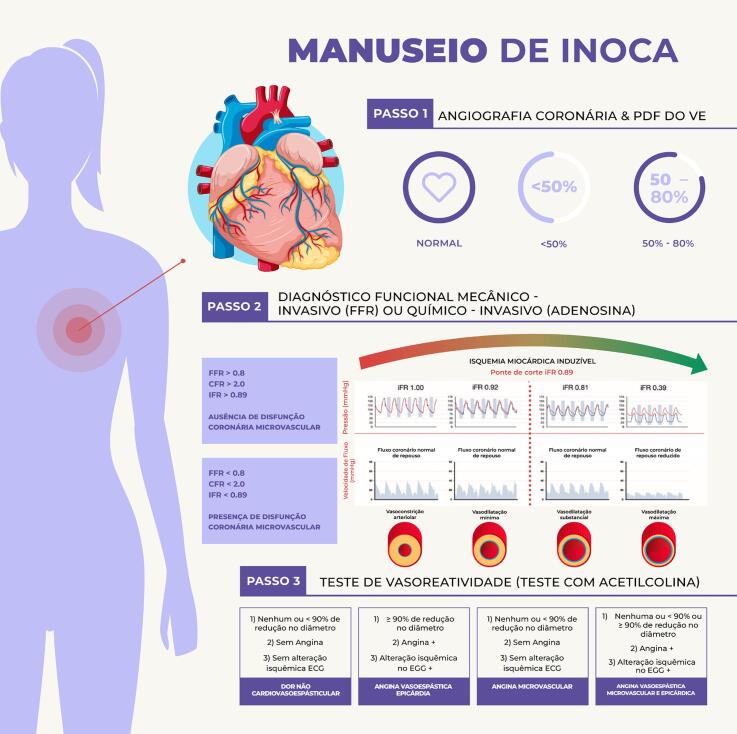
– Manuseio da INOCA. Baseada no Consenso EAPCI sobre INOCA.77 *PDF: pressão diastólica final.*

A resposta negativa ao teste de acetilcolina é a ausência de espasmo coronariano epicárdico (nenhuma redução do diâmetro ou redução < 90%), sem angina e sem alterações isquêmicas no ECG (Figura 10.2).

Em sendo o teste com acetilcolina negativo, tem-se ainda a opção de teste com adenosina intracoronária. A RFC é calculada a partir de velocidade de fluxo coronariano (CFV), que é a razão entre a CFV de pico e a CFV da linha de base. A RFC < 2,0 indica disfunção microvascular coronariana independente do endotélio. Esses pacientes com RFC anormal também serão considerados como tendo alteração na microcirculação.^260^

## 10.4. Tratamento Percutâneo da Doença Aterotrombótica Coronária em Mulheres

### 10.4.1. Revascularização para Síndromes Coronarianas Crônicas

O objetivo da RVM nas síndromes coronarianas crônicas é o alívio da angina e não a melhora da mortalidade. Portanto, ao considerar as opções de tratamento médico ou ICP, deve-se levar em consideração a maior incidência e frequência de angina nas mulheres. Em uma subanálise do estudo COURAGE, não houve diferenças significativas no efeito do tratamento nos principais resultados entre mulheres e homens. No entanto, as mulheres designadas para ICP demonstraram maior benefício em relação aos homens, com redução da hospitalização por IC e da necessidade de RVM futura.^264^

O ensaio clínico ISCHEMIA mostrou que as mulheres apresentaram maior carga de sintomas de angina do que os homens, apesar de terem doença coronariana menos extensa e isquemia menos grave.^265^

**10.4.1.1. Doença do Tronco de Coronária Esquerda**

Até anos recentes, a cirurgia de RVM era o tratamento recomendado para a doença do tronco da coronária esquerda (TCE), mas a ICP tem sido cada vez mais adotada no tratamento da doença do TCE.

No ensaio EXCEL,^266^ as mulheres submetidas a ICP do TCE desprotegido apresentaram tendência a piores desfechos, achado relacionado a comorbidades associadas e aumento das complicações periprocedimento. No entanto, o sexo não foi um preditor independente de desfechos adversos após a RVM, conclusão encontrada também no estudo NOBLE.^267^

**10.4.1.2. Oclusão Total Crônica**

As oclusões totais crônicas representam um subgrupo importante de lesões coronárias e são encontradas em até 18% das angiografias diagnósticas.

É conhecido que a ICP com oclusão total crônica bem-sucedida está associada ao alívio sintomático da angina, melhora da função ventricular esquerda e da qualidade de vida e redução da mortalidade. Contudo, vários estudos têm mostrado que, na mulher, a ICP com oclusão total crônica bem-sucedida não foi associada a risco reduzido de mortalidade cardiovascular ou de eventos coronarianos adversos maiores (MACE) em comparação com o tratamento clínico isoladamente, o que difere dos homens, que apresentam redução significativa na taxa de MACE após ICP com oclusão total crônica bem-sucedida.^268^

### 10.4.2. Revascularização para Infarto do Miocárdio sem Supradesnivelamento do Segmento ST

**Figura 10.3:**
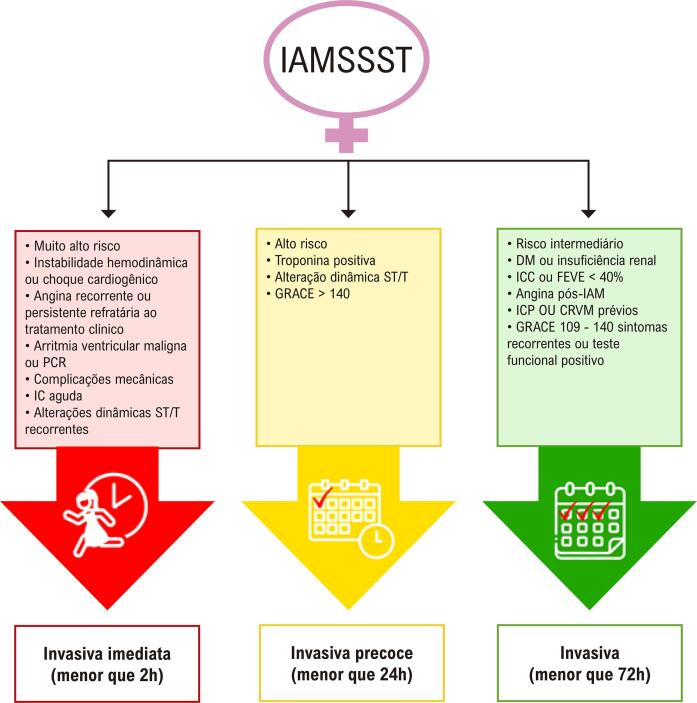
– Avaliação invasiva no infarto agudo do miocárdio sem supra do segmento ST. Baseado na Diretrizes da Sociedade Brasileira de Cardiologia sobre Angina Instável e Infarto Agudo do Miocárdio sem Supradesnível do Segmento ST – 2021.*^65^* CRVM: cirurgia de revascularização do miocárdio; DM: diabetes mellitus; FEVE: fração de ejeção de ventrículo esquerdo; IAM: infarto agudo do miocárdio; IAMSSST: infarto agudo do miocárdio sem supradesnivelamento do segmento ST; IC: insuficiência cardíaca; ICC: insuficiência cardíaca congestiva; ICP: intervenção coronariana percutânea; PCR: parada cardiorrespiratória.

Em pacientes com IAMSSST, a abordagem invasiva inicial está associada a melhores resultados e a uma taxa mais baixa de desfecho combinado de morte, infarto do miocárdio ou angina refratária em 4 a 6 meses de acompanhamento, especialmente em pacientes de alto risco; contudo, as mulheres são submetidas a ICP com menor frequência, especialmente as mais jovens.^269^ Os benefícios de uma abordagem invasiva são mais pronunciados entre pacientes com biomarcadores elevados ou outros achados de alto risco, independentemente do sexo.^270^Um fluxograma resume a escolha da estratégia e o momento da avaliação invasiva (Figura 10.3).^65^

### 10.4.3. Revascularização para Infarto do Miocárdio Com Supradesnivelamento do Segmento ST

**Figura 10.4:**
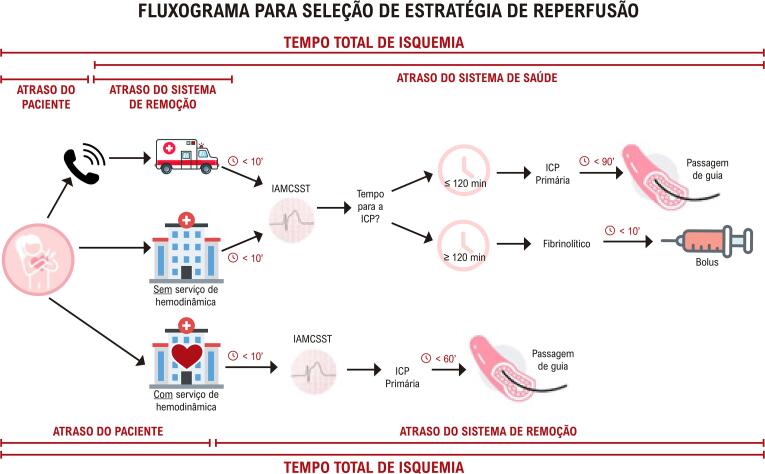
– Seleção da estratégia de reperfusão. Baseada na 2018 ESC/EACTS Guidelines on Myocardial Revascularization.271 *IAMSSST: infarto agudo do miocárdio sem supradesnivelamento do segmento ST; ICP: intervenção coronariana percutânea.*

O sexo feminino tem sido associado a demora na apresentação hospitalar desde o início dos sintomas e a atrasos na ICP primária, o que tem sido atribuído, em parte, a sintomas atípicos em mulheres. Além disso, as mulheres são menos propensas a receber terapias invasivas para o IAMCSST, possivelmente devido a mais comorbidades e fragilidade na admissão e a menos doença coronariana obstrutiva na angiografia. Juntos, esses atrasos e disparidades no atendimento contribuem para uma pior mortalidade hospitalar em mulheres com IAMCSST. Uma vez iniciado o tratamento, as taxas de sucesso do procedimento, fluxo epicárdico pós-procedimento, perfusão miocárdica e resolução do segmento ST são semelhantes em ambos os sexos após ICP primária.^269^ A Figura 10.4 apresenta a seleção da estratégia de reperfusão com os tempos que devem ser buscados para a melhora dos resultados no atendimento do IAMCSST.^271^

**10.4.3.1. Estratégias na Abordagem da Doença Coronariana Múltipla**

Os pacientes que mais se beneficiam da revascularização de artérias não infartadas incluem aqueles com grande área de miocárdio em risco e aqueles sem comorbidades significativas que aumentariam o risco de RVM. Os dados do estudo COMPLETE apoiam a RVM completa e o tratamento das lesões não culpadas no momento da ICP primária para IAMCSST ou como um procedimento estagiado. No entanto, as análises de subgrupos desse estudo demonstraram uma tendência de modificação do efeito por sexo (p interação = 0,08), com as mulheres não apresentando nenhum benefício de mortalidade cardíaca com a RVM completa.^272^

**10.4.3.2. Choque Cardiogênico**

Choque cardiogênico, IC e infarto do ventrículo direito ocorrem com mais frequência em mulheres com IAMCSST do que em homens. As mulheres também têm PA e débito cardíaco mais baixos em comparação aos homens em situação de choque cardiogênico.^65^Contudo, vários estudos, incluindo o CULPRIT-SHOCK, mostraram que o sexo não influenciou a mortalidade de acordo com as diferentes estratégias de RVM.^273^

### 10.4.4. Considerações sobre o Dispositivo durante a Revascularização Percutânea

**10.4.4.1. Stents Farmacológicos**

Nas últimas duas décadas, houve importante evolução na tecnologia dos *stents* farmacológicos, incluindo a otimização de drogas, polímeros e desenho dos *stents*, o que deu suporte à segurança e à eficácia dos *stents* farmacológicos mais recentes. Estudos angiográficos de *stents* farmacológicos avaliando a perda tardia do lúmen demonstraram graus semelhantes de hiperplasia neointimal em mulheres e homens, sugerindo que os perfis de eluição de drogas são eficazes em ambos os sexos.^274^

**10.4.4.2. Balão Farmacológico**

A ICP com *stent* farmacológico é o modo mais comum de revascularização para DIC. No entanto, a reestenose intra-*stent* e vasos finos são limitações conhecidas. O balão farmacológico consiste em um balão revestido com agentes antiproliferativos encapsulados em uma matriz polimérica, que são liberados na parede durante a insuflação e contato com a íntima. O fato de não deixar metal no vaso tratado traz benefícios para vasos finos, reestenose intra-*stent* e vasos médios e distais, onde *stents* distais podem ser uma desvantagem para futuras RVM.^275-277^

**10.4.4.3. Aterectomia Rotacional e Litotripsia Intravascular**

A aterectomia rotacional e a litotripsia intravascular estão indicadas em lesões calcificadas para prevenir a subexpansão do *stent*, que é associada a taxas mais altas de reestenose. Nas mulheres, a aterectomia rotacional está associada a maior risco de complicações periprocedimento, mas com taxas de sobrevida global ajustadas a longo prazo livre de MACE semelhantes às dos homens. No entanto, a litotripsia intravascular apresenta complicações angiográficas, segurança e eficácia semelhantes em mulheres e homens.^65,278^

## 10.5. Terapia Farmacológica Adjunta

Embora estudos randomizados sugiram benefícios semelhantes da terapia antitrombótica adjunta para doença coronariana em ambos os sexos, mulheres recebem menos frequentemente o tratamento proposto nas diretrizes.^269,279,280^ Vulnerabilidade para sangramento é uma das razões. Mulheres, particularmente idosas e de baixo peso, apresentam maior risco de complicações hemorrágicas, que podem estar relacionadas a menor volume de distribuição corporal, menores taxas de filtração glomerular e diferenças nas atividades das enzimas hepáticas.^269^ No sentido de mitigar esse risco, além do acesso radial, é importante atentar para o ajuste nas doses de anticoagulantes e de antiplaquetários para peso e função renal, sempre que pertinente, além de monitorar o tempo de coagulação ativado a cada 30-60 minutos durante a intervenção, administrando doses suplementares de heparina, se necessário, para assegurar níveis de anticoagulação adequados.^281^

Quanto à terapia antiplaquetária oral, a dupla antiagregação plaquetária com ácido acetilsalicílico e um inibidor P2Y12 (clopidogrel, prasugrel ou ticagrelor) é recomendada para prevenção de eventos trombóticos pós-intervenção.^155^ Apresentação clínica, risco de sangramento, risco isquêmico e comorbidades devem orientar a seleção do tipo de inibidor P2Y12, o momento ideal para administrar sua dose de ataque, bem como a duração da dupla antiagregação plaquetária, independentemente de sexo.^155,281^ Mulheres, entretanto, apresentam risco mais elevado de sangramento com qualquer dos inibidores.^282,283^ Estratégias para minimizar esse risco incluem: encurtar a duração da dupla antiagregação plaquetária; descontinuar o ácido acetilsalicílico, mantendo monoterapia com inibidor P2Y12 após curto período de dupla antiagregação plaquetária; ou descalonar o inibidor P2Y12, de prasugrel ou ticagrelor para clopidogrel.^284,285^ A suspensão do ácido acetilsalicílico após curto período peri-intervenção (na alta ou após uma semana) mostrou-se efetiva para reduzir sangramentos em portadores de FA com necessidade de anticoagulação oral, submetidos a ICP.^286^ Análises sexo-específicas da segurança e efetividade dessas estratégias poderão auxiliar na seleção ou personalização do regime antiplaquetário ideal para mulheres.

## 10.6. *Gaps* no Conhecimento

Prevalência de doença coronariana obstrutiva e não obstrutiva no sexo feminino, em diferentes cenários clínicos, agudo e crônico.
Validação clínica de limites de referência para os testes de avaliação fisiológica invasiva coronariana (hiperêmicos e não hiperêmicos) específicos para as mulheres, frente a diferenças em calibre coronário, massa miocárdica e resistência microvascular entre os sexos.
Impacto diferencial da utilização do método de imagem intravascular em mulheres, considerando seu papel diagnóstico nos casos de MINOCA e seu valor para otimização da ICP no sexo feminino, que tipicamente envolve vasos de menor calibre e risco mais elevado de sangramento.
Seleção do método ideal de RVM (percutâneo *versus* cirúrgico) em mulheres com lesão de TCE ou multiarterial, reavaliando a influência do sexo nos desfechos terapêuticos no cenário atual.
Otimização das estratégias diagnósticas e de tratamento para as diferentes etiologias de MINOCA (e diagnósticos diferenciais), avançando na compreensão fisiopatológica, identificação de FR sexo-específicos e de preditores de recorrência para DEAC e cardiomiopatia de Takotsubo.
Particularidades da ICP nas mulheres em cenários complexos como choque cardiogênico, lesões calcificadas, bifurcações e oclusões coronárias crônicas.

## 10.7. Recomendações

**Table:** 

Recomendação	Classe de recomendação	Nìvel de evidência	Referência
Em pacientes que necessitam de revascularização coronariana, as decisões de tratamento devem ser baseadas na indicação clínica, independentemente de sexo, raça ou etnia	I	B	155
Em pacientes com SCA submetidos a ICP, a abordagem radial é indicada preferencialmente à abordagem femoral para reduzir o risco de morte, complicações vasculares ou sangramento	I	A	155
Em pacientes com doença isquêmica estável submetidos a ICP, a abordagem radial é recomendada para reduzir o sangramento no local de acesso e as complicações vasculares	I	A	155
ICP guiada por FFR deve ser considerada nos pacientes com doença multiarterial submetido a ICP	IIa	B	271
A estratégia invasiva urgente/imediata está indicada em pacientes com SCASSST com angina refratária e/ou instabilidade hemodinâmica e/ou elétrica (sem comorbidades graves ou contraindicações para esses procedimentos).	I	A	65
No IAMCSST a terapia de reperfusão é indicada em todos os pacientes com início dos sintomas <12 h de duração e elevação persistente do segmento ST	I	A	271
Todos os pacientes devem ser estratificados e classificados em risco alto, intermediário ou baixo de sangramento.	I	B	65

## 11. Intervenção Cirúrgica, Transplante Cardíaco

### 11.1. Revascularização do Miocárdio

Diversos fatores, sejam de ordem epidemiológica, anatômica e/ou relacionados à técnica operatória, fazem com que os resultados da cirurgia de RVM em mulheres sejam menos favoráveis do que em homens.^287^Diversas séries demonstraram que, no momento da indicação cirúrgica, as mulheres tendem a apresentar perfil clínico de maior risco operatório, com maior incidência de comorbidades associadas, tais como HAS, DM, IC, SCA, além de maior grau de comprometimento da função respiratória.^288-291^

O’Connor *et al*. demonstraram que as artérias coronárias são diretamente proporcionais à superfície corpórea dos indivíduos e, em consequência, nas mulheres elas são habitualmente menores do que nos homens.^292^Desde a década de 80, as análises do *Coronary Artery Surgery Study* (CASS) demonstraram que o diâmetro das artérias coronárias tinha influência direta nos resultados imediatos da cirurgia de RVM.^293^

Quando estratificados por grupos, a mortalidade em pacientes com coronárias calibrosas (2,5 - 3,5mm) foi de 1,5%, aumentando para 4,6% naqueles com diâmetro intermediário (1,5 - 2,0mm) e chegando a 15,8% naqueles cujas artérias coronárias tinham diâmetro médio de 1mm.^292^ Além do menor diâmetro das artérias coronárias, a qualidade, a delicadeza e a fragilidade dos enxertos influenciam na decisão da tática operatória a ser empregada e aumentam a complexidade técnica das operações.^294^

Apesar de as diretrizes americanas de 2021 recomendarem o uso preferencial de enxertos arteriais,^155^ vários estudos têm demonstrado a subutilização desses enxertos em pacientes do sexo feminino.^295,296^ Em decorrência do menor calibre das artérias mamárias e dos enxertos de artéria radial, existe uma menor utilização de enxertos arteriais, que poderiam aumentar a proporção de vasos revascularizados com enxertos de maior perviabilidade tardia.^297^ As características menos favoráveis dos enxertos, tanto arteriais como venosos, nas mulheres podem levar a maior predisposição a trombose precoce e tendência a espasmos, aumentando a chance de resultados desfavoráveis.^292,298^ Da mesma forma, apesar de a importância da RVM completa em pacientes com lesões multiarteriais ser bem estabelecida, diversos estudos demonstraram que as mulheres acabam por receber menor número de enxertos e, portanto, ser incompletamente revascularizadas.^299^

Os benefícios da cirurgia de RVM sem a utilização de circulação extracorpórea (CEC) ainda são bastante controversos. Alguns estudos demonstram mortalidade semelhante entre os sexos e outros não confirmam esses achados.^300,301^ Porém, admite-se que, em pacientes com doença cerebrovascular mais avançada, disfunção renal e/ou respiratória, essa técnica possa ser mais segura. Entretanto, pelos aspectos anatômicos e técnicos já mencionados, a cirurgia de RVM sem CEC em mulheres é frequentemente mais desafiadora e problemática do que em homens.

Nos últimos anos, houve um aumento significativo no número de mulheres submetidas à cirurgia de RVM. No entanto, na maior parte dos estudos existentes, ainda se observa o predomínio do sexo masculino nas amostras analisadas. Por dados de literatura previamente conhecidos, sabe-se que, quando comparadas aos homens, as mulheres apresentam piores desfechos e prognóstico após a cirurgia de RVM. De acordo com Attia *et al.*, a sobrevida a longo prazo após a cirurgia de RVM é pior em mulheres do que em homens, mesmo após ajuste para diferenças nos FR.^288^

Análises prévias identificaram o sexo feminino como um FR independente de mortalidade tanto operatória como de longo prazo após cirurgia de RVM, mesmo após ajuste de variáveis, como idade mais avançada e aumento da prevalência de comorbidades.^290^ Tanto no estudo EXCEL quanto no estudo NOBLE*,* observou-se que as mulheres tinham maior prevalência de FR como DM, HAS e dislipidemia. No entanto, a complexidade anatômica de suas lesões coronarianas era menor.^302^

Vaccarino *et al*. realizaram uma meta-análise dos estudos relacionados com diversos aspectos da cirurgia de RVM: *ART TRIAL, CORONARY TRIAL, GOPCABE TRIAL* e *PREVENT TRIAL*. As pacientes após os primeiros 5 anos da cirurgia apresentaram piores desfechos cardíacos e cerebrovasculares, porém com mortalidade semelhante quando comparadas aos homens. Essas diferenças não são evidentes após os 75 anos de idade (a diferença desses resultados entre os sexos está inversamente associada à idade). Os estudos registraram que as mulheres apresentam uma recuperação mais difícil do que os homens no pós-operatório de cirurgia de RVM. Em 6 a 8 semanas de pós-operatório, as mulheres reportaram mais queixas físicas e efeitos colaterais do que os homens no mesmo período. Além disso, apresentavam mais baixa capacidade funcional, mais sintomas depressivos e foram duas vezes mais propensas a readmissão hospitalar. Essas diferenças permaneceram substanciais e estatisticamente significativas mesmo após a análise multivariada. Nesse estudo ainda foi observado que a cirurgia de RVM tem impacto muito maior no humor das mulheres do que no dos homens.^303^

Apesar das diferenças relacionadas ao gênero, as indicações para cirurgia de RVM estão bem estabelecidas em recomendações e diretrizes atuais.^155^ Cabe aos provedores de saúde conhecer as particularidades relacionadas ao sexo feminino, a fim de promover estratégias de cuidado diferenciadas, melhorando a assistência às mulheres submetidas a cirurgia de RVM.

Historicamente, mulheres apresentam pior evolução pós-operatória em comparação aos homens na cirurgia de RVM.^293,304,305^ Vários aspectos têm sido analisados na tentativa de identificação de fatores que impactam nos desfechos negativos. Dentre as diferenças não modificáveis, a menor superfície corpórea mais frequentemente encontrada no sexo feminino foi estudada e considerada como fator que pudesse contribuir de forma negativa nos resultados pós-cirúrgicos, sem que houvesse definição conclusiva.^293,306^ Uma característica anatômica importante refere-se aos aspectos próprios da circulação coronariana no sexo feminino, em que se observam artérias coronárias mais finas e de menor calibre, conferindo maior complexidade técnica na confecção de anastomoses e maior risco de oclusão precoce de enxertos.^293,302,307^

Em função do calibre, um número menor de artérias recebe enxertos, de forma que áreas miocárdicas permanecem desprotegidas, elevando o risco tanto de recorrência de eventos isquêmicos, quanto de disfunção ventricular e IC no pós-operatório a médio e longo prazo. O risco de infarto do miocárdio peroperatório e de oclusão precoce de enxertos contribui para mortalidade intra-hospitalar notadamente maior do que a observada nos homens, comprometendo também resultados tardios.^305^

Outro dado importante relacionado à evolução desfavorável pós-operatória diz respeito à utilização de enxertos arteriais. Constata-se menor uso desses condutos em mulheres.^307,308^ Mesmo a utilização da artéria torácica interna esquerda isolada para revascularizar a artéria descendente anterior, considerada padrão-ouro na cirurgia de RVM, ocorre com menor frequência em pacientes do sexo feminino.^305,306^ O benefício potencial dos enxertos arteriais pode ser perdido em pacientes de maior risco, hipótese que explicaria porque as mulheres que apresentam pior perfil de risco basal não receberiam tantos enxertos arteriais quanto os homens.^302^

Pacientes do sexo feminino encaminhadas para tratamento cirúrgico têm propensão a serem mais idosas, apresentarem comorbidades significativamente maiores, incluindo HAS, DM, hiperlipidemia, doença arterial e venosa periférica, e *status* clínico de doença coronariana mais avançada com angina instável, angina pós-infarto, IC e indicação de cirurgia de RVM em caráter de urgência.^309^

**Figura 11.1:**
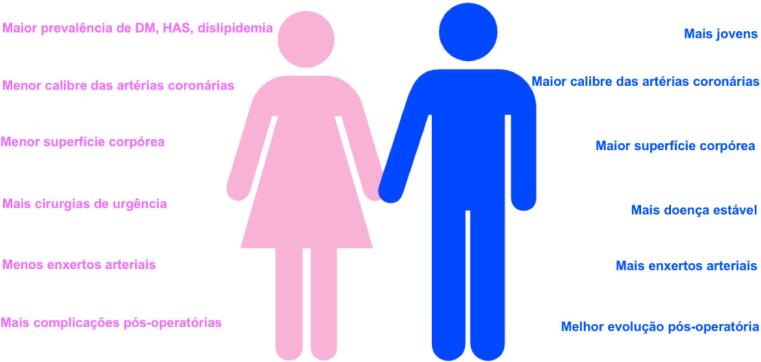
– Múltiplos fatores que influenciam os resultados pós-operatórios de mulheres e homens. DM: Diabetes mellitus; HAS: hipertensão arterial sistêmica.

Sendo assim, o diagnóstico da insuficiência coronariana, a indicação e o encaminhamento para tratamento cirúrgico ocorrem mais tardiamente em mulheres, com consequentes impactos negativos nos resultados pós-operatórios.^310^ As mulheres evoluem com complicações pós-operatórias imediatas mais frequentemente que os homens. Apenas as taxas de reoperação por sangramento são menores em pacientes do sexo feminino^311^ (Figura 11.1).

Com relação a aspectos técnicos, Puskas *et al.*^300^ descreveram que, na cirurgia de RVM sem CEC, os resultados pós-operatórios eram semelhantes em mulheres e homens, considerando que essa estratégia equiparava os desfechos entre os sexos. Observou que, especialmente em pacientes de maior risco, o sexo feminino não mostrou ser FR independente de mortalidade em pacientes submetidos a cirurgia de RVM sem CEC.

No entanto, de acordo com meta-análise de Gaudino *et al*.,^302^ variações na técnica cirúrgica utilizada nos procedimentos com o uso de CEC não melhoraram os resultados em mulheres, não havendo redução de mortalidade, considerando a diferença entre os sexos na cirurgia de RVM sem CEC.

Com relação às técnicas cirúrgicas utilizadas na cirurgia de RVM, as diretrizes internacionais recomendam fortemente o uso da artéria torácica interna esquerda para artéria descendente anterior esquerda, maior número de enxertos arteriais na doença isquêmica coronariana multiarterial e a cirurgia de RVM completa para a obtenção de melhores resultados pós-operatórios no curto e longo prazos após a cirurgia de RVM. Não obstante, mesmo em conformidade com as recomendações das diretrizes, as mulheres ainda estão sujeitas a chances menores, de 14% a 22%, de serem submetidas a cirurgia de RVM.^155,312-317^

#### 11.1.1. Cirurgia de Revascularização do Miocárdio em Mulheres - Recomendações

**Table:** 

Recomendação	Classe de recomendação	Nível de evidência	Referência
Revascularização completa com mínima manipulação da aorta	I	B	314,315
Utilização da artéria mamária esquerda para a artéria descendente anterior	I	B	316
Utilização da artéria radial como segunda opção para territórios com obstruções arteriais >80%	I	B	313
Esqueletonização na dissecção da artéria mamária para prevenção de infecção	I	B	317

## 11.2. Transplante Cardíaco

Vários são os fatores considerados para escolha do doador, como a concordância entre os sexos, tamanho e altura doador-receptor, IMC, hipertensão pulmonar e, mais recentemente, avaliação da massa cardíaca predita (pHM). É razoável usar o cálculo da pHM para ajudar na correspondência entre doador e receptor. Uma relação pHM doador/receptor de 0,86 ou superior prediz um bom prognóstico, entre 0,86 e 0,7 pode ser considerada para casos individuais, mas uma relação pHM inferior a 0,7 pode estar associada a resultados adversos pós-transplante.^318,319^ A correspondência de sexo doador-receptor atraiu considerável interesse recentemente.^320,321^ Ao longo dos anos, houve um ligeiro aumento nos transplantes de mulheres para mulheres e um declínio correspondente nos transplantes de mulheres para homens, talvez devido à sobrevida inferior observada após transplantes não concordantes por sexo. Isso pode ser explicado por diferenças imunológicas^291^ ou pela correspondência de tamanho do doador e do receptor.^320^

A distribuição por sexo dos receptores de transplante cardíaco vem mudando ao longo do tempo, com um aumento na proporção de receptores do sexo feminino de 19,3% de 1992 a 2000, para 22,4% de 2001 a 2009 e para 25,6% de 2010 a 2018. Não são claramente identificáveis os fatores pelos quais as mulheres têm reduzida representatividade entre os receptores de transplante cardíaco; um deles seria o desenvolvimento dos quadros de IC e consequente indicação de transplante em faixa etária mais avançada.^321-323^

As mulheres correm maior risco de sensibilização, sendo um dos principais FR a gravidez. Transfusões, dispositivos de assistência ventricular e transplante prévio são outros FR. Os anticorpos pré-formados podem causar rejeição hiperaguda e aumentar o risco de rejeição após o transplante, além de predisporem as pacientes ao desenvolvimento de vasculopatia do enxerto cardíaco.^324,325^ Precisamos detectar anticorpos anti-HLA para diminuir o risco de rejeição hiperaguda. A utilização do *crossmatch* virtual na avaliação pré-transplante, a partir de um banco de dados, substitui o *crossmatch* prospectivo e permite aumentar o *pool* de doadores e diminuir o tempo de teste, assim como decidir quais pacientes sensibilizados requerem tratamento antes do procedimento.^324-326^

**Figura 11.2:**
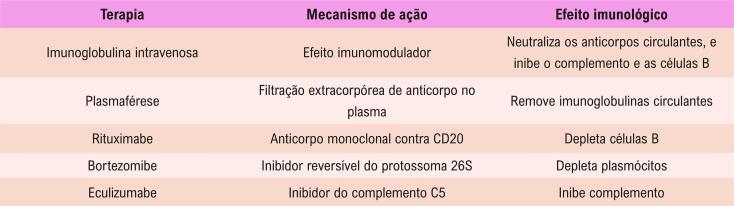
– Opções terapêuticas para dessensibilização e aumento do número de doadores. Fonte: Adaptado de Bacal F. 3ª Diretriz Brasileira de Transplante Cardíaco.*^327^*

O painel de reatividade de anticorpos deve ser realizado em todos os candidatos a transplante. Quando o painel de reatividade de anticorpos está elevado (≥ 10%), uma avaliação adicional é recomendada.^319^ Pode ser feita a dessensibilização dos receptores previamente ao transplante, com infusão venosa de imunoglobulina, plasmaférese isolada ou combinada, rituximabe e, em casos muito selecionados, esplenectomia.^319^ Tendo como alvo os componentes da resposta humoral, a dessensibilização pode ser usada como uma opção terapêutica para aumentar o número de doadores e permitir maior chance do transplante em pacientes sensibilizados (Figura 11.2).

O tratamento da rejeição hiperaguda deve ser iniciado imediatamente após o diagnóstico, mesmo se ainda na sala de cirurgia. Os tratamentos considerados incluem, de acordo com as diretrizes atuais da *International Society for the Heart and Lung Transplantation (ISHLT*):^319^ (1) suporte circulatório mecânico; (2) alta dose venosa de corticosteroides; (3) plasmaférese; (4) imunoglobulinas venosas; (5) rituximabe (6) terapia imunossupressora citolítica; (7) eculizumabe; (8) ciclosporina, tacrolimus com níveis-alvo aumentados e inibidores do ciclo metabólico (micofenolato); (9) inotrópicos e vasopressores venosos; (10) heparina.

A doença vascular do enxerto permanece altamente prevalente e uma das principais causas de morte tardia após o transplante cardíaco.^319^ Rejeição celular aguda grave (ISHLT 3R) diagnosticada por biópsia de endomiocárdio deve ser tratada mesmo na ausência de sintomas ou evidência de disfunção do aloenxerto cardíaco. Os FR significativos para o desenvolvimento de doença vascular do enxerto incluem morte do doador devido a AVC e diagnósticos do receptor de CMI e retransplante. Foi observado que o sexo feminino, tanto do doador quanto do receptor, representou um risco reduzido para desenvolvimento de doença vascular do enxerto.^328^ Estudos anteriores discordaram e mostraram uma maior incidência de desenvolvimento de doença vascular do enxerto em doadoras e receptoras do sexo feminino ou nenhuma diferença baseada no sexo para desenvolvimento de doença vascular do enxerto.^329-331^

Transplante cardíaco para miocardite periparto permanece relativamente infrequente. Alguns estudos demonstram que tardiamente a incidência de doença vascular do enxerto é semelhante à dos transplantes realizados em mulheres por outras etiologias.^332^

A malignidade após o transplante cardíaco continua sendo uma causa significativa de morbidade e mortalidade em receptores. Os dados do registro da *ISHLT* demonstram uma prevalência cumulativa de todos os tipos de malignidade após transplante cardíaco em adultos de 16% em 5 anos e 28% em 10 anos. O câncer de pele continua sendo a malignidade pós-transplante mais comum.^328^ Rudasill *et al.*, em recente análise do registro *United Network for Organ Sharing (UNOS),* não mostraram associação de malignidade do doador com a sobrevida do receptor em 10 anos.^333^ No entanto, um histórico de malignidade pré-transplante no receptor foi associado a um risco aumentado de malignidade pós-transplante, especialmente de neoplasias cutâneas. Houve, no entanto, incidência menor nas mulheres. Deve-se ter rigorosa vigilância relativa ao rastreamento de câncer de pele, mama na população feminina e colón na população geral.^334^

De acordo com o relatório de Transplante de Coração em Adultos da ISHLT de 2021, doadores-receptores do sexo feminino aumentaram a mortalidade em 1 ano em comparação com a combinação doador-receptor do sexo masculino (HR 1,16). Quando há discordância masculino /feminino há aumento da mortalidade em 5 anos (HR 1,1). Em contraste, a combinação doadora-receptora do sexo feminino está associada a um risco menor (HR 0,90) em comparação ao masculino/masculino em 5 anos.^323^ Além da incompatibilidade de tamanho, essas diferenças relacionadas ao sexo na sobrevida sugerem que as influências hormonais na resposta imunológica também possam estar associadas.^319^ A cardiomiopatia hipertrófica e a cardiopatia congênita foram associadas ao aumento da mortalidade em 1 ano. Outras variáveis associadas ao aumento da mortalidade em 5 anos, condicionada à sobrevida de 1 ano, incluem cardiomiopatia restritiva, isquêmica e retransplante.

De maneira indistinta entre os gêneros, painel de reatividade de anticorpos do receptor e tempo de isquemia estão associados a mortalidade em 1 ano, enquanto as características crônicas do receptor estão associadas a mortalidade em 5 anos. Portanto, sequelas crônicas do DM, doença vascular e doença renal crônica podem afetar os resultados pós-transplante de longo prazo. Outras variáveis associadas à mortalidade em 5 anos, condicionadas à sobrevida em 1 ano, incluem idade do receptor, IMC, resistência vascular pulmonar, função renal, idade do doador e volume de cirurgias do centro de transplante.^323^

O gênero feminino é um forte preditor de mortalidade em lista de espera para transplante cardíaco.^326-335^ No entanto, sexo não foi demonstrado como uma variável significativa ou importante associada à mortalidade pós-transplante, com poucas interações do gênero como preditor de mortalidade, apesar de as mulheres viverem mais que os homens.^336^

Apesar do aumento do número de mulheres submetidas a transplante cardíaco, elas continuam sub-representadas. Receptores do sexo feminino têm características basais diferentes quando comparados com os do sexo masculino destinatários, e a sobrevida pós-transplante é equivalente entre mulheres e homens após ajuste para destinatário e características do doador.^321^

As mulheres tendem a ter melhor sobrevida em longo prazo do que os homens após o transplante cardíaco, com menor risco de desenvolver doença arterial coronária ou vasculopatia do enxerto e aparecimento de tumores malignos, mas com maior risco de rejeição mediada por anticorpos.^330^

A mortalidade pós-transplante precoce (< 1 ano) deve-se principalmente à falha do enxerto, infecção e falência de múltiplos órgãos, enquanto a mortalidade pós-transplante tardia (> 5 anos) deve-se principalmente a malignidade, falha do enxerto e doença vascular do enxerto.^330^

A sobrevida global é enfatizada como um desfecho primário na evolução após transplante cardíaco. Entretanto, a qualidade de vida relacionada à saúde é um ponto importante nos transplantados cardíacos. Pesquisa recente utilizando o formulário resumido de 36 itens (SF-36) para avaliação da qualidade de vida, em concordância com a Organização Mundial da Saúde, demonstrou que 22% dos pacientes sobreviventes exerciam atividade laborativa após 1 ano e quase 33% trabalhavam após 2 anos. As complicações neurológicas e nefrológicas têm impacto negativo e são importantes preditores da qualidade de vida após o transplante cardíaco. O gênero é um dos fatores relacionados aos determinantes sociais da saúde, assim como raça e etnia, condição socioeconômica e nível educacional.^337-339^

Na coorte do estudo para desenvolver e validar modelos de *“machine learning”* para aumentar a precisão preditiva da mortalidade após transplante cardíaco, que compreendeu 18.625 pacientes, os modelos demonstraram boa precisão preditiva dos resultados após o transplante cardíaco, sendo um desses modelos o escore do *Index for Mortality Prediction after Cardiac Transplantation* (IMPACT). Entretanto, somente 27% dos pacientes eram do gênero feminino.^340^

A rejeição humoral crônica e a doença vascular do enxerto são as principais causas de morbimortalidade tardia de pacientes submetidos a transplante cardíaco, tendo o diagnóstico por métodos não invasivos baixa sensibilidade.^327^

### 11.2.1. Transplante Cardíaco em Mulheres – Recomendações

As recomendações para transplante não diferem entre os sexos, devendo-se seguir as recomendações da diretriz para transplante cardíaco.^327^

## 12. Reabilitação na Cardiomiopatia Isquêmica das Mulheres

É consenso entre diretrizes internacionais^341^ e nacionais^95^ a indicação de reabilitação cardíaca na DIC. Após SCA, RVM percutânea ou cirúrgica e na angina estável, o encaminhamento para reabilitação cardíaca deve fazer parte da prescrição médica. Está bem estabelecida eficácia na melhora da qualidade de vida, redução dos FR modificáveis e mortalidade, além da prevenção em readmissões por novos eventos.^342^

Estudos mostram que mulheres são menos aderentes que homens. Em amostra de 44% de mulheres, apenas 14,3% participaram da reabilitação cardíaca após IAM, comparado a 22,1% dos homens.^343^ O encaminhamento para reabilitação cardíaca na alta hospitalar tem se relacionado com menor mortalidade, especialmente em mulheres e minorias étnicas.^344^ Mulheres apresentam mais chances de óbito, IC ou AVC do que homens dentro dos 5 anos após IAM, independentemente da idade.^345^

Uma das principais barreiras para participação em reabilitação cardíaca é a ausência de encaminhamento médico. Em mulheres, o encaminhamento e reforço pelo médico sobre a importância da reabilitação cardíaca como parte do tratamento é forte preditor da admissão nos programas.^346^ Outras importantes barreiras são suporte social, baixa capacidade funcional, desemprego, idade mais avançada e medo de exercício físico, responsabilidades concorrentes de cuidados com a família, múltiplas comorbidades, ausência de reembolso para as sessões, limitada acessibilidade e diversidade nos programas.^344,347^

Durante avaliação inicial para admissão no programa de reabilitação cardíaca é necessário que sejam interrogadas questões específicas da mulher e que estão relacionadas com DCV nessa população. Pré-eclâmpsia, DM e hipertensão durante a gestação, nascimento pré-termo e menopausa precoce são alguns dos FR adicionais importantes de serem abordados, assim como sintomas de menopausa, presença de incontinência urinária, questões musculoesqueléticas e percepção de fadiga.^348^

**Figura 12.1:**
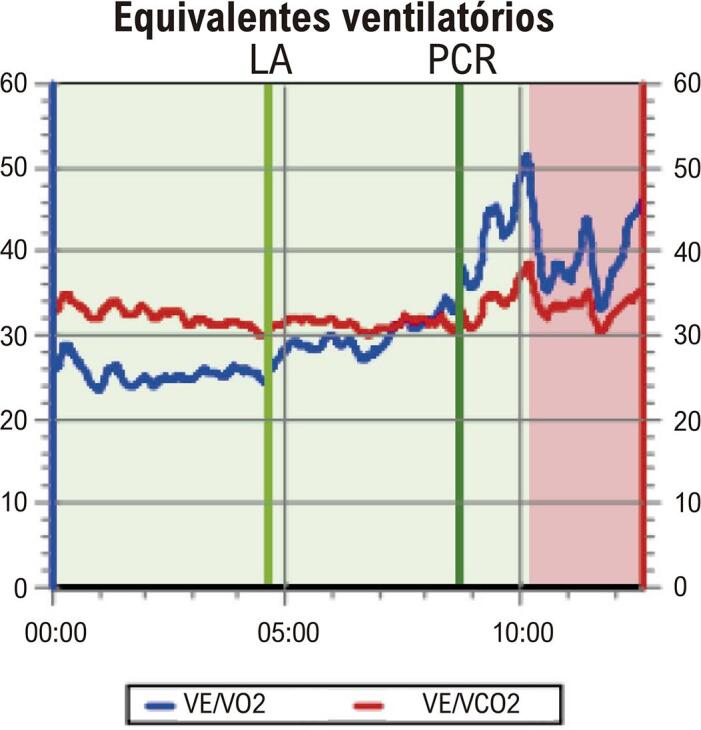
– Prescrição dos exercícios físicos aeróbicos baseada nos limiares ventilatórios 1 e 2 do TCPE. LA: limiar anaeróbico; PCR: ponto de compensação respiratória.

Para maior individualização e otimização da prescrição do exercício físico aeróbico indica-se, idealmente, o TCPE, com a detecção dos limiares ventilatórios sendo os limites de intensidade a serem utilizados (Figura 12.1), na ausência de isquemia e/ou alterações de outros indicadores, como platô precoce ou queda do PO_2_. Quando essas variáveis e alterações do segmento ST estiverem presentes, deverão ser consideradas na prescrição, que deverá se manter abaixo do limiar isquêmico. Caso não haja disponibilidade do TCPE, percentuais da FC pico preditos ou medidos por TE, teste de fala (*talk test*) e percepção subjetiva de esforço podem auxiliar na prescrição da intensidade do exercício físico (Tabela 12.1).^349^ Objetiva-se em linhas gerais a intensidade moderada com impacto em mortalidade e qualidade de vida, mas a prescrição será sempre individualizada (Tabela 12.2).

**Tabela 12.1:** – Métodos alternativos de prescrição de exercícios físicos aeróbicos.

MÉTODO	DESCRIÇÃO
Sensação subjetiva de esforço (Escala de Borg)	Exercícios com autopercepção de esforço
Teste de fala (*Talk test*)	Execução dos exercícios na intensidade em que a respiração seja ofegante, porém controlada, de modo que se consiga completar uma frase sem pausas (intensidade moderada)
Percentuais da FCMprev	Exercícios baseados no percentual da FCMprev FC alvo = percentual × FCMprev $\mathrm{FCMprev}=208-(0.7x\text{ idade }{)}^{*}\text{ ou }220-{\text{ idade }}^{**}$
Frequência cardíaca de reserva (*Karvonen*)	FC alvo =FC repouso +%× (FCpico − FCrepouso )

FC: frequência cardíaca; FCMprev: FC máxima prevista; FCR: FC de reserva; *Fórmula de Tanaka, **Fórmula de Karvonen.^*349*^

**Tabela 12.2:** – Níveis de intensidade de exercícios aeróbicos.

Intensidade	%VO_**2**_ máx	%FCMprev	%FCR	MET^*^ (absoluto)	Escala de Borg
Muito leve	< 37	< 57	< 30	< 2	< 9
Leve	37-45	57-64	30-40	2-3,9	9-11
Moderado	46-64	65-76	40-60	4-6	12-13
Intenso	65-91	76-96	60-90	6,1-8,8	14-17
Muito intenso	> 91	> 96	> 90	> 8,9	> 17

Modificado de ACSM.10 *MET: unidade metabólica basal; %VO2 máx: valores percentuais do consumo máximo de oxigênio; %FCM prev: valores percentuais da frequência cardíaca máxima prevista para a idade; %FCR: valores percentuais da frequência cardíaca de reserva; Escala de Borg: escala linear de percepção do esforço, graduação de 6 a 20.

Particularmente, as mulheres têm ganhos menores na capacidade funcional após programas de reabilitação cardíaca, independentemente de menor capacidade funcional inicial. Ajustes na prescrição se fazem, então, necessários no decorrer do programa, já que a melhora da capacidade funcional é fator primordial para efetividade do programa em relação aos seus benefícios.^347^

Paralelamente à capacidade funcional, existe associação da redução da força muscular com aumento da DCV e mortalidade. Mulheres apresentam menor força pelo teste de preensão palmar. Khadanga *et al*.^347^ reforçam que é importante foco adicional na prescrição de treino resistido, com intensidade de até 80% de 1 repetição máxima e objetivo de melhora da força de membros inferiores, capacidade de caminhada e ganho para o alcance das demandas de atividades da vida diária. Sugerem, ainda, inclusão de treinos aeróbicos intervalados de alta intensidade (90-95% da FCpico) baseado em pequeno estudo randomizado de seu grupo, onde, comparado ao treino moderado (70-85% da FCpico), o VO_2_ apresentou maior aumento (23% *versus* 7%).^348-350^ Entretanto, mais estudos são necessários nessa área para confirmar esses achados. Mulheres são mais propensas a dor musculoesquelética e fadiga. Traçar um programa com objetivos compartilhados e oferecer métodos de exercícios complementares como Yoga, dança, Tai-Chi podem evitar a desistência dos programas. Contudo, estudos precisam ser realizados para comprovar a efetividade desses métodos na redução de RCV, morbidade e mortalidade.^348^

Outras situações mais prevalentes na população feminina precisam ser citadas quando abordamos a reabilitação cardíaca e o seu papel na DIC:

**Dissecção espontânea coronariana**: A ausência de consenso sobre exercício após essa condição e sua possível relação com esforços intensos causa insegurança entre profissionais de saúde e pacientes para o retorno às atividades físicas. Entretanto, em registro da *Mayo Clinic* com 354 pacientes com DEAC, onde 96% da população era composta por mulheres, benefícios da participação em reabilitação cardíaca foram confirmados, sem complicações clínicas.^351^ Programa direcionado para DEAC foi desenvolvido no *Vancouver General Hospital,*^352^ com melhora na capacidade funcional, no estresse psicológico e na redução da necessidade de RVM durante o acompanhamento. Portanto, o encaminhamento a centros de reabilitação cardíaca deve ser incentivado, inclusive para reduzir o medo do exercício que muitas pacientes apresentam após episódio de DEAC.

**MINOCA**: Diferentemente do consenso inequívoco do papel da reabilitação cardíaca na doença coronariana obstrutiva, dados de eficácia e segurança na disfunção microvascular coronariana são escassos. Entretanto, estudos na área realizados em mulheres com angina microvascular já foram capazes de demonstrar melhora na capacidade funcional, em variáveis metabólicas, qualidade de vida e perfusão miocárdica, após diferentes períodos de um programa de reabilitação cardíaca e devem ser estimulados.^353,354^
