# Supplementary material for: Posicionamento sobre Doença Isquêmica do Coração – A Mulher no Centro do Cuidado – 2023
Source: Arq Bras Cardiol. 2023 Jul 27;120(7):e20230303. [Article in Portuguese] doi: 10.36660/abc.20230303 (PMC10382148; doi:10.36660/abc.20230303)
Supplement: Translation

# Translation: Position Statement on Ischemic Heart Disease – Women-Centered Health Care – 2023

**Authors:** Gláucia Maria Moraes de Oliveira, Maria Cristina Costa de Almeida, Daniela do Carmo Rassi, Érika Olivier Vilela Bragança, Lidia Zytynski Moura, Magaly Arrais, Milena dos Santos Barros Campos, Viviana Guzzo Lemke, Walkiria Samuel Avila, Alexandre Jorge Gomes de Lucena, André Luiz Cerqueira de Almeida, Andréa Araujo Brandão, Andrea Dumsch de Aragon Ferreira, Andreia Biolo, Ariane Vieira Scarlatelli Macedo, Breno de Alencar Araripe Falcão, Carisi Anne Polanczyk, Carla Janice Baister Lantieri, Celi Marques-Santos, Claudia Maria Vilas Freire, Denise Pellegrini, Elizabeth Regina Giunco Alexandre, Fabiana Goulart Marcondes Braga, Fabiana Michelle Feitosa de Oliveira, Fatima Dumas Cintra, Isabela Bispo Santos da Silva Costa, José Sérgio Nascimento Silva, Lara Terra F. Carreira, Lucelia Batista Neves Cunha Magalhães, Luciana Diniz Nagem Janot de Matos, Marcelo Heitor Vieira Assad, Marcia M. Barbosa, Marconi Gomes da Silva, Maria Alayde Mendonça Rivera, Maria Cristina de Oliveira Izar, Maria Elizabeth Navegantes Caetano Costa, Maria Sanali Moura de Oliveira Paiva, Marildes Luiza de Castro, Marly Uellendahl, Mucio Tavares de Oliveira, Olga Ferreira de Souza, Ricardo Alves da Costa, Ricardo Quental Coutinho, Sheyla Cristina Tonheiro Ferro da Silva, Sílvia Marinho Martins, Simone Cristina Soares Brandão, Susimeire Buglia, Tatiana Maia Jorge de Ulhôa Barbosa, Thais Aguiar do Nascimento, Thais Vieira, Valquíria Pelisser Campagnucci, Antonio Carlos Palandri Chagas

**Affiliations:** 1 Brazil Universidade Federal do Rio de Janeiro (UFRJ),Rio de Janeiro, RJ – Brazil; 2 Brazil Centro Universitário de Belo Horizonte, Belo Horizonte, MG – Brazil; 3 Brazil Faculdade de Medicina da Universidade Federal de Goiás (UFG), Goiânia, GO – Brazil; 4 Brazil RitmoCheck, São José dos Campos, SP – Brazil; 5 Brazil Pontifícia Universidade Católica do Paraná (PUC-PR), Curitiba, PR – Brazil; 6 Brazil Hospital do Coração (HCor), São Paulo, SP – Brazil; 7 Brazil Hospital Universitário de Sergipe, Aracaju, SE – Brazil; 8 Brazil Cardiocare – Clínica Cardiológica Ltda., Curitiba, PR – Brazil; 9 Brazil Instituto do Coração (Incor) do Hospital das Clínicas da Faculdade de Medicina da Universidade de São Paulo (FMUSP) São Paulo, SP – Brazil; 10 Brazil Hospital Agamenon Magalhães, Recife, PE – Brazil; 11 Brazil Santa Casa de Misericórdia de Feira de Santana, Feira de Santana, BA – Brazil; 12 Brazil Universidade do Estado do Rio de Janeiro (UERJ), Rio de Janeiro, RJ – Brazil; 13 Brazil Instituto de Neuro e Cardiologia de Curitiba (INC), Curitiba, PR – Brazil; 14 Brazil Universidade Federal do Rio Grande do Sul (UFRGS), Porto Alegre, RS – Brazil; 15 Brazil Santa Casa de Misericórdia de São Paulo, São Paulo, SP – Brazil; 16 Brazil Hospital de Messejana, Fortaleza, CE – Brazil; 17 Brazil Hospital de Clínicas da UFRGS, Porto Alegre, RS – Brazil; 18 Brazil Centro Universitário Faculdade de Medicina ABC, Santo André, SP – Brazil; 19 Brazil Universidade Tiradentes (UNIT), Aracaju, SE – Brazil; 20 Brazil Hospital São Lucas Rede D’Or São Luis, Aracaju, SE – Brazil; 21 Brazil Empresa Brasileira de Serviços Hospitalares (EBSERH), Belo Horizonte, MG – Brazil; 22 Brazil Hospital São Lucas da Pontifícia Universidade Católica do Rio Grande do Sul (PUC-RS), Porto Alegre, RS – Brazil; 23 Brazil Instituto do Coração de Pernambuco, Recife, PE – Brazil; 24 Brazil Universidade Federal de São Paulo (UNIFESP), São Paulo, SP – Brazil; 25 Brazil Instituto do Câncer do Estado de São Paulo, São Paulo, SP – Brazil; 26 Brazil Pronto Socorro Cardiológico de Pernambuco da Universidade de Pernambuco (PROCAPE/UPE), Recife, PE – Brazil; 27 Brazil Cardiologia Nuclear de Curitiba, Curitiba, PR – Brazil; 28 Brazil Hospital Pilar, Curitiba, PR – Brazil; 29 Brazil Faculdade de Medicina da Universidade Federal da Bahia (UFBA), Salvador, BA – Brazil; 30 Brazil Hospital Israelita Albert Einstein, São Paulo SP – Brazil; 31 Brazil Instituto Nacional de Cardiologia (INC), Rio de Janeiro, RJ – Brazil; 32 Brazil Hospital Socor, Belo Horizonte, MG – Brazil; 33 Brazil SPORTIF – Clínica do Exercício e do Esporte, Belo Horizonte, MG – Brazil; 34 Brazil Universidade Federal de Alagoas (UFAL), Maceió, AL – Brazil; 35 Brazil Centro Universitário do Estado Pará (CESUPA), Belém, PA – Brazil; 36 Brazil INTERVE, Natal, RN – Brazil; 37 Brazil Faculdade IPEMED de Ciências Médicas, Belo Horizonte, MG – Brazil; 38 Brazil DASA – Diagnósticos da América S/A, São Paulo, SP – Brazil; 39 Brazil Rede D’Or, Rio de Janeiro, RJ – Brazil; 40 Brazil Instituto Dante Pazzanese de Cardiologia, São Paulo, SP – Brazil; 41 Brazil Faculdade de Ciências Médicas da Universidade de Pernambuco (UPE), Recife, PE – Brazil; 42 Brazil Hospital Universitário Osvaldo Cruz da Universidade de Pernambuco (UPE), Recife, PE – Brazil; 43 Brazil CEMISE Oncoclínicas, Aracaju, SE – Brazil; 44 Brazil Hospital das Clínicas da Universidade Federal de Pernambuco (UFPE), Recife, PE – Brazil; 45 Brazil CARDIOCENTRO Cirurgia Cardiovascular, Brasília, DF – Brazil; 46 Brazil Hospital de Base do Distrito Federal, Brasília, DF – Brazil; 47 Brazil Cardio Ritmo Serviços Médicos, Salvador, BA – Brazil; 48 Brazil Rede D’Or, Aracaju, SE – Brazil; 49 Brazil Hospital Universitário da Universidade Federal de Sergipe (UFS), Aracaju, SE – Brazil; 50 Brazil Irmandade da Santa Casa de São Paulo, São Paulo, SP – Brazil

**Table:** 

Position Statement on Ischemic Heart Disease – Women-Centered Health – 2023	
The report below lists declarations of interest as reported to the SBC by the experts during the period of the development of these statement, 2022/2023.	
Expert	Type of relationship with industry
Alexandre Jorge Gomes de Lucena	Financial declaration A - Economically relevant payments of any kind made to (i) you, (ii) your spouse/partner or any other person living with you, (iii) any legal person in which any of these is either a direct or indirect controlling owner, business partner, shareholder or participant; any payments received for lectures, lessons, training instruction, compensation, fees paid for participation in advisory boards, investigative boards or other committees, etc. From the brazilian or international pharmaceutical, orthosis, prosthesis, equipment and implants industry: - Cardiopapers; Afya.
André Luiz Cerqueira de Almeida	Financial declaration A - Economically relevant payments of any kind made to (i) you, (ii) your spouse/partner or any other person living with you, (iii) any legal person in which any of these is either a direct or indirect controlling owner, business partner, shareholder or participant; any payments received for lectures, lessons, training instruction, compensation, fees paid for participation in advisory boards, investigative boards or other committees, etc. From the brazilian or international pharmaceutical, orthosis, prosthesis, equipment and implants industry: - Boston Scientific: Speaker on Prosthesis.
Andréa Araujo Brandão	Financial declaration A - Economically relevant payments of any kind made to (i) you, (ii) your spouse/partner or any other person living with you, (iii) any legal person in which any of these is either a direct or indirect controlling owner, business partner, shareholder or participant; any payments received for lectures, lessons, training instruction, compensation, fees paid for participation in advisory boards, investigative boards or other committees, etc. From the brazilian or international pharmaceutical, orthosis, prosthesis, equipment and implants industry: - Servier: Triplixan; Daiichi Sankyo: Benicar; Libbs: Venzer. B - Research funding under your direct/personal responsibility (directed to the department or institution) from the Brazilian or international pharmaceutical, orthosis, prosthesis, equipment and implants industry: - Servier: Hypertension. Other relationships Funding of continuing medical education activities, including travel, accommodation and registration in conferences and courses, from the brazilian or international pharmaceutical, orthosis, prosthesis, equipment and implants industry: - Servier: Hypertension.
Andrea Dumsch de Aragon Ferreira	Nothing to be declared
Andreia Biolo	Financial declaration B - Research funding under your direct/personal responsibility (directed to the department or institution) from the Brazilian or international pharmaceutical, orthosis, prosthesis, equipment and implants industry: - Alnylam Pharmaceuticals: Amyloidosis.
Antonio Carlos Palandri Chagas	Financial declaration A - Economically relevant payments of any kind made to (i) you, (ii) your spouse/partner or any other person living with you, (iii) any legal person in which any of these is either a direct or indirect controlling owner, business partner, shareholder or participant; any payments received for lectures, lessons, training instruction, compensation, fees paid for participation in advisory boards, investigative boards or other committees, etc. From the brazilian or international pharmaceutical, orthosis, prosthesis, equipment and implants industry: - Novo Nordisk; Viatris; Instituto Vita Nova. Outros relacionamentos Financiamento de atividades de educação médica continuada, incluindo viagens, hospedagens e inscrições para congressos e cursos, provenientes da indústria farmacêutica, de órteses, próteses, equipamentos e implantes, brasileiras ou estrangeiras: - Novo Nordisk.
Ariane Vieira Scarlatelli Macedo	Financial declaration A - Economically relevant payments of any kind made to (i) you, (ii) your spouse/partner or any other person living with you, (iii) any legal person in which any of these is either a direct or indirect controlling owner, business partner, shareholder or participant; any payments received for lectures, lessons, training instruction, compensation, fees paid for participation in advisory boards, investigative boards or other committees, etc. From the brazilian or international pharmaceutical, orthosis, prosthesis, equipment and implants industry: - Bayer: Anticoagulation and heart failure; Pfizer: Anticoagulation and Amyloidosis; Jannsen: Leukemia. Other relationships Funding of continuing medical education activities, including travel, accommodation and registration in conferences and courses, from the brazilian or international pharmaceutical, orthosis, prosthesis, equipment and implants industry: - Bayer: Heart failure.
Breno de Alencar Araripe Falcão	Financial declaration A - Economically relevant payments of any kind made to (i) you, (ii) your spouse/partner or any other person living with you, (iii) any legal person in which any of these is either a direct or indirect controlling owner, business partner, shareholder or participant; any payments received for lectures, lessons, training instruction, compensation, fees paid for participation in advisory boards, investigative boards or other committees, etc. From the brazilian or international pharmaceutical, orthosis, prosthesis, equipment and implants industry: - Edwards Lifescience: TAVI and TMVR proctor; Medtronic: TAVI proctor; Boston Scientific: CTO PCI proctor.
Carisi Anne Polanczyk	Nothing to be declared
Carla Janice Baister Lantieri	Nothing to be declared
Celi Marques-Santos	Nothing to be declared
Claudia Maria Vilas Freire	Nothing to be declared
Daniela do Carmo Rassi	Nothing to be declared
Denise Pellegrini	Nothing to be declared
Elizabeth Regina Giunco Alexandre	Financial declaration A - Economically relevant payments of any kind made to (i) you, (ii) your spouse/partner or any other person living with you, (iii) any legal person in which any of these is either a direct or indirect controlling owner, business partner, shareholder or participant; any payments received for lectures, lessons, training instruction, compensation, fees paid for participation in advisory boards, investigative boards or other committees, etc. From the brazilian or international pharmaceutical, orthosis, prosthesis, equipment and implants industry: - Lilly: Trulicity, Jardiance, Glyxambi. Other relationships Funding of continuing medical education activities, including travel, accommodation and registration in conferences and courses, from the brazilian or international pharmaceutical, orthosis, prosthesis, equipment and implants industry: - Novo Nordisk: Ozempic.
Érika Olivier Vilela Bragança	Nothing to be declared
Fabiana Goulart Marcondes Braga	Financial declaration A - Economically relevant payments of any kind made to (i) you, (ii) your spouse/partner or any other person living with you, (iii) any legal person in which any of these is either a direct or indirect controlling owner, business partner, shareholder or participant; any payments received for lectures, lessons, training instruction, compensation, fees paid for participation in advisory boards, investigative boards or other committees, etc. From the brazilian or international pharmaceutical, orthosis, prosthesis, equipment and implants industry: - Novartis: Lectures; AstraZeneca: Lectures and Advisory Board; Boehringer: Advisory Board.
Fabiana Michelle Feitosa de Oliveira	Nothing to be declared
Fatima Dumas Cintra	Nothing to be declared
Gláucia Maria Moraes de Oliveira	Nothing to be declared
Isabela Bispo Santos da Silva Costa	Nothing to be declared
José Sérgio Nascimento Silva	Nothing to be declared
Lara Terra F. Carreira	Nothing to be declared
Lidia Zytynski Moura	Financial declaration A - Economically relevant payments of any kind made to (i) you, (ii) your spouse/partner or any other person living with you, (iii) any legal person in which any of these is either a direct or indirect controlling owner, business partner, shareholder or participant; any payments received for lectures, lessons, training instruction, compensation, fees paid for participation in advisory boards, investigative boards or other committees, etc. From the brazilian or international pharmaceutical, orthosis, prosthesis, equipment and implants industry: - Novartis: Entresto; AstraZeneca: Forxiga; Boehringer: Jardiance; Bayer: Vericiguat; Vifor: Ferrinject. B - Research funding under your direct/personal responsibility (directed to the department or institution) from the Brazilian or international pharmaceutical, orthosis, prosthesis, equipment and implants industry: - Bayer: Vericiguat.
Lucelia Batista Neves Cunha Magalhães	Nothing to be declared
Luciana Diniz Nagem Janot de Matos	Nothing to be declared
Magaly Arrais	Financial declaration A - Economically relevant payments of any kind made to (i) you, (ii) your spouse/partner or any other person living with you, (iii) any legal person in which any of these is either a direct or indirect controlling owner, business partner, shareholder or participant; any payments received for lectures, lessons, training instruction, compensation, fees paid for participation in advisory boards, investigative boards or other committees, etc. From the brazilian or international pharmaceutical, orthosis, prosthesis, equipment and implants industry: - Edwards / Boston: Transcatheter valve implantation; Medtronic: Implant.
Marcelo Heitor Vieira Assad	Financial declaration A - Economically relevant payments of any kind made to (i) you, (ii) your spouse/partner or any other person living with you, (iii) any legal person in which any of these is either a direct or indirect controlling owner, business partner, shareholder or participant; any payments received for lectures, lessons, training instruction, compensation, fees paid for participation in advisory boards, investigative boards or other committees, etc. From the brazilian or international pharmaceutical, orthosis, prosthesis, equipment and implants industry: - Novo Nordisk: Semaglutida; AstraZeneca: Dapagliflozina; BI: Empagliflozina; GSK: Shingrix; Biolab: Evolucumabe; Daiichi Sankyo: Benicar Triplo; Novartis: Dyslipidemia. B - Research funding under your direct/personal responsibility (directed to the department or institution) from the Brazilian or international pharmaceutical, orthosis, prosthesis, equipment and implants industry: - Amgen: LP(A). Other relationships Funding of continuing medical education activities, including travel, accommodation and registration in conferences and courses, from the brazilian or international pharmaceutical, orthosis, prosthesis, equipment and implants industry: - Novo Nordisk: Semaglutina; BI: Empagliglozina.
Marcia M. Barbosa	Nothing to be declared
Marconi Gomes da Silva	Nothing to be declared
Maria Alayde Mendonça Rivera	Nothing to be declared
Maria Cristina Costa de Almeida	Nothing to be declared
Maria Cristina de Oliveira Izar	Financial declaration A - Economically relevant payments of any kind made to (i) you, (ii) your spouse/partner or any other person living with you, (iii) any legal person in which any of these is either a direct or indirect controlling owner, business partner, shareholder or participant; any payments received for lectures, lessons, training instruction, compensation, fees paid for participation in advisory boards, investigative boards or other committees, etc. From the brazilian or international pharmaceutical, orthosis, prosthesis, equipment and implants industry: - Amgen: Repatha; Amryt Pharma: Lojuxta; AstraZeneca: Dapagliflozina; Aché: Trezor, Trezete; Biolab: Livalo; Abbott: Lipidil; EMS: Rosuvastatina; Eurofarma: Rosuvastatina; Sanofi: Praluent, Zympass, Zympass Eze, Efluelda; Libbs: Plenance, Plenance Eze; Novo Nordisk: Ozempic, Victoza; Servier: Acertamlo, Alertalix; PTCBio: Waylivra. B - Research funding under your direct/personal responsibility (directed to the department or institution) from the Brazilian or international pharmaceutical, orthosis, prosthesis, equipment and implants industry: - PTCBio: Waylivra; Amgen: Repatha; Novartis: Inclisiran, Pelacarsen; NovoNordisk: Ziltivekimab. Other relationships Funding of continuing medical education activities, including travel, accommodation and registration in conferences and courses, from the brazilian or international pharmaceutical, orthosis, prosthesis, equipment and implants industry: - Novo Nordisk: Diabetes.
Maria Elizabeth Navegantes Caetano Costa	Financial declaration A - Economically relevant payments of any kind made to (i) you, (ii) your spouse/partner or any other person living with you, (iii) any legal person in which any of these is either a direct or indirect controlling owner, business partner, shareholder or participant; any payments received for lectures, lessons, training instruction, compensation, fees paid for participation in advisory boards, investigative boards or other committees, etc. From the brazilian or international pharmaceutical, orthosis, prosthesis, equipment and implants industry: - Libbs: Plenance Enze; Servier: Vastarel. Other relationships Funding of continuing medical education activities, including travel, accommodation and registration in conferences and courses, from the brazilian or international pharmaceutical, orthosis, prosthesis, equipment and implants industry: - Libbs; Servier: Congress participation.
Maria Sanali Moura de Oliveira Paiva	Nothing to be declared
Marildes Luiza de Castro	Financial declaration A - Economically relevant payments of any kind made to (i) you, (ii) your spouse/partner or any other person living with you, (iii) any legal person in which any of these is either a direct or indirect controlling owner, business partner, shareholder or participant; any payments received for lectures, lessons, training instruction, compensation, fees paid for participation in advisory boards, investigative boards or other committees, etc. From the brazilian or international pharmaceutical, orthosis, prosthesis, equipment and implants industry: - AstraZeneca: Forxiga/Heart failure; Servier: Acertil/Hypertension.
Marly Uellendahl	Financial declaration A - Economically relevant payments of any kind made to (i) you, (ii) your spouse/partner or any other person living with you, (iii) any legal person in which any of these is either a direct or indirect controlling owner, business partner, shareholder or participant; any payments received for lectures, lessons, training instruction, compensation, fees paid for participation in advisory boards, investigative boards or other committees, etc. From the brazilian or international pharmaceutical, orthosis, prosthesis, equipment and implants industry: - GE/Healthcare: Lectures and training in the field of Cardiovascular Magnetic Resonance.
Milena dos Santos Barros Campos	Nothing to be declared
Mucio Tavares de Oliveira Junior	Financial declaration A - Economically relevant payments of any kind made to (i) you, (ii) your spouse/partner or any other person living with you, (iii) any legal person in which any of these is either a direct or indirect controlling owner, business partner, shareholder or participant; any payments received for lectures, lessons, training instruction, compensation, fees paid for participation in advisory boards, investigative boards or other committees, etc. From the brazilian or international pharmaceutical, orthosis, prosthesis, equipment and implants industry: - Sanofi/Pasteur: Vaccines; AstraZeneca / Boehringer Ingelheim / Merck: Lectures; Novo Nordisk: Advisory board.
Olga Ferreira de Souza	Other relationships Funding of continuing medical education activities, including travel, accommodation and registration in conferences and courses, from the brazilian or international pharmaceutical, orthosis, prosthesis, equipment and implants industry: - Daiichi Sankyo.
Ricardo Alves da Costa	Nothing to be declared
Ricardo Quental Coutinho	Nothing to be declared
Sheyla Cristina Tonheiro Ferro da Silva	Financial declaration A - Economically relevant payments of any kind made to (i) you, (ii) your spouse/partner or any other person living with you, (iii) any legal person in which any of these is either a direct or indirect controlling owner, business partner, shareholder or participant; any payments received for lectures, lessons, training instruction, compensation, fees paid for participation in advisory boards, investigative boards or other committees, etc. From the brazilian or international pharmaceutical, orthosis, prosthesis, equipment and implants industry: - Palestras para Novartis: Entresto; AstraZeneca: Forxiga/ Xigduo; aliaça Boeringher-Lilly: Jardiance; Servier: Acertil, Acertalix, triplixan; Novo Nordisk: Saxenda, Ozempic, Rybelsus; Libbis: Naprix; Vifor: Ferinject. Other relationships Funding of continuing medical education activities, including travel, accommodation and registration in conferences and courses, from the brazilian or international pharmaceutical, orthosis, prosthesis, equipment and implants industry: - Aliança Boeringher-Lilly: Jardiance; Novonordisk: Ozempic, Rybelsus, Saxenda; Servier: Acertil, Acertalix, Triplixan.
Sílvia Marinho Martins	Nothing to be declared
Simone Cristina Soares Brandão	Nothing to be declared
Susimeire Buglia	Nothing to be declared
Tatiana Maia Jorge de Ulhôa Barbosa	Nothing to be declared
Thais Aguiar do Nascimento	Financial declaration A - Economically relevant payments of any kind made to (i) you, (ii) your spouse/partner or any other person living with you, (iii) any legal person in which any of these is either a direct or indirect controlling owner, business partner, shareholder or participant; any payments received for lectures, lessons, training instruction, compensation, fees paid for participation in advisory boards, investigative boards or other committees, etc. From the brazilian or international pharmaceutical, orthosis, prosthesis, equipment and implants industry: - Abbott: Consultancy.
Thais Vieira	Other relationships Funding of continuing medical education activities, including travel, accommodation and registration in conferences and courses, from the brazilian or international pharmaceutical, orthosis, prosthesis, equipment and implants industry: - Boehringer / AstraZeneca / Torrent / Novo Nordisk: Speaker.
Valquíria Pelisser Campagnucci	Nothing to be declared
Viviana Guzzo Lemke	Nothing to be declared
Walkiria Samuel Avila	Nothing to be declared

Table of contents

1. Introduction/Highlights 11

1.1. Introduction 11

1.2. Highlights of this Position Statement 12

**1.2.1. Epidemiology** 12

**1.2.2. Pathophysiological Bases of Atherothrombotic Disease** 12

**1.2.3. Clinical Presentation, Diagnosis, and Clinical Treatment** 12

**1.2.4. Diagnosis by use of Graphic Functional Assessment** 13

**1.2.5. Diagnosis by use of Noninvasive Cardiovascular Imaging** 13

**1.2.6. Arrhythmias in Ischemic Heart Disease** 13

**1.2.7. Atherothrombosis in Pregnancy, Contraception, Infertility, Antiphospholipid Syndrome** 13

**1.2.8. Ischemic Cardiomyopathy** 14

**1.2.9. Percutaneous Coronary Intervention** 14

**1.2.10. Myocardial Revascularization and Cardiac Transplantation** 14

**1.2.11. Rehabilitation in Ischemic Cardiomyopathy** 14

2. Epidemiology of Ischemic Heart Disease in Women 14

2.1. Introduction 14

2.2. Mortality 15

2.3. Prevalence and Incidence 20

2.4. Burden of Diseases 20

2.5. Risk Factors 21

2.6. Conclusion 22

3. Pathophysiological Bases of Atherothrombotic Disease 22

3.1. Introduction 22

3.2. Plaque Rupture 22

3.3. Spontaneous Coronary Artery Dissection 22

3.4. Coronary Artery Spasm 23

3.5. Coronary Microvascular Dysfunction 23

3.6. Embolism and Thrombosis 24

3.7. Takotsubo Syndrome 24

3.8. Myocarditis 24

4. Ischemic Heart Disease Clinical Presentation, Diagnosis, and Clinical Treatment 24

4.1. Chest Pain of Ischemic Etiology 24

4.2. Established and Modifiable Risk Factors 26

4.3. Established and Non-Modifiable Risk Factors 26

4.4. Women-Specific Risk Factors 27

4.5. Underrecognized Risk Factors 27

**4.5.1. Recommendations** 27

4.6. Drug Treatment in the Different Forms of Ischemia Manifestation 27

**4.6.1. Recommendations** 28

5. Diagnosis through Graphic Functional Assessment 29

5.1. Electrocardiogram at Rest 29

5.2. Exercise Testing 29

**5.2.1. Recommendations** 30

5.3. Cardiopulmonary Exercise Test 30

6. Diagnosis by Use of Ergometric-Functional Evaluation 31

6.1. Introduction 31

6.2. Echocardiography at Rest and Under Stress 31

6.3. Vascular Ultrasound 33

6.4. Computed Tomography 33

6.5. Cardiac Magnetic Resonance Imaging 34

6.6. Nuclear Medicine 34

7. Arrhythmias in Ischemic Cardiomyopathy 35

7.1. Atrial Fibrillation and Ischemic Heart Disease 35

7.2. Ventricular Arrhythmias: Sudden Death, Prevention, and Treatment 36

7.3. Cardiac Resynchronization Therapy 38

7.4. Recommendations 40

8. Atherothrombosis in Pregnancy, Contraception, Infertility, Antiphospholipid Syndrome 40

8.1. Introduction 40

8.2. Pregnancy 40

8.3. Contraception 41

**8.3.1. Recommendations** 42

8.4. Infertility 42

8.5. Antiphospholipid Syndrome 44

**8.5.1. Recommendations** 46

9. Ischemic Cardiomyopathy in Women 46

9.1. Introduction 46

9.2. Clinical Treatment 47

9.3. Devices and Advanced Heart Failure 47

9.4. Implantable Cardioverter Defibrillator 50

9.5. Advanced Heart Failure 50

9.6. Recommendations 50

**9.6.1. Clinical Management and Indications for Advanced Therapies** 50

10. Percutaneous Coronary Intervention 50

10.1. Introduction 50

10.2. Vascular Access to Cardiac Catheterization and Percutaneous Coronary Intervention in Women 50

10.3. Diagnosis 51

**10.3.1. Coronary Angiography** 51

**10.3.2. Intravascular Imaging** 51

**10.3.3. Invasive Tests with Measuring Guide** 51

***10.3.3.1. Fractional Flow Reserve*** 51

***10.3.3.2. Instantaneous Wave-free Pressure Ratio*** 52

**10.3.4. Functional Tests** 52

10.4. Percutaneous Treatment of Coronary Atherothrombotic Disease in Women 52

**10.4.1. Revascularization for Chronic Coronary Syndromes** 52

***10.4.1.1. Disease of the Left Main Coronary Artery*** 53

***10.4.1.2. Total Chronic Occlusion*** 54

**10.4.2. Revascularization for Non-ST-Elevation Myocardial Infarction** 54

**10.4.3. Revascularization for ST-Elevation Myocardial Infarction** 54

***10.4.3.1. Strategies to Approach Multivessel Coronary Artery Disease*** 55

***10.4.3.2. Cardiogenic Shock*** 55

**10.4.4. Considerations on Device During Percutaneous Revascularization** 55

***10.4.4.1. Drug-eluting Stents*** 55

***10.4.4.2. Drug-Coated Balloon*** 55

***10.4.4.3. Rotational Atherectomy and Intravascular Lithotripsy*** 56

10.5. Adjunct Pharmacological Therapy 56

10.6. Knowledge Gaps 56

10.7. Recommendations 56

11. Surgical Intervention, Heart Transplantation 57

11.1. Myocardial Revascularization 57

**11.1.1. Surgical Myocardial Revascularization in Women – Recommendations** 58

11.2. Heart Transplantation 58

**11.2.1. Heart Transplant in Women – Recommendations** 60

12. Rehabilitation in Women’s Ischemic Cardiomyopathy 60

References 62

## 1. Introduction/Highlights

### 1.1. Introduction

The differences between sexes go beyond the chromosomal differences between men (XY) and women (XX). Different social values, perceptions, and behaviors determine patterns and create different roles in society, which can generate differences in lifestyle and behavior that might influence epidemiology, clinical manifestation, and treatment.^1^

It is worth noting that, from the clinical viewpoint, ischemic heart disease (IHD) occurs earlier in men. However, its incidence and prevalence in women increase markedly after menopause. In addition, a higher proportion of women with anginal symptoms and acute coronary syndrome (ACS) have nonobstructive IHD.

In women, IHD includes classic coronary atherosclerosis, and its pathophysiology is varied, consisting of coronary microvascular dysfunction, endothelial dysfunction, vasomotor abnormalities, and spontaneous coronary artery dissection (SCAD).^2^

Regarding anatomy, women’s epicardial coronary arteries are smaller than those of men, even when adjusted for body surface and left ventricular (LV) mass. However, as compared to men, women have a lower prevalence of obstructive coronary atherosclerosis and different plaque characteristics even for comparable ischemia levels.^3^

Women with obstructive IHD are usually older than men, have more cardiovascular comorbidities and a higher incidence of adverse cardiovascular outcomes, such as mortality after acute myocardial infarction (AMI).^4^

In women, as compared to men, plaque rupture occurs less often, and culprit-artery revascularization can be more challenging because of access site bleeding and smaller and more tortuous coronary arteries.^5^

Chest pain is the most prevalent symptom of AMI in both sexes. However, women more often have atypical symptoms, such as pain in the upper back and neck, fatigue, nauseas, and vomiting.^6^ Most women with AMI have prodromal symptoms, such as shortness of breath, uncommon fatigue or discomfort that spreads to the arm/jaw in the previous weeks. Stable angina is the most frequent clinical presentation in women with IHD as opposed to AMI or sudden death.^7^

A recently published review summarizes some gender-related aspects of the major diagnostic methods, such as their advantages and disadvantages, as well as sensitivity and specificity values.^8^ The lower sensitivity of the exercise test (ET) to detect obstructive coronary disease in women limits its use in the context of ischemic cardiomyopathy (ICM).^9^ The diagnostic accuracy of exercise or dobutamine stress echocardiography (SEC) is higher than that of ET, although inferior to that of other methods, with studies showing similar or inferior performance in women.^10,11^ The addition of tissue doppler assessment has enabled quantitative analysis of viability. The diagnostic accuracy of single photon emission computed tomography (SPECT) myocardial perfusion imaging in women is good, mainly regarding sensitivity.^12^ Some limitations in women are related to false-positive results due to breast attenuation and lower accuracy in small hearts.^13^ Positron emission tomography (PET) is better that SPECT to assess myocardial stress regarding imaging quality and accuracy for both women and men.^14^Additional data characterize plaque inflammation and vulnerability, adverse events, and potential benefit of revascularization.^15^

Both ET and SEC are considered safe for use during pregnancy, because they involve no exposure to radiation, and dobutamine and dipyridamole are considered category B. The SPECT and PET-CT techniques should be avoided, while cardiac magnetic resonance imaging (CMRI) is a good option in pregnancy.^16^

In past decades, differences in cardiovascular physiology and pathophysiology between women and men have been documented. These differences include the electrophysiological properties of cardiac cells, which can influence the occurrence of different clinical arrhythmias between the sexes. These differences might have a multifactorial origin. However, hormonal action and autonomic influence are important factors in the different electrophysiological behaviors between women and men.^17^

The mean heart rate (HR) of women is approximately 3-5 beats/minute higher than that of men.^17^ In addition, women have been shown to have a shorter sinus node recovery time, shorter HV interval, higher ventricular conduction velocity, and increased QT interval.^18^

The prevalence of inappropriate sinus tachycardia is much higher in women. A study of 321 patients with inappropriate sinus tachycardia has shown that 92% of them were of the female sex.^19^ Approximately 60% of the narrow-QRS tachycardias observed in clinical practice are secondary to nodal reentrant tachycardia, and their prevalence is twice higher in women.^20^ The refractory period of the slow pathway is shorter in the female sex, which can enlarge the arrhythmia induction window and justify the larger number of cases in women.^21^ However, it is worth noting that this characteristic does not interfere with the success of the ablation treatment, which corresponds to 95% of the cases in both sexes. In contrast, atrioventricular reentrant tachycardia predominates in the male sex.^18^ Men most frequently have a manifest accessory pathway, located on the left side. Women, however, have almost three-times more pathways on the right side.^21^

The age-adjusted incidence of atrial fibrillation (AF) is 1.5- to 2-times higher in men. However, the risk of AF throughout life is similar in both sexes because of the longer life expectancy of the female sex. In women, there is a disproportionate increase in AF as age advances, thus, at the age of 85 years, the differences in prevalence are discrete between sexes.^17,22^ In addition, women are more symptomatic and have worse quality of life as compared to men. There are several mechanisms associated with the differences between sexes in AF; however, it is worth noting that IHD, more reported in the male sex, can contribute to the higher incidence of AF in the group. Regarding treatment with antiarrhythmic drugs (AADs), women have more adverse effects. The increase in the baseline QT interval can affect the tolerance to AADs, especially the class III ones, requiring more careful monitoring in that group of patients. Regarding ablation outcomes, observational studies have shown that women are less often and belatedly submitted to ablation, usually with worse postprocedural results.^23^

The epidemiological characteristics of ventricular arrhythmias in patients with normal heart vary between sexes. Ventricular tachycardia (VT) originating from the right ventricular outflow tract is more frequent in women, while arrhythmias with a fascicular origin are more frequent in men.^24^ Prepubescent male patients with type I long QT syndrome and prepubescent female patients with type II long QT syndrome are at higher risk for ventricular arrhythmia.^25^ The occurrence of sudden cardiac death in women is almost half that in men, even after adjusting for predisposing factors.^26^

Women with heart failure (HF) are usually older than men and have higher prevalence of HF with preserved ejection fraction (HFpEF). In addition, they more often have nonischemic heart disease, diabetes *mellitus* (DM), and systemic arterial hypertension (SAH). Several factors contribute to the lower inclusion of women in trials on IHD. The factors related to the patient’s conditions are as follows: (1) need to travel and miss work; (2) high burden of family responsibilities; (3) need for high level of commitment, (4) socioeconomic, psychological, cultural, and health barriers. The factors related to trials are as follows: (1) low referral rates and eligibility screening; (2) lack of sex-related eligibility criterion; (3) heterogeneous leadership of the trials, mainly represented by men; (4) exclusion of the elderly. Future actions are important for a higher inclusion of women with IHD in large studies.^27^This position statement, through a joint action of cardiology specialties with expertise in women’s health, is mainly aimed at disseminating information on IHD for the better understanding of its particularities, better management of patients, and consequent reduction in IHD morbidity and mortality.

### 1.2. Highlights of this Position Statement

#### 1.2.1. Epidemiology

• IHD remains the major cause of death in women and men in Brazil. From 1990 to 2019, there was a more marked percent reduction in the standardized IHD mortality rate in women, -55.5 (95% UI, -58.7; -52.3), than in men, -49.5 (95% UI, -52.5; -46.6). That decline differed in the Federative Units in both sexes, being related to population aging and the 2019 Sociodemographic Index (SDI).
• The IHD incidence and prevalence decreased in Brazil in the past 20 years in women and men, despite the increase in early mortality due to IHD between the ages of 18 years and 55 years, especially in women. The age-standardized IHD incidence in women differed in the Brazilian regions, with the Southern and Southeastern regions showing the highest and the Northern region, the lowest rates.
• Women have significantly lower primary angioplasty rates and significantly higher in-hospital mortality rates. The prevalence of MINOCA (myocardial infarction with nonobstructive coronary arteries) is higher in women, with mortality similar to that of obstructive IHD, associated with the risk for major events.
• The 2019 Global Burden of Diseases (GBD) study estimated standardized rates of DALYs (Disability-Adjusted Life Years) due to IHD per 100 000 inhabitants of 1088.4 (992.8; 1158.9) in women and of 2116.5 (95% UI, 1989.9; 2232.2) in men. In 2019, IHD was the second cause of DALYs in women (after neonatal disorders) and in men (after interpersonal violence) in Brazil. Those rates differed in the different Brazilian geographic regions, and the trend of the age-standardized DALYs rates in women from 1990 to 2019 was similar to that of the mortality rates.
• Women are most often exposed to nontraditional cardiovascular risk factors (CVRF), such as mental stress and depression, and more affected by the consequences of social disadvantages due to race, ethnicity, and income. In addition, they are exposed to sex-specific risk factors (RFs), such as pregnancy, menopause, and menarche.

#### 1.2.2. Pathophysiological Bases of Atherothrombotic Disease

Obstructive coronary disease, characterized by the presence of atherosclerotic plaques in coronary artery walls, is the most frequent substrate of IHD in women. However, nonobstructive coronary disease with evidence of damage to the cardiac muscle or other signs of coronary illness is known to disproportionately affect more women.

• The pathophysiological mechanisms involved in MINOCA include coronary plaque rupture, SCAD, coronary vasospasm, coronary microvascular dysfunction, and embolism/thrombosis. The syndromes that clinically mimic MINOCA, such as Takotsubo, myocarditis, and nonischemic cardiomyopathy, are worth noting.

#### 1.2.3. Clinical Presentation, Diagnosis, and Clinical Treatment

• Female sex-specific biological and sociocultural differences in the IHD’s chest pain presentation can explain the differences in its clinical presentation, diagnosis, and management, leading to management delays and, thus, unfavorable outcomes.
• Women’s ischemic symptoms are more often related to emotional or mental stress and less often precipitated by physical activity as compared to men’s. Global risk score and characterization of angina should not be used uniformly in women and men, because of the different impact of RFs and the different clinical manifestations between sexes.
• The relevance of the RFs, such as SAH, obesity, DM, and smoking, differs between sexes. Female-specific RFs are important in risk stratification, and preeclampsia and gestational diabetes increase cardiovascular risk (CVR) throughout a woman’s life.
• Women less often undergo coronary angiography and surgical treatment, including mechanical circulatory support in cardiogenic shock. However, they have higher mortality and more postoperative complications, despite their lower atherosclerotic burden.
• Less than 50% of female patients undergo appropriate drug treatment; in addition, adhesion to treatment is low, and cardiac rehabilitation is underused.
• The treatment of MINOCA and INOCA (ischemia with nonobstructive coronary arteries) is based on lifestyle change, control of RFs, and antianginal treatment.

#### 1.2.4. Diagnosis by use of Graphic Functional Assessment

• Misplacement of the electrocardiogram (ECG) electrodes can lead to misdiagnosis in women. Large breasts or breast prostheses can generate low-voltage complexes and reduce the R wave amplitude in V1 and V2 leads, simulating an inactive area. For chest pain assessment, the same diagnostic ECG criteria are used for women and men, except for the analysis of a subepicardial lesion.
• The ET is recommended as the initial method to assess symptomatic women at intermediate risk for IHD, with normal ECG at rest, and who can exercise. In addition to the ST-segment changes, the ability to exercise, the chronotropic and blood pressure (BP) responses, the HR recovery, and the Duke treadmill score (DTS) assessment are prognostic information that increase ET accuracy, especially in women. Physical functioning is the most important prognostic variable for morbidity and all-cause mortality in women, including the asymptomatic ones. The inability to reach 5 MET is an independent predictor of high risk, with a three-fold increase in mortality as compared to those who exceed 8 MET.
• The cardiopulmonary exercise test (CPET) allows the diagnosis, prognosis, follow-up after therapeutic intervention, and the prescription of aerobic exercises in IHD. The CPET has higher diagnostic accuracy in women’s IHD as compared to the ET. In addition to clinical, hemodynamic, and electrocardiographic criteria, CPET provides the analysis of oxygen pulse, which enables the inference of exertion-induced ischemic ventricular dysfunction, whose finding can be relevant to the diagnosis of macro- and microvascular IHD in women.

#### 1.2.5. Diagnosis by use of Noninvasive Cardiovascular Imaging

• Stress echocardiography has good accuracy to investigate women’s IHD, assesses LV systolic function, enables differential diagnosis, and is safe because it involves no radiation.
• Vascular ultrasound (VUS) is useful to detect carotid plaques as a risk modifier in women with intermediate probability and/or nontraditional RFs, to screen abdominal aortic aneurysm in female smokers or former smokers aged 55-75 years, and to search for silent peripheral arterial disease (PAD).
• Coronary computed tomography angiography (CCTA) has good diagnostic and prognostic accuracy to assess women’s IHD, characterizing and quantifying lesions. Classically, women have less calcified and nonobstructive lesions as compared to men.
• CMRI provides more information to detect women’s IHD: identifies ischemia due to obstructive and nonobstructive IHD, MINOCA, assesses myocardial viability, differentiates ischemic from inflammatory disease, and defines the Takotsubo diagnosis.
• Nuclear imaging assesses the entire IHD spectrum, from obstructive coronary disease to coronary microvascular dysfunction, with no limitation regarding kidney function, arrhythmias, obesity, and intracardiac devices.

#### 1.2.6. Arrhythmias in Ischemic Heart Disease

• As compared to men, women more often have inappropriate sinus tachycardia and nodal reentrant tachycardia and less often have AF, malignant ventricular arrhythmias, such as VT and ventricular fibrillation (VF), and sudden cardiac death. Despite the differences in prevalence between sexes, women benefit from the treatment of cardiac arrhythmias.
• Women with AF have a higher prevalence of SAH, obesity, depression, HFpEF, and valvular heart disease as the cause of arrhythmia. Other risk predictors of AF in women are the lack or scarcity of physical exercise practice, estrogen monotherapy, and multiparity. Women with AF are at a high risk for stroke, with no significant difference in the risk for stroke or systemic embolism and gastrointestinal bleeding between women and men on anticoagulants. A significant reduction in intracranial hemorrhage and all-cause mortality in women with AF on direct oral anticoagulants (DOACs) was observed. Women with AF have worse quality of life than men and are less often submitted to procedures, such as catheter ablation or electric cardioversion (ECV).
• Women less often have ICM as compared to men; in addition, women with IHD and implantable cardioverter defibrillator (ICD) have fewer episodes of VT/VF and of electric storm, and fewer ICD shocks. Women less often have ICM and a smaller fibrosis load than men submitted to cardiac resynchronization therapy (CRT) and respond better to CRT than men, with longer time intervals until the first hospitalization and lower mortality. Therapies, such as ICD and CRT, yield benefits regarding mortality. Women represent 30% of the studies’ population samples with those therapies.
• The percentage of women in studies on VT ablation in individuals with IHD is low (7-13%). The smaller number of indications for invasive procedures, of sustained VT induction, and of appropriate shocks is a factor that might contribute to that reduced percentage.

#### 1.2.7. Atherothrombosis in Pregnancy, Contraception, Infertility, Antiphospholipid Syndrome

• Atherothrombotic disease is one of the most frequent causes of AMI during pregnancy and puerperium.
• The management of acute IHD during pregnancy should prioritize maternal life and follow the recommendations for the general population.
• The triad (smoking, age over 35 years, and prolonged use, > 10 years, of oral combined contraceptive) is considered the determinant factor of the clinical manifestation of atherothrombotic disease during pregnancy and puerperium.
• The complaint of chest pain during pregnancy in women with RF for cardiovascular disease (CVD) should not be underestimated and follow the conventional protocol of ACS investigation.
• Contraceptives are not free from atherothrombotic effects, but, when not prescribed, there is the risk of unplanned pregnancy, mainly of adolescents and women with comorbidities. The contraceptive method selection should be individualized, considering the patient’s preference and age, as well as the method’s safety and efficacy.
• The frequency of proatherosclerotic metabolic disorders, particularly obesity and increased total cholesterol, LDL-cholesterol, and triglycerides, is higher among infertile women.
• The treatment of infertility is considered a potential RF for hypertensive disorders in the subsequent pregnancy. However, a correlation between fertilization treatment and cardiovascular events has not yet been demonstrated.
• Diagnosis of antiphospholipid syndrome (APS) should be considered when clinical manifestations of vascular thrombosis and/or recurrent obstetrical complications are present, and its investigation is mandatory when stroke and AMI occur in young women.

#### 1.2.8. Ischemic Cardiomyopathy

• Heart failure related to ICM is an important cause of morbidity and mortality in women, who tend to develop the condition at a more advanced age than men.
• The HFpEF is more common in women, but the ischemic etiology tends to manifest in the dilated form and reduced ejection fraction, although with less endomyocardial fibrosis as compared to men.
• Women are less represented in clinical trials of HF. Nevertheless, the guideline recommendations on pharmacological and advanced treatment do not suggest individualized treatment for women.

#### 1.2.9. Percutaneous Coronary Intervention

• For the interventional procedure, strategies to reduce the risk of bleeding and vascular complications should be adopted, prioritizing the radial access and use of auxiliary drugs at doses appropriate to weight and kidney function.
• Functional assessment in coronary disease, by analyzing the fractional flow reserve (FFR), is highly useful in intermediate obstructions (40% to 70%), when ischemia cannot be confirmed by use of noninvasive methods. Women seem to have higher FFR values in nonobstructive coronary disease, confirming the importance of measuring FFR in women, which is a significant univariate predictor of prognosis in the group.
• Profiles of high risk for bleeding should be identified and excellence in the intervention outcomes should be sought by use of careful selection and proper preparation of lesions, low threshold for the use of intravascular imaging guidance, optimized stent implantation, and personalized post-intervention antiplatelet strategy.
• In cases of MINOCA, strategies should be used in the catheterization laboratory to optimize the etiological diagnosis.
• The diagnosis of microvascular dysfunction, most frequent in women, should be considered in both the chronic and acute scenarios, and that diagnosis can be confirmed by invasive coronary physiological methods.

#### 1.2.10. Myocardial Revascularization and Cardiac Transplantation

• The use of arterial grafts in myocardial revascularization (MRV) is less frequent in women.
• In the postoperative period, women have a higher rate of complications.
• A delay in surgical indication has a negative impact on the postoperative results.
• Currently women correspond to 25% of heart transplantation recipients, have a higher rate of complications, mainly rejection, and better survival than men after cardiac transplantation.
• Women have a lower incidence of malignant tumors postoperatively.

#### 1.2.11. Rehabilitation in Ischemic Cardiomyopathy

• Referral to cardiac rehabilitation should be part of the medical prescription for women with IHD, including those with SCAD and MINOCA.
• The initial assessment and cardiac rehabilitation program prescription should be directed to the women’s specificities, aimed at better adherence and lower abandonment rates.

## 2. Epidemiology of Ischemic Heart Disease in Women

### 2.1. Introduction

The population growth and extended life expectancy have contributed to increase the total number of deaths due to IHD around the world. The age-standardized mortality rates due to IHD in women and men have gradually decreased in most countries, probably because of the improvement in diagnosis and treatment, despite the significant increase in obesity, fasting serum glucose elevation, and metabolic syndrome.^28-31^

In Brazil, IHD remains the major cause of death in women and men. The IHD incidence and prevalence have decreased in Brazil over the past 20 years in women and men, despite the increase in early mortality due to IHD between the ages of 18 years and 55 years, especially in women. In addition, IHD was the second major cause of DALYs in women in Brazil from 1990 to 2019.^31^

Women suffer a greater impact from traditional CVRFs and have worse prognosis, although their IHD risk and atherothrombotic burdens are lower. Women more frequently have nontraditional CVRFs, such as mental stress and depression, and suffer from social disadvantages due to race, ethnicity, and income.^32^

MINOCA predominates in women,^29^ whose outcomes are substantially worse as compared to those of men. In addition, younger women (<55 years) and the subgroups of women defined by race, ethnicity, socioeconomic *status*, and schooling have even more marked disparities regarding IHD diagnosis, treatment, and prognosis.^29,32^

This chapter is aimed at summarizing the findings on IHD epidemiology in women, especially the Brazilian ones.

### 2.2. Mortality

Recent data from the GBD 2021 project have estimated for CVD in Brazil standardized rates of 3568.0 DALYs (1 DALY represents the loss of the equivalent of 1 year of full health) and of 162.2 deaths per 100 000 inhabitants, with a standardized prevalence rate of 6905.6 per 100 000 inhabitants. In addition, according to the GBD 2021, the estimated rates for IHD in Tropical Latin America (Brazil and Paraguay), in 2021, were as follows: prevalence of 1989.5, mortality of 67.7, and 1439.6 DALYs per 100 000 inhabitants. Despite the significant progress in decreasing the number of deaths from CVD from 1980 to the end of 2021, there has been a worrisome increase in the crude mortality rate and number of DALYs from CVD in recent years.^28^

Cardiovascular disease is the major cause of death in women worldwide and, in 2021, CVD was responsible for approximately one third of all female deaths. Mortality from CVD decreased globally in the past 30 years, with a more significant decline in countries with high SDI (SDI = mean composed by per capita income, mean education level, and fertility rate). However, in high-income regions, the CVD mortality reduction trend has decreased, and, in 2017, the number of female deaths increased in some countries, such as the United States and Canada.^29^

In the region of the Americas, the age-adjusted IHD mortality rate decreased from 2000 to 2019 in men, passing from 149.08 (95% UI, 138.23; 168.08) to 96.02 (95% UI, 83.48; 117.19), with a percent change of -2.3 (95% UI, -2.5; -2.1), and in women, passing from 92.36 (95% UI, 81.35; 109.42) to 54.84 (95% UI, 45.28; 71.76), with a percent change of -2.7 (95% UI, -3.0; -2.5). In that same period, the mortality rates decreased significantly in 24 countries. Costa Rica, Canada, and Chile had the highest percent reductions, while a significant increase occurred in the Dominican Republic and Grenada.^30^

In Brazil, from 1990 to 2019, a decline in standardized CVD mortality rate was observed in women. According to the GBD 2019 study, IHD (defined as individuals with previous myocardial infarction, s angina, or ischemic HF) was the major responsible for death in women, followed by type 2 DM and stroke, in that order (Figure 2.1). That decline was different in the Brazilian Federative Units in both sexes (Figure 2.2 and Figure 2.3A), being related to population aging and the 2019 SDI.

**Figure 2.1:** – Ranking of the ischemic heart disease mortality rates (per 100 000 inhabitants) according to sex, in Brazil, in 1990 and 2019. COPD: chronic obstructive pulmonary disease.

Source: Global Burden of Diseases (GBD) 2019 Study.*^31^*

**Figure 2.2:** – Ranking of the ischemic heart disease mortality rates (per 100 000 inhabitants) according to the Federative Units and sex, in Brazil, in 1990 and 2019.

Source: Global Burden of Diseases (GBD) 2019 Study.*^31^*

**Figure 2.3:** – Ischemic heart disease in women: standardized rates of mortality (A), DALYs (B), prevalence (C), and incidence (D), per 100 000 inhabitants, according to the Federative Units, in Brazil, in 2019.

Source: Global Burden of Diseases (GBD) 2019 Study.*^31^*

Data from the GBD 2019 estimated that, in 2019, the age-standardized IHD mortality rates were 58 (95% UI, 51; 63) and 96 (95% UI, 88; 101) per 100 000 inhabitants in Brazil, in women and men, respectively. From 1990 to 2019, there was a more marked percent reduction in the standardized IHD mortality rate in women, -55.5 (95% UI, -58.7; -52.3), than in men, -49.5 (95% UI, -52.5; -46.6) (Figure 2.4A). In all age groups, the IHD mortality rates were higher in men than in women and increased with aging in both sexes (Table 2.1).^31^

**Figure 2.4:** – Ischemic heart disease: standardized rates of mortality (A), DALYs (B), prevalence (C), and incidence (D), per 100 000 inhabitants, according to sex, in Brazil, in 2019.

Source: Global Burden of Diseases (GBD) 2019 Study.*^31^*

**Table 2.1:** – Ischemic heart disease: numbers of death, rates of mortality, DALYs, prevalence, and incidence, and percent changes of the rates, per 100 000 inhabitants, according to age group and sex, Brazil, 1990 and 2019. Source: Global Burden of Diseases (GBD) 2019 Study.31

ISCHEMIC HEART DISEASES	1990		2019		Percent Change for rate (UI 95%)
	Number (UI 95%)	Rate (UI 95%)	Number (UI 95%)	Rate (UI 95%)	
**DEATH**					
**Female**					
15-49 years	3909.7 (3741.1;4083)	10 (9.6;10.5)	3813.4 (3539.1;4106.8)	6.5 (6.1;7)	-35.1 (-40.4;-28.7)
50-69 years	15601.9 (14922.1;16385.3)	191.1 (182.8;200.7)	20769 (19331.1;22110.3)	97 (90.3;103.2)	-49.3 (-53.2;-44.9)
70+ years	30524.2 (27413.3;32326.3)	1301.6 (1169;1378.5)	50625.3 (42567.4;55652)	670.5 (563.8;737)	-48.5 (-53.1;-44.6)
Age-standardized	50035.8 (46474.1;52421.2)	130.1 (118.1;137.2)	75207.7 (66247.3;81307.1)	57.9 (51;62.6)	-55.5 (-58.7;-52.3)
All Ages	50035.8 (46474.1;52421.2)	66.5 (61.7;69.6)	75207.7 (66247.3;81307.1)	67.8 (59.8;73.3)	2 (-6.1;9.8)
**Male**					
15-49 years	9180.2 (8829;9551.6)	24.3 (23.4;25.3)	9018.2 (8513.2;9574.1)	15.8 (14.9;16.8)	-35 (-39.3;-30.4)
50-69 years	29205.2 (28207.5;30291.4)	388.1 (374.8;402.5)	39360.8 (37264.7;41601.2)	207.9 (196.9;219.8)	-46.4 (-50;-42.7)
70+ years	28825.8 (26903;30176.3)	1529.2 (1427.2;1600.9)	47659.7 (42457.3;51239)	860.6 (766.7;925.3)	-43.7 (-47.9;-40.1)
Age-standardized	67211.2 (64502.5;69571)	189.3 (178.2;197)	96038.7 (89069.1;101545.9)	95.6 (87.7;101.3)	-49.5 (-52.5;-46.6)
All Ages	67211.2 (64502.5;69571)	91.4 (87.7;94.6)	96038.7 (89069.1;101545.9)	90.8 (84.2;96)	-0.6 (-7.2;5.6)
**INCIDENCE**					
**Female**					
15-49 years	7197 (5499.8;9046.3)	18.5 (14.1;23.2)	11635.5 (9059.9;14317.5)	19.9 (15.5;24.5)	7.6 (0.9;15.5)
50-69 years	16567.2 (13251.3;20250.1)	203 (162.3;248.1)	45832.6 (37412.2;55078.3)	214 (174.7;257.2)	5.4 (0.8;11.4)
70+ years	16711.1 (13816.2;20044.8)	712.6 (589.2;854.8)	43920.1 (37060.4;51680.9)	581.7 (490.8;684.4)	-18.4 (-21.7;-14.7)
Age-standardized	40475.2 (35479;45761.4)	88.7 (77.7;99.9)	101388.2 (89389.1;114296.5)	78.2 (69;88.1)	-11.8 (-14.7;-8.5)
All Ages	40475.2 (35479;45761.4)	53.8 (47.1;60.8)	101388.2 (89389.1;114296.5)	91.5 (80.6;103.1)	70.1 (64.3;76)
**Male**					
15-49 years	13927.2 (11155;17134.5)	36.9 (29.6;45.4)	22270 (17691.1;27177.9)	39.1 (31;47.7)	5.8 (0.2;11.6)
50-69 years	33048.3 (27279.8;39599.5)	439.2 (362.5;526.2)	84424.7 (70604.7;101021)	446 (373;533.7)	1.6 (-2.6;6.2)
70+ years	21875.5 (18078.2;25972.6)	1160.5 (959.1;1377.8)	52578.4 (44648;61725.3)	949.5 (806.2;1114.6)	-18.2 (-22.1;-14.6)
Age-standardized	68851 (60766.7;77363.5)	167.8 (148;189)	159273.1 (140195.3;178778.9)	148 (130.4;166.3)	-11.8 (-14.7;-9)
All Ages	68851 (60766.7;77363.5)	93.6 (82.6;105.2)	159273.1 (140195.3;178778.9)	150.5 (132.5;169)	60.9 (55.6;66.1)
**PREVALENCE**					
**Female**					
15-49 years	67405.4 (56057.1;81268.7)	173.1 (144;208.7)	124695.9 (103531.2;149594.6)	213.2 (177;255.8)	23.2 (20;26.5)
50-69 years	204529 (170177.7;249197.2)	2505.7 (2084.9;3053)	534224.8 (445383.3;647280.5)	2494.7 (2079.8;3022.6)	-0.4 (-2.8;2)
70+ years	212483.8 (175519;255435.7)	9060.9 (7484.6;10892.5)	694914.7 (582274.2;826926.3)	9203.3 (7711.5;10951.6)	1.6 (-1.7;5)
Age-standardized	484418.3 (417746.5;563544.8)	1071.8 (925.1;1242.3)	1353835.3 (1172305.1;1562949.3)	1045.6 (904.6;1208.7)	-2.4 (-4.2;-0.5)
All Ages	484418.3 (417746.5;563544.8)	643.6 (555;748.7)	1353835.3 (1172305.1;1562949.3)	1221.1 (1057.4;1409.8)	89.7 (85.1;95.2)
**Male**					
15-49 years	147353.9 (119423.1;180805.2)	390.8 (316.7;479.5)	280252.9 (227615.4;344765)	491.6 (399.3;604.8)	25.8 (21.9;29.2)
50-69 years	477021.9 (392697.1;589599.4)	6339 (5218.4;7835)	1231669.7 (1021223.9;1511754.2)	6506.9 (5395.1;7986.6)	2.6 (0.2;5)
70+ years	371414.9 (301282.3;450276.3)	19703.6 (15983;23887.2)	1138137.6 (940499.4;1375886.1)	20552.3 (16983.4;24845.5)	4.3 (1.6;7.3)
Age-standardized	995790.6 (851258.4;1169649.6)	2498.8 (2137;2941.3)	2650060.2 (2275770;3115190.4)	2534 (2170.4;2975.5)	1.4 (-0.4;3.3)
All Ages	995790.6 (851258.4;1169649.6)	1353.6 (1157.1;1589.9)	2650060.2 (2275770;3115190.4)	2504.8 (2151;2944.4)	85 (80.7;89.2)
**DALY**					
**Female**					
15-49 years	189243.4 (181288.6;197784.2)	486 (465.6;508)	182088.5 (169104.9;195954)	311.4 (289.2;335.1)	-35.9 (-41.1;-29.8)
50-69 years	453002.6 (433556.6;475548.9)	5549.8 (5311.6;5826.1)	607233.3 (565939.9;646284.2)	2835.6 (2642.8;3018)	-48.9 (-52.8;-44.6)
70+ years	407987.8 (373418.1;429318.3)	17397.8 (15923.6;18307.4)	625969.5 (538771.7;681196.9)	8290.2 (7135.3;9021.6)	-52.3 (-56.1;-49)
Age-standardized	1050233.8 (998139;1093952.2)	2303.2 (2162.5;2403.9)	1415291.4 (1291761.5;1506628.9)	1088.4 (992.8;1158.9)	-52.7 (-56.1;-49.3)
All Ages	1050233.8 (998139;1093952.2)	1395.3 (1326.1;1453.4)	1415291.4 (1291761.5;1506628.9)	1276.6 (1165.2;1359)	-8.5 (-15.3;-1.6)
**Male**					
15-49 years	443883.8 (427008;461879.9)	1177.1 (1132.3;1224.8)	437993 (414351.3;464321.6)	768.3 (726.8;814.5)	-34.7 (-38.8;-30.3)
50-69 years	873719.6 (844632.7;906253.8)	11610.6 (11224.1;12042.9)	1188364.7 (1122117.2;1256445.4)	6278.1 (5928.1;6637.8)	-45.9 (-49.5;-42.3)
70+ years	425524.4 (400309;444510.9)	22574.1 (21236.4;23581.3)	679374.4 (620110.3;725589)	12268 (11197.9;13102.6)	-45.7 (-49.2;-42.3)
Age-standardized	1743127.7 (1681550;1801264.2)	4013.2 (3852.3;4150.3)	2305732.1 (2173570.4;2429437.7)	2116.5 (1989.9;2232.2)	-47.3 (-50.4;-44)
All Ages	1743127.7 (1681550;1801264.2)	2369.5 (2285.8;2448.5)	2305732.1 (2173570.4;2429437.7)	2179.4 (2054.4;2296.3)	-8 (-13.6;-2.2)

A study with Brazilian data from the Brazilian Mortality Information System (SIM) has reported that, between 1981 and 2001, the coefficient of death related to IHD remained stable for women in the Northern and West-Central regions, while decreased in the Southern and Southeastern regions and increased in the Northeastern region. For men, there was a decreasing trend in the events in the Southern and Southeastern regions.^33^

A study assessing 166 514 coronary angioplasty procedures to treat IHD performed in 180 hospitals between 2005 and 2008 has reported mean in-hospital mortality of 2.3% (minimum of 0%, maximum of 11.4%), which varied according to the geographic region, being lowest in the Southeastern (2.0%) and highest in the Northern region (3.6%). The mortality rate was higher in women and in patients over the age of 65 years.^34^

Women undergoing MRV have higher mortality and more postoperative complications, despite their smaller atherosclerotic burden. The increase in mortality at the time of MRV is higher at younger ages than at more advanced ages, and a three-fold higher risk of death is estimated in women aged < 50 years, despite adjustment for RFs.^35^

A study performed from 1996 to 2016 with data from the SIM corrected for underreporting and garbage code [causes of death that should not be considered as underlying causes of death or are nonspecific, being, thus, considered insufficient in terms of prevention, such as ICD-10 codes I50 (heart failure) and R96 (sudden death)] analyzed the trends of AMI mortality according to sex, Brazilian geographical regions, and place of residence (capital *versus* noncapital cities). The authors have reported that the age-standardized AMI mortality rate decreased 44% in Brazil, with significant regional differences (+5%, North; +11%, Northeast; -35%, West-Central; -68%, Southeast; and -85%, South). The temporal variations were more significant in women and in the capital cities. The corrected age-standardized AMI mortality rates decreased 49% and 23% among women living in the capital cities and other municipalities, respectively.^36^

Another study performed with data from the SIM has reported an approximately 2.2% decline in AMI over the past 20 years in the most developed geographical regions (Southeastern, Southern, and West-Central), stabilization in the Northern region, and increase in the Northeastern region. The authors have predicted that the trend will persist up to 2030. Those variations might have been related to improvements in social development, in CVRFs, and in access to the health system and its coverage, as well as to the better coding discrimination in the certificates of death in the Northern and Northeastern regions.^37^

The VICTIM registry has assessed 878 patients diagnosed with ST-elevation myocardial infarction (STEMI) admitted to one public and three private hospitals performing primary angioplasty in the Sergipe state from December 2014 to June 2018. Of those patients, 33.4% were women. Of the total, only 53.3% underwent myocardial reperfusion (134 women *versus* 334 men). Women had significantly lower primary angioplasty rates (44% *versus* 54.5%; p = 0.003) and significantly higher in-hospital mortality rates (16.1% *versus* 6.7%; p < 0.001) than men.^38^

Another prospective single-center study conducted in the city of Recife, Pernambuco state, on 709 consecutive patients with STEMI (women, 36%; mean age, 61 years) from February 2018 to February 2019 has reported that women were older (63.13 years *versus* 60.53 years, p = 0.011), most frequently had SAH (75.1% *versus*. 62.4%, p = 0.001), DM (42.2% *versus* 27.8%, p < 0.001), and dyslipidemia (34.1% *versus* 23.9%, p = 0.004), and less frequently underwent percutaneous coronary intervention (PCI) through the radial access (23.7% *versus* 46.1%, p < 0.001) than men. The in-hospital mortality rate was significantly higher in women as compared to men (13.2% *versus* 5.6%, p = 0.001), and the female sex was an independent predictor of in-hospital mortality (OR 2.79; 95% CI, 1.15 – 6.76; p = 0.023).^39^

### 2.3. Prevalence and Incidence

According to data from the GBD 2019 study, the age-standardized IHD prevalence rates in Brazil were 1046 (95% UI, 905; 1209) per 100 000 women and 2534 (95% UI, 2170; 2975) per 100 000 men (Table 2.1).^31^The age-standardized IHD prevalence in women differed in the Brazilian regions, being higher in the Southeastern and Southern regions and lower in the Northern region (Figure 2.3C).^31^ The standardized IHD prevalence rate per 100 000 inhabitants from 1990 to 2019 had a percent reduction in women, -2.4 (95% UI, -4.2; -0.5), but a slight increase in men, 1.4 (95% UI, -0.4; 3.3) (Table 2.1 and Figure 2.4C).

The GBD 2019 study estimated an IHD incidence of 260 661 (95% UI, 230 100-293 617) events (mainly myocardial infarction) in Brazil in 2019. In all age groups, the IHD incidence was higher in men than in women (Table 2.1). In 2019, the age-standardized IHD incidence rates were 78 (95% UI, 69; 88) per 100 000 women and 148 (95% UI, 130; 166) per 100 000 men. The age-standardized IHD incidence in women differed in the Brazilian geographic regions, being higher in the Southeastern and Southern regions and lower in the Northern region (Figure 2.3D).^31^ The standardized IHD incidence rate per 100 000 inhabitants from 1990 to 2019 had a percent reduction in women, -11.8 (95% UI, -14.7; -8.5), and in men, -11.8 (95% UI, -14.7; -9) (Table 2.1 and Figure 2.4D).

The AMI prevalence in women is lower in the younger than in the older age groups, but there has been a worrisome trend in recent years, considering that the proportion attributable to young patients (35-54 years) increased from 27% to 32% in the past two decades, and that increase was higher among young women (21% to 31%).^40^

The prevalence of MINOCA is higher in women. The VIRGO (*Variation in Recovery: Role of Gender on Outcomes of Young AMI Patients*) study, performed from 2008 to 2012, included prospectively 2690 patients with AMI, aged 18-55 years, from 103 hospitals, at the proportion of 2 women to 1 man. Of the 2374 patients submitted to coronary angiography, women were 5-fold more likely to have MINOCA than men (14.9% *versus* 3.5%; OR 4.84; 95% CI, 3.29 – 7.13). Women with significant coronary obstruction more often were menopausal (55.2% *versus* 41.2%; p < 0.001) or had a gestational diabetes history (16.8% *versus* 11.0%; p = 0.028). It is worth noting that mortalities from MINOCA at 1 and 12 months were similar to those from coronary obstruction at the same times.^30^

### 2.4. Burden of Diseases

The GBD 2019 study estimated standardized DALYs rates due to IHD per 100 000 inhabitants of 1088.4 (992.8; 1158.9) in women and of 2116.5 (95% UI, 1989.9; 2232.2) in men (Table 2.1). The second most common cause of DALYs in Brazil was IHD among women, following neonatal complications, and among men, following interpersonal violence, in 2019.^31^ Those rates differed in the different Brazilian geographic regions, and the trend of the age-standardized DALYs rates from 1990 to 2019 in women was similar to that of the mortality rates (Figure 2.3B). The Federative Units with the highest numbers of DALYs due to IHD per 100 000 inhabitants in women in 2019 were Rio de Janeiro, Pernambuco, Rio Grande do Sul, Paraíba, Alagoas, and São Paulo, in that order (Figure 2.5).

**Figure 2.5:** – Ranking of the ischemic heart disease DALYs rates (per 100 000 inhabitants) according to the Federative Units and sex, in Brazil, in 1990 and 2019.

Source: Global Burden of Diseases (GBD) 2019 Study.*^31^*

From 1990 to 2019, there was a higher percent reduction in the standardized DALYs rate due to IHD per 100 000 inhabitants in women, -52.7 (95% UI, -56.1; -49.3), than in men, -47.3 (95% UI, -50.4; -44) (Table 1 and 4B).

**Figure 4.1:** – Pathophysiology of the ischemic cascade of anginal equivalents.

### 2.5. Risk Factors

The traditional RFs, such as SAH, hyperlipidemia, DM, smoking, poor eating habits, and sedentary lifestyle, are prevalent among women with IHD and they go along with the emerging RFs, such as metabolic, pregnancy-related, and autoimmune disorders, in addition to sleep apnea, chronic diseases, low socioeconomic level, burnout, and psychosocial factors, such as depression and anxiety.^41^

In the PURE (*Prospective Urban Rural Epidemiological*) Study, 202 072 individuals aged 35-70 years, from urban and rural communities in 27 countries, from January 2005 to May 2019, were followed up for a mean of 9.5 (IQR: 8.5–10.9) years. Women had a lower CVRF burden according to two different traditional risk scores (INTERHEART and Framingham). Primary prevention strategies (healthy lifestyle and use of approved medications) were most frequent in women, whose incidence of CVD was lower. However, secondary prevention treatments for IHD were less frequent in women than in men. The differences between women and men with or without previous CVD and IHD regarding treatments and results were more significant in low/middle-income countries, with few differences in high-income countries.^42^

The IHD attributable RFs are shown in Figure 2.6. Dietary risks and SAH are number one in the ranking of IHD attributable RFs in both sexes, worldwide.^28^

**Figure 2.6:** – Ranking of the risk factors attributable to ischemic heart disease worldwide in both sexes, in 2021.

Source: Global Burden of Diseases (GBD) 2019 Study.*^28^*

In the case-control study derived from the VIRGO study, with 2264 patients with AMI (18-55 years) and 2264 paired controls, 3122 (68.9%) were women, with a median age of 48 (44-52) years. The following seven RFs represent collectively the larger part of the total risk of AMI in women (83.9%) and men (85.1%): DM [OR 3.59 (95% CI, 2.72-4.74) in women *versus* 1.76 (1.19-2.60) in men]; depression [OR 3.09 (95% CI, 2.37-4.04) in women *versus* 1.77 (1.15-2.73) in men]; SAH [OR 2.87 (95% CI, 2.31-3.57) in women *versus* 2.19 (1.65-2.90) in men]; current smoking [OR 3.28 (95% CI, 2.65-4.07) in women *versus* 3.28 (2.65-4.07) in men]; family history of early myocardial infarction [OR 1.48 (95% CI, 1.17-1.88) in women *versus* 2.42 (1.71-3.41) in men]; low family-income [OR 1.79 (95% CI, 1.28-2.50) in women *versus* 1.35 (0.82-2.23) in men]; hypercholesterolemia [OR 1.02 (95% CI, 0.81-1.29) in women *versus* 2.16 (1.49-3.15) in men]. There were significant differences between the sexes in RF associations: SAH, depression, DM, current smoking, and family history of DM showed stronger associations with AMI in young women, while hypercholesterolemia showed a stronger association in young men.^43^

A study with 10 112 patients (29% women) with IHD recruited in Europe, Asia, and the Middle East between 2012 and 2013 has reported that, as compared to men, women were less likely to achieve targets for total cholesterol [OR 0.50 (95% CI, 0.43-0.59)], LDL-cholesterol [OR 0.57 (95% CI, 0.51-0.64)], and glucose [OR 0.78 (95% CI, 0.70-0.87)], or to be physically active [OR 0.74 (95% CI, 0.68-0.81)] or non-obese [OR 0.82 (95% CI, 0.74-0.90)]. However, women showed better BP control [OR 1.31 (95% CI, 1.20-1.44)] and were more likely to not smoke [OR 1.93 (95% CI, 1.67-2.22)] than men. The authors have concluded that control for secondary prevention of RFs related to IHD was usually worse in women than in men.^44^

### 2.6. Conclusion

Ischemic heart disease contributes significantly to women’s morbidity and mortality. Although the IHD mortality, DALYs, prevalence and incidence rates have decreased over the past 20 years, data indicate that mortality from IHD in women aged 35-54 years is increasing. Women’s IHD recognition, discussion, education, and appropriate treatment are necessary to reduce the IHD diagnosis and treatment gaps, and the unfavorable IHD outcomes in women.

## 3. Pathophysiological Bases of Atherothrombotic Disease

### 3.1. Introduction

The recognition of traditional RFs for atherosclerotic CVD, as well as emerging and nontraditional RFs that are specific to or more frequent in women, in addition to their different impacts contribute to the new understanding of the mechanisms that lead to worse outcomes in women (Figure 3.1). More details on the importance of RFs in women can be seem in the subsequent chapter.

**Figure 3.1:** – Traditional and nontraditional risk factors for atherosclerotic cardiovascular disease in women.

The presentations of atherosclerotic CVD can be acute, such as MINOCA, which represents 3% to 15% of the cases, or can be related to cases of INOCA.^45,46^ The diagnostic criterion for MINOCA is the presence of AMI with no obstructive lesion (> 50%) in any epicardial artery and lack of alternative diagnosis.^45-47^

The pathophysiological mechanisms involved in MINOCA include coronary plaque rupture, SCAD, vasospasm, coronary microvascular dysfunction, and embolism/thrombosis. The syndromes that clinically mimic MINOCA, such as Takotsubo, myocarditis, and nonischemic cardiomyopathy are worth mentioning.^48^

### 3.2. Plaque Rupture

Plaque rupture is a comprehensive term that comprises rupture, erosion, and calcified nodules in the coronary arteries. Plaque rupture with thrombus formation is the usual manifestation of ACS, but it might not obstruct the vessel lumen. Erosion is the formation of a thrombus adjacent to the luminal surface, resulting from cell apoptosis and recruitment of neutrophiles. The calcified nodules are usually identified by use of intravascular imaging as protrusions into the coronary lumen, and they can undergo fracture with fibrin accumulation in the fibrous cap.^48^ Female sex and smoking are associated with a higher incidence of erosion than that of plaque rupture. The intravascular changes can be detected by use of ultrasound or optic coherence tomography (OCT). The HARP Study has shown that plaque rupture is the most frequent cause of MINOCA, evidenced in 43% of the women with that condition.^49^

### 3.3. Spontaneous Coronary Artery Dissection

Spontaneous coronary artery dissection results from the formation of a false arterial lumen in the absence of significant atherosclerotic disease. Two mechanisms have been described, one involving laceration of the intimal layer and resulting in false lumen (*inside-out*), while the other involves intramural hemorrhage with or without rupture of the intimal layer (*outside-in*).^48^ There are multiple predisposing factors, such as genetic factors, female sex, pregnancy and estrogen therapy, fibromuscular dysplasia, systemic inflammatory diseases, in addition to external factors, such as emotional stress, intense physical activity, and use of stimulant drugs. Data from international registries point to SCAD as the major cause of AMI in the perigestational period, occurring more frequently in the third gestational trimester and right after delivery.^50^

According to angiographic characteristics, four types of SCAD have been described: type 1: evident arterial wall with multiple radiolucent lumens; type 2: one segment with diffuse narrowing (normally > 20mm) and normal proximal and distal segments (type 2A) or diffuse narrowing extending to the distal extremity of the vessel (type 2B); type 3: short segment of stenosis (< 20mm) that mimics atherosclerosis; type 4: characterized by dissection with total abrupt occlusion of a distal coronary segment. According to international guidelines, type 4 SCAD does not constitute MINOCA, while the other types with nonobstructive lesions or lesions not identified on angiography are included in that classification.^51^

### 3.4. Coronary Artery Spasm

Coronary artery spasm as a cause of ischemia is diagnosed in the presence of chest pain with or without ischemic change on ECG and vasoconstriction > 90% on angiography. It can occur spontaneously or be induced by acetylcholine or ergonovine. The pathophysiological mechanism is hyperreactivity of the muscle layer of epicardial arteries and microcirculation, but still not completely clarified.^52^ Some individuals have distinct vasospasm triggers, such as stress, hyperventilation, daily periods and seasonal cycles (clusters), suggesting intracellular and post-receptor changes related to hyperreactivity. Smoking and Asian ethnicity are reported as predisposing factors, while the other traditional RFs seem not related to an increased risk. Some studies have suggested that women are more susceptible to spasms induced by acetylcholine than by ergonovine.^53^

### 3.5. Coronary Microvascular Dysfunction

The coronary microvascular circulation comprises vessels with diameter < 0.5mm, not visualized on conventional angiography, although they represent more than 70% of the coronary vascular resistance.^54^Coronary microvascular dysfunction is defined as a change in the coronary flow reserve (CFR) or an increase in intramyocardial resistance in the absence of obstruction of epicardial coronary arteries. The CFR can be assessed by using noninvasive measures, with doppler or thermodilution techniques. The CFR is expressed as the ratio between coronary flows at maximal hyperemia and at baseline condition, and a value <2.0 is considered abnormal. The maximum index of microcirculatory resistance is calculated by multiplying the distal coronary pressure during maximal hyperemia by the mean hyperemic transit time, and a value ≥ 25 indicates dysfunction of the microcirculation. The diagnosis of microvascular angina has been recently proposed by the COVADIS (*Coronary Vasomotor Disorders International Study*) Group in the presence of suggestive findings of ischemia, absence of obstructive coronary disease, and evidence of microvascular dysfunction.^54^

From the pathophysiological viewpoint, the causes seem diverse and complex. Two endotypes have been described, structural and functional, and they often coexist. In the structural aspect, there is endothelial dysfunction, arteriolar remodeling, and capillary rarefaction, which lead to a reduction in the increment of coronary flow at rest and an increase in demand during exertion. The functional manifestation relates to the inefficient cardiac-coronary coupling, resulting from the increased microvascular tonus during peak exercise and at rest, leading to a higher myocardial oxygen demand in the scenario of exhausted coronary vasodilator reserve.^48,52^

Several studies have shown sex-related differences in response to functional tests, indicating that women more often have coronary microvascular dysfunction and epicardial vasospasm as compared to men.^53^

### 3.6. Embolism and Thrombosis

Epicardial coronary artery embolism without atherosclerotic substrate is an uncommon cause of MINOCA, usually diagnosed by exclusion or presumptively.^55^The National Cerebral and Cardiovascular Center Group classifies this condition as possible when there is evidence of embolism/thrombosis in a coronary artery without atherosclerosis, concomitant embolism to other arteries or multiple sites, and/or in the presence of predisposing factors to embolism. Several causes of embolism, such as direct, paradoxical, and iatrogenic sources, or even systemic hypercoagulability, have been described. The direct sources include thrombi formed in the left atrial appendage (LAA), when AF is present, and in the left ventricle, mitral and aortic valves, and proximal coronary segments. The emboli can have an hematogenic origin or originate from any other tissue content, such as neoplasms and valvular debris. Individuals with right-left shunts, such as atrial communication, patent *foramen ovale*, and arteriovenous malformations, can have paradoxical embolism. In addition, the increased number of invasive coronary and valvular procedures, as well as of those with systemic access, has been related to cases of coronary embolism.^45,55^

### 3.7. Takotsubo Syndrome

Takotsubo syndrome is an acute cardiac entity, with clinical presentation similar to that of ACS.^56^Several clinical and phenotypic presentations are known, the typical one being a circumferential left (bi-) ventricular contraction abnormality profile that extends beyond a coronary artery supply territory and seems to follow the anatomical cardiac sympathetic innervation. The syndrome affects predominantly postmenopausal women, being preceded, in more than 70% of the cases, by emotional or physical stress. Regarding incidence, there is an imbalance between sexes, with women representing 90% of the cases of Takotsubo syndrome, responsible for up to 10% of the postmenopausal women with acute chest pain. The gender differences in the pathophysiology of the syndrome remain underreported, thus requiring further investigation. The Takotsubo syndrome pathogenesis remains undefined. Several pathophysiological mechanisms have been proposed, such as myocardial ischemia (multivessel coronary vasospasm, microvascular dysfunction, aborted myocardial infarction), LV outflow tract obstruction, blood-borne catecholamine myocardial toxicity, epinephrine-induced switch in signal trafficking, and autonomic nervous system (ANS) dysfunction.^56,57^

### 3.8. Myocarditis

Myocarditis is an inflammatory heart disease that can be caused by an infection, most frequently, or by immune system activation or toxicity. One common presentation of myocarditis is mimicking AMI findings, such as chest pain and increased troponin levels, usually in young men. The pathophysiological aspects vary according to the causing agent and etiology but involve nonischemic degeneration and necrosis of the cardiomyocyte. In a series of cases of myocarditis due to parvovirus B19, the induction of epicardial coronary spasm reproduced the symptoms, suggesting that part of the symptoms can have a coronary origin and be related to a virus-induced arteritis.^58^

## 4. Ischemic Heart Disease Clinical Presentation, Diagnosis, and Clinical Treatment

### 4.1. Chest Pain of Ischemic Etiology

Chest pain is the second most common complaint in emergency services, after traumatic causes.^59,60^ It affects 20% to 40% of the population,^61^ being more frequent in women. Thus, the differences in IHD clinical presentation, diagnosis, and management between men and women should be known.^60^Only 5.1% of the patients with chest pain in emergency services have ACS, and more than half of those patients have chest pain of noncardiac etiology.^59^

Until recently, the publications have only rarely reported on sex-specific differences in CVD, and the pathophysiological differences exclusively related to women were not understood^32^ (Figure 4.1). The lack of knowledge about the sex-specific biological and sociocultural differences in IHD chest pain presentation can partially explain those disparities, which lead to diagnosis and treatment delays. The women’s ischemic symptoms are more often related to emotional or mental stress and less frequently precipitated by physical activity as compared to those of men. Sex and gender differences in the pain mechanisms, including psychological susceptibility, ANS reactivity, and visceral innervation, might contribute to differences in chest pain presentation. Thus, risk scores and typical/atypical angina characterization do not seem more relevant and should not be used uniformly in women and men.^60^

Differences in chest pain perception between sexes have been described, the female sex having higher somatic awareness as compared to the male sex. This might lead to women’s higher sensitivity, but lower specificity for cardiac chest pain. Premenopausal younger women with relatively high estrogen levels have a higher perception of pain as compared to postmenopausal older women with lower estrogen levels.^62^

A comprehensive assessment of sex and gender differences in pain includes proximate cause contributions of experiential (abuse, labor, and delivery), psychological (anxiety, depression, post-traumatic stress), genetic (X chromosome imprinting/Y chromosome), neurochemical (adenosine, cytokine expression), organizational (steroid action in development), activational (steroid action in adulthood), systems level (cortical connectivity, vagal nerve modulation), and sociocultural (gender roles, gender role expectations) aspects.^62^

Ischemia triggered by mental stress predicts a twice higher mortality, and an abnormal autonomic response to stress leads to an increase in coronary vascular reactivity.

In addition to the CVD manifestations, the clinical history of chest pain in women should include the classic RFs, the female sex-specific RFs, and the psychosocial determinants of health, notably depression and stress. Usually, the myocardial ischemic pain is reported in the chest, but can occur in any region from the epigastrium to the jaw or teeth, between the scapulae, or in an arm radiating to the wrist or fingers.^63^

Women usually have pain characteristics similar to those of men, but have a higher prevalence of other symptoms, such as palpitations, dyspnea, maxillary, cervical, and dorsal pain. A substudy of the High-STEACS clinical trial (high-sensitivity troponin in the assessment of patients with ACS; 1941 patients, 39% of whom women) has assessed chest pain prospectively and with descriptors of typical nature, presence of radiation, and additional symptoms more common in women with suspected ACS. As compared to men, women more frequently reported palpitations as the initial symptom (11% *versus* 7%), pain radiation to the left arm (36% *versus* 31%), dorsum (31% *versus* 17%) or neck/jaw (28% *versus* 20%), and higher propensity to nausea (34% *versus* 22%).^64^

Dyspnea can accompany pain or chest discomfort, and less specific symptoms, such as fatigue or fainting, nausea, burning, restlessness, or imminent death sensation, can occur. The discomfort duration is short, usually less than 10 minutes.^65^ A peculiar aspect of the ischemic pain manifestation in women is the sensation of chest discomfort, even in severe ACS. Despite individual variability, the suffering induced by myocardial ischemia is usually characteristic, and, thus, fundamental to the diagnosis. The most likely criteria to be associated with ischemia have been described as “typical”, more common in men, and as “atypical” (nauseas, discomfort, epigastralgia), more common in women. However, the denomination “atypical” pain as a female characteristic should be avoided, and the assessment of women’s myocardial ischemia symptoms should be standardized. In addition, “atypical chest pain” is a questionable term because, despite the intention to indicate absence of “typical” symptoms, it suggests a noncardiac origin, and, thus, its use is discouraged.^59^

A better understanding of the female anginal symptoms and a more appropriate risk stratification for women enable early recognition, correct diagnosis, and ideal treatment, improving the IHD prognosis and course in women.

In addition, “chest pain” means pain, pressure, crushing or discomfort in the thorax, shoulders, arms, neck, dorsum, upper part of the abdomen, jaw, as well as dyspnea and fatigue, which should be considered anginal equivalents. Thus, these are unspecific symptoms, even described as weakness, dizziness, perspiration, and dyspepsia, and can be manifestations of myocardial ischemia in women.^66^Chest pain or discomfort occurs in more than 80% of women and men with ACS; women, however, more often attribute this symptom to noncardiac conditions, such as reflow, stress, or anxiety, hindering the diagnosis.^67^Women have more additional symptoms than men do, and are more likely to present without chest pain.

The ACS presentation forms are different in women, who, thus, are at a higher risk for misdiagnosis, as well as for diagnosis and treatment delays, which can lead to catastrophic events, such as cardiac arrest. Patients with dyspnea of cardiac origin as the single manifestation are known to be at a twice higher risk for sudden death.^68^

Women with ACS are more likely older, having, thus, more comorbidities than men. In addition, women have continuous symptoms, try self-medication, and usually report similar symptoms in the past.

Young age and absence of chest discomfort are some of the strongest predictors of ACS misdiagnosis and early or inappropriate discharge from the emergency sector. Women discharged with the diagnosis of unspecific chest pain can have twice more subsequent coronary events.^67^

The risk scores created to stratify patients are not perfect, notably for women, whose emerging and specific RFs are not considered. The HEART score performed better than the TIMI, GRACE, and EDACS scores to predict adverse events in emergency patients presenting with chest pain or anginal equivalents, but 5.7% of the patients classified as low risk had events.^12^In the HEART score, the classical considerations for stratification are *H*istory, *E*CG, *A*ge, *R*isk factors, and *T*roponin, and the score is the addition of those five variables. Women have variable clinical presentations, frequent dubious ECG changes, and female sex-specific RFs, thus, that and other scores have a reduced power to stratify them.^68^

Knowing the RFs enables the health professional to use preventive strategies that can, in the medium and long run, change the CVD prognosis and outcome.^69^

The anatomical and metabolic differences between sexes should be considered when studying the risk groups. The relevance of the RFs differs, therefore, studying them separately is important.^69,70^ It is worth noting that the well-established RFs (modifiable and nonmodifiable), as well as the female sex-specific RFs, should be considered. Some factors of impact on women’s cardiovascular health, such as depression, domestic violence, and psychosocial factors, are underrecognized and should be assessed.^32^ Usually, the RFs manifest together, and the higher the number of RFs, the higher the patient’s overall CVR.^29^

### 4.2. Established and Modifiable Risk Factors

• **Obesity/overweight:** Obesity is a disease, and, for women at reproductive age, it is the modifiable RF more directly associated with the presence of SAH. Obesity in pregnancy increases the risk of developing hypertensive disease of pregnancy and/or gestational diabetes, which play an important role in maternal and fetal mortality. In the Framingham Heart Study, obesity increased the risk of coronary disease by 64% in women and by 46% in men.^29,71^
• **Sedentary lifestyle:** This is the RF with direct impact on CVD since early childhood.^29^ From the first years of life, women participate less and less in physical activity, which leads to a cascade of changes produced by the lack of physical conditioning, with significant clinical relevance in the unhealthy aging process.^29,67^ In countries where women live under social/religious restrictions, the cardiovascular data are worse, hindering health promotion and increasing the prevalence of other RFs, such as obesity.^29^
• **Smoking:** Is an epidemic habit. Its prevalence in men is higher than in women; however, recent studies have shown that the CVR associated with smoking is 25% higher in women than in men.^29^ It is worth noting that electronic cigarettes have been consolidated as a popular practice, reaching more and more young women worldwide, contributing to the formation of endothelial lesions. In all age groups, except for the 30-44 year age group, women are at a 25% higher risk of IHD associated with smoking as compared to men.^29,67^
• **Dyslipidemia:** Is a RF related to lifestyle and genetic factors, but studies have shown lower attention to women’s health and therapeutic proposals as compared to those of men. An aggravating factor to consider is menopause, because of the relevant increase in total cholesterol and LDL-cholesterol concentrations and smaller protective effect of HDL-cholesterol, thus leading to a higher predisposition to CVD. Treatment strategies are well established in the guidelines, not differing from those for men. However, it is worth emphasizing that atheroma regression and benefit from LDL-cholesterol reduction seem higher in women than in men.^29,71^
• **Diabetes mellitus:** Women with DM are at a three-fold higher risk of fatal IHD than women without DM. Myocardial infarction occurs earlier and has higher mortality in women than in men with DM. Women with DM have a significantly higher risk of developing chronic arterial disease and/or HFpEF than men.^67^ Considering the worldwide increase in DM, directly related to the increase in obesity, women with DM have a 44% higher incidence of IHD than men.^29^
• **Stress:** Mental health is a subject of great relevance, mainly after the COVID-19 pandemic. The relation between stress and CVR has been well established, and anxiety and depression are associated with an increase in morbidity and mortality from CVD.^29,67^Domestic violence, which affects a large part of the female population, is the triggering factor of significant chronic stress, leading to sequelae that persist after the end of the abuse, causing other mental disorders.^29^
• **Systemic arterial hypertension:** Is the major RF for women. Hypertensive women are at higher risk of AMI as compared to hypertensive men because of the higher velocity of disease progression, higher negligence in diagnosis, and lower adherence to treatment.^29,70^ Although the SAH guidelines have been well established, the impact of establishing different BP goals according to sex has been recently discussed.^29^

### 4.3. Established and Non-Modifiable Risk Factors

• **Family history:** Women under the age of 65 years with maternal history of AMI have a 4-fold higher risk of ACS than men at the same age or older women.^67^
• **Age:** Age propitiates the development of metabolic syndrome, which conceptually associates with the major RFs – DM, SAH, obesity, and dyslipidemia^70^ and increases the insulin resistance as well as the pro-inflammatory and prothrombotic status, compounding the risk for CVD.^29^

### 4.4. Women-Specific Risk Factors

• **Menopause:** Estrogen is well known for its protective effect on the vascular endothelium. In menopause, the absence of estrogen protection has negative effects on the cardiovascular function/metabolism, such as: body fat change, vascular inflammation, endothelial dysfunction, sympathetic tonus increase, and higher insulin resistance.^67^ However, hormone therapy in menopause is not indicated for the primary or secondary prevention of CVD, its use being indicated for replacement in early menopause. In addition, in the presence of important vasomotor symptoms that hinder the quality of life, hormone therapy can be used, but individual interprofessional assessment is required to ensure treatment safety and efficacy.^70,71^
• **Pregnancy-related disorders:** Women are subject to complications, such as gestational diabetes and gestational hypertensive disease, which increase the risk of ACS by three to four times throughout life.^70^ Thus, their obstetric history should be carefully investigated, and the presence of those comorbidities indicates the need for continuous follow-up. Women with preeclampsia are at a 3.7-fold higher risk of developing SAH 14 years later, 2.16-fold higher risk of developing IHD in 12 years, 1.8-fold higher risk for stroke in 10 years, and 1.78-fold higher risk for venous thromboembolism in 5 years. Gestational diabetes increases the risk of type 2 DM by 7 times, the risk of stroke by 2 times, and of myocardial infarction by 4 times, independently of the patient becoming diabetic or not.^29,71^
• **Oral contraceptive hormones:** The use of oral contraceptive hormones doubles the risk of ACS of atherothrombotic etiology, which is compounded by smoking, SAH, and DM.^29^
• **Polycystic ovary syndrome:** By promoting excessive androgens and oligoanovulation, polycystic ovary syndrome (POS) increases the likelihood of developing dyslipidemia, SAH, and DM.^29^
• **Systemic inflammatory and autoimmune disorders:** The chronic inflammation of some diseases, such as systemic lupus erythematosus and rheumatoid arthritis, leads to endothelial dysfunction and higher predisposition to CVD. Women have a higher prevalence of such disorders as compared to men. The women to men ratio for rheumatoid arthritis is 2.5:1, and for systemic lupus erythematosus, 9:1. For women with rheumatoid arthritis, the risk of AMI is 2-3 times higher, and of stroke, 50% higher. In systemic lupus erythematosus, the risk of AMI is 9-50 times that of the general population. The usual risk scores underestimate the CVR in women with rheumatoid arthritis and systemic lupus erythematosus, suggesting multiplying by 1.5 the absolute risk.^29,71^
• **Radiation and chemotherapy to treat breast cancer:** Radiation therapy for breast cancer involves heart exposure to ionizing radiation, increasing the risk of IHD. This risk is proportional to the dose, begins a few years after exposure, and continues for 20 years. It is associated with the presence of RFs and is higher in the left breast radiation as compared to the right one. It can manifest as valvular heart disease or cardiomyopathies.^29,69^

At least 20% of women have a coronary event with no well-established RF for CVD.^69^ Women should be assessed in a particular way to identify and control RFs, both the well-established and specific ones, whose knowledge is crucial for the prevention of CVD and health promotion in women’s longitudinal follow-up.^29^

### 4.5. Underrecognized Risk Factors

• Some RFs, such as anxiety/depression disorders, and social determinants of health, such as sexual abuse and violence, socioeconomic deprivation, and low education level, are considered CVD enhancers, and their investigation in women’s risk stratification is important. In addition, the social determinants of health lead to negative outcomes, such as vascular inflammation and endothelial dysfunction, mainly when associated with the classic RFs.^32^
• Anxiety/depression disorders increase by two times the risk of IHD, because they change the hypothalamus-pituitary-adrenal axis and ANS, increasing oxidative stress and inflammatory response, with consequent endothelial dysfunction, atherothrombosis, and IHD. Young women have a higher risk for depression and higher mortality after AMI than men, as well as a lower decline in death rates than men.^29^

#### 4.5.1. Recommendations

The specific recommendations on the CVRFs are described in the Position Statement on Women’s Cardiovascular Heath.^32^

## 4.6. Drug Treatment in the Different Forms of Ischemia Manifestation

As compared to men, women, including the younger ones, have a greater delay in ACS diagnosis and treatment. Therefore, they less often undergo coronary angiography and surgical treatment,^72^ including mechanical circulatory support in cardiogenic shock, and their probability of in-hospital death is higher.

The lower surgical treatment rate in women seems to be due to the presence of MINOCA, small-caliber arteries, and less severe atherosclerotic disease. Women have a higher prevalence of other pathophysiological mechanisms of IHD, such as Takotsubo cardiomyopathy (stress-induced), coronary microvascular dysfunction, coronary embolism, coronary vasospasm, and SCAD. However, this does not explain why women less often undergo coronary angiography and circulatory support in cardiogenic shock.^72^

INOCA is more common in women (> 50% *versus* 7-17% in men) and associates with the risk of unfavorable outcomes, such as recurrent hospitalizations, interventions, worse quality of life, higher mortality, and 5- to 10-times higher risk of developing HFpEF. Thus, its diagnosis and prognosis, as well as its management, continue to be a challenge for physicians.^73^ Although INOCA associates with the risk for major events, less than 50% of the female patients undergo appropriate drug treatment, adherence to prescribed treatment is low, and cardiac rehabilitation is underused.^74^ It has been suggested that women less often undergo drug therapy because of the lack of evidence about their treatment, mainly in the presence of associated coronary microvascular dysfunction.^75,76^ Another factor limiting the consensus on INOCA treatment is the presence of different phenotypes of INOCA, either predominating coronary microvascular dysfunction, or coronary vasospasm, or both. Thus, the therapeutic responses are varied and uncertain.

The INOCA/MINOCA treatment is based on three pilars:^77^

1. Lifestyle change
2. Control of CVRF
3. Antianginal treatment

Lifestyle change, control of CVRF, such as SAH, DM, and dyslipidemia, and cardiovascular rehabilitation are fundamental measures to reduce morbidity and mortality in INOCA/MINOCA,^73^ as well as in coronary disease.

Randomized studies, such as WISE and WARRIOR (ongoing), have shown the efficacy of the angiotensin-converting enzyme inhibitor (ACEI) in CFR and in anginal improvement. Thus, hypertensive female patients with INOCA/MINOCA can benefit from its use.^73^

Because of their inhibitory effect on oxidative stress and anti-inflammatory properties, statins act on CFR and coronary microvascular dysfunction, improving tolerance to exertion and reducing exertion-induced ischemia, mainly when associated with diltiazem.^78^

Antianginal drugs, such as nitrates, beta-blockers (BB), and calcium-channel blockers (CCB), are used in INOCA and MINOCA, but their efficacy varies according to the predominant phenotype (coronary microvascular dysfunction, coronary vasospasm, or both).^77^ In patients with coronary microvascular dysfunction, long-acting nitrates can worsen symptoms, because of the possible coronary steal phenomenon, thus, CCB should be preferred. Short-acting nitrates have uncertain efficacy and require repeated doses.

For coronary vasospasm, CCBs are first-choice drugs, being followed by nitrates. For coronary microvascular dysfunction, reduced CFR and/or increased microcirculatory resistance, suggesting arteriolar remodeling, BBs are used initially, and, for refractory cases, CCBs. Ranolazine and trimetazidine can be considered in the treatment of nonobstructive IHD, mainly for refractory angina.

Nebivolol is the most often used BB because of its B1-selective action and vasodilator effect via the nitric oxide production. It has been studied in women with coronary microvascular dysfunction, improving angina and tolerance to physical exertion. Its intracoronary infusion improves CFR and reduces myocardial oxygen consumption (VO_2_). In addition, it improves the LV filling pressure and CFR in patients with uncomplicated SAH, possibly because of the nitric oxide action on microcirculation.^78^

Promising drugs have been studied for INOCA, such as nicorandil and trimetazidine. The former is a potassium channel activator that leads to microcirculation vasodilation similarly to nitrates. Trimetazidine acts in the metabolism of cardiac muscle cells by inhibiting the oxidation of free fatty acids, increasing the use of glucose in the production of high-energy phosphates. Trimetazidine in monotherapy or in combination with antianginal drugs has improved tolerance to exertion in patients with stable angina and INOCA. In addition, improvement in myocardial perfusion and endothelial function has been described, probably due to a reduction in oxidative stress. A single dose of trimetazidine increases exercise duration and the signs and symptoms of ischemia on exertion ECG. Recent studies have evidenced metabolic cardioprotection with trimetazidine for patients with stable angina and ACS after PCI.^73,78^

Ranolazine inhibits late calcium channels, reducing their concentration inside cardiomyocytes, thus improving the relaxation function and microcirculation. It seems to have a favorable action on women with INOCA and patients with reduced CFR.^73,78^

Considering that women have mental or emotional stress as triggers for ischemic pain, as well as higher psychological susceptibility and ANS reactivity, they might benefit from low doses of tricyclic antidepressants to help reduce symptoms.^77^

In conclusion, women should be managed with a differentiated approach from the cardiovascular viewpoint, considering the peculiarities of IHD clinical manifestations due to different pathophysiological mechanisms, which hinder the diagnosis, and, thus, the therapy. Therefore, those factors can be deleterious to health. The RF analysis should be comprehensive, including enhancers of RFs, in addition to the women-specific RFs and the underrecognized ones.

### 4.6.1. Recommendations

The treatment of INOCA/MINOCA is based on lifestyle changes, RF control, and antianginal treatment. For hypertensive women, ACEI/angiotensin receptor blocker (ARB) should be preferred. Statins act on CFR and coronary microvascular dysfunction, improving tolerance to exertion and reducing exertion-induced ischemia. Nitrates, BBs, and CCBs can be used as antianginal drugs; however, in patients with coronary microvascular dysfunction, long-acting nitrates can worsen symptoms, because of the possible coronary steal phenomenon, thus, CCB should be preferred. For coronary vasospasm, CCBs are first-choice drugs. In cases of arteriolar remodeling (coronary microvascular dysfunction, reduced CFR and/or increased microcirculatory resistance), BBs should be initially used (nebivolol is the most often used BB because of its B1-selective action and vasodilator effect via nitric oxide production) and, in refractory cases, the CCBs. Ranolazine and trimetazidine can be considered for refractory angina.^78^

Low doses of tricyclic antidepressants can help reduce symptoms in women with mental or emotional stress as a trigger of ischemic pain.^77^

## 5. Diagnosis through Graphic Functional Assessment

### 5.1. Electrocardiogram at Rest

Women’s ECG have differences as compared to that of men, such as higher HR at rest, QRS with smaller amplitude and longer duration, longer QT intervals, and ST-segment changes.^79,80^

For IHD assessment, the correct position of electrodes is fundamental to avoid misdiagnosis. Large breasts or breast prostheses generate low-voltage complex and reduce the R-wave amplitude in the V1 and V2 leads, simulating inactive area. In addition, women who underwent mastectomy have QRS widening, simulating LV hypertrophy.^80^

On women’s chest pain assessment, the ECG maintains the diagnostic criteria described in the male sex, except for the subepicardial lesion. In women, J-point elevation and ST-segment values ≥ 1.5mm in the precordial V1 to V3 leads are considered pathological.^80^ Other variables, such as greater QRS-T angle and longer QRS duration, can be considered independent predictors of death, HF, and nonfatal AMI.^81^Postmenopausal ventricular repolarization abnormalities can be important predictors of coronary events and mortality from IHD.^82^

### 5.2. Exercise Testing

The ET is an accessible, safe, reproducible, low-cost, radiation-free functional test that can analyze the cardiovascular response to exertion, providing clinical, hemodynamic, metabolic, autonomic, and electrocardiographic information to support the diagnosis, risk stratification, therapeutic assessment, and physical exercise prescription.^83^

The multifactorial view of the ET has widened its interpretation by including prognostic variables, mainly in women. It is recommended as the first-choice method to assess symptomatic women at intermediate risk for IHD, with normal ECG at rest, and who can exercise.^79,84^

In a meta-analysis of 19 studies, the mean sensitivity of ET was lower in women than in men (61% *versus* 70%), as was its specificity (72% *versus* 77%).^1^ In symptomatic women, the positive predictive value of the ST-segment depression was lower than in men (47% *versus* 77%, p<0.05); however, its negative predictive value was similar (78% *versus* 81%). A maximal and negative ET is useful to rule out the presence of significant obstructive IHD and to predict excellent event-free survival.^79^

In women, the prevalence of electrocardiographic changes without correspondence with obstructive IHD is higher.^83^ The factors that contribute most to lower diagnostic accuracy include: lower prevalence of multivessel IHD; higher prevalence of nonobstructive IHD, mitral valve prolapse, vasospastic and microvascular angina; low-voltage ECG and ventricular repolarization changes; predominantly atypical symptoms; pre- and postmenopausal hormonal factors; lower tolerance to exertion and achieved HR.^79,83^

The ST-segment changes with slow or convex ascending morphology, mainly restricted to the inferior leads, which appear at high HR, higher work load and rapidly normalize in recovery (within the first minute), tend to not correlate with significant obstructive IHD, differently from changes ≥ 2.0mm, with horizontal or descending morphology, low loads and HR, persisting for several minutes in recovery, mainly if accompanied by symptoms.^85^ In addition to ST-segment changes, the ET contributes with important prognostic information through the analysis of parameters, such as physical functioning, chronotropic and BP responses, HR recovery, and assessment of scores.

Physical functioning is the major independent predictor of death in women, including the asymptomatic ones.^86^The ability to reach ≥ 10 MET indicates very low prevalence of significant myocardial ischemia, independently of the gender.^87^However, when < 7 MET are reached, the probability of ischemia is significantly higher (0.4% *versus* 7.1%).^88^ The inability to reach 5 MET is an independent predictor of high risk, with a three-fold increase in mortality as compared to women reaching > 8 MET.^89^

The inability to reach 85% of the predicted maximal HR for age predicts increased risk for mortality and obstructive IHD in women.^79^ The presence of chronotropic incompetence increased by 30% the risk of all-cause mortality.^89^ A HR reduction in the first minute of recovery < 12 bpm is another important prognostic marker, being an independent predictor of all-cause mortality.^79^

The BP increase is smaller, mainly in younger women, and non-elevation (“plateau”) is a frequent finding, usually not related to cardiac disease, which differs in the male sex. Its interpretation should be associated with other variables.^89^

Scores can be useful to improve the IHD diagnosis, the Duke treadmill score being the most frequently used, with diagnostic and prognostic value. The DTS is calculated as follows: exercise duration (Bruce protocol) – 5x (ST-segment deviation) – 4x (angina, with: 0 = none; 1 = nonlimiting pain; 2 = exercise-limiting pain).^90^The score categories are: low risk (DTS ≥ 5), moderate risk (DTS ranging from -10 to 4), and high risk (DTS ≤ -11).^91^ Low DTS is associated with an annual mortality rate of ≈ 0.25% as compared to ≈5% for high risk, and the mortality rates are lower in women. In a cohort of 976 symptomatic women with ET and angiography, coronary stenosis ≥ 75% was present in 19%, 35%, and 89% of those with low-, moderate-, and high-risk DTS, respectively.^92^ Women with intermediate DTS should be referred for additional risk stratification with stress imaging.^79^ The DTS is a valuable tool to predict the risk of events (cardiac death, nonfatal AMI, and late revascularization) in women, but might be less effective in those aged ≥ 75 years.^93^

Table 5.1 shows the indicators of elevated risk in the women’s ET.

**Table 5.1:** – Indicators of elevated risk that should be valued and reported in women’s exercise testing.

Variable	High-risk indicators
Functional capacity	< 5 METs
ST-segment changes	Depression ≥ 2mm Depression ≥ 1mm with < 5 MET or > 5 min in recovery Elevation ≥ 2mm (in leads with no Q wave or aVR)
HR decline in the first min of recovery	≤ 12 bpm
Duke treadmill score	≤ -11
BP response	Reduction > 10mmHg as compared to rest
Ventricular arrhythmias	Persistent VT /fibrillation

*Adapted from Mieres et al.^79^ HR: heart rate; BP: blood pressure; VT: ventricular tachycardia.*

In situations with high likelihood of CVD, orthopedic diseases, obesity, and sedentary lifestyle, the association with myocardial perfusion scintigraphy (MPS) can be useful in the presence of nonconclusive test results.

#### 5.2.1. Recommendations

Individualized assessment and directed anti-ischemic therapies after ET should be based on the load reached, the symptoms, and the test’s grade of abnormality.

The choice of the diagnostic method should be based on the patient’s clinical profile, pretest probability of CVD, symptoms, ECG findings at rest, and presence/absence of proper conditions to perform good-quality ET. The analysis should be multifactorial to optimize the clinical management and therapy.

## 5.3. Cardiopulmonary Exercise Test

The CPET provides noninvasive and integrated assessment of the cardiovascular, respiratory, and peripheral systems on exertion. Although CPET has been well-established for the clinical assessment of patients with HF, its indication for IHD investigation has not been consolidated in the guidelines. In a review article on the applicability of CPET in IHD, the following indications have been established:^94^

1. Moderate to high pretest probability of IHD
2. To increase the IHD diagnostic accuracy in the presence of clinical or electrocardiographic changes that hinder the diagnosis on ET
3. Large myocardial ischemic area that hinders LV function
4. Assessment of the evolution after mechanical or surgical MRV.

In addition to the assessment of clinical, hemodynamic, and electrocardiographic criteria, the CPET provides additional information on the ventilatory parameters in the analysis of ischemia, quantifies physical functioning precisely, and stratifies the prognosis. Low physical functioning [VO_2_ < 60% of the predicted value for age and sex] and myocardial ischemia signs and symptoms at low load (VO_2_ < 15ml.kg^-1^.min^-1^) stratify patients as at high risk for cardiovascular events.^95^

In patients with macrovascular IHD and reversible perfusion defects on myocardial perfusion imaging, the assessment of CPET variables, as compared to those of ET, increased sensitivity from 46% to 87% and specificity from 66% to 74%.^96^

The CPET identifies the beginning of LV dysfunction induced by ischemia during exertion, and, thus, can improve diagnostic accuracy, mainly in the female sex, reducing the rate of false-positive ET.^97^ This is particularly important, because, in the myocardial ischemic cascade, changes in the LV diastolic and systolic functions occur before the ischemic changes on ECG.^98^

The major variables obtained during CPET that evidence LV dysfunction triggered by IHD are: VO_2_ curve and ascension, oxygen pulse, and cardiac work rate (VO_2_ variation/load variation in *Watt*), the latter exclusively on cycle ergometer. A linear VO_2_ increase in parallel with a workload of 10 mL/min/*watt* under physiological conditions is expected.^94,99^

The analysis of oxygen pulse improves the diagnostic accuracy of IHD. Oxygen pulse is assessed as follows: the value obtained at peak exertion in relation to the predicted one, as well as the morphology of the oxygen pulse curve over time, which should show an increasing trend, most commonly in the shape of a parabola (Figure 5.1).^94,99^ In the presence of obstructive IHD, the reduced systolic volume causes a compensatory increase in HR that impacts both the cardiac work rate and oxygen pulse. A reduction in the VO_2_/load curve progression and a decline in or absence of progressive increase in oxygen pulse are seen (Figures 5.2 e 5.3). In the presence of an early plateau or, mainly, decline of oxygen pulse during exertion, the prescription of exercise intensity can be limited to loads below that threshold.^99,100^

**Figure 5.1:** – Ascending behavior of the oxygen pulse curve over time. HR: heart rate; RCP: respiratory compensation point; AT: anaerobic threshold.

**Figure 5.2:** – Descending behavior of the oxygen pulse curve over time. HR: heart rate; RCP: respiratory compensation point; AT: anaerobic threshold.

**Figure 5.3:** – Oxygen pulse plateau. HR: heart rate; RCP: respiratory compensation point; AT: anaerobic threshold.

A substudy of the ORBITA-Trial has assessed the CPET parameters related to myocardial ischemia and anginal symptoms in patients with chronic coronary syndrome. The oxygen pulse plateau was the only variable that could detect the ischemia severity and predict the PCI efficacy in severe single-vessel IHD.^101^

Recent studies have assessed the role of CPET in coronary microvascular dysfunction, particularly in women, whose ET is less accurate. A study in postmenopausal women with typical angina and nonobstructive IHD on angiography has shown that ischemia on scintigraphy was associated with LV systolic dysfunction after stress, suggesting microvascular IHD. Considering the CPET potential to infer LV dysfunction in a noninvasive and cost-effective way, the CPET implementation, as compared to that of ET, in the microvascular IHD investigation increases the diagnostic potential.^102,103^ In addition, CPET can be an important tool to assess patients with left bundle branch block (LBBB) because of the limitation of ET in that population. Normal VO_2_ and oxygen pulse curves could rule out significant coronary stenosis as the cause of LBBB.^99^

Some limitations of CPET, such as restriction of the cardiac work rate assessment to only cycle ergometer, lower sensitivity in single-vessel IHD, and associated clinical conditions that can interfere with VO_2_ (anemia, pulmonary disease, HF), are worth noting.^99^ The CPET is a valuable tool in daily clinical practice, but further investigations should be directed to a more accurate assessment of its role in IHD diagnosis, prognosis, and treatment efficacy evaluation, mainly in women.

## 6. Diagnosis by Use of Ergometric-Functional Evaluation

### 6.1. Introduction

Noninvasive imaging methods play a fundamental role in the diagnosis and management of IHD. In women, especially, there are particularities regarding the etiology of myocardial ischemia involving obstructive IHD and coronary microvascular dysfunction.^104^ This chapter addresses several cardiovascular imaging modalities, with important information on the assessment of women’s myocardial ischemia symptoms, defining diagnostic performance, female sex-related peculiarities, as well as advantages and limitations of each method (Figure 6.1). The sensitivity and specificity values of the major diagnostic methods for men and women, according to a recently published review, are shown in Table 6.1 and Figure 6.1.^8^

**Figure 6.1:** – Importance of noninvasive imaging methods in women’s ischemic heart disease. CAC: coronary artery calcium score; CCTA: coronary computed tomography angiography; CMRI: cardiac magnetic resonance imaging; HR: heart rate; IHD: ischemic heart disease; INOCA: ischemia with nonobstructive coronary arteries; LV: left ventricular; MINOCA: myocardial infarction with nonobstructive coronary arteries; NM: nuclear medicine; PET: positron emission tomography; RF: risk factor; SEC: stress echocardiography; SPECT: single photon emission computed tomography; VUS: vascular ultrasound (carotid).

**Table 6.1:** – Sensitivity and specificity according to sex of the major diagnostic methods.

Modality	ET	SEC	SPECT-MPS	PET-MPS	CCTA	CMRI
Sensitivity						
Women	43-71%	70-96%	84-91%	81-100%	90-98%	84-91%
Men	68-80%	70-80%	85-94%	81-92%	90-97%	90-91%
Specificity						
Women	64-85%	79-92%	58-91%	86%	84%	81-88%
Men	74-83%	84-93%	62-89%	85-89%	83%	74-94%

*CCTA: coronary computed tomography angiography; CMRI: cardiac magnetic resonance imaging; ET: exercise test; MPS: myocardial perfusion scintigraphy; PET: positron emission tomography; SEC: stress echocardiography; SPECT: single photon emission computed tomography.*

### 6.2. Echocardiography at Rest and Under Stress

Transthoracic echocardiography (TTE) at rest or using several modalities of stress (physical exercise, administration of inotropic drugs or vasodilators) is useful to detect myocardial ischemia in women.^104^ The technique is particularly appealing because it involves no radiation to the breast tissue, especially for younger women, considering their expected cumulative exposure throughout life, when serial studies might be necessary. In addition, the method has good accuracy.^104,105^

Findings compatible with myocardial ischemia include: abnormalities of the segmental LV wall contractility at rest, altered contractile response during stress, reduced microvascular perfusion assessed on contrast echocardiography and/or impairment of the flow of epicardial arteries analyzed by use of Doppler.^79,106^ In addition, TTE is useful to identify other causes of chest pain, such as pericarditis, aortic dissection, and pulmonary embolism.^59,79^

The TTE at rest is recommended for all female patients with suspicion of IHD to assess segmental contractility, as well as the global LV systolic function by use of LV ejection fraction (LVEF).^59,106^ The presence of regional contractile abnormalities can be chronic or acute, an issue better solved if previous studies are available for comparison.^106^ It is worth noting that the segments with changes due to IHD should be contiguous and correspond to the territories of coronary arteries, because segmental changes can be caused by several conditions other than IHD.^106^

The LV segmental contractility is assessed visually based on wall thickening during the systole. More recent quantitative methods have been applied, such as the left ventricular global longitudinal strain (LVGLS) analysis. Subendocardial muscle fibers, longitudinally oriented, are more vulnerable to ischemia, and the LVGLS assessment at rest has shown to be more sensitive for the detection of segmental abnormalities as compared to the analysis of wall contractility in ACS.^106^

The preferred modality for ischemia assessment on SEC is physical exertion, especially with supine bicycle. In addition to providing additional prognostic data, such as physical functioning, that method comprises more physiological data for ischemia assessment.^79^ When compared to ET, SEC has higher sensitivity and specificity in women.^59,79^ A meta-analysis of 14 studies, assessing the sensitivity and specificity of SEC with physical stress for the detection of women’s IHD, has reported values of 79% (95% CI, 74%–83%) and 83% (95% CI, 74%–89%), respectively.^107^

The SEC with dobutamine is preferably indicated for patients who cannot exercise.^79^Vasodilators, such as dipyridamole or adenosine, have slightly lower sensitivity in women.^108^In a comparison between SEC with dobutamine and ET for the detection of IHD in symptomatic women, the former showed higher accuracy in women with chest pain, with sensitivity of 70.4% *versus* 53.7% and specificity of 94.6% *versus* 73.6% to detect coronary stenosis >50%. The higher accuracy of SEC persisted after excluding the patients who reached HR higher than 85% of that predicted for age before ischemia induction.^109^ The results are different when comparing the diagnostic performance of SEC between men and women.^8^ The literature shows that the performance in women is similar or inferior to that in men.^11,109^ When compared to SPECT for the investigation of myocardial ischemia, SEC has higher specificity, because it has no false positive due to breast attenuation.^8^

The SEC can be limited by the impossibility of obtaining appropriate acoustic windows in the peak of stress,^104^ particularly in women, because of the breast tissue. The use of echocardiographic contrast media has become an important tool to properly visualize the endocardium of all LV segments. Thus, in the presence of two or more contiguous segments with limited technical quality, the use of those agents is indicated.^110^

In pregnancy, echocardiography with physical or pharmacological stress is considered safe and avoids exposure to radiation; dobutamine and dipyridamole are considered category B. Nuclear medicine techniques should be avoided.^1^

### 6.3. Vascular Ultrasound

Atherothrombosis is a systemic arterial disease that involves mainly the intima layer of large- and medium-caliber arteries (carotid, aorta, coronary, and peripheral arteries). The VUS is an excellent method for the morphological assessment and severity classification of carotid obstructive disease, abdominal aortic aneurysm, and PAD. In addition, VUS is an important tool to diagnose atherothrombosis in other arterial territories affected simultaneously with the coronary territory, being particularly appealing because of its low cost, wide availability, and noninvasive character.^111^

The detection of subclinical disease and information on the characteristics of the atherosclerotic plaque and burden are useful in some scenarios of women’s atherosclerotic disease. The presence or absence of plaque can re-stratify patients beyond the traditional RFs, while the atherosclerotic burden quantification can personalize even more the risk assessment.^32,112-114^ The plaque identification and atherosclerotic burden measurement in the carotid and/or peripheral arterial territory can be useful to reclassify the risk, as shown in recent studies.^115^The analysis of the characteristics and volume of the plaque on 3D seems to significantly improve the atherosclerotic burden estimation and identification of more vulnerable plaques, and can play a role in women’s risk stratification, because women seem to have a higher atherosclerotic burden than men.^114-116^ In addition, the use of contrast-enhanced VUS is an attractive tool to assess neovascularization in carotid plaques, because women have more signs of neovascularization.^117^

Peripheral arterial disease is prevalent in men and women, and its subclinical form is more common in women. Patients with previous preeclampsia, gestational hypertension, and placental insufficiency are three times more likely to develop PAD.^118^ Recently, the assessment of carotid and femoral atherosclerosis has been suggested to improve the early detection of disease, and the presence of a femoral, rather than a carotid, plaque is more indicative of IHD. In women, the combined femoral and carotid plaque areas have higher sensitivity and can aid in the identification of false-positive stress tests.^119^

Women have a lower prevalence of abdominal aortic aneurysm and at a more advanced age. However, women are four-times more likely to undergo rupture as compared to men at the same age and have worse results after emergency surgery. The sensitivity of the physical examination is 29% in abdominal aortic aneurysms with diameter of 3-3.9cm; 50% in those with diameter of 4-4.9cm; and 75% in those with diameter over 5cm, justifying that a large part of diagnosis is incidental. Thus, in selected women, screening is indicated: age of 55-75 years; family history of abdominal aortic aneurysm; heavy smokers; and/or history of stroke.^120^

### 6.4. Computed Tomography

Particularities of women’s IHD make noninvasive stratification an important step in the management of those patients. The findings of women’s tests differ from those of men’s regarding anatomy, distribution and extension of the lesions, as well as clinical repercussion. In this scenario, assessment of coronary calcification and obstruction grade on CCTA is important to define the patients’ treatment and follow-up.

Anatomically, epicardial coronary arteries in women are smaller than those in men, even after adjusting for age, body mass, and body surface area. In addition, the vasomotor tonus of those arteries is lower, determining higher coronary flow, which, associated with the reduced size of the arteries, can result in different phenotypes of IHD between the sexes.^121^

Despite the anatomical differences, the diagnostic potential of IHD by use of CCTA is similar between the sexes,^122^ and CCTA, as compared to invasive methods, has excellent accuracy to detect obstructive IHD and characterize plaques.^123^ The CCTA sensitivity and specificity values to detect IHD are shown in Table 6.1.^8^

The presentation pattern of women’s IHD is predominantly nonobstructive, and the atherosclerotic burden of nonobstructive IHD (defined as stenosis < 50%) affects women more markedly,^124,125^ with high risk for coronary events as compared to that of the general population, being particularly higher in women < 75 years.^126,127^ Analyses of the CONFIRM registry that assessed 24 775 patients (12 128 women) have shown an increased mortality risk of patients with obstructive and nonobstructive IHD. In women, the risk increased with the increase in the number of vessels with obstructive disease (three-vessel disease: HR: 4.21; 95% CI, 2.47-7.18; p < 0.0001 *versus* HR: 3.27; 95% CI, 1.96-5.45; p < 0.0001) as compared to men.^128^ The presence of atherosclerotic plaques at high risk for thrombosis (low attenuation, punctate calcification, and napkin ring signal) is a predictor of cardiac events in women.^129^In addition, coronary calcification assessed by use of coronary artery calcium score (CAC) differs between the sexes. For patients of the same race and age, CAC quantification is lower in women. Data have consistently shown that the prevalence and severity of coronary calcification increase with age and the male sex, women showing lower prevalence and severity.^130^

New technologies incorporate functional assessment to CCTA, which enables adding the anatomical evaluation to the ischemia investigation. The FFR by computed tomography can increase the diagnostic accuracy in detecting obstructive IHD as compared to CCTA alone. Although promising, the clinical applicability of FFR is reserved to a few centers that provide the technology.^131^

The SCAD, a more frequent complication in women than in men, can be well assessed by CCTA. Pregnant patients have high mortality associated with SCAD. The indication for CCTA in those patients should be individualized, considering the clinical context, and the invasive strategy might be necessary when the clinical suspicion is very relevant.^132^

### 6.5. Cardiac Magnetic Resonance Imaging

Cardiac magnetic resonance imaging has contributed to a better understanding of the pathophysiological phenomena, structural changes, diagnosis, and prognostic assessment of women’s CVD.^132^ The CMRI is a method of high spatial resolution that enables assessment of the morphology, function, and ischemia, in addition to tissue characterization with high accuracy. The fact that it does not have visual limitation due to interposition of the breast tissue, obesity, pulmonary disease, or breast prostheses enables a more detailed assessment of the female heart. In addition, by not using ionizing radiation, it is an innocuous option to the female breast tissue, especially useful for younger women. The female heart has specific morphological characteristics, being relatively smaller than the male heart, with smaller volume, mass and parietal thickness, and can be more accurately assessed by CMRI.^132-134^

Myocardial perfusion assessment with gadolinium enhancement under pharmacological stress has high sensitivity and has been well-established in both sexes since studies, such as MR-Impact, MR-Impact II, and CE-MARC, have shown high sensitivity to detect ischemia as compared to SPECT, being recommended mainly for the female population.^135-137^

In women, coronary microvascular dysfunction is a mechanism responsible for the presence of INOCA,^138^ and CMRI is useful to identify the subendocardial perfusion defect under pharmacological stress in that population.^132,139^ In addition, tissue characterization by use of late enhancement and T1 and T2 maps provides better assessment of the effects of cardiovascular events on the myocardium, more specifically atherothrombotic disease. Thus, myocardial infarction, MINOCA, Takotsubo, and viral or autoimmune myocarditis can be well defined with that methodology.^132,138^

The late enhancement technique has been established as the best way to identify fibrosis *in vivo* and can differentiate ischemic from nonischemic injury, enabling the differential diagnosis between myocardial infarction and inflammatory conditions. The quantification of the fibrotic involvement of the transmural thickness is determinant to assess the myocardial viability grade and the prognosis.^138,140^

Contraindications to the use of CMRI are restricted to situations incompatible with the magnetic field, in addition to the limitations to the use of gadolinium in patients with kidney failure (creatinine clearance below 30 mL/min/BS), because of the risk of nephrogenic systemic fibrosis, and in pregnant patients, because of the risk of fetal exposure to the paramagnetic agent.^131^

The CMRI enables the diagnostic differentiation of cardiovascular events causing anginal symptoms and ischemia in women, thus it is the preferred method for the functional assessment of that group of patients.^137^ The advance of new myocardial perfusion sequences, quantitative perfusion assessment software, movement correction techniques, and higher availability of parametric maps are perspectives that contribute to the increasing use of the methodology. Therefore, CMRI plays a unique promising role in women’s heart assessment, aggregating a greater amount of diagnostic and prognostic information.^132^

### 6.6. Nuclear Medicine

Nuclear imaging can assess the entire spectrum of IHD, from epicardial obstructive IHD to coronary microvascular dysfunction.^141^ National and international guidelines emphasize, for IHD assessment, the use of MPS, which can be performed in all patients, regardless of renal function, presence of arrhythmias, obesity, or intracardiac devices.^79,142^ The techniques used are SPECT and PET.

For over three decades, MPS by the SPECT technique has been widely used in clinical practice because of its wide availability and extensive body of literature that supports its value in the IHD diagnosis and risk stratification.^79,142,143^Its is the most commonly used noninvasive imaging technique to assess women at intermediate-high risk for IHD and stable ischemic symptoms.^141^

One single test provides information on the extension and severity of perfusion abnormalities, myocardial wall motility, LVEF, and intraventricular synchronism at rest and under stress.^79,142,143^ The total perfusion deficit and extension can be calculated, being objective parameters of ischemic burden and myocardium at risk that facilitate the definition of therapeutic management and follow-up.^142^

The ET should be chosen for the stress phase when the patient can exercise properly (reach ≥ 5 MET), because it provides important additional diagnostic and prognostic information. For patients who cannot exercise properly, pharmacological stress with a vasodilator (dipyridamole, adenosine, or regadenoson) should be chosen. Another option of pharmacological stress is the positive inotropic drug use, such as dobutamine, either sensitized or not with atropine. Women with LBBB and intracardiac devices should always undergo pharmacological stress with a vasodilator, regardless of their physical functioning to avoid imaging artifacts.^79,142^

The prognostic value of a normal MPS in women is excellent, including elderly women and several racial and ethnic groups, with 99% event-free survival, similarly to that in men, as shown in a large meta-analysis.^104^

The major disadvantage of MPS is exposure to radiation, although more recent technologies, such as new solid-state CZT gamma-camera detectors, improvement in radionuclides, protocols, and individualized doses of the radiotracer, have reduced that exposure.^143^ False-positive results can occur because of artifact due to breast attenuation in the anterior LV wall, in addition to possible lower accuracy in women with small hearts.

Although less available, the MPS with PET has an excellent diagnostic performance, with sensitivity of 90-92% and specificity of 81-88% to detect angiographically significant stenoses.^104^ In addition to higher accuracy, PET enables the quantification of myocardial blood flow in milliliters per minute per gram of tissue, and the calculation of CFR, an important prognostic marker of CVR.^143^ Thus, PET improves the detection of severe multivessel obstructive IHD with balanced ischemia pattern and coronary microvascular dysfunction, which are difficult to identify with SPECT because of its technical limitations.^142^ Another advantage of PET is the lower exposure to radiation (effective dose of 2-3mSv *versus* 9-12mSv with SPECT), because it uses short-duration radionuclides, which makes it particularly useful for women of reproductive age.^79^ Studies with PET have shown that coronary microvascular dysfunction is prevalent in women and men (54% and 51%, respectively), and regardless of sex, CFR proved to be a powerful incremental predictor of events, a trend maintained even in patients with no coronary calcification.^104^The new CZT gamma-cameras have shown promising results in the CFR assessment with SPECT, increasing the availability of coronary microvascular dysfunction assessment in regions where the PET technique is not available.^141,142^

In addition, abnormal CFR is associated with diastolic dysfunction. A recent study with 64.7% of women without obstructive IHD has shown an independent association of CFR reduction (defined as < 2) and diastolic dysfunction, and an increase in cardiovascular events or HFpEF. Those findings suggest a pathophysiological relation between coronary microvascular dysfunction and HFpEF.^104,144^

Imaging protocols with hybrid equipment (PET/CT or SPECT/CT) add the ability of assessing anatomical changes with CAC quantification, thus increasing the test’s sensitivity for IHD diagnosis with a single test.^104,142^

## 7. Arrhythmias in Ischemic Cardiomyopathy

### 7.1. Atrial Fibrillation and Ischemic Heart Disease

The age-adjusted incidence of AF is 1.5- to 2-times higher in men, but the risk of AF throughout life is similar in both sexes because of the longer life expectancy of the female sex. At the age of 85 years, the differences in prevalence are mild.^1,19-23^ Women have more symptoms and worse quality of life as compared to men. There are innumerous mechanisms associated with the differences between sexes in AF, but it is worth noting that the IHD, more commonly observed in the male sex, can contribute to the higher incidence of AF in the group.

When the rhythm control strategy with AAD is analyzed, women have more adverse events. The increase in the baseline QT interval can affect tolerance to the use of AAD, especially of class III, requiring more careful monitoring in that group of patients. Regarding the results of catheter ablation in the female sex, observational studies have shown that women less often undergo ablation and at a later time, with worse procedural outcome as compared to men.^24^

Innumerous studies have shown that AF treatment based on *“Atrial Fibrillation Better Care (ABC) pathway”* causes significant reductions in the risk of stroke, myocardial infarction, hospitalization, and mortality, with no difference related to sex.^145^ The ABC strategy comprises three aspects: ‘A’ - to prevent stroke; ‘B’ - to provide better control of symptoms with focus on the patient; and ‘C’ - to prevent and/or treat the CVRFs and comorbidities that contribute to the AF appearance.^146^

Regarding ‘C’, women with AF have a higher prevalence of SAH, obesity, depression, HFpEF, and valvular heart disease as the cause of AF.^146,147^ Physical activity is another important predictor in women: the higher the exercise intensity, the lower the incidence of AF. Women who practice vigorous physical activity have a 28% reduction in the incidence of AF.^148^ In a large prospective study with 30 034 women, menopause did not significantly relate to the incidence of AF, while the use of estrogen monotherapy was associated with an increase in its risk, suggesting a pathophysiological relation between estrogen exposure and arrhythmia in women.^149^ As compared to nulliparity, multiparity was associated with a linear increase in the risk of AF in a large cohort of initially healthy women. The repeated exposure to hormonal, metabolic, and physiological changes during pregnancy can predispose to AF later in life.^150^

Regarding ‘A’, women with AF have a higher incidence of stroke than men, and women’s stroke is more severe and causes higher permanent disability, and that is the reason why anticoagulation, based on risk scores, is essential in both sexes.^23,151^ Antithrombotic therapy with oral anticoagulants is indicated for patients with AF and CHA_2_DS_2_VASc ≥ 2, but the decision should be individualized.^146,147^ Patients with indication of continuous use of anticoagulants require risk assessment of bleeding caused by those drugs before initiating their use. A high risk of bleeding does not contraindicate anticoagulation and indicates the need for higher control of RFs. There are innumerous scores for that purpose, and the HAS-BLED score has been recommended in the last guidelines on AF. Individuals with a score ≥ 3 are considered at higher risk of bleeding.^146,147^ Clinical studies on anticoagulation in AF have shown higher safety in the use of DOACs – dabigatran, rivaroxaban, apixaban, edoxaban – in relation to warfarin. The percutaneous occlusion of the LAA has been indicated when there is absolute contraindication to the use of anticoagulants, as occurs in intracranial bleeding of irreversible cause. The surgical occlusion or exclusion of the LAA can be considered when the patient with AF has indication for cardiac surgery.^23,146,147,151-153^

Regarding ‘B’, women with AF have more symptoms, worse quality of life, and higher mortality, justifying the need for a proper and early approach regarding HR control with AADs or ablation of the atrioventricular node and, when possible, reversion to sinus rhythm with ECV, chemical cardioversion, or catheter ablation.^23,149-151^ Catheter ablation with electrical isolation of the pulmonary veins is currently indicated for the treatment of paroxysmal/persistent AF in patients without graft vascular disease, those with AF-induced tachycardiomyopathy, as well as in those with graft vascular disease and HF to reduce hospitalization and mortality.^146,147^

Association between AF and ICM is less frequent in women than in men, but the high risks of that association have no sex-related differences.^146^ In patients with ACS, the incidence of AF varies from 2% to 23%, being associated with higher in-hospital mortality in the first month and first year, and higher frequency of stroke, with no evidence of sex-related differences. These outcomes are more frequent in individuals who develop AF during hospitalization due to ACS, as compared to those who already had AF previously to the acute ischemic event.^154^ The hemodynamic instability attributed to AF in the presence of ACS should be treated with synchronized ECV. In stable patients, HR control can be obtained with the intravenous use of BB or CCB. The administration of amiodarone is a proper alternative to control ventricular frequency and can favor the reversion to sinus rhythm. Propafenone should not be used in patients with AF and ACS for reversion to sinus rhythm.^146,147^

Recent studies on revascularization in men and women with AF and ACS or chronic coronary syndrome undergoing angioplasty and stent implantation have shown that, in patients with indication for anticoagulation due to AF, the use of dual antiplatelet therapy (acetylsalicylic acid and a P2Y12 receptor inhibitor – clopidogrel, prasugrel, ticagrelor) is recommended for at least one week for those at lower risk of thrombosis and/or higher risk of bleeding, and can last for four weeks, with acetylsalicylic acid suspension after that period.^153^ Dual antiplatelet therapy (anticoagulant + antiplatelet) should be maintained for at least 6 months in patients with AF and chronic coronary syndrome and for 12 months in those with AF and ACS.^146,147,155^

In acute or chronic ICM associated with AF, there is no evidence that investigation or treatment strategies differ in men and women; thus, those strategies should be equally provided to prevent the innumerous and severe complications related to those diseases.

Table 7.1 summarizes the indications for the current treatment of AF and shows the particularities and perspectives related to the female sex.

**Table 7.1:** – Treatment of atrial fibrillation: indications, particularities, and perspectives related to the female sex.

TREATMENT STRATEGIES	INDICATIONS	PARTICULARITIES	PERSPECTIVES
HR CONTROL	1. Control of symptoms; 2. Strategies: drugs isolated or in combination for all patients with AF; 3. Goal: HR < 110 bpm.	1. Women are more symptomatic; 2. Women are less often recruited for clinical trials.	1. Therapeutic strategy should pay more attention to symptoms; 2. Recruit more women for clinical trials.
BB Calcium channel blocker (diltiazem, verapamil) Digoxin Atrioventricular node ablation	1. BB: first choice for any LVEF; 2. Diltiazem/verapamil: patients with LVEF > 40%; 3. Digoxin: patients with LVEF < 40%; 4. Low dose of digoxin associated with a lower risk; 5. Amiodarone and ablation: when the others fail.	1. Women most frequently receive a prescription of digoxin; 2. Atrioventricular node ablation, rather than reversion to sinus rhythm, is most frequently indicated for women.	1. Proper and time-limited digoxin prescription; 2. Proper monitoring of electrolytes.
RHYTHM CONTROL	1. Restore and maintain sinus rhythm for symptom control and quality of life improvement; 2. Strategies: ECV, chemical cardioversion and/or catheter ablation (associated with anticoagulation, HR control and treatment of risk factors for AF).	1. Women have more symptoms and worse quality of life; 2. Fewer women are recruited for clinical trials.	1. Therapeutic strategy should pay more attention to symptoms; 2. Systematic quality of life investigation; 3. Early and proper indication of strategies, including catheter ablation; 4. Recruit more women for clinical trials.
ECV Class I drugs Class III drugs Catheter ablation Maze surgery	1. ECV for patients with AF and hemodynamic instability, including in the presence of ACS; 2. Class I drugs for patients without structural heart disease (including ischemic); 3. Catheter ablation (complete electrical isolation of pulmonary veins) indicated for paroxysmal/persistent AF in patients without graft vascular disease, with AF-induced tachycardiomyopathy, and those with graft vascular disease to reduce hospitalization and mortality; 4. Maze surgery indicated concomitantly with mitral or revascularization surgery.	1. Women receive less indication for ECV; 2. Women are later referred to sinus rhythm reversion strategies, including catheter ablation; 3. There are more reports of complications in women undergoing ablation; 4. Women have a higher frequency of adverse events related to the use of antiarrhythmic drugs (acquired long QT syndrome; sinus node disease).	1. Monitoring of electrolytes and ECG when using antiarrhythmic drugs; 2. Proper design of catheters for ablation in women.
ANTICOAGULATION	1. Reduce thrombus formation and prevent stroke in patients with CHA2DS2VASc ≥ 3 (women) and low risk for bleeding (HAS-BLED < 3).	1. Women higher frequency of stroke, which can be more severe and permanently disabling, with higher mortality; 2. Fewer women are recruited for clinical trials.	1. Periodical stratification of thrombosis and bleeding scores for therapy adequacy; 2. DOACs, at proper doses, are more often indicated; 3. Proper INR control over time, when using warfarin; 4. Recruit more women for clinical trials.
DOACs Warfarin LAA occlusion	1. DOACs are preferable to warfarin, except when AF is associated with moderate/severe mitral valve stenosis or mechanical prostheses; 2. LAA occlusion is indicated when oral anticoagulants are contraindicated; 3. Periodical reassessments of CHA2DS2Vasc and HAS-BLED; 4. When using warfarin: target range for INR = 2.0-3.0, with time in the therapeutic range > 70%. 5. For women with AF and ACS, oral anticoagulants are indicated when CHA2DS2Vasc ≥ 2.	1. Women receive lower doses of anticoagulants; 2. When on warfarin, women remain less time in the INR therapeutic range and have a higher residual risk for stroke; 3. Women have a lower risk of bleeding when on DOACs.	

*ACS: acute coronary syndrome; AF: atrial fibrillation; BB: beta-blocker; DOACs: direct oral anticoagulants; ECV: electric cardioversion; HR: heart rate; INR: international normalized ratio; LAA: left atrial appendage; LVEF: left ventricular ejection fraction.*

### 7.2. Ventricular Arrhythmias: Sudden Death, Prevention, and Treatment

Women’s representation in clinical trials has increased in recent decades, but is still very low, mainly in cardiology, including studies on arrhythmias.^156^ Despite all medical advances, sudden cardiac death remains the major cause of cardiovascular death in patients with ICM,^157^ including acute and chronic IHD and different ventricular arrhythmias.^158^

A recent review on ventricular arrhythmias in acute myocardial ischemia, focused on the role of age and sex, has concluded that the mechanisms responsible for sex-dependent differences in susceptibility to malignant ventricular arrhythmias during acute ischemia are still misunderstood.^159^ In addition, the underrepresentation of women as subjects in clinical trials contributes to the paucity of scientific evidence to develop sex-dependent methods for sudden cardiac death prevention. Despite the scarcity of data on women’s arrhythmia mechanisms, parameters that promote ventricular arrhythmias, such as fibrosis, cardiac hypertrophy, abnormalities of calcium signaling, and electrophysiological changes, differ according to age and sex, stressing the need for further research.^159^ In the major studies of primary prevention, the percentage of women undergoing ICD implantation has ranged from 8% to 29%, indicating the low representation of women, which hinders the assessment of the procedure’s real benefit on that population. In addition, the individual analyses of those studies on the mortality benefit are not appropriate, because those studies had not been designed with statistical power to answer the question.^160^

Regardless of the sex, the prevention and treatment of ventricular arrhythmias of all etiologies involve the control of modifiable RFs, use of AADs, catheter ablation, and ICD use, the last one as primary or secondary prevention.

The ICD therapy for patients with indication is another important point. In 2007, Hernandes *et al.* assessed more than 13 000 patients of the GWTG-HF program (*American Heart Association’s Get With the Guidelines – Heart Failure*) and concluded that the ICD therapy rates differed in black men, black women, and white women, being 27%, 38%, and 44% lower, respectively, as compared to that of white men.^161^ The analysis of the GWTG-HF program in 2012 showed an improvement in those rates and the end of the differences, drawing attention to the need to maintain attention and continuing education around the world.^162^ However, a huge difference persists between the patients who, according to the guidelines, need and those who receive an ICD, especially women.

Regarding individuals with an ICD, there are differences between women and men related to outcomes other than mortality.^163^ A subanalysis of the MADIT-CRT study has concluded that women have a lower incidence of VT/VF, suggesting that, in women, non-arrhythmic sudden death is higher than in men. In addition, the presence of appropriate shock therapy by the ICD was a predictor of death, mainly in women.^164^ In 2022, a study on assessment of ventricular arrhythmias in women (propensity score-matched analysis) was conducted in individuals with ICD. After pairing for the major comorbidities, indications, concomitant therapy, and demographic data, women remained with a lower risk profile for sustained ventricular arrhythmia than men, except for the subgroup of women with CRT and those with LVEF < 30%.^165^

Catheter ablation is an effective therapy to control ventricular arrhythmias, being recommended in several scenarios in the guidelines.^160,166,167^ However, in VT ablation studies, the percentage of women in the population with IHD is low (7-13%). The less often indication for invasive procedures, less often induction of sustained VT, and the smaller number of appropriate shocks are factors that might contribute to that reduced percentage.^166^

### 7.3. Cardiac Resynchronization Therapy

Clinical studies, such as MIRACLE, COMPANION, CARE-HF, MADIT-CRT, RAFT, REVERSE, and MIRACLE, have shown that CRT improves the outcomes in symptomatic patients with reduced LVEF and prolonged QRS duration. Although in those studies the percentage of women has ranged from 17% to 33%, an even greater benefit was demonstrated in women as compared to men (Table 7.2).^160,167^Those studies were performed in ischemic and nonischemic patients, and ischemia was present in 36% to 69% of the patients studied.^168-173^

**Table 7.2:** – Results of clinical studies with cardiac resynchronization therapy according to gender.

Study	N of patients	Inclusion criterion	Randomization	HR of events (95%; P value)
COMPANION^2^	Men: 1025 (67%) Women: 495 (33%)	LVEF ≤ 35% NYHA III-IV QRS ≥ 120ms	ODT vs ODT + CRT-D	HR for death: men: 0.63 (0.4-0.9) women: 0.58 (0.25-1.13)
CARE-HF^3^	Men: 597 (73%) Women: 216 (27%)	LVEF ≤ 35% NYHA III-IV QRS ≥ 130ms LVEDV ≥ 300ms	ODT vs ODT + CRT	HR for death or cardiac hospitalization: men: 0.62 (0.49-0.79) women: 0.64 (0.42-0.97)
MADIT CRT^4^	Men: 1367 (75%) Women: 453 (25%)	LVEF ≤ 30% NYHA II-III QRS ≥ 120ms	ICD vs CRT-D	HR for HF or death: men: 0.76 (0.59-0.97) women: 0.37 (0.22-0.61)
RAFT^5^	Men: 1490 (83%) Women: 308 (17%)	LVEF ≤ 30% NYHA II-III QRS ≥ 130ms	ICD vs CRT-D	HR for death or HF hospitalization: men: 0.82 (0.7-0.95) women: 0.52 (0.35-0.85)
REVERSE^6^	Men: 479 (78.5%) Women: 131 (21.5%)	LVEF < 40% NYHA I-II QRS > 120ms	CRT-on vs CRT-off	HR composite clinical outcome: men: 0.69 (0.43-1.11) women: 0.75 (0.26-2.19)
MIRACLE^7^	Men: 216 (67%) Women: 107 (33%)	LVEF < 35% NYHA III-IV QRS > 130ms	CRT-on vs CRT-off	NYHA. quality of life. exercise capacity; women, but not men, with CRT experienced longer times for first HF hospitalization or death (p = 0.157)

*CRT: cardiac resynchronization therapy; CRT-D: cardiac resynchronization therapy with defibrillator; HF; heart failure; HR: hazard ratio; ICD: implantable cardioverter defibrillator; LVEDV: left ventricular end-diastolic volume; LVEF: left ventricular ejection fraction; NYHA: New York Heart Association;ODT: optimized drug therapy; vs: versus.*

The MIRACLE study has assessed 453 patients with NYHA functional class III or IV HF, LVEF ≤ 35% and QRS duration ≥ 130ms, comparing CRT on *versus* off, and 33% of the patients were women.^173^ Woo *et al*. have analyzed eight pre-specified subgroups in the MIRACLE study, and, when assessing response according to sex, they observed that women receiving CRT had longer times for the first hospitalization or death as compared to the control group, deriving more benefit from CRT than men.^174^

The MADIT-CRT study has assessed 1820 patients with NYHA functional class I or II HF (25% were women), LVEF ≤ 30%, and QRS duration ≥ 130ms. The RAFT study has assessed 1798 patients with NYHA functional class II or III HF (17%, women), LVEF ≤ 30%, and QRS duration ≥ 120ms or ≥ 200ms when stimulated by previous pacemaker. Both studies compared cardiac resynchronization therapy with defibrillator (CRT-D) *versus* ICD and showed a favorable HF-free survival curve in the CRT-D group, with a reduction in the composite outcome of HF and death of 34% and 25%, respectively. In the multivariate analysis in both studies for the outcome death and HF, a marked benefit was observed in women. In the MADIT-CRT study, women receiving CRT-D had a more favorable survival curve than women and men not receiving ICD or even men who received CRT-D. There was a 72% reduction in the risk of death when comparing women with CRT-D *versus* ICD, but there was no difference in the survival curves of men with CRT-D *versus* ICD.^175^

A meta-analysis of the MADIT-CRT, RAFT, and REVERSE studies with 4076 patients, 22% of the female sex, has shown, in the analysis of the outcome HF and death, favorable curves for CRT in women. In addition, for the outcome death, the same results with favorable curve to CRT *versus* ICD in women were found and no difference between men.^176^

A study has assessed 741 patients aged 66 ± 11 years, 33% women, 78% white, LVEF 28 ± 9%, 58% ICM, 75% with LBBB and LV end-systolic volume of 65 ± 30mL/m^2^, undergoing CRT-D implantation, with randomly assigned atrioventricular interval as either fixed at 120ms, echocardiography-determined, or SmartDelay algorithm-programmed. The composite outcome was death and HF hospitalization and >15% reduction in the end-diastolic diameter at 6 months. The response of both sexes to CRT was similar, and the sex disparities in CRT outcomes were explained by the sex differences in HF substrate, treatment, and comorbidities.^177^

Beela *et al*. have investigated to which extent best CRT outcomes could be explained by differences in the baseline characteristics between the sexes. They assessed 1058 patients (24% women) receiving CRT with primary endpoint of all-cause mortality. Women less often had ICM and less fibrosis, more LBBB, and more mechanical dyssynchrony. Considering the baseline characteristics, the survival response was similar in both sexes. The best result found in women was due to the lower ICM rate and smaller scars.^178,179^

The presence of LBBB is a determinant factor of better response to CRT and the same benefit is not observed when LBBB is absent.^21^ A study with 75 079 patients with NYHA functional class III or IV HF, reduced LVEF (≤ 35%), and QRS duration ≥ 120ms, comparing CRT between women and men, has shown benefit in both sexes. The same occurred in the presence of LBBB, although a more attenuated mortality curve was observed in women. In the group without LBBB, women had a better mortality curve than men for even wider QRS complexes (150-159ms).^180^ Women with LBBB responded better than men with narrower QRS complexes and a possible reason might be that women have smaller LV mass and narrower QRS complexes than men (approximately 10ms shorter). Thus, any absolute QRS prolongation in women can correspond to a higher grade of dyssynchrony.^181^

Women have twice the number of major complications related to CRT procedures. This difference can be significant between women and men receiving CRT, the most common complication being pneumothorax/hemothorax. Infection requiring reoperation was more frequent in women. The best predictor of complications in women was a small body mass index (BMI).^182^ The major differences between women and men receiving CRT are shown in Chart 7.1.

**Chart 7.1:** – Major differences between women and men receiving cardiac resynchronization therapy.

1. Women represent 30% or less of the studied populations
2. As compared to men, women more often have: ✓ Nonischemic cardiomyopathy ✓ Left bundle branch block and narrower QRS ✓ Procedural complications ✓ Advanced age ✓ Hypertension and diabetes
3. As compared to men, women less often have: ✓ Atrial fibrillation ✓ Ischemic cardiomyopathy ✓ Reduced ejection fraction

Although therapies, such as ICD and CRT, are beneficial regarding mortality, women receive fewer devices than men (Figure 7.1). This disproportion suggests that women either do not meet the criteria for CRT or are not selected for the studies or not even properly maintained in the studies.^27,183^

**Figure 7.1:** – Distribution of implantable cardiac electronic devices per type and gender. Adapted from Chen-Scarabelli et al.183 *CRT-D: cardiac resynchronization therapy with defibrillator; CRT-P: cardiac resynchronization therapy with pacemaker; ICD: implantable cardioverter defibrillator; PM: pacemaker.*

### 7.4. Recommendations

For stroke risk assessment, the CHA_2_DS_2_VASc score is recommended to identify patients at low risk (CHA_2_DS_2_VASc score = 0 for men and 1 for women) who should not receive oral anticoagulants. Antithrombotic therapy with oral anticoagulants is indicated for patients with AF and CHA_2_DS_2_VASc ≥ 2, but the decision should be individualized.^146,147^

The recommendations relative to management of arrhythmias are the same for both sexes.

## 8. Atherothrombosis in Pregnancy, Contraception, Infertility, Antiphospholipid Syndrome

### 8.1. Introduction

Specific circumstances of women’s biological cycle add risks that contribute to the different course of atherosclerosis and thrombotic disease in women and remain under investigation. This chapter addresses specific topics of reproductive age, such as pregnancy, contraception, infertility, and APS. The APS, prevalent in the female sex, is considered a potential trigger of atherothrombotic disease in women.

### 8.2. Pregnancy

Ischemic heart disease is not common during pregnancy. According to data from the World Health Organization, the AMI rate is 3.34 events per 100 000 pregnancies, non-ST-elevation myocardial infarction (NSTEMI) being the most frequent.^184^

Figure 8.1 shows the major RFs for IHD during pregnancy, in addition to the other RFs, identified in a careful anamnesis and complete physical examination.

**Figure 8.1:** – Major risk factors for ischemic heart disease in pregnancy and additional risk factors. IHD: ischemic heart disease.

Maternal age over 40 years is a progressive RF, thus, for each year of a woman’s life, there is a 20% increase in the risk for myocardial infarction in pregnancy. A contemporary review^185^ has reported on the most frequent mechanisms related to myocardial infarction during pregnancy and puerperium (Figure 8.2).

**Figure 8.2:** – Pathophysiological mechanisms of myocardial infarction during pregnancy and puerperium.

Spontaneous coronary artery dissection accounts for almost half of the causes of myocardial infarction, with a prevalence of 1.81 event per 100 000 pregnancies, being most frequent in the third gestational trimester. The results of pregnancy-associated SCAD have worse prognosis as compared to those of the general population.^51,186^

The management of myocardial infarction due to atherothrombotic disease during pregnancy and after delivery does not differ from that of the general population, even regarding the percutaneous or surgical MRV techniques.^187^ Regarding pharmacological therapy, acetylsalicylic acid at low doses is safe for the pregnancy and fetus,^188^ as is clopidogrel, and should be suspended 7 days before the programmed delivery date.

In cases of SCAD, clinical treatment has been most often indicated, including the use of BBs, which, except for atenolol, can be used in all gestational trimesters.^186^ Absence of residual ischemia or ventricular dysfunction allows a “new” pregnancy with individualized indication.

Table 8.1 summarizes the findings of IHD during pregnancy and puerperium.

**Table 8.1:** – Summary of definition, etiology, pathophysiology, clinical presentation, and prevention of ischemic heart disease during pregnancy and puerperium.

	SCAD	ATHEROSCLEROSIS	VASOSPASM	CORONARY THROMBOSIS	MICROCIRCULATION DISEASE
DEFINITION	Spontaneous rupture of the intimal layer of coronary arteries and intramural hemorrhages in the arterial “false lumen”	Related to traditional and emerging risk factors for cardiovascular disease	Reversible diffuse or focal vasoconstriction of coronary arteries	No atherosclerosis; possible relation to coagulation disorders and hypercoagulability of pregnancy and puerperium; paradoxycal embolism	Mechanisms not yet established, including transient spasms
ETIOLOGY & PATHOPHYSIOLOGY	Disorganization and weakening of coronary walls related to pregnancy hormones	Gestational hypertensive disease, gestacional diabetes, smoking, prolonged use of contraceptives prior to gestation, age > 35 years	Endothelial dysfunction, chronic inflammation, oxidative stress, and smooth muscle hyperreactivity	Hypercoagulability of pregnancy and predisposing genetic factors	Increased vascular reactivity, use of ergot derivatives
CLINICAL PRESENTATION	Varied: from mild chest pain to sudden death. Symptoms of acute coronary syndrome during pregnancy and post-partum	Chest, jaw, and neck pain, fatigue, and nauseas	Varied: from asymptomatic to sudden death, angina, and acute myocardial infarction		
PREVENTION	Avoid emotional stress, hormone therapy, and new pregnancy	Treat the cardiovascular disease risk factors and raise awareness of early diagnosis	Avoid illicit drugs, amphetamines, and alcohol		

*DEAC: dissecção espontânea de artéria coronária. SCAD: spontaneous coronary artery dissection.*

### 8.3. Contraception

Hormonal contraception is well-known to be efficient and safe in healthy women, despite the scarce evidence on its effects on individuals with comorbidities, mainly SAH and cerebrovascular diseases, or history of ischemic events, such as deep venous thrombosis (DVT) and pulmonary thromboembolism (PTE), regardless of the composition or administration route.

In recent decades, there has been an increasing number of young women with CVD, corroborating North-American registries that have shown an 11.5% prevalence of CVD in women aged 20-29 years.^189^ Adolescent or young women often have to face pregnancy without prior counseling about conception planning. Pregnancy, either programmed or not, is a “window of opportunity” to propitiate contraception, for both healthy women and those with some type of comorbidity.^190^

In this scenario, it is worth mentioning that self-identification as transgender occurs in 0.69% of the Brazilian population.^191^ The population assigned female at birth receives no reproductive counseling throughout life, although the use of contraceptives might relieve the ovulatory cycle-related symptoms and reduce undesired pregnancy.^192^

The hormonal contraceptives are classified as combined hormonal contraceptives (CHC - estrogen and progesterone), oral and patch, progestin-only contraceptives (POCs), and long-acting contraceptives (subdermal etonogestrel implant, levonorgestrel-releasing intrauterine system). The copper intrauterine device is a non-hormonal contraceptive. The failure and efficacy of contraceptives can be calculated by the Pearl index, which considers the number of pregnancies/100 women/year^193^(Figure 8.3).

**Figure 8.3:** – Pearl index of the major contraceptive methods. IUD: intrauterine device. Source: adaptado de Curtis et al.*^193^*

Contraceptives in general and the CHC can be associated with atherothrombotic disease. A meta-analysis has shown that the use of CHC represents a risk 1.7-fold higher of myocardial infarction and ischemic stroke.^194^ The effect is due to the action of estrogen, when passing through the liver, to promote changes in the hemostatic pathways that lead to a procoagulant and prothrombotic state. The same does not occur with the POCs, except for medroxyprogesterone acetate, which changes the glycidic and lipidic profiles.^195^

It is worth noting that the presence of RFs for CVD (smoking > 15 cigarettes/day, age > 35 years, obesity, SAH > 160/100mmHg, vascular disease, thrombophilia, history of DVT/PTE, prolonged immobilization, stroke, and myocardial ischemia) significantly increases the risk of thromboembolism in women on hormonal contraceptives. According to the 2015 WHO-MEC eligibility criteria, in such situations, the CHCs, the patches and ring with combined contraceptives, and the injectable combined contraceptives are contraindicated, while the POCs, the subdermal etonogestrel implant, and the levonorgestrel-releasing intrauterine system can be indicated^196^ (Chart 8.1).

**Chart 8.1:** – Use of contraceptives in thrombotic and atherothrombotic diseases.

Contraceptives: Introduction and continuation according to thrombotic and atherothrombotic diseases							
CONDITION	Co-IUD	LNG	Implant	DMPA	POC	CHC	CHC
IHD	Recent or previous	1	2	3	3	2	4
Diagnosed thrombogenic mutation		1	2	2	2	2	4
CVRF	Advanced age, smoking, DM, SAH, dyslipidemia	1	2	2	3	2	4
Ischemic stroke	History of stroke	1	2	3	3	2	4
CVD	Noncomplicated Complicated (pulmonary hypertension, atrial fibrillation, and endocarditis)	1	1	1	1	1	2
		1	1	1	1	1	4
DVT /PTE	History of DVT/PTE without anticoagulation						
	High risk of recurrence	1	2	2	2	2	4
	Low risk of recurrence	1	2	2	2	2	3
	Acute DVT/PTE	2	2	2	2	2	4
	DVT/PTE with anticoagulation for at least 3 months						
	High risk of recurrence	2	2	2	2	2	4
	Low risk of recurrence	2	2	2	2	2	4
	Family history	1	1	1	1	1	2
	Large surgery						
	With prolonged immobilization	1	2	2	2	2	4
	Without prolonged immobilization	1	1	1	1	1	2
	Small surgery without immobilization	1	1	1	1	1	1

*Categories: 1= no use restriction; 2= benefit exceeds potential risk; 3= risk exceeds benefit; 4= unacceptable risk. CHC: combined hormonal contraceptive; Co-IUD: copper intrauterine device; CVD: cardiovascular disease; CVRF: cardiovascular risk factor; DM: diabetes mellitus; DMPA: depot medroxyprogesterone acetate; DVT: deep venous thrombosis; IHD: ischemic heart disease; LNG: levonorgestrel; POC: progestin-only contraceptive; PTE: pulmonary thromboembolism; SAH: systemic arterial hypertension. Source: adapted from Curtis et al.*^193^

In conclusion, although CHCs are the contraceptives most often used worldwide, we emphasize that the subdermal implants and the levonorgestrel-releasing intrauterine system have a smaller impact on atherothrombotic disease. It is worth noting that the selection of a contraceptive method should consider the patient’s intrinsic factors, as well as the safety and efficacy of the contraceptive, in addition to involving a multidisciplinary approach throughout a woman’s reproductive life.^197^

#### 8.3.1. Recommendations

The use of contraceptives in thrombotic and atherothrombotic disease should be guided by the presence or absence of RFs for CVD, in which the following are contraindicated: CHCs, patches and ring with combined contraceptives, and injectable combined contraceptives. In such situations, POCs, subdermal etonogestrel implant, and levonorgestrel-releasing intrauterine system can be indicated.^193^

## 8.4. Infertility

Infertility is a disease characterized by the failure to establish a clinical pregnancy after 12 months of regular and unprotected sexual intercourse. It is estimated to affect 8-12% of couples at reproductive age worldwide. Secondary infertility is the most common type of female infertility, often due to reproductive tract infections. The three major factors influencing the spontaneous likelihood of conception are the time of unwanted non-conception, female partner’s age, and causes related to diseases.^198^

The factors that affect both sexes’ fertility are hypogonadotropic hypogonadism, hyperprolactinemia, ciliary function disorders, cystic fibrosis, infections, systemic diseases, and lifestyle-related factors, while premature ovarian insufficiency, POS, endometriosis, uterine myomas, and endometrial polyps are important causes of female infertility.^199^

In addition to those comorbidities, infertility is often associated with mental disorders, such as depression and anxiety, and has a strong relation to CVD because of the impact of androgenic hormones on CVRFs and on metabolic syndrome.^200^

A meta-analysis,^201^ comparing groups of women of the same age range, with or without infertility, has shown higher frequency of proatherosclerotic metabolic disorders, mainly obesity and increased total cholesterol, LDL-cholesterol, and triglycerides, in infertile women. In that meta-analysis, an isolated study^202^ has shown an increase in IHD, stroke, and HF in infertile women in a follow-up of at least 5 years as compared to healthy women (HR 1.35; 1.16–1.57; p < 0.0001).

Women with POS are at a higher risk for obesity, SAH, glucose intolerance, dyslipidemia, and sleep obstructive apnea. Regarding metabolic disorders, obesity is present in 50% of the cases. Insulin resistance, present in 60-95% of the cases, causes glucose intolerance in 31-35% and type 2 DM in 7.5-20% of those women. However, the lipid profile changes characterized by low HDL-cholesterol levels, high triglyceride plasma levels, and increased LDL-cholesterol levels are the most frequent metabolic change in POS.^203^

On the 20^th^ year of the CARDIA prospective study, the analysis of the images of coronary artery calcifications and measures of the carotid intima-media thickness (IMT) has shown a high risk for subclinical CVD (OR 2.70; 95% CI, 1.31 – 5.60) in the presence of hyperandrogenism and anovulation in POS.^204^ These data have been emphasized in the meta-analysis by Zhang *et al.*, who have reported an increased risk for myocardial infarction, IHD, and stroke (OR 1.66; 95% CI, 1.32 – 2.08) in women with POS.^205^

An equally important cause of infertility is endometriosis, with prevalence of almost 10% in the population and associated with chronic diseases, such as asthma, autoimmune diseases, gynecologic cancers, and CVD.^206^ In endometriosis, there is a chronic inflammatory process mediated by substances, such as intercellular adhesion molecule (ICAM-1), interleukin 1 and 6 (IL-1 and IL-6), tumor necrosis factor (TNF-a), and vascular endothelial growth factor (VEGF), which increase oxidative stress and LDL-cholesterol levels, with subsequent atherogenic effect.^207^

Endometriosis has been shown to associate with well-established RFs for CVD, such as SAH and dyslipidemia, with higher atherogenic profile and increased risk for venous thromboembolism, coronary artery disease, HF, and stroke. A study considering the combination of IHD, HF, and cerebrovascular disease as the primary endpoint has established a positive association of those events with endometriosis (OR 1.24; 95% IC, 1.13 – 1.37), placing endometriosis as a RF for CVD.^208^ These observations were in accordance with a prospective study showing the association of endometriosis with an increased risk for myocardial infarction/ coronary ischemia and surgical and percutaneous MRV.^209^

Fertility therapy is considered a potential RF for hypertensive disorders in pregnancy.^210^ However, a systematic review grouping ACS, stroke, venous thromboembolism, SAH, and DM in women undergoing fertility treatment has shown no increase in the combined events. That analysis, however, with only six very heterogeneous studies, has serious limitations and has not met the statistical criterion to provide more robust evidence.^211^

In conclusion, the reproductive age is a convenient time to estimate the CVR throughout a woman’s life. In that phase of the female biological cycle, attention should be paid to risks and interventions in clinical pathologies not included in traditional CVR scores, stratifying those women differently for effective prevention of CVD.

## 8.5. Antiphospholipid Syndrome

The APS is an autoimmune thrombotic disease that affects mainly young women at the proportion of 5:1.^212,213^ The diagnosis is made in the presence of clinical suspicion of thrombosis in any vascular territory and/or recurring obstetrical complications, such as spontaneous abortions, premature deliveries, and preeclampsia/eclampsia. The APS causes placental insufficiency and restriction of intrauterine growth associated with the persistent presence of antiphospholipid antibodies (aPL): anticardiolipin (aCL), anti-beta2-glycoprotein 1, and/or lupus anticoagulant (LAC) antibodies.^213^

The aPL are strongly associated with stroke in women aged less than 50 years. The presence of aCL and/or LAC in young patients with ischemic stroke or transient ischemic attack, with no concomitant diagnosis of systemic lupus erythematosus, is frequent. In addition, patients with aPL and cerebral ischemia have a higher frequency of multiple events than patients without those antibodies. A prothrombotic state associated with aPL can be a determinant of recurrent ischemia in women with atherosclerosis.^214^

The APS is associated with myocardial infarction in approximately 2.8% of patients affected by the disease. The mechanism of myocardial ischemia in APS is considered acute thrombosis of the coronary arteries, which requires anticoagulation therapy, in contrast to atherosclerotic plaque rupture, which is effectively treated with antiplatelet drugs and stenting*.* Considering the different forms of treatment, the distinction between undiagnosed APS patients is fundamental for therapeutic success and prognosis. Although uncommon, APS should be considered in young women with myocardial infarction, especially in the presence of previous unprovoked thrombosis, lower platelet counts, high partial thromboplastin time, and normal coronary arteries or coronary thrombosis. Anticoagulation should be maintained throughout life, even from the first episode. The role of coronary stenting in such patients requires further studies.^215^

The evidence of significantly elevated titles of different aPLs in the initial phase of myocardial infarction suggests that these antibodies are present before the event and are not secondary to it. The disappearance of high aPL levels after 3 months from myocardial infarction can be due to an effect of absorption or possibly to a cyclical phenomenon similar to that found in other autoimmune diseases. The aPL can be an additional RF for myocardial infarction and should be considered especially in younger patients with no apparent CVRF.^216^

The clinical and laboratory criteria for the APS diagnosis should be present concomitantly in a window inferior to 5 years. The progress in the knowledge on the molecular bases of vascular involvement considers APS a multifactorial disease that develops in genetically predisposed individuals. Several mechanisms contribute to the development of thrombosis in patients with APS, mainly the synergistic effect of autoantibodies with prothrombotic molecules, adhesion receptors, inflammatory mediators, oxidative stress, and intracellular signaling molecules.^217^

The presence of aPLs induces a proatherothrombotic state through the expression of prothrombotic and proinflammatory molecules, including tissue factor and VEGF, and induces oxidative stress and mitochondrial dysfunction in monocytes and neutrophils, in addition to increased formation of extracellular “traps” of neutrophils.^218^

Genetic predisposition has been shown in associations of human leukocyte antigen (HLA) with the disease and the occurrence of aPLs in patients with APS. The major histocompatibility complex genes seem to influence not only the production of autoantibodies, but disease expression as well.^218^ In addition, genetic polymorphisms have been associated with thrombosis in patients with APS, including variants of clotting factors, antithrombotic molecules, and inflammatory mediators.^219^ The aPLs induce genomic and epigenetic changes that support a prothrombotic state.

Epigenetics, defined as changes or modifications in DNA that influence the phenotype without changing the genotype, represents a new mechanism of genetic regulation. Epigenetic and post-transcriptional regulatory mechanisms are altered in autoimmune and cardiovascular diseases, with modifications in DNA methylation, histones, and microRNA activities, changing the expression of genes and proteins.^217^ MicroRNAs affect the immune system and have an important role in the pathogenesis of autoimmune and inflammatory conditions, acting as the major regulators of several gene targets involved in the APS clinical characteristics, such as immune response, atherosclerosis, and thrombosis.^220^ Two microRNAs (miR-19b/miR-20a) are known to function as potential modulators of the tissue factor, the major receptor involved in the development of thrombosis in APS. Thus, a specific signature of circulating microRNAs has been recently identified in patients with APS as potential biomarkers^221^(Figure 8.4).

**Figure 8.4:** – Genomic risk factors of atherothrombosis in antiphospholipid syndrome.*^217^*

Once the patient had a thrombotic event, independently of being venous or arterial, warfarin is the therapy of choice, with a target INR (International Normalized Ratio) between 2 and 3. Heparin, administered acutely at the time of the thrombotic event, can have two benefits: first, complement activation blockade (even if only in a prophylactic dose), and second, anticoagulation. Given the high risk of APS recurrence, long-term anticoagulation is recommended, because that risk can reach 24% with anticoagulation suspension, particularly in cases of systemic lupus erythematosus.^214^

Continuous progress in the knowledge of genomic bases and epigenetic biomarkers boosts clinical pharmacology and provides more safety to the APS therapy. Thus, although larger studies are needed, the new discoveries provide better understanding of the possibility of new models directed to therapeutic options to prevent the APS thrombosis.

Menopause represents a significant transition in a woman’s life and is characterized by the cessation of the menstrual cycle and the subsequent dramatic reduction in the levels of the sexual hormones, estrogen and progesterone. Although testosterone also decreases in postmenopausal women, that reduction is more gradual and the effects of the androgens in a woman’s health after the menopausal transition have not been totally understood. Hormone replacement therapy is a method by which women can control menopausal symptoms during and after that transition. However, there is no sufficient understanding of the exact effects that different hormone replacement therapy forms have on the woman’s physiology, particularly in the context of risk for CVD. The interaction of advanced age and female sex with CVD in addition to the role of hormone replacement therapy and menopause in the CVD progression leads to future questions to address the gaps in the current understanding of the health of elderly women at risk for CVD.^222^

### 8.5.1. Recommendations

Antiphospholipid syndrome should be considered in young women with myocardial infarction, in situations of previous unprovoked thrombosis, lower platelet counts, high partial thromboplastin time, and normal coronary arteries or coronary thrombosis. Anticoagulation should be maintained throughout life, even from the first episode, particularly in systemic lupus erythematosus.^214,215^

## 9. Ischemic Cardiomyopathy in Women

### 9.1. Introduction

In a study involving individuals with ICM undergoing heart transplantation, the assessment of explanted hearts showed an increase in LV mass, myocyte volume, and cellular length in men. This difference was not identified in idiopathic cardiomyopathy, suggesting that gender may influence local myocardial response to ischemic injury.^223^

The evolution after AMI in women is more severe. Higher hospital readmission and mortality rates have been registered in women as compared to men, and they remain higher at 1 (26% x 19%) and 5 years (47% x 36%) after the acute episode.^224^ In addition, the incidence of symptomatic HF after AMI is higher in the female sex, especially in older women. The increase in risk extends beyond the initial episode of AMI. The female sex is an independent predictor of cardiogenic shock, even in AMI with a less severe IHD.^224^

Despite the better LVEF and lower obstructive IHD burden, women with IHD and ICM have lower physical functioning and quality of life, but similar mortality.^126^

There is more information on ICM regarding men, possibly due to its higher frequency. The SAH and DM play a more significant role in women’s risk for HF, with a distinct phenotypic pattern.^7^

Over the past decade, there was an extension of the debate on inequality in CVD care between genders, especially in AMI treatment; however, the underrepresentation of women in clinical trials and the lower optimization of their treatment currently persist. Men are more promptly taken care of and referred for treatment, and more often receive therapy supported by guidelines.^2^ Somehow, this diagnostic-therapeutic limitation may be influencing the unfavorable outcomes in women.

Regarding remodeling in IHD, the differences seem related to the sexual hormones, in addition to the sexual chromosomes and epigenetics. Several genes related to adverse processes of cardiac remodeling, such as activation of macrophages, apoptosis, and lipidic metabolism, have been shown to be in the chromosome.^225^

Female mitochondria have been reported to better tolerate oxygen deprivation and oxidative injury than the male ones, and women seem to be better protected from apoptosis induced by ischemia/reperfusion.^224,226^ High levels of calcium increase the ischemia-reperfusion injury, and estrogen can reduce the levels of calcium before ischemia, leading to fewer lesions in women.^227^

In animal models of infarction and fibrosis due to massive apoptosis, necrosis in the infarcted area is larger, with more collagen, in male mice compared to female ones.^228^ This has been confirmed in trials with human beings, in whom apoptosis after myocardial infarction follows the same pattern, being more prevalent in men.^229^

Thus, although they seem not to improve mortality and hospitalizations, estrogens can protect against oxidative stress and modulate apoptosis; in addition, estradiol can reduce fibrosis by negatively regulating collagen synthesis, although mortality remains high.^224^

In ICM, recent data have suggested that obesity is more frequent in the female population, correlating with worse prognosis as compared to men.^230^

Regarding the HF symptoms, women tend to be more symptomatic, with complaints of dyspnea and orthopnea across the entire functional class spectrum as compared to men.^230^

Several noninvasive methods are available for the diagnosis of women’s ICM and, in many cases, they can aid not only the diagnosis, but the prognostic assessment and therapy definitions as well. The choice of the best modality should consider the diagnostic performance, the gender-related peculiarities, and, sometimes, the presence of special situations, such as pregnancy.

The CCTA has high sensitivity and similar performance in men and women.^231^ The addition of technologies for functional assessment enables the identification of hemodynamically significant lesions and helps define therapies. In addition, it aids the diagnosis of SCAD, which affects mainly women. The CMRI has high diagnostic accuracy in both women and men.^232^ In addition, in cardiomyopathy, different patterns of late enhancement and tissue changes can help the identification of nonischemic etiologies.

Briefly, several noninvasive modalities are available for ICM assessment. Knowing the advantages and disadvantages of each one helps choose the method to be used and interpret the results. It is worth noting, however, that invasive assessment with coronary angiography may be necessary and should not be delayed in case of doubt. In addition, not only in the presence of severe cardiomyopathy and clinical findings, but also of characteristics that increase the probability of ischemic etiology (anginal symptoms or segmental changes), definitive assessments that exclude or identify the ischemic etiology should be considered.

### 9.2. Clinical Treatment

In recent decades, different clinical trials have reported advances in the treatment of HF with reduced ejection fraction (HFrEF). Thus, the major guidelines recommend the use of quadruple therapy, with ACEI/ARB or angiotensin receptor/neprilysin inhibitor (ARNI) + BB + aldosterone antagonist + SGLT2 inhibitors, for the treatment of patients with HFrEF because of the impact on overall and cardiovascular mortality.^233-235^

For patients who persist symptomatic, other classes of drugs, such as hydralazine-nitrate, ivabradine, digoxin, and, more recently, guanylate cyclase stimulators, in addition to CRT, can be added. It is worth noting that, in most clinical studies, ischemia has been the most frequent etiology, ranging from 50% to 70% (Table 9.1). In addition, as a treatment with impact on mortality reduction, the use of ICD is indicated, especially in patients with ICM.^7^

**Table 9.1:** – Studies on the therapy for ischemic cardiomyopathy.

Studies	Drug	Women	Ischemic	Population	Primary endpoint	Result	Interaction M/W	P interaction
**Acetylsalicylic acid**								
Secondary prevention	16 clinical trials of secondary prevention	----	All	17 000 patients High cardiovascular risk	Serious vascular event	HR 0,81 (0.75–0.87) RRR 19%	Men: HR 0,81 (0.73–0.90) Women: HR 0.81 (0.64–1.02)	NS
**BB**								
CIBIS II	Bisoprolol* 10mg/d	20%	50%	2647 patients NYHA FC III-IV LVEF ≤ 35% Follow-up: 16 m	Overall death	HR 0.66 (0.54-0.81) RRR = 34% NNT = 18	Men: HR 0.53 (0.42–0.67) Women: HR 0.37 (0.19–0.89)	NS
MERIT HF	Metoprolol succinate* 200mg/d	22%	65%	3991 patients NYHA FC II-IV LVEF ≤ 40% Follow-up: 12 m	Overall death	HR 0.66 (0.53–0.81) RRR = 34% NNT = 27	In women, there was no reduction in overall death (7.5% x 6.9%)	< 0.05
COPERNICUS	Carvedilol* 25mg 2xd	20%	67%	2289 patients NYHA FC IV LVEF < 25% Follow-up: 11 m	Overall death	RRR = 35% NNT = 15	No significant difference	NS
CIBIS II MERIT HF COPERNICUS					Overall death (grouped data)		Men: HR 0.66 (0.58 to 0.75) Women: HR 0.69 (0.51 to 0.93)	NS
**ACEI/ARB**								
SOLVD	Enalapril* 10mg 2xd	20%	70%	2569 patients NYHA FC II-IV LVEF ≤ 35% Follow-up: 37 m	Overall death	HR 0.84 (0.74-0.95) RRR = 16% NNT = 22	In women, there was no reduction in overall death	< 0.05
CHARM	Candesartan* 32mg/d	32%	67%	2028 patients NYHA FC II-IV LVEF < 40% Follow-up: 33 m	CV death or HFH	HR 0.77 (0.67–0.89) RRR = 27% NNT = 14	No significant difference	
**Aldosterone antagonist**								
RALES	Spironolactone* 25-50mg/d	27%	54%	1663 patients NYHA FC III-IV LVEF ≤ 35% Follow-up: 24 m	Overall death	HR 0.69 (0.58-0.82) RRR = 31% NNT = 10	Men: HR 0.71 (0.60–0.82) Women: HR 0.72 (0.57–0.97)	NS
EMPHASIS	Eplerenone* 25-50mg/d	22%	68%	2737 patients NYHA FC II LVEF ≤ 35% Follow-up: 21 m	CV death or HFH	HR 0.66 (0.56–0.78) RRR = 37% NNT = 13	No significant difference	0.36 (NS)
**ARNI**								
PARADIGM-HF	Sacubitril-Valsartan^†^ 200 mg 2x day	21%		8442 patients NYHA FC II-IV LVEF < 40% / LVEF ≤ 35% Follow-up: 27 m	CV death or HFH	HR 0.80 (0.73–0.87) RRR = 20% NNT = 21	Men: HR 0.80 (0.73–0.87) Women: HR 0.79 (0.66–0.94)	NS
**SGLT2i**								
DAPA-HF	Dapagliflozine* 10mg/day	23%	55%	4744 patients NYHA FC II-IV LVEF < 40% Follow-up: 18 m	CV death or HFH	HR 0.75 (0.65–0.85) RRR = 26% NNT = 21	Men: HR 0.73 (0.63–0.85) Women: HR 0.79 (0.59–1.06)	NS
EMPEROR-Reduced	Empagliflozine* 10mg/day	24%	51%	3730 patients NYHA FC II-IV LVEF < 40% Follow-up: 16 m	CV death or HFH	HR 0.75 (0.65–0.86) RRR = 25% NNT = 19	Men: HR 0.80 (0.68–0.93) Women: HR 0.59 (0.44–0.80)	NS
**Direct vasodilators**								
A-HeFT	Hydralazine 225mg/d + Isosorbide dinitrate* 120mg/d	36%	23%	1050 Black patients NYHA FC III/ IV EF ≤ 35%, or EF < 45% if LVDD > 6.5cm or > 2.9cm/m^2^ Follow-up: 18 m	Primary endpoint: composite score (1^st^ HFH, quality of life, and event-free survival)	RRR = 43% NNT = 25	Men: HR 0.67 (0.49–0.92) Women: HR 0.58 (0.39–0.86)	NS p = 0.806
**If Inhibitors**								
SHIFT	Ivabradine* 5 – 7.5mg 2x day	23%	67%	6558 patients HF LVEF < 35% Sinus rhythm/ Heart rate > 70 Follow-up: 23 m	CV death or HFH	HR 0.82 (0.75–0.90) RRR = 18% NNT = 26	Men: HR 0.84 (0.76–0.94) Women: HR 0.74 (0.60–0.91)	NS
**Digitalis**								
DIG	Digoxin* 0,25mg/d	22%	65%	6800 patients LVEF < 45% NYHA FC II-III Follow-up: 37 m	Overall death No reduction	No mortality reduction	Increased risk of death in women if serum level > 1.2mg/mL	---
**Resynchronization therapy**								
MADIT-CRT	CRT-D *versus* ICD alone	25%	50%	1820 patients LVEF < 30% NYHA FC I-II QRS > 30ms Follow-up: 4.5 years	Overall death or nonfatal HF event	HR 0.66 (0.52–0.84) Higher benefit in women	Men: HR 0.76 (0.59–0.97) Women: HR 0.37 (0.22–0.61)	p = 0.01

*ACEI: angiotensin-converting-enzyme inhibitor; ARB: angiotensin-receptor blockers; ARNI: Angiotensin Receptor/Neprilysin Inhibitor; BB: beta-blockers; CRT: cardiac resynchronization therapy; CRT-D: cardiac resynchronization therapy associated with defibrillator; CV: cardiovascular; FC: functional class; HF: heart failure; HFH: HF hospitalization; HR: hazard ratio; ICD: implantable cardioverter defibrillator; LVDD: left ventricular diastolic diameter; LVEF: left ventricular ejection fraction; m, months; SGLT2i: sodium-glucose cotransporter 2 inhibitor; NNT: defined for primary endpoint/all-cause death during the entire follow-up; NS: nonsignificant; NYHA: New York Heart Association; RRR: relative risk reduction.*

Despite recent advances, treating women with ICM is challenging. The first challenge refers to women’s underrepresentation in the major clinical trials of HFrEF. While women represent approximately 50% of the patients in HFpEF trials, their representation in HFrEF trials ranges from 20% to 30% only. Thus, the gender-specific differences are limited, based on analyses of subgroups, and, thus, should be cautiously interpreted.^7^

The therapy for ICM and its major supporting studies are as follows:

1. **Beta-blockers:** a meta-analysis involving the major studies on BB in HFrEF has shown that the reduction in mortality occurs similarly in women and men. Sub-analyses have shown that bisoprolol improved the survival of 515 women studied in the CIBIS-II study (HR 0.37; IC 95%, 0.19 – 0.69), carvedilol reduced the composite outcome of death and hospitalization in 469 women with LVEF < 25% in the COPERNICUS sub-study (HR 0.23; IC 95%, 0.07 – 0.69), and metoprolol succinate reduced the length of hospital stay due to HF in women with LVEF < 25% (p = 0.004), but did not reduce mortality alone.^236-238^
2. **ACE inhibitors:** studies with ACEI, such as SOLVD and CONSENSUS, have shown an impact on mortality reduction of patients with HFrEF and NYHA functional class II/III and IV, respectively; however, when assessing women alone, that benefit was not observed.^239^
3. **Angiotensin-receptor blockers:** data from a subanalysis of the CHARM trials (candesartan) have shown no sex-related difference in the primary endpoint of the study on cardiovascular death and HF hospitalization.^240^
4. **Angiotensin receptor-neprilysin inhibitors:** analysis of subgroups of the PARADIGM-HF study has suggested no difference between women and men regarding the primary endpoint of cardiovascular death or HF hospitalization. Recently, a real-world study involving 427 patients (29% women) has shown similar tolerability to the use of ARNI in women and men. ^241^
5. **Aldosterone antagonists:** analysis of subgroups from studies involving spironolactone (RALES) and eplerenone (EMPHASIS) has revealed that the impact on overall and cardiovascular death/HF hospitalization, respectively, occurred in women and men, with no statistically significant difference.^7^
6. **SGLT2 inhibitors:** the studies assessing dapagliflozine (DAPA-HF) or empagliflozine (EMPEROR-Reduced) in patients with HFrEF have shown that the addition of that new class of drugs to triple therapy causes a reduction in the composite outcome of cardiovascular death and HF hospitalization, and that benefit is similar in men and women^242,243^(Table 9.1).

Regarding additional therapies, the subanalysis of the A-HeFT trial, which investigated the hydralazine-nitrate association, and of the SHIFT study, which assessed ivabradine in patients with HFrEF, has shown a reduction in the primary endpoint in women and men. Regarding digoxin use, the DIG study has shown no mortality reduction in the total population, but it is worth noting that, in the subgroup of women with serum digoxin outside the therapeutic range (higher than 1.2mg/mL), the mortality rate was higher. Thus, lower doses and strict control of digoxin serum levels in women are recommended when that medication is indicated.^244-246^

As previously described, most clinical trials on HFrEF have shown equivalent efficiency of the medicamentous treatment for women and men, and prognosis-modifying drugs should be used in both sexes. However, it is worth noting that those observations result from the analyses of subgroups from studies, and, thus, should be cautiously considered.

Table 9.1 summarizes data from the major clinical trials in ICM and their results comparing men and women.

### 9.3. Devices and Advanced Heart Failure

The advances in electric and mechanical devices have provided substantial benefits to patients’ symptoms, hospitalization, and results, with strong evidence in both sexes.

The range of devices includes ICD, CRT, and defibrillators of CRT. However, recent data have shown that women are less likely to receive ICD, and, when they do, their rates of implantation-related complications, such as pneumothorax and infection, are higher.^230^

### 9.4. Implantable Cardioverter Defibrillator

As previously mentioned, women less often have IHD as the etiology, and, as remodeling response, less fibrosis, and a lower rate of ventricular arrhythmias, resulting in fewer sudden cardiac deaths. A meta-analysis assessing studies involving patients (n=7229) with ischemic (74%) and nonischemic cardiomyopathy, 22% of women, has shown that the benefit was significantly higher in men (HR 0.67; 95% CI, 0.58-0.78, p < 0.001) than in women (HR 0.78; 95% CI, 0.57-1.05, p = 0.1).^247^

However, even after adjusting for age and comorbidities, the female sex had lower probability of receiving an ICD as compared to the male sex.^248^

It is worth noting that women have higher rates of complications related to device implantation, such as pneumothorax, infection, bleeding, and tamponade.^249^

However, because of the less fibrotic profile and even the structural and anatomical characteristics, studies have suggested that women respond more favorably to CRT, which results in improvement of symptoms, quality of life, LVEF, and mortality. Data from the MADIT-CRT study, comparing CRT-D to implantation of ICD alone, have shown a higher benefit in women as shown in Table 9.1 (p = 0.01).^170^

### 9.5. Advanced Heart Failure

Ventricular assist devices are already well implemented in the practice of advanced HF services as a bridge or a destination therapy. Women are more likely to be hospitalized due to advanced HF, but less often receive a ventricular assist device. In a recent study analyzing the EUROMACS registry, 966 patients (151 women) have been included. Characteristically, at the time of implantation, women showed worse INTERMACS 1 and 2 profiles (51.7% *versus* 41.6% in men) and experienced more complications, such as major bleeding (p = 0.001), arrhythmias (p = 0.02), and right ventricular insufficiency (p < 0.001), with worse 1-year survival (75.5% *versus* 83.2%).^7^

Heart transplantation continues to be the gold-standard for the treatment of advanced HF, with only 25% of recipients of the female sex, usually because of height, weight, blood type, and immune panel mismatch. In addition, women have more complications after heart transplantation, such as rejection mediated by antibodies and cardiac allograft vasculopathy.

### 9.6. Recommendations

#### 9.6.1. Clinical Management and Indications for Advanced Therapies

**Class I/B –** Female patients with ICM and HF with reduced ejection fraction should receive pharmacological treatment according to HF guidelines.^233-235^

**Class I/B –** Female patients with ICM and HF with improved ejection fraction should receive pharmacological treatment according to HF guidelines.^233-235^

**Class I/B –** Female patients with ICM and HF with preserved ejection fraction (LVEF>50%) should receive pharmacological treatment according to HF guidelines.^233-235^

**Class I/C –** Female patients with ICM and HF with reduced ejection fraction, refractory to pharmacological treatment according to HF guidelines, should be referred for cardiac resynchronization.^233-235^

**Class I/C –** Female patients with ICM and advanced HF, refractory to pharmacological and nonpharmacological treatment according to HF guidelines, should be considered for heart transplantation.^233-235^

## 10. Percutaneous Coronary Intervention

### 10.1. Introduction

Recent studies have reported a significant increase in the mortality rates of acute coronary disease in young women (< 55 years).^250^ Despite the increasing evidence of sex-related differences in baseline RFs, coronary anatomy and function, symptoms, comorbidities, treatment efficacy, and outcomes in ACSs, the mechanisms of those differences are not completely known.^67^ These gaps of knowledge are due to women’s underrepresentation in clinical trials; thus, the scientific community should be encouraged to change this paradigm.

Despite the general benefit of MRV, the female sex has been consistently associated with an increased risk of bleeding and vascular complications associated with PCI, evidencing the need to consider the major biological differences, such as the size of the vessels of percutaneous accesses and the prevalence of nonobstructive coronary artery disease in women, in addition to the need to apply the recommendations of current guidelines.^251^

As compared to men, women submitted to PCI are older and have higher prevalence of kidney failure, anemia, and DM. As compared to men, women with ACS have higher mortality and less often undergo the recommended treatments, such as early invasive strategy and antithrombotic therapy.^252^

Clinical factors, such as advanced age, kidney failure, cardiogenic shock, and use of major introducers, were specifically identified as predictors of the risk of bleeding in women. However, the female propensity for bleeding persists in addition to those RFs. Sex-specific mechanisms regarding BMI, puncture site anatomy, platelet biology, and PCI-related pharmacotherapy may play an important role.

### 10.2. Vascular Access to Cardiac Catheterization and Percutaneous Coronary Intervention in Women

Mason Sones performed the first selective coronary angiography in 1958 through dissection of the brachial artery. In 1967, Judkins and Amplatz developed the technique of femoral artery puncture and appropriate catheters for that technique, which is still widely used because it provides excellent access in cases of complex angioplasties that require larger introducers and the assessment of bypasses and grafts of patients with previous surgical MRV.^253^

The radial access, described in 1989 by Campeau, is a more complex technique that requires higher ability and experience, in addition to a longer learning curve. A higher proportion of women have more tortuous radial arteries of smaller caliber as compared to men. Patients with chronic kidney disease, DM, low BMI, and elderly have a higher rate of unsuccessful radial access.^254,255^

The femoral artery access has predominated for decades, because of its significant viability and reproducibility. The radial artery access, however, has shown to be efficient to reduce bleedings and other vascular complications, especially in patients with ACS, thus, becoming the access of choice recently.^253^

In addition, the radial access use can be associated with a better quality of life and lower costs as compared to the femoral one.^253,255,256^ However, the magnitude of the benefit associated with radial access can vary widely, depending mainly on the patient’s risk for femoral access complications. Thus, it is fundamental to ensure the preferential use of the radial access in patients at higher risk for complications from the vascular access, such as women.^256^

### 10.3. Diagnosis

#### 10.3.1. Coronary Angiography

Women’s epicardial coronary arteries are significantly smaller than those of men, even after adjusting for age, body structure, and LV mass. The discrepancy in the size of the coronary arteries between women and men is considered an important base of the sex-related differences in the outcomes of the coronary artery disease, despite the smaller atherosclerotic plaque volume of women.^67,77^

The important implication of the smaller size of the coronary arteries is that the comparatively lower atherosclerotic plaque and thrombus burden can result in obstructive disease. This may be a potential reason for the higher incidence of sudden cardiac death in younger women, despite their lower atherosclerotic plaque burden. In addition, smaller vessels can represent a higher risk of re-stenosis.^77^

In addition, the baseline and hyperemic myocardial blood flow, as assessed on PET, is typically higher in women as compared to men. Thus, the smaller diameter of female epicardial coronary arteries and their higher baseline myocardial blood flow are suggested to significantly increase in conditions of endothelial shear stress in women and can explain some sex-related differences in susceptibility to coronary artery disease^67,77^ (Figure 10.1).

**Figure 10.1:** – Diagnosis of ischemic heart disease. Creation of the authors. ICUS: intracoronary ultrasound; OCT: optical coherence tomography.

#### 10.3.2. Intravascular Imaging

The characteristics of the atherosclerotic plaque, the vascular response to coronary stent implantation, and the vascular endothelium can be assessed with a close correlation with the histopathological findings by use of intravascular imaging methods performed during cardiac catheterization. The current technologies of intracoronary imaging include mainly intracoronary ultrasound (ICUS), OCT, and near infrared spectroscopy (NIRS).^67^

The ICUS enables the precise atherosclerotic plaque burden quantification with axial resolution of 70-200 mm and penetration > 5mm. Using that method, the PROSPECT study (*Providing Regional Observations to Study Predictors of Events in the Coronary Tree*) could evaluate the effect of sex on the coronary artery disease extent and characteristics and confirm significant differences between the sexes. Women have less extensive IHD. The morphology and composition of the plaque in women have smaller necrotic nucleus and calcification, for a similar atherosclerotic plaque burden, although smaller minimum luminal area and more lesions with minimum luminal area < 4.0mm^2^, as compared to men.^257^

The OCT has a lateral resolution of 10-20 mm and enables the characterization of plaques and thrombi, but with lower penetration, hindering the precise assessment of the atherosclerotic plaque burden. However, it has high resolution and power to detect the atherosclerotic plaque morphology, helping identify vulnerable plaques or thin-cap fibroatheroma. In addition, OCT enables the clarification of some mechanisms of nonobstructive coronary disease aggravation, such as dissection, and corroborates findings that differentiate coronary disease between sexes*.*^258,259^

The NIRS uses an optical fiber catheter with an absorption pattern specific for lipids and other plaque components, improving the accuracy of the identification of vulnerable atherosclerotic plaques.^67^

Together, these three intravascular imaging methods allow us to know some differences in the characteristics of atherosclerosis and IHD between the sexes. In women, the plaque burden is lower, erosion is more prevalent than rupture as the mechanism of coronary artery disease aggravation, and there is a higher concentration of cholesterol crystals and calcification in ruptured atherosclerotic plaques.^67,259^

#### 10.3.3. Invasive Tests with Measuring Guide

The diagnostic and therapeutic routine has concentrated on stenosis of the epicardial coronary arteries, although evidence in recent decades has established the concept that epicardial obstructive stenosis is not a mandatory condition to cause ischemic symptoms of stable coronary disease. Innumerous publications have shown that symptomatic women are more likely than men to have nonobstructive IHD and coronary microvascular dysfunction, accounting for up to 40% of the cases.^260^

***10.3.3.1. Fractional Flow Reserve***

The measurement of FFR consists in the use of angioplasty guidewires with a sensor on their tips, which were developed to measure the pressure and blood flow across a coronary artery stenosis in the catheterization lab. The FFR measures the proximal (aortic pressure) and distal (guidewire pressure) pressures in coronary artery stenosis under maximum blood flow and creates a pressure ratio, representing the proportion of flow through the stenosis. For accurate FFR measures, the pressures should be obtained during hyperemia. Maximum blood flow (hyperemia) is most commonly induced by use of intravenous (140mcg/kg/min) or intracoronary (right coronary artery 50-100 mcg, left coronary artery 100-200mcg in *bolus*) adenosine. The ratio between distal coronary pressure and aortic pressure (as registered by the guidewire) during maximum hyperemia is called FFR. The normal value is 1, while values < 0.80 are associated with provocable ischemia with a precision over 90%.^261^

For certain grades of stenosis, the FFR values in female patients tend to be much higher than those in male ones. Considering that female patients are at a higher risk of in-hospital mortality and post-PCI adverse results, the importance of measuring FFR should be emphasized especially in women to avoid unnecessary PCI.^262^

***10.3.3.2. Instantaneous Wave-free Pressure Ratio***

An index derived from the resting pressure, independently of adenosine, has been developed and tested as surrogate for FFR. Using wave intensity analysis, the period of diastole in which the balance between the pressure waves of aorta and the distal microcirculatory reflection was the “wave-free period” was determined to meet the criteria of FFR as having a minimum constant resistance. The diastolic pressure/aortic pressure (Pd/Pa) during the wave-free period (75% in diastole finishing 5ms before the R wave) is called instantaneous wave-free pressure ratio (iFR). It has been shown that, for iFR cut-off points of > 0.93 or < 0.86, there was a strong correlation with normal and abnormal FFR values (using 0.80 as the FFR cut-off point). In the ADVISE II study, iFR was compared to FFR in 690 intermediate stenoses. As compared to FFR (< 0.80), the iFR cut-off of 0.89 classified correctly 83% of the stenoses. The iFR classified correctly those stenoses outside the iFR grey zone from 0.85 to 0.94 with 92% of concordance. Thus, the iFR-FFR hybrid approach for intermediate stenosis can be assessed without the hyperemic stimulus in 65% of the patients.^262,263^

#### 10.3.4. Functional Tests

Microvascular coronary vasoconstriction can also be assessed by use of functional tests with intracoronary acetylcholine, which produces coronary vasodilation in the presence of healthy endothelium or paradoxical vasoconstriction in the presence of endothelial dysfunction. Incremental doses are administered for 3 minutes until the response is produced or the target dose is reached.

The positive response for epicardial coronary spasm is a focal or diffuse coronary artery diameter reduction > 90% (as compared to the relaxed state). Patients experience the reproduction of angina and ischemic changes on ECG. These patients are considered to have vasospastic angina.

The positive response for microvascular vasoconstriction is the absence of epicardial coronary spasm (no diameter reduction or reduction < 90%). Patients experience the reproduction of angina and ischemic changes on ECG (ST-segment depression or elevation).

The negative response to the acetylcholine test is the absence of epicardial coronary spasm (no diameter reduction or reduction < 90%), with no angina and no ischemic changes on ECG (Figure 10.2).

**Figure 10.2:** – Management of INOCA. Based on the EAPCI Consensus on INOCA.*^77^* CFR: coronary flow reserve; FFR: fractional flow reserve; IFR: instantaneous wave-free pressure ratio; LVEDP: left ventricular end-diastolic pressure.

If the test with acetylcholine is negative, there is still the option of testing with intracoronary adenosine. The CFR is calculated based on the coronary flow velocity (CFV), which is the ratio between peak CFV and baseline CFV. A CFR < 2.5 indicates endothelium-independent coronary microvascular dysfunction. These patients with abnormal CFR will also be considered to have changes in the microcirculation.^260^

## 10.4. Percutaneous Treatment of Coronary Atherothrombotic Disease in Women

### 10.4.1. Revascularization for Chronic Coronary Syndromes

The objective of MRV in chronic coronary syndromes is to relieve angina rather than reduce mortality. Thus, when considering the medicamentous or PCI treatment options, the higher incidence and frequency of angina in women should be considered. In a subanalysis of the COURAGE study, there was no significant difference in the effect of treatment on the major results between women and men. However, women assigned for PCI have shown a higher benefit, as compared to men, with a reduction in HF hospitalization and in the need for future MRV.^264^

The ISCHEMIA clinical trial has shown that women, as compared to men, have a higher burden of angina symptoms, although their coronary disease is less extensive, and ischemia is less severe.^265^

***10.4.1.1. Disease of the Left Main Coronary Artery***

Until recently, coronary artery bypass grafting (CABG) was the treatment recommended for left main coronary artery (LMCA) disease, but PCI has been more and more adopted to treat the LMCA disease.

In the EXCEL trial,^266^ women undergoing PCI for unprotected LMCA showed a trend towards worse outcomes, a finding related to associated comorbidities and increased periprocedural complications. However, sex was not an independent predictor of post-MRV adverse outcomes, the same conclusion of the NOBLE study.^267^

***10.4.1.2. Total Chronic Occlusion***

Total chronic occlusions represent an important subgroup of coronary lesions and are found in as much as 18% of diagnostic angiographies.

A well-succeeded PCI for total chronic occlusion is known to be associated with the symptomatic relief of angina, improvement in LV function and quality of life, as well as mortality reduction. However, several studies have shown that, in women, a well-succeeded PCI for total chronic occlusion has not been associated with a reduced risk for cardiovascular mortality or major adverse coronary events (MACE) as compared to clinical treatment alone, which differs from that in men, who have a significant reduction in the rate of MACE after well-succeeded PCI for total chronic occlusion.^268^

### 10.4.2. Revascularization for Non-ST-Elevation Myocardial Infarction

In patients with NSTEMI, the initial invasive approach is associated with better results and a lower rate of combined outcomes of death, myocardial infarction or refractory angina in 4 to 6 months of follow-up, especially in patients at high risk; however, women undergo PCI less often, especially the younger ones.^269^ The benefits of an invasive approach are more pronounced in patients with elevated biomarkers or other findings of high risk, regardless of sex.^270^A flowchart summarizes the strategy choice and the invasive assessment time (Figure 10.3).^65^

**Figure 10.3:** – Invasive assessment of non-ST-elevation myocardial infarction. Based on the Brazilian Society of Cardiology Guidelines on Unstable Angina and Non-ST-Elevation Myocardial Infarction – 2021.*^65^* AMI: acute myocardial infarction; CABG: coronary artery bypass grafting; CHF: congestive heart failure; CPA: cardiopulmonary arrest; DM: diabetes mellitus; HF: heart failure; LVEF: left ventricular ejection fraction; NSTEMI: non-ST-elevation myocardial infarction; PCI: percutaneous coronary intervention.

### 10.4.3. Revascularization for ST-Elevation Myocardial Infarction

The female sex has been associated with late presentation to the hospital and a delay in primary PCI, which has been partially attributed to women’s atypical symptoms. Women are less likely to undergo invasive therapies for STEMI, possibly because of their higher number of comorbidities and fragility of admission. In addition, there are fewer female patients with obstructive coronary disease on angiography. These delays and disparities in healthcare contribute to increase in-hospital mortality in women with STEMI. Once the treatment is initiated, the rates of procedural success, postprocedural epicardial flow, myocardial perfusion, and ST-segment resolution are similar in both sexes after primary PCI.^269^Figure 10.4 shows the reperfusion strategy selection with the times that should be pursued to improve the results of STEMI care.^271^

**Figure 10.4:** – Selection of reperfusion strategy. Based on 2018 ESC/EACTS Guidelines on Myocardial Revascularization.*^271^* NSTEMI: non-ST-elevation myocardial infarction; PCI: percutaneous coronary intervention.

***10.4.3.1. Strategies to Approach Multivessel Coronary Artery Disease***

The patients who benefit the most from revascularization of nonculprit arteries include those with large myocardial area at risk and those without significant comorbidities that would increase the MRV risk. Data from the COMPLETE study support complete MRV and the treatment of nonculprit lesions either at the time of primary PCI for STEMI or after the index hospitalization. However, the analyses of subgroups of that study have shown a trend towards a change in the sex-related effect (p interaction = 0.08), and no benefit regarding cardiac mortality with complete MRV for women.^272^

***10.4.3.2. Cardiogenic Shock***

Cardiogenic shock, HF and right ventricular infarction occur more frequently in women with STEMI as compared to men. In addition, women have lower BP and cardiac output as compared to men in cardiogenic shock.^65^However, several studies, such as the CULPRIT-SHOCK trial, have shown that sex had no influence on mortality according to different MRV strategies.^273^

### 10.4.4. Considerations on Device During Percutaneous Revascularization

***10.4.4.1. Drug-eluting Stents***

In the past two decades, there was an important advance in the technology of drug-eluting stents, such as drug optimization, polymers, and stent design, supporting the safety and efficacy of those most recent devices. Angiographic studies of drug-eluting stents assessing in-stent late loss have shown similar degrees of neointimal hyperplasia in women and men, suggesting that the drug-eluting profiles are effective in both sexes.^274^

***10.4.4.2. Drug-Coated Balloon***

The PCI with drug-eluting stent is the most common mode of revascularization for IHD. However, in-stent restenosis and thin vessels are known limitations. Drug-coated balloon is a balloon coated with antiproliferative agents encapsulated in a polymeric matrix, which are released in the wall during insufflation and contact with the intima. Leaving no metal in the vessel treated is beneficial for thin vessels, in-stent restenosis, and intermediate and distal vessels, where distal stents can be disadvantageous for future MRVs.^275-277^

***10.4.4.3. Rotational Atherectomy and Intravascular Lithotripsy***

Rotational atherectomy and intravascular lithotripsy are indicated for calcified lesions to prevent stent underexpansion, which is associated with higher restenosis rates. In women, rotational atherectomy is associated with a higher risk of periprocedural complications, but with long-term MACE-free adjusted overall survival rates similar to those in men. However, intravascular lithotripsy has angiographic complications, safety, and efficacy similar in women and men.^65,278^

## 10.5. Adjunct Pharmacological Therapy

Although randomized studies have suggested similar benefits of adjunct antithrombotic therapy for coronary disease in both sexes, women less often receive the treatment recommended in the guidelines.^269,279,280^ Vulnerability for bleeding is one reason. Women, particularly the elderly and low-weight ones, have higher risk of hemorrhagic complications, which can be related to a smaller body distribution volume, lower glomerular filtration rates, and differences in the activities of hepatic enzymes.^269^ To mitigate that risk, in addition to the radial access, it is worth noting the need to adjust the doses of anticoagulants and antiplatelet drugs to weight and renal function, whenever pertinent, and to monitor the activated clotting time every 30-60 minutes during the intervention, administering supplementary doses of heparin, if needed, to ensure proper anticoagulation levels.^281^

Regarding oral antiplatelet therapy, the dual antiplatelet therapy with acetylsalicylic acid and a P2Y12 inhibitor (clopidogrel, prasugrel or ticagrelor) is recommended to prevent post-intervention thrombotic events.^155^ Clinical presentation, risk of bleeding, ischemic risk, and comorbidities should guide the selection of the P2Y12 inhibitor type, the ideal time to administer its loading dose, and the dual antiplatelet therapy duration, regardless of sex.^155,281^ Women, however, have a higher risk of bleeding with any inhibitor.^282,283^ Strategies to minimize that risk include to shorten the dual antiplatelet therapy duration, discontinue the acetylsalicylic acid, maintaining monotherapy with a P2Y12 inhibitor after a short period of dual antiplatelet therapy, or de-escalation of the P2Y12 inhibitor, from prasugrel or ticagrelor to clopidogrel.^284,285^ The acetylsalicylic acid suspension after a short peri-intervention period (at hospital discharge or after 1 week) has proven effective to reduce bleedings in individuals with AF requiring oral anticoagulation and undergoing PCI.^286^ Sex-specific analyses of safety and effectiveness of those strategies can help select or personalize the ideal antiplatelet regimen for women.

## 10.6. Knowledge Gaps

• Prevalence of obstructive and nonobstructive coronary disease in the female sex, in different clinical scenarios, acute and chronic.
• Clinical validation of the women-specific reference values for the invasive coronary physiological assessment tests (hyperemic and non-hyperemic), regarding the differences in coronary caliber, myocardial mass, and microvascular resistance between sexes.
• Different impact of the intravascular imaging method on women, considering its diagnostic role in cases of MINOCA and its value to optimize PCI in the female sex, which typically involves thinner vessels and higher risk of bleeding.
• Selection of the ideal MRV method (percutaneous *versus* surgical) in women with LMCA or multivessel lesion, reassessing the influence of sex in current therapeutic outcomes.
• Optimization of the diagnostic and treatment strategies for the different etiologies of MINOCA (and differential diagnoses), advancing in the pathophysiological understanding, and identification of sex-specific RFs and predictors of recurrence of SCAD and Takotsubo cardiomyopathy.
• Particularities of PCI in women in complex scenarios, such as cardiogenic shock, calcified lesions, bifurcations, and chronic coronary occlusions.

## 10.7. Recommendations

**Table:** 

Recommendation	Class of recommendation	Level of evidence	Reference
For patients requiring coronary revascularization, treatment decision should be based on clinical indication, regardless of sex, race, or ethnicity	I	B	155
For patients with ACS undergoing PCI, radial rather than femoral access is preferred to reduce the risk of death, vascular complications, and bleeding	I	A	155
For patients with stable ischemic disease undergoing PCI, radial access is recommended to reduce bleeding at the access site and vascular complications	I	A	155
FFR-guided PCI should be considered for patients with multivessel disease undergoing PCI	IIa	B	271
Urgent/immediate invasive strategy is indicated for patients with ACS without ST-segment elevation with refractory angina and/or hemodynamic and/or electric instability (no severe comorbidity or contraindication to those procedures)	I	A	65
In STEMI, reperfusion therapy is indicated for all patients with symptom onset <12 h and persistent ST-segment elevation	I	A	271
All patients should be stratified and classified as at high, intermediate, or low risk for bleeding	I	B	65

*ACS: acute coronary syndrome; PCI: percutaneous coronary intervention; FFR: fractional flow reserve; STEMI: ST-elevation myocardial infarction.*

## 11. Surgical Intervention, Heart Transplantation

### 11.1. Myocardial Revascularization

Several factors, such as epidemiological, anatomical, and/or operative technique-related ones, make the results of women’s CABG less favorable as compared to those of men.^287^Several series have shown that, at the time of surgical indication, women tend to have a higher surgical risk, with more comorbidities associated, such as SAH, DM, HF, ACS, in addition to a higher respiratory function impairment.^288-291^

O’Connor *et al*. have shown that coronary artery diameters are directly proportional to body surface, being, thus, usually smaller in women than in men.^292^Since the 1980s, the analyses of the *Coronary Artery Surgery Study* (CASS) have shown that the coronary artery diameters had a direct influence on the immediate results of CABG.^293^

When stratified by groups, mortality in patients with large coronary arteries (2.5 – 3.5mm) was 1.5%, increasing to 4.6% in those with intermediate diameter (1.5 – 2.0mm), and reaching 15.8% in individuals whose coronary arteries had a mean diameter of 1mm.^292^ In addition to the coronary artery diameters, the quality and fragility of the grafts influence the decision on the surgical tactic to be used and increase the complexity of the surgical techniques.^294^

Although the 2021 ACC/AHA/SCAI guidelines recommend the preferential use of arterial grafts,^155^ several studies have shown the underuse of those grafts in female patients.^295,296^ Because of the smaller caliber of the mammary arteries and radial arterial grafts, there is an underuse of arterial grafts, which could increase the proportion of revascularized vessels with grafts of higher late permeability.^297^ The less favorable characteristics of both arterial and venous grafts in women can lead to a higher predisposition to early thrombosis and trend to spasms, increasing the chance of unfavorable results.^292,298^ Similarly, although the importance of complete MRV in patients with multivessel lesions has been well established, several studies have shown that women receive a smaller number of grafts, being, thus, not completely revascularized.^299^

The benefits of CABG with no cardiopulmonary bypass, off-pump CABG, are still controversial. Some studies have shown similar mortality between the sexes, while others do not confirm those findings.^300,301^ However, in patients with more advanced cerebrovascular disease, kidney and/or respiratory dysfunction, that technique might be safer. However, because of the anatomical and technical aspects already mentioned, the off-pump CABG in women is frequently more challenging than in men.

In recent years, there was a significant increase in the number of women submitted to CABG. However, in most studies, the male sex still predominates in the samples analyzed. There is evidence in the literature that, as compared to men, women have worse outcomes and prognosis after CABG. According to Attia *et al.*, the long-term survival after CABG is worse in women than in men, even after adjusting for differences in RFs.^288^

Previous analyses have identified the female sex as an independent RF for intra-operative and long-term mortality after CABG, even after adjusting for some variables, such as more advanced age and increased prevalence of comorbidities.^290^ Both in the EXCEL and NOBLE studies, women had a higher prevalence of RFs, such as DM, SAH, and dyslipidemia. However, the anatomical complexity of their coronary lesions was smaller.^302^

Vaccarino *et al*. have conducted a meta-analysis of studies related to several aspects of CABG: ART TRIAL, CORONARY TRIAL, GOPCABE TRIAL, and PREVENT TRIAL. After the first 5 years from surgery, female patients had worse cardiac and cerebrovascular outcomes, but similar mortality as compared to men. These differences are not evident after the age of 75 years (the difference of these results between the sexes is inversely associated with age). Those studies have reported that women, as compared to men, have a more difficult postoperative recovery from CABG. Between the 6^th^ and 8^th^ postoperative weeks, women reported more physical complaints and side effects than men in the same period. In addition, they had lower physical functioning, more depressive symptoms, and were twice more likely to be readmitted to hospital. These differences remained substantial and statistically significant even after multivariate analysis. In addition, those authors have observed that CABG had a much higher impact on the women’s mood as compared to that of men.^303^

Despite the gender-related differences, the indications for CABG are well established in current recommendations and guidelines.^155^ Healthcare providers should know the female sex-related particularities to promote differentiated strategies to improve the healthcare to women undergoing CABG.

Historically, women have a worse postoperative course as compared to men regarding CABG.^293,304,305^Several aspects have been analyzed to identify factors with a negative impact. Of the non-modifiable differences, the smaller body surface most frequently found in the female sex has been studied and considered a factor that can negatively contribute to postoperative results, with no conclusion.^293,306^ An important anatomical characteristic regards the aspects of the female coronary circulation, with thinner coronary arteries of smaller caliber, increasing the complexity of the anastomosis technique and the risk of early graft occlusions.^293,302,307^

Because of their smaller caliber, fewer arteries receive grafts and, thus, some myocardial areas remain unprotected, increasing the risk of recurrent ischemic events, ventricular dysfunction, and HF in the mid- and long-term postoperative period. The risk of perioperative myocardial infarction and early graft occlusion contributes to a marked higher in-hospital mortality as compared to that observed in men, affecting the late results.^305^

Other important data related to unfavorable postoperative course concerns the use of arterial grafts, which is less often in women.^307,308^ Even the isolated use of the left internal thoracic artery to revascularize the anterior descending artery, considered gold-standard in CABG, is less frequent in female patients.^305,306^ The potential benefit of arterial grafts can be lost in patients of higher risk, which would explain why women with worse baseline risk profiles would not receive as many arterial grafts as men.^302^

Female patients referred for surgical treatment are more likely to be older, have significantly more severe comorbidities, such as SAH, DM, hyperlipidemia, peripheral arterial and venous disease, and more advanced clinical status of coronary disease with unstable angina, post-infarction angina, HF, and indication for urgent CABG.^309^

Thus, the diagnosis of coronary insufficiency, as well as the indication and referral for surgical treatment occur later in women, with consequent negative impacts on the postoperative results.^310^ Women most often than men have immediate postoperative complications, but the rates of reoperation due to bleeding are lower in female patients^311^ (Figure 11.1).

**Figure 11.1:** – Multiple factors that influence the postoperative results of women and men. DM: Diabetes mellitus; SAH: systemic arterial hypertension.

Regarding technical aspects, Puskas *et al.*^300^ have reported that, in off-pump CABG, the postoperative results were similar in men and women, considering that the strategy reduces gender disparity in postoperative clinical outcomes. In addition, especially in patients at higher risk, the female sex was not an independent RF of mortality after off-pump CABG.

However, according to a meta-analysis by Gaudino *et al*.,^302^ variations in the surgical technique used in procedures with cardiopulmonary bypass neither improved the results in women nor reduced mortality, considering the difference between sexes in off-pump CABG.

Regarding the surgical techniques used in CABG, the international guidelines strongly recommend the use of the left internal thoracic artery for the left anterior descending artery, a higher number of arterial grafts for ischemic multivessel coronary disease, and complete CABG to obtain better short- and long-term postoperative results after CABG. However, even following the recommendations of the guidelines, women are likely to less often, 14% to 22%, undergo CABG.^155,312-317^

#### 11.1.1. Surgical Myocardial Revascularization in Women – Recommendations

**Table:** 

Recommendation	Class of recommendation	Level of evidence	Reference
Complete revascularization with minimum manipulation of the aorta	I	B	314,315
Use of the left mammary artery for the anterior descending artery	I	B	316
Use of the radial artery as the second option for the territories with arterial obstruction >80%	I	B	313
Mammary artery skeletonization to prevent infection	I	B	317

## 11.2. Heart Transplantation

Several factors should be considered when choosing a donor, such as concordance between the donor-recipient sexes, sizes, and heights, BMI, pulmonary hypertension, and, more recently, predicted total heart mass (pHM) assessment. It is reasonable to use the pHM calculation to help the donor-recipient correspondence. A donor-recipient pHM of 0.86 or greater predicts a good prognosis; a pHM between 0.86 and 0.7 can be considered for individual cases; and a pHM lower than 0.7 can be associated with adverse posttransplant results.^318,319^

The donor-recipient sex correspondence has recently attracted considerable attention.^320,321^ There has been a slight increase in transplants from women to women and a corresponding decline in transplants from women to men, maybe because of the shorter survival observed after donor-recipient sex mismatch transplantations. This can be explained by immune differences^291^ or the donor-recipient size matching.^320^

The sex distribution of heart transplant recipients has changed over time, with an increase in the proportion of female recipients from 19.3% in 1992-2000, to 22.4% in 2001-2009, and to 25.6% in 2010-2018. The reasons why women have reduced representation among heart transplant recipients have not been clearly identified; one would be the HF development and consequent indication for transplant at a more advanced age.^321-323^

Women are at higher risk of sensitization, pregnancy being one of the major RFs. Transfusions, ventricular assist devices, and previous transplantation are other RFs. Preformed antibodies can cause hyperacute rejection and increase the risk of posttransplant rejection, in addition to predisposing patients to the development of cardiac allograft vasculopathy.^324,325^ Anti-HLA antibodies should be detected to reduce the risk of hyperacute rejection. The use of virtual crossmatch, from a data bank, in pre-transplant assessment replaces prospective crossmatch, can increase the pool of donors and reduce the testing time, in addition to helping decide which sensitized patients require treatment before the procedure.^324-326^

Panel reactive antibody should be performed in all candidates for transplant. When the panel reactive antibody is elevated (≥ 10%), an additional assessment is recommended.^319^ Desensitization of the recipients can be performed prior to transplantation with venous infusion of immunoglobulin, plasmapheresis isolated or in combination, rituximab, and, in certain very special cases, splenectomy.^319^ Targeting at the humoral response components, desensitization can be used as a therapeutic option to increase the number of donors and, thus, increase the likelihood of transplantation for sensitized patients (Figure 11.2).

**Figure 11.2:** – Therapeutic options for desensitization and increase in the number of donors. Source: Adapted from Bacal F. 3ª Diretriz Brasileira de Transplante Cardíaco.*^327^*

The treatment of hyperacute rejection should be initiated immediately after the diagnosis, even in the operating room. According to the current International Society for the Heart and Lung Transplantation (ISHLT) guidelines, the treatments include:^319^ (1) mechanical circulatory support; (2) high dose of venous corticosteroids; (3) plasmapheresis; (4) venous immunoglobulins; (5) rituximab; (6) cytolytic immunosuppressive therapy; (7) eculizumab; (8) cyclosporin, tacrolimus with increased target levels and inhibitors of the metabolic cycle (mycophenolate); (9) venous inotropic and vasopressors; and (10) heparin.

Cardiac allograft vasculopathy remains highly prevalent and one of the major causes of late death after heart transplantation.^319^ Severe acute cellular rejection (ISHLT 3R) diagnosed by use of endomyocardial biopsy should be treated even in the absence of symptoms or evidence of cardiac allograft dysfunction. The significant RFs for the development of cardiac allograft vasculopathy include donor’s death due to stroke and diagnosis of recipient’s ICM and retransplantation. The female sex of both the donor and the recipient has been reported to reduce the risk of developing cardiac allograft vasculopathy.^328^ Previous studies disagree and have shown either a higher incidence of cardiac allograft vasculopathy in procedures with female donors or recipients or no sex-related difference regarding the development of cardiac allograft vasculopathy.^329-331^

Heart transplantation for peripartum myocarditis remains relatively infrequent. Some studies have shown that, in its late stage, the incidence of cardiac allograft vasculopathy is similar to that of transplantations performed in women due to other etiologies.^332^

Malignancy after heart transplantation continues to be a significant cause of morbidity and mortality in recipients. Data from the ISHLT registry have shown a cumulative prevalence of all types of malignancy after heart transplantation in adults of 16% in 5 years and of 28% in 10 years. Skin cancer continues to be the most common posttransplant malignancy.^328^ Rudasill *et al.*, in a recent analysis of the United Network for Organ Sharing (UNOS) registry, have shown no association of donor’s malignancy with the recipient’s 10-year survival.^333^ However, a recipient’s history of pretransplant malignancy has been associated with an increased risk of posttransplant malignancy, especially skin neoplasms. However, that incidence was smaller in women. It is worth noting the need for rigorous surveillance of skin and breast malignancy in the female population and of colon malignancy in the general population.^334^

According to the 2021 ISHLT report on Adult Heart Transplantation, female donor-recipient had a higher 1-year mortality as compared to the male donor-recipient combination (HR 1.16). The male/female combination increases the 5-year mortality (HR 1.1). However, the female donor-recipient combination is associated with a lower risk (HR 0.90) as compared to the male donor-recipient combination in 5 years.^323^ In addition to size mismatch, those sex-related differences in survival suggest that hormonal influences on immune response can be associated.^319^ Hypertrophic cardiomyopathy and congenital heart disease were associated to an increased 1-year mortality. Other variables associated with increased 5-year mortality, conditional on 1-year survival, include restrictive, ischemic, and retransplant cardiomyopathy.

Indistinctly between genders, the recipient’s panel reactive antibodies and the ischemia duration are associated with 1-year mortality, while the recipient’s chronic characteristics are associated with 5-year mortality. Thus, chronic sequelae of DM, vascular and chronic kidney disease can affect the long-term posttransplant results. Other variables associated with 5-year mortality, conditional on 1-year survival, include recipient’s age, BMI, pulmonary vascular resistance, renal function, donor’s age, and transplant center’s volume of surgeries.^323^

The female gender is a strong predictor of waitlist mortality in heart transplantation.^326-335^ However, sex has not been shown to be a significant or important variable associated with posttransplant mortality, with few interactions of gender as a mortality predictor, although women live longer than men.^336^

Despite the increased number of women submitted to heart transplantation, they continue underrepresented. Female recipients have different baseline characteristics when compared to male recipients, and posttransplant survival is equivalent in women and men after adjusting for recipient and donor’s characteristics.^321^

Women tend to have better long-term survival than men after heart transplantation, with smaller risk of developing coronary artery disease, cardiac allograft vasculopathy, and malignancies, but a higher risk of antibody-mediated rejection.^330^

Early posttransplant mortality (< 1 year) is mainly due to graft failure, infection, and multiple organ failure, while late posttransplant mortality (> 5 years) is mainly due to malignancy, graft failure, and cardiac allograft vasculopathy.^330^

Overall survival is emphasized as the primary endpoint after heart transplantation. However, health-related quality of life is important in heart transplant recipients. A recent study using the Short Form with 36 items (SF-36) for quality-of-life assessment, in accordance with the World Health Organization, has shown that 22% of the survivors were employed at 1 year and almost 33% at 2 years following heart transplantation. Neurological and nephrological complications have a negative impact and are important predictors of quality of life after heart transplantation. Gender is a factor related to social determinants of health, as well as race and ethnicity, in addition to socioeconomic and education levels.^337-339^

In a study cohort to develop and validate machine learning models to increase the predictive accuracy of mortality after heart transplantation, with 18 625 patients, the models demonstrated good predictive accuracy of the posttransplant results, and one of those models was the Index for Mortality Prediction after Cardiac Transplantation (IMPACT) score. However, only 27% were female patients.^340^

Chronic humoral rejection and cardiac allograft vasculopathy are the major causes of late morbidity and mortality in patients submitted to heart transplantation, and the diagnosis by use of noninvasive methods has low sensitivity.^327^

### 11.2.1. Heart Transplant in Women – Recommendations

The recommendations for heart transplant, which can be found in the guidelines on heart transplant, do not differ between sexes.^327^

## 12. Rehabilitation in Women’s Ischemic Cardiomyopathy

The indication of cardiac rehabilitation in IHD is a consensus in international^341^ and national^95^ guidelines. After ACS and percutaneous or surgical MRV and for stable angina, referral for cardiac rehabilitation should be part of the medical prescription. Its efficacy to improve the quality of life and to reduce modifiable RFs and mortality, as well as to prevent readmissions due to new events, has been well established.^342^

Studies have shown that women less often adhere to cardiac rehabilitation than men. In a sample with 44% of women, only 14.3% used cardiac rehabilitation after AMI as compared to 22.1% of men.^343^ Referral to cardiac rehabilitation at hospital discharge has been related to lower mortality, especially in women and ethnical minorities.^344^ Among women, the likelihood of death, HF or stroke 5 years after AMI is higher than among men, independently of age.^345^

One of the major barriers to cardiac rehabilitation use is the lack of medical referral for it. In women, cardiac rehabilitation referral and incentive by the physician regarding the importance of cardiac rehabilitation as part of the treatment is a strong predictor of admission to programs.^346^ Other important barriers are lack of social support, low physical functioning, unemployment, more advanced age, fear of physical exercise, high burden of family responsibilities, multiple comorbidities, absence of reimbursement of the sessions, limited accessibility, and diversity of programs.^344,347^

During the initial assessment for admission to the cardiac rehabilitation program, it is necessary to interrogate women’s specific questions that are related to CVD in that population. Preeclampsia, DM, gestational hypertension, preterm birth, and early menopause are some important additional RFs to be addressed, as are menopause symptoms, presence of urinary incontinence, musculoskeletal questions, and fatigue perception.^348^

To increase the individualization and optimization of the aerobic exercise prescription, a CPET is ideally indicated, with detected ventilatory thresholds being the intensity limits used in the absence of ischemia and/or changes of other indicators, such as early plateau or oxygen pulse drop (Figure 12.1). When those variables and ST-segment changes are present, they should be considered in the prescription and maintained below the ischemic threshold. When CPET is not available, percentages of peak HR predicted or measured on ET, talk test, and subjective perception of exertion might help the prescription of physical exercise intensity (Table 12.1).^349^ The aim is to achieve moderate intensity with impact on mortality and quality of life, but the prescription should always be individualized (Table 12.2).

**Figure 12.1:** – Prescription of aerobic exercises based on ventilatory thresholds 1 and 2 of the cardiopulmonary exercise test. AT: anaerobic threshold; RCP: respiratory compensation point.

**Table 12.1:** – Alternative methods for the prescription of aerobic exercises.

METHOD	DESCRIPTION
Perceived exertion (Borg scale)	Exercise practice with self-perception of exertion
Talk test	Exercise practice at moderate and controlled intensity, at which the individual can talk without pausing for a breath
Percentages of maxHR predictor	Exercise prescription based on maxHR percentage target HR= percentage ×maxHR $\mathrm{maxHR}=208-(0.7x\text{ age }{)}^{*}\text{ or }220-{\text{ age }}^{**}$
Reserve HR (Karvonen)	target HR= resting HR+%×(maxHR− resting HR)

HR: heart rate; maxHR: maximum heart rate; *Tanaka formula, **Karvonen formula.^349^

**Table 12.2:** – Aerobic exercise intensity levels.

Intensity	%VO_**2**_ max	%maxHRpred	%RHR	MET* (absolute)	Borg Scale
Very mild	< 37	< 57	< 30	< 2	< 9
Mild	37-45	57-64	30-40	2-3,9	9-11
Moderate	46-64	65-76	40-60	4-6	12-13
Intense	65-91	76-96	60-90	6,1-8,8	14-17
Very intense	> 91	> 96	> 90	> 8,9	> 17

*Modified from ACSM*^349^ *MET: metabolic equivalent; %VO_*2*_ max: percentage values of maximum oxygen consumption; %maxHRpred: percentage values of maximum heart rate predicted for age; %RHR: percentage values of reserve heart rate; Borg scale: linear scale of perceived exertion, grading from 6 to 20.

Women have lower gains in physical functioning after cardiac rehabilitation programs, independently of the initial physical functioning levels. Thus, prescription adjustments are necessary throughout the program, because physical functioning improvement is the main factor for the effectivity of the program regarding its benefits.^347^

In parallel with physical functioning, the reduction in muscle strength associates with an increase in CVD and mortality. Women have less strength in the hand grip test. Khadanga *et al*.^347^ have emphasized that additional attention should be paid to the prescription of resistance training, with intensity of up to 80% of 1 maximal repetition and aimed at improving leg strength and walking capacity to meet the demands of daily life activities. In addition, those authors have suggested the inclusion of high-intensity interval aerobic trainings (90-95% of maximum HR) based on a small, randomized study from their group, in which, as compared to moderate training (70-85% of maximum HR), oxygen consumption had a higher increase (23% *versus* 7%).^348-350^ However, further studies are needed to support those findings. Women are more likely to experience musculoskeletal pain and fatigue. To elaborate a program with shared objectives and to offer methods of complementary exercises, such as Yoga, dance, Tai-Chi, might prevent drop out from cardiac rehabilitation programs. However, further studies are necessary to confirm the effectiveness of the methods to reduce CVR, morbidity, and mortality.^348^

The following situations, more prevalent in the female population, should be considered when addressing cardiac rehabilitation and its role in IHD:

**Spontaneous coronary dissection**: The lack of consensus on exercise practice after SCAD and its possible relation to intense exertion cause uncertainty among health professionals and patients regarding resuming physical activities. However, a Mayo Clinic registry with 354 patients with SCAD, 96% of whom were women, has confirmed the benefits of participating in cardiac rehabilitation programs, without clinical complications.^351^ A dedicated cardiac rehabilitation program for SCAD survivors was designed at Vancouver General Hospital*,*^352^ with improvement in physical functioning and psychological stress, as well as a reduction in the need for MRV during follow-up. Thus, referral to cardiac rehabilitation centers should be encouraged, addressing the concerns about exercise that many patients have after the first SCAD episode.

**MINOCA**: Differently from the unequivocal consensus on the role of cardiac rehabilitation in obstructive coronary disease, data on the efficacy and safety of cardiac rehabilitation in coronary microvascular dysfunction are scarce. However, studies performed in women with microvascular angina have been able to show improvement in physical functioning, metabolic variables, quality of life, and myocardial perfusion after different periods of a cardiac rehabilitation program, and, thus, further studies should be encouraged.^353,354^
